# Synthesis, Absolute Configuration, Biological Profile and Antiproliferative Activity of New 3,5-Disubstituted Hydantoins

**DOI:** 10.3390/ph17101259

**Published:** 2024-09-24

**Authors:** Mladenka Jurin, Ana Čikoš, Višnja Stepanić, Marcin Górecki, Gennaro Pescitelli, Darko Kontrec, Andreja Jakas, Tonko Dražić, Marin Roje

**Affiliations:** 1 Laboratory for Chiral Technologies, Division of Organic Chemistry and Biochemistry, Ruđer Bošković Institute, Bijenička Cesta 54, 10000 Zagreb, Croatia; mladenka.jurin@irb.hr (M.J.); darko.kontrec@irb.hr (D.K.); andreja.jakas@irb.hr (A.J.); 2 NMR Centre, Ruđer Bošković Institute, Bijenička Cesta 54, 10000 Zagreb, Croatia; ana.cikos@irb.hr; 3 Laboratory for Machine Learning and Knowledge Representation, Division of Electronics, Ruđer Bošković Institute, Bijenička Cesta 54, 10000 Zagreb, Croatia; visnja.stepanic@irb.hr; 4 Institute of Organic Chemistry, Polish Academy of Sciences, 01-224 Warsaw, Poland; marcin.gorecki@icho.edu.pl; 5 Department of Chemistry and Industrial Chemistry, University of Pisa, Via Giuseppe Moruzzi 13, 56124 Pisa, Italy; 6 Laboratory for Biocolloids and Surface Chemistry, Division of Physical Chemistry, Ruđer Bošković Institute, Bijenička Cesta 54, 10000 Zagreb, Croatia; tdrazic@gmail.com

**Keywords:** 3,5-disubstituted hydantoins, preparative HPLC separation, NMR analysis, absolute configuration, CD/VCD, antiproliferative activity, in silico biological profiling

## Abstract

Hydantoins, a class of five-membered heterocyclic compounds, exhibit diverse biological activities. The aim of this study was to synthesize and characterize a series of novel 3,5-disubstituted hydantoins and to investigate their antiproliferative activity against human cancer cell lines. The new hydantoin derivatives **5a**–**i** were prepared as racemic mixtures of *syn*- and *anti*-isomers via a base-assisted intramolecular amidolysis of C-3 functionalized β-lactams. The enantiomers of *syn*-**5a** and *anti*-hydantoins **5b** were separated by preparative high-performance liquid chromatography (HPLC) using *n*-hexane/2-propanol (90/10, *v*/*v*) as the mobile phase. The absolute configuration of the four allyl hydantoin enantiomers **5a** was assigned based on a comparison of the experimental electronic circular dichroism (ECD) and vibrational circular dichroism (VCD) spectra with those calculated using density functional theory (DFT). The antiproliferative activity evaluated *in vitro* against three different human cancer cell lines: HepG2 (liver hepatocellular carcinoma), A2780 (ovarian carcinoma), and MCF7 (breast adenocarcinoma), and on the non-tumor cell line HFF1 (normal human foreskin fibroblasts) using the MTT cell proliferation assay. In silico drug-like properties and ADMET profiles were estimated using the ADMET Predictor ver. 9.5 and the online server admetSAR. Eighteen new 3,5-disubstituted hydantoins were synthesized and characterized. The compound *anti*-**5c** showed potent cytotoxic activity against the human tumor cell line MCF7 (IC_50_ = 4.5 µmol/L) and the non-tumor cell line HFF1 (IC_50_ = 12.0 µmol/L). In silico analyzes revealed that the compounds exhibited moderate water solubility and membrane permeability and are likely substrates for CYP3A4 and P-glycoprotein and have a high probability of antiarthritic activity.

## 1. Introduction

**Figure 1:**
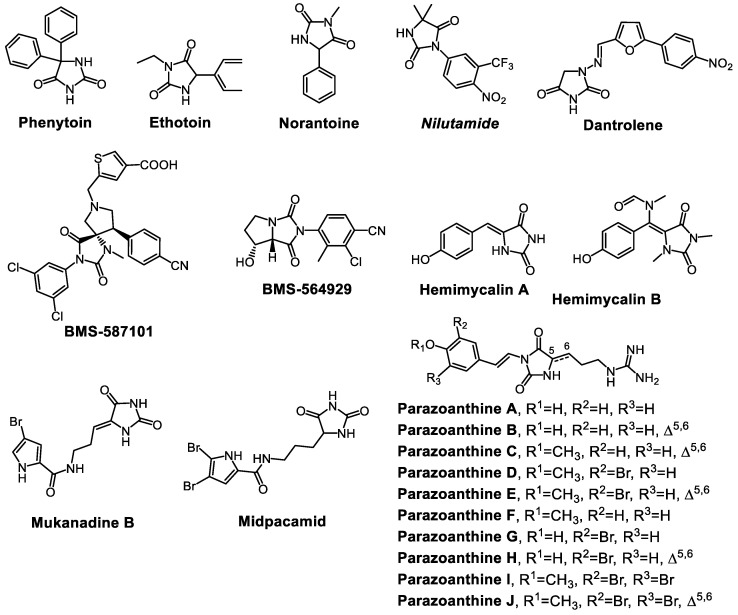
Chemical structures of some biologically active compounds and natural products containing a hydantoin moiety.

Imidazolidine-2,4-diones, commonly referred to as hydantoins, are five-membered heterocyclic compounds characterized by the presence of two nitrogen atoms at positions one and three, and two carbonyl functions at positions two and four within the hydantoin ring [1,2,3,4]. The hydantoin moiety is an important structural element found in various bioactive medicinal compounds. The biological activity of these compounds depends on the nature of the substituents at positions N-1, N-3, and C-5 of the hydantoin ring. The hydantoin core structure is found in marketed drugs for the treatment of epileptic seizures (phenytoin, ethotoine, and norantoine) [5,6], and metastatic prostate cancer (nilutamide) [7,8], muscle relaxants drugs, and drugs for the prevention of malignant hyperthermia (dantrolene) [9] (Figure 1). BMS-587101 is a potent and orally active antagonist of leukocyte function associated antigen-1 (LFA-1) [10], while BMS-564929 is a highly potent, orally active, nonsteroidal tissue selective androgen receptor (AR) modulator [11]. In addition, hydantoin derivatives exhibit diverse biological and pharmacological activities in medicine, such as antimicrobial [12,13], antiviral [14,15,16], anticonvulsant [17,18,19,20,21], antitumor [7,22], antiarrhythmic [23,24], antihypertensive [25], antithrombotic, anti-inflammatory [26], antidiabetic [27,28], and agrochemical applications, such as herbicidal and fungicidal [29]. Hydantoins also have an inhibitory effect on some enzymes (human aldose reductase and leucocyte elastase) [30,31]. Furthermore, the hydantoin moiety is frequently found in natural products, predominantly isolated from a diverse range of marine organisms and bacteria [32]. For example, hemimycallins A and B (Figure 1) were extracted from the marine sponge *Hemimycale arabica* [31], mukanadine B (Figure 1) was extracted from the marine sponge *Agelas nakamurai* [33], midpacamide (Figure 1) was extracted from the Fijian sponge *Agelas mauritiana* [34], and parazoanthines A–J (Figure 1) were extracted from the Mediterranean anemone *Parazoanthus axinellae* [35,36]. From the viewpoint of organic synthesis, hydantoins have been widely used as precursors for the synthesis of some optically pure natural and unnatural amino acids [37]. In addition, the optically pure hydantoins have been widely used as chiral auxiliaries [38] and metal ligands in asymmetric catalysis [39]. 

Numerous methods for the synthesis of the hydantoin derivatives have been described in the literature [10,40]. Hydantoins can be prepared either in solution by classical methods, on the solid phase, under microwave irradiation, by mechanochemistry, or by the continuous flow method [10,40,41,42,43,44,45,46,47,48,49,50,51,52]. Zhang et al. prepared enantiomerically pure hydantoins by the cyclization of optically pure α-amino amides with triphosgene [41]. The same group reported that the use of 1,1′-carbonyldiimidazole (CDI) led to a racemization of the stereogenic center. They suggest that the imidazole carbamate intermediate is responsible for the observed racemization with CDI in this type of reaction. Chen et al. recently prepared a library of enantiomerically pure 3,5-disubstituted hydantoins by a direct cyclization of the corresponding ureas with sodium hydride [42]. The obtained hydantoins were isolated in good to high yields and with excellent enantioselectivities. Tanwar et al. obtained 3,5-disubstituted hydantoins, including the anticonvulsant ethotoin, in a one-pot synthesis of α-amino methyl ester hydrochlorides with carbamates [43]. This reaction led to the formation of the corresponding ureido derivatives, which subsequently cyclized under basic conditions to give the corresponding hydantoins in good yields. Liu et al. used a simple activation strategy mediated by trifluoromethanesulfonic anhydride to prepare highly substituted chiral hydantoins from simple Boc-protected dipeptides in a single step under mild conditions [44]. Mehra and Kumar performed a base-promoted intramolecular amidolysis of C-3 functionalized azetidin-2-ones, prepared from racemic *cis*-β-lactam carbamates and amines, with sodium methoxide to afford the 3,5-disubstituted hydantoins [45]. The basic condition led not only to the formation of the desired hydantoins but also to the formation of their dimers. Rajić et al. synthesized 3,5-disubstituted hydantoins by a base-induced cyclization of the corresponding *N*-(1-benzotriazolecarbonyl)-L- and D-amino acid amides and investigated their antiviral and antitumor activity [14]. Of all of the hydantoins synthesized, cyclohexyl-5-phenyl hydantoin showed antitumor activity against HeLa and MCF7 cell lines but also cytotoxic effects on human non-tumor fibroblasts WI 38, whereas 3-benzhydryl-5-phenyl hydantoin showed moderate antitumor activity against HeLa, MCF7, MiaPaCa-2, H 460, and SW 620 but no cytotoxic effects on normal cells. In 2016, Konnert et al. reported the mechanochemical reaction of α-amino methyl esters with CDI or with substituted alkyl isocyanates to give a series of 3,5 disubstituted hydantoins, including ethotoin, with or without poly(ethylene glycol)s as grinding assisting agents [46]. Konnert et al. also developed a mechanochemical solvent-free method to prepare various 3,5-disubstituted hydantoin derivatives from amino esters or dipeptides via a CDI-mediated one-pot/two-step cyclization reaction involving unsymmetrical urea or carboxy-imidazolyl dipeptide ester intermediates [47]. Recently, Mascitti et al. optimized the mechanochemical synthesis of highly functionalized 3,5-disubstituted hydantoins from α-amino esters by liquid-assisted grinding (LAG) in the presence of various poly(ethylene) glycols [48]. The first reported solid-phase synthesis of 3,5-disubstituted hydantoins was described by De Witt and coworkers [49]. A series of polystyrene Wang resin, ester-linked amino acids were exposed to isocyanates to give resin-linked α-ureido acids, which were then treated with 6 M hydrochloric acid. The strongly acidic conditions promoted *N*-cyclisation and the simultaneous cleavage of hydantoin from the resin. Colacino et al. reported a microwave-assisted solid-phase synthesis of 3,5 hydantoin derivatives in which resin-bound amino acids with a free *N*-terminal moiety reacted with phenyl isocyanate [37]. The ureido derivatives formed were then simultaneously cyclized and released from the resin in the presence of triethylamine to afford the corresponding hydantoins. Colacino et al. discussed [50] innovative mechanochemical methods for the synthesis of hydantoin-based active pharmaceutical ingredients (APIs) and emphasized their environmentally friendly nature and advantages over conventional synthetic approaches. The authors emphasize the improved yields, the simplified reaction conditions and the lower environmental impact and advocate mechanochemistry as a sustainable alternative in drug development. Furthermore, the hydantoins can also be synthesized using flow chemistry techniques. Monteiro et al. [51] presented a new continuous flow method for the synthesis of hydantoins using the Bucherer–Bergs reaction. The authors successfully optimized the reaction conditions and achieved high yields for a variety of substrates. They also investigated the selective N(3)-monoalkylation of the hydantoins, which opens up possibilities for further modifications and potentially new therapeutic applications. Vukelić et al. [52] synthesized hydantoins from commercially available amines using a semi-continuous flow process involving photooxidative cyanation followed by carbon dioxide introduction at elevated temperatures. Using this method, various hydantoins were produced in good yields without the need for extensive purification steps, demonstrating an efficient and sustainable approach to hydantoin synthesis.

In the present work, a series of novel 3,5 disubstituted hydantoins **5a**–**i** were prepared as racemic mixtures of *syn*- and *anti*-isomers via a base-assisted intramolecular amidolysis of C-3 functionalized β-lactams [45]. Each synthesized hydantoin **5a**–**i** has two stereogenic carbon centers, one at the C-5 position of the hydantoin ring and the other in the side chain. The absolute configuration of the four isolated enantiomers of allyl hydantoin **5a** was determined by a comparison of experimental and DFT calculated ECD and VCD spectra. All of the synthesized compounds **5a**–**i** were evaluated in vitro for their antiproliferative activity against liver hepatocellular carcinoma (HepG2), ovarian carcinoma (A2780), breast adenocarcinoma (MCF7), and untransformed human fibroblasts (HFF-1). In silico drug-like properties and ADMET (Absorption, Distribution, Metabolism, Excretion, and Toxicity) profiles of the synthesized hydantoin compounds **5a**–**i** were estimated using the commercial software ADMET Predictor ver. 9.5 [53] and the free online server admetSAR [54]. The biological activities of each hydantoin **5a**–**i** were predicted by the commercial software PASS 2020 [55] and the open access web server SwissTargetPrediction [56].

## 2. Results and Discussion

### 2.1. Synthesis and Separation of Syn- and Anti-(±)-3,5-Disubstituted Hydantoins ***5a**–**i***

**Scheme 1:**
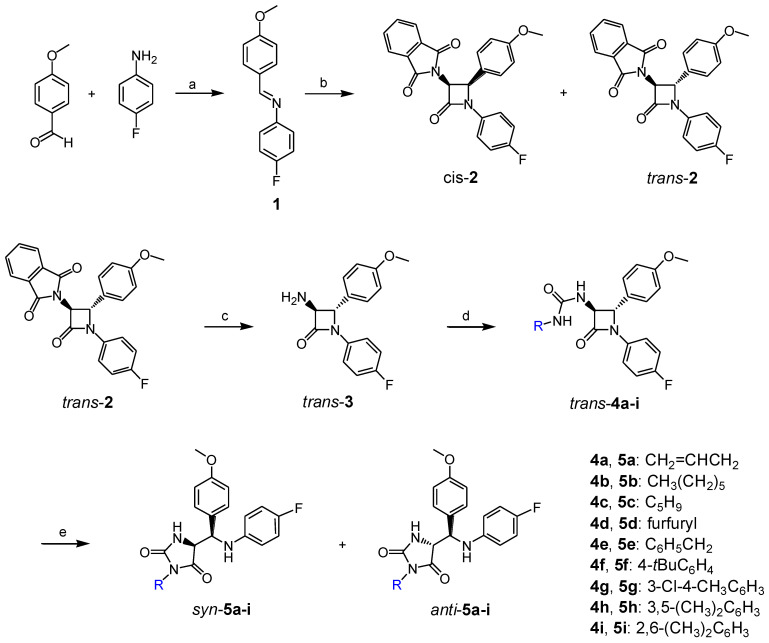
Reagents and conditions: (**a**) CH_2_Cl_2_, room temperature, 20 h; (**b**) phthalimidoacetic acid, 2-chloro-1-methylpyridinium iodide, triethylamine, CH_2_Cl_2_, 0 °C, 2 h, room temperature, 20 h; (**c**) ethylenediamine, EtOH, 65 °C, 1 h; (**d**) R-NCO, acetonitrile, 0 °C, 2 h, room temperature, 18 h; and (**e**) 25% NaOMe in MeOH, MeOH, 65 °C, 1 h.

The synthesis of the new 3,5-disubstituted hydantoins **5a**–**i** was carried out as shown in Scheme 1. This study demonstrates the in situ cyclization of racemic *trans*-β-lactam ureas **4a**–**i** under basic conditions by intramolecular amidolysis leading to the formation of a five-membered hydantoin ring. In the first step, imine **1** was prepared by a condensation reaction between 4-methoxybenzaldehyde and 4-fluoroaniline in an anhydrous dichloromethane medium, using powdered molecular sieves 4 Å (Scheme 1) [57]. The isolated imine **1** was obtained in a yield of 73% after recrystallization from a mixture of ethyl acetate and *n*-hexane. 

Following the formation of imine **1**, a reaction was carried out with *N*-phthalimidoglycine in the presence of triethylamine and 2-chloro-1-methylpyridinium iodide, a reagent known as Mukaiyama reagent. This step led to the preparation of a racemic mixture of *cis*- and *trans*-3-phthalimido-β-lactam **2**, as shown in Scheme 1 [58,59]. The choice of starting materials, 4-methoxybenzaldehyde and 4-fluoroaniline, lays in the already demonstrated bioactivity of these pharmacophores [59]. The [2 + 2]-cycloaddition reaction of imine **1** with in situ-generated ketene from acid and triethylamine, also known as the Staudinger reaction, is characterized by the sequential formation of the covalent N1–C2 and C3–C4 bonds within the β-lactam ring [60,61]. To gain a deeper insight into the nature of the reaction, the crude reaction mixture was analyzed by ^1^H NMR spectroscopy. The analysis revealed the presence of two isomeric β-lactams, with a remarkable *cis*/*trans* ratio of approximately 1:5. This finding was subsequently validated by HPLC analysis, which provided a definitive representation of the isomer distribution. In the ^1^H NMR spectra of the *cis* stereoisomer, the two protons of the β-lactam ring, H-3 and H-4, were observed as two doublets at 5.42 ppm and 5.65 ppm, respectively, with a coupling constant ^3^*J*_H3,H4_ of 5.5 Hz. In contrast, the *trans*-isomer exhibited a coupling constant of *J* = 2.6 Hz, with the corresponding protons appearing as doublets at 5.28 and 5.31 ppm. These spectral data enabled the accurate determination of the structures of the expected diastereomeric β-lactams, namely (±)-*cis*-**2** and (±)-*trans*-**2**, as shown in Scheme 1. Flash column chromatography was used to separate the *cis*- and *trans*-isomers, yielding pure *trans*-**2** (75%) and pure *cis*-**2** (11%). Given that the ketene generated in situ from the reaction between *N*-phthalimidoglycine and triethylamine corresponds to a Sheehan ketene, it exhibits a preferential formation of the *trans*-β-lactam [62].

The protecting group, the phthalimide group, was removed with ethylenediamine in anhydrous ethanol, yielding the (±)-3-amino-β-lactam **3** in a 67% yield (Scheme 1) [58]. 

In the next reaction step, compound **3** was reacted with various aliphatic and aromatic isocyanates in dry acetonitrile at room temperature, resulting in the formation of (±)-*trans*-β-lactam ureas **4a**–**i** (Scheme 1) [63]. The resulting products **4a**–**i** were obtained in yields ranging from 75 to 98%. ^1^H and ^13^C NMR, HRMS, and FTIR were used to characterize these compounds **4a**–**i**. In ^1^H NMR, the appearance of proton signals at about *δ* = 4.40 ppm and about 5.00 ppm, respectively, as doublets, correspond to the protons of the C-3 and C-4 of the β-lactam ring, respectively. The signal of the NH proton bound to the ring system is observed as a doublet in the chemical shift range of 5.78 to 6.55 ppm. The signal of the second NH proton is observed as a singlet, doublet, or triplet, depending on which alkyl or aryl substituent is bound to this NH proton. The ^13^C NMR spectra also showed the peaks for the β-lactam and urea carbonyl carbons at about 166 and 156 ppm, respectively, confirming the synthesis of the β-lactam ureas. The appearance of peaks at 3311–3396 cm^−1^ (N-H stretching), 1732–1756 cm^−1^ (C=O stretching), and 1426 cm^−1^ (C–N stretching), in the FTIR spectra reconfirmed the β-lactam and urea moieties.

Subsequently, the treatment of *trans*-β-lactam urea **4a**–**i** with sodium methoxide in dry methanol at 65 °C for 1 h [45] resulted in the formation of hydantoins **5a**–**i** in good to excellent yields but with poor-to-modest diastereoselectivities (Scheme 1 and Table 1). The diastereomeric ratio was determined by RP-HPLC analysis and ^1^H NMR spectroscopy of reaction mixtures. For example, the diastereomeric ratio for the allyl derivative **5a** (Entry 1) is 52.5:47.5, indicating a slight preference for the *syn*-isomer. However, the diastereomeric ratio for the 2,6-dimethylphenyl derivative **5i** (Entry 9) is 58.8:41.2, indicating a much stronger preference for the *syn*-isomer. The diastereomeric ratio for 3-chloro-4-methylphenyl **5g** indicates a slight preference for the *anti*-isomer, but it is not a highly diastereoselective reaction (48.3:51.7).

**Scheme 2:**
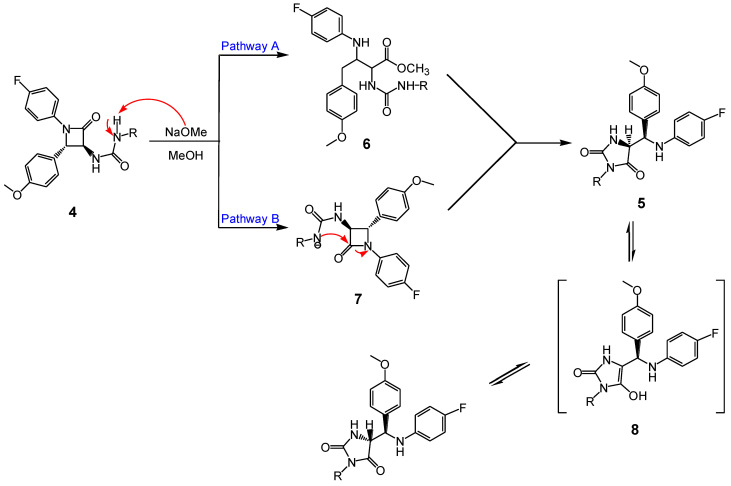
Plausible mechanism for the formation of hydantoin **5a**–**i** from β-lactam urea **4a**–**i**.

According to the reaction mechanism proposed by Mehra and Kumar (Scheme 2), the formation of hydantoin **5** from β-lactam urea **4** can involve methoxide-assisted tandem intermolecular amidolysis–intramolecular cyclization (pathway A, Scheme 2) or a base-assisted formation of a ureido anion followed by intramolecular amidolysis (pathway B, Scheme 2) [45,64]. If the reaction was carried out under mild reaction conditions, the second reaction pathway is more likely. In the presence of a base, such as sodium methoxide, optically active hydantoin tautomerizes easily, indicating the existence of an enol tautomer **8** [65]. 

The racemic *trans*-β-lactam ureas **4a**–**i** were used to prepare 3,5-disubstituted hydantoins, and as a result, a racemic mixture of *syn*- and *anti*-hydantoins **5a**–**i** was obtained. The diastereomerically pure hydantoins **5a**–**i** were obtained using preparative HPLC with a linear gradient of water (mobile phase A) and acetonitrile (mobile phase B). 

The isolated *syn*- and *anti*-diastereomers **5a**–**i** were characterized using various analytical techniques, including melting point determination, thin-layer chromatography (TLC), infrared (IR) spectroscopy, nuclear magnetic resonance (NMR) spectroscopy, high-performance liquid chromatography (HPLC), and high-resolution mass spectrometry (HRMS), as detailed in the Materials and Methods section.

The IR spectroscopy study confirms the structure of the compounds mentioned above. The IR spectra of the hydantoin derivatives **5a**–**i** show absorption peaks in the range of 3200 to 3300 cm^−1^, corresponding to the N-H stretching vibrations of the imide and amide functionalities. Absorption bands observed between 1770 and 1705 cm^−1^ indicate the asymmetric and symmetric stretching of the carbonyl groups in the hydantoin ring. In addition, the IR spectra of compounds **5a**–**i** show absorption bands around 1610 cm^−1^ (amide I) and around 1510 cm^−1^ (amide II), which are characteristic of amide groups.

### 2.2. NMR Analysis of Syn- and Anti-***5a***

**Figure 2:**
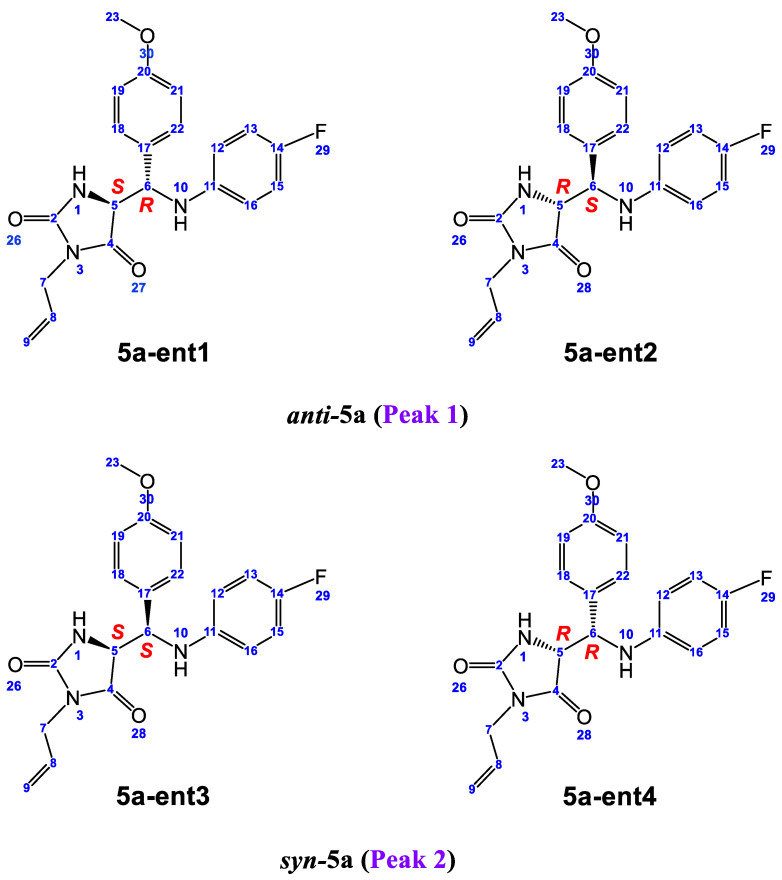
Structures, stereochemistry, and numbering for Peak 1 and Peak 2.

A comparison of chemical shifts, NOE interactions, and coupling constants was performed to obtain more information about the conformation of the two diastereoisomers. Structures, stereochemistry, and numbering for the first eluted (Peak 1) and the second eluted (Peak 2) diastereomer are shown in Figure 2. A comparison of the chemical proton shifts (Table S1) revealed the largest difference in the allylic protons CH_2_-7, H-8 and CH_2_-9, which does not reflect differences in the stereochemistry of the two isomers, but is most likely a consequence of the different positioning of the allylic moiety, possibly due to a different conformation of the hydantoin ring in the two diastereoisomers. A comparison of the carbon chemical shifts (Table S2) revealed the largest difference in the chemical shifts of C-6 and C-17, which is a direct consequence of the different stereochemistry at position C-6. A comparison of the NOE interactions (Table S3) revealed only one significant difference. Two weak interactions exist in the first eluted diastereoisomer (Peak 1), H-5/H12,16 and H-5/H18,22, indicating that in this molecule both aromatic rings are positioned equally close to the hydantoin ring proton H-5. In the second eluted diastereoisomer (Peak 2), however, there is only one medium interaction H-5/H18,22 which means that in this molecule the conformation is different and the methoxybenzene is close to H-5, but the fluorobenzene is not. A comparison of the coupling constants (Table S4) showed the largest difference in ^3^*J*_H-6,NH-10_, which was 6.4 Hz for Peak 1 (*anti*-**5a**) and 10.6 Hz for Peak 2 (*syn*-**5a**). This suggests that the angle between H-9 and NH-10 has changed with different stereochemistry. Although it was not possible to unambiguously determine the stereochemistry of **5a** using NMR data alone, the combination with molecular modelling proved to be successful.

### 2.3. ECD and VCD Analysis and Absolute Configuration Determination of Syn-***5a*** and Anti-***5a*** Allyl Hydantoin 

**Figure 3:**
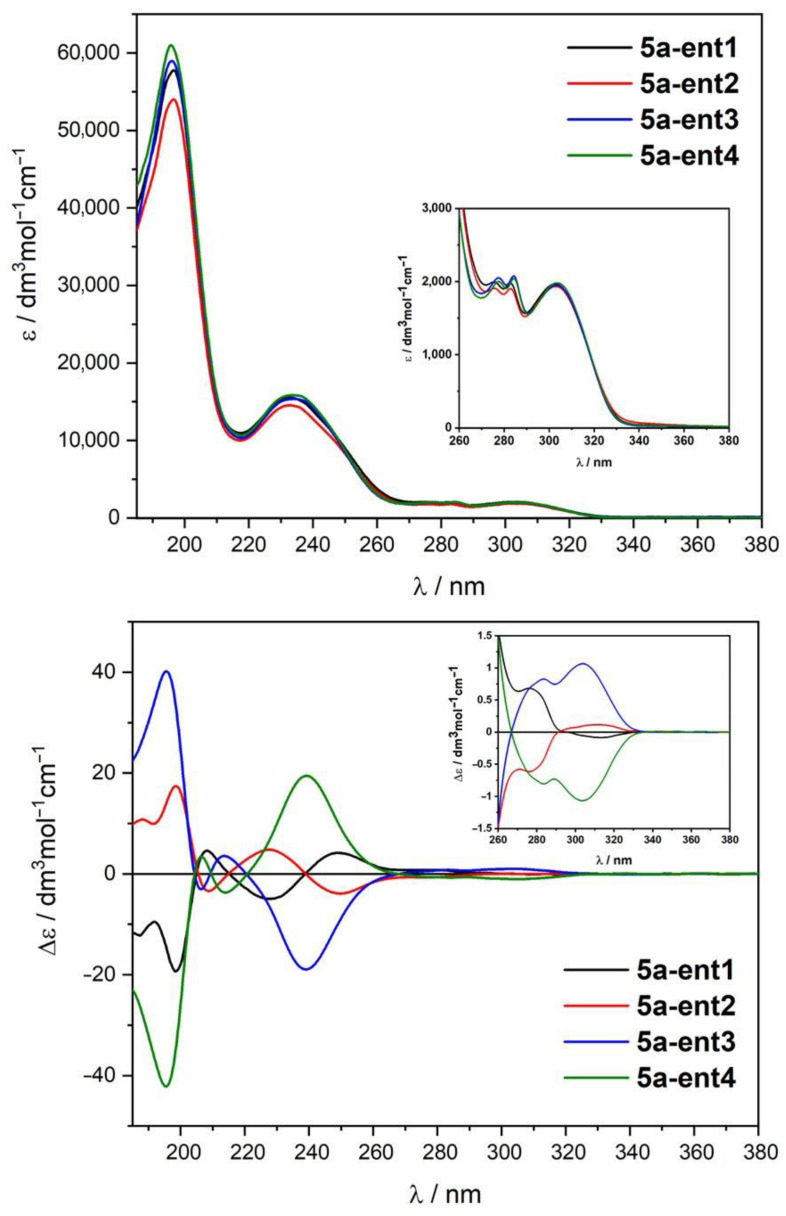
Experimental UV and ECD spectra of hydantoins **5a**-**ent1** (black line), **5a**-**ent2** (red line), **5a**-**ent3** (blue line), and **5a**-**ent4** (green line) recorded in CH_3_CN.

The racemic *syn*- and *anti*-allyl hydantoins, *syn*-**5a** and *anti*-**5a**, were selected for this study and purified to enantiomeric purity (e.e. > 99%) by preparative HPLC on a chiral semi-preparative CHIRAL ART Amylose-SA column using *n*-hexane/2-PrOH (90/10, *v*/*v*) as the mobile phase. Electronic circular dichroism (ECD) and vibrational circular dichroism (VCD) spectra of the enantiomers **5a**-**ent1**, **5a**-**ent2** (*anti*-**5a**, Peak 1), **5a**-**ent3,** and **5a**-**ent4** (*syn*-**5a**, Peak 2) were recorded in acetonitrile and calculated by density functional theory (DFT). Figure 3 reports the UV and ECD spectra of the four isomers of **5a**. As expected, the UV spectra are all very similar, while the ECD spectra appear paired in mirror-image couples for the two enantiomers of each diastereomer of **5a**. It was anticipated that ECD is dominated by the reciprocal arrangement between the two aromatic chromophores, and therefore, could be especially sensitive to the configuration at C-6; however, major differences also emerge between the diastereomeric pairs, related to the configuration at C-5.

**Figure 4:**
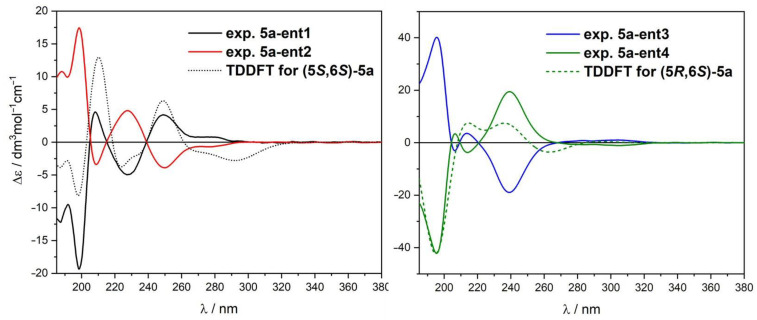
Comparison between experimental ECD spectra of hydantoins **5a**-**ent1**/**5a**-**ent2** (black/red lines) and **5a**-**ent3**/**5a**-**ent4** (blue/green lines) recorded in CH_3_CN with the spectra calculated for (5S,6S)-**5a** (black dotted line) and (5*R*,6*S*)-**5a** (green dashed line) at TD B3LYP/def2-TZVP/PCM//B3LYP-D3BJ/6-311+G(d,p) level as Boltzmann averages over eight and five conformers, respectively. Plotting parameters for (5*S*,6*S*)-**5a**: UV shift +15 nm, σ = 0.24 eV. Plotting parameters for (5*R*,6*S*)-**5a**: UV-shift +4 nm, σ = 0.38 eV.

The computational procedure employed for ECD calculations followed a well-established workflow [66,67]. The input structures had (5*R*,6*S*) and (5*S*,6*S*) configurations for the *syn*- and *anti*-isomers of **5a**, respectively. The conformational space was sampled by means of a systematic search with molecular mechanics force field (MMFF). All conformers thus obtained were first optimized by DFT at B3LYP-D3/6-31G(d) level in vacuo, then re-optimized at B3LYP-D3BJ/6-311+G(d,p) level in vacuo, and final energies were estimated at the same level including the PCM solvent model for acetonitrile. In this way, five different conformers were obtained for (5*R*,6*S*)-**5a** and eight for (5*S*,6*S*)-**5a** with Boltzmann populations > 3% at 300 K (see Supporting Information, Tables S5 and S6). Although comparing experimental NOE data with optimized geometries is not trivial for compounds with multiple fast-exchanging conformers, we notice that the (5*S*,6*S*)-**5a** isomer yields low-energy structures with H-2 directed toward the fluorobenzene ring, while this is not true for (5*R*,6*S*)-**5a**. According to NOE results (see above), this suggests an *anti* stereochemistry for Peak 1 and *syn* stereochemistry for Peak 2, corresponding, respectively, to configuration (5S*,6S*) and (5R*,6S*). ECD calculations were then run with time-dependent DFT (TD-DFT) at CAM-B3LYP/def2-TZVP/PCM and B3LYP/def2-TZVP/PCM levels. Final spectra were generated as Boltzmann averages at 300 K using internal energies and plotted as a sum of Gaussians with best-fit bandwidth. The comparison between the calculated and experimental ECD spectra (Figure 4) suggests that the isomer **5a**-**ent1** has a (5*S*,6*S*) configuration and the isomer **5a**-**ent4** has a (5*R*,6*S*) configuration. The assignment was substantiated by evaluating similarity factors between experimental and calculated spectra (Supporting Information, Table S7) [68]. Furthermore, to confirm the assignment for **5a**-**ent1**/**5a**-**ent2**, the uncertainty related to the conformational averaging was reduced by considering a truncated model where the *N*-allyl group was replaced by *N*-methyl. This truncation approach [69] led to the same assignment as above (Supporting Information, Tables S8 and S9 and Figure S147).

**Figure 5:**
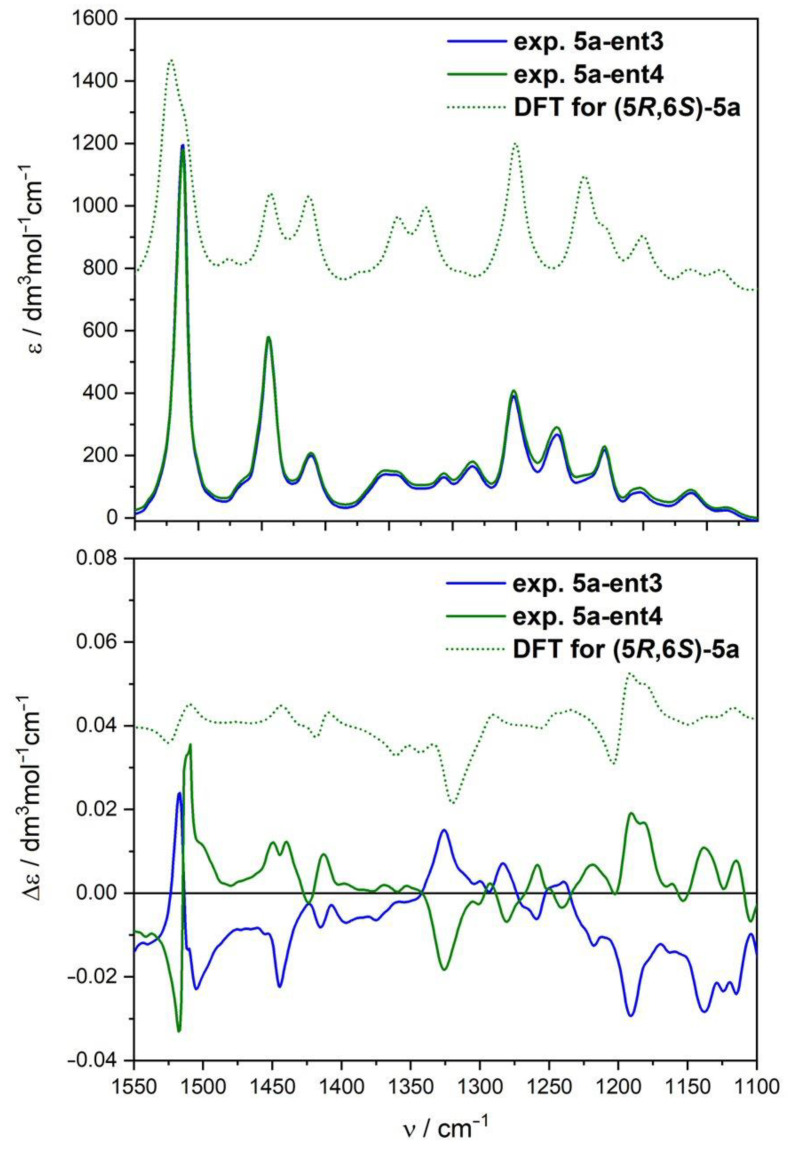
Comparison between experimental IR (top) and VCD spectra (bottom) of hydantoins **5a**-**ent3** (blue line) and **5a**-**ent4** (green line) recorded in CD_3_CN with the spectrum calculated for (5R,6S)-**5a** at B3LYP-D3BJ/6-311+G(d,p)/PCM level as Boltzmann averages over 8 conformers. Plotting parameters: scaling factor 0.985, σ = 8 cm^−1^.

Finally, following the advice to use multiple chiroptical techniques to avoid potentially incorrect assignments [70,71], we extended our study to vibrational CD (VCD). The VCD spectra measurements for **5a**-**ent3** and **5a**-**ent4** were especially good in terms of signal-to-noise ratio thanks to the availability of a larger amount of enantiopure samples and looked like mirror images throughout the fingerprint region (Figure 5). DFT calculations run at B3LYP-D3BJ/6-311+G(d,p)/PCM level on (5*R*,6*S*)-**5a** reproduced satisfactorily the experimental VCD spectrum for **5a**-**ent4**, thus confirming the assignment obtained by ECD (Figure 5; similarity factors are provided in the Supporting Information, Table S7). In conclusion, the chiroptical analysis afforded the following assignments: (5*S*,6*S*)-**5a**-**ent1**, (5*R*,6*R*)-**5a**-**ent2**, (5*S*,6*R*)-**5a**-**ent3**, and (5*R*,6*S*)-**5a ent4**.

### 2.4. Antiproliferative Activity of Syn- and Anti-***5a***–***i***

In the present work, the eighteen synthesized hydantoin derivatives, *syn*-**5a**–**i** and *anti*-**5a**–**i**, were evaluated in vitro for their antiproliferative activity by MTT assay. Three human cancer cell lines, including liver hepatocellular carcinoma cells (HepG2), ovarian carcinoma cells (A2780), and breast adenocarcinoma cells (MCF7), and a human non-tumor foreskin fibroblasts cell line HFF-1, were selected to determine in vitro antiproliferative activity. The results are summarized in Table 2. The following compounds, *anti*-**5b**, *syn*-**5f**, *anti*-**5f,** and *anti*-**5g,** showed moderate activity against liver cancer cell lines HepG2 with IC_50_ values in the range 15–35 μmol/L, while the other *syn*- and *anti*-hydantoin compounds showed insignificant inhibitory potential on HepG2 cell lines. Most of the tested compounds showed moderate antiproliferative activity, while the two hydantoins *anti*-**5a** and *syn*-**5d** showed no cytotoxicity against A2780 cells. Of the eighteen tested hydantoins, eleven exhibited moderate antiproliferative potential against MCF7 cells with IC_50_ values in the range 20–55 μmol/L. Compounds *syn*-**5a**, *anti*-**5a**, *syn*-**5d**, *anti*-**5d,** and *syn*-**5e** showed no cytotoxicity against MCF7 cells. Interestingly, the compound *anti*-**5c** with a cyclopentyl group at the N-3 position of the hydantoin ring showed significant antiproliferative effects on human breast carcinoma cell line MCF7 with an IC_50_ value of 4.5 μmol/L. However, its diastereomer *syn*-**5c** only showed moderate antiproliferative effect against the same cell line (IC_50_ = 41 μmol/L). The hydantoin compounds *anti*-**5b**, *anti*-**5c**, *syn*-**5f**, *syn*-**5g**, *anti*-**5g**, *syn*-**5h**, and *anti*-**5h** showed moderate cytotoxic effects, whereas the other hydantoins had less or no toxic effect on the HFF-1 healthy cells.

### 2.5. In Silico Physicochemical and Biological Profiling of Syn/Anti-***5a**–**i***

The ADMET profile for the synthesized derivatives **5a**–**i** was estimated using the program ADMET Predictor [53] and the web server admetSAR [54]. All synthesized 3,5-disubstituted hydantoins **5a**–**i**, with the exception of **5f**, are drug-like molecules compliant with Lipinski’s and Veber’s rules (Table S12). Orally administered molecules are likely to have logP ≤ 5, MW ≤ 500, HBD ≤ 5, and HBA ≤ 10 according to Lipinski’s rule of five [72,73], and/or TPSA ≤ 140 Å^2^ (or 12 or fewer HBD and HBA), and a number of rotatable bonds equal to or less than 10 according to Veber’s rule [74]. The derivative **5f** with a 4-*tert*-butylphenyl substituent violates Lipinski’s rule in terms of lipophilicity (logP = 5.01). While all 3,5-disubstituted hydantoins **5a**–**i** are nonionizable compounds and most of them have logP ≥ 3 (Table S10), they are predicted to be moderately water soluble (Table S11). All molecules are also predicted to have moderate membrane permeability according to the ADMET predictor’s Peff and MDCK values, which indicates their good oral bioavailability. The membrane permeability of a compound may be reduced by its interaction with efflux pumps such as P-glycoprotein (Pgp). The compounds **5b**–**i** are estimated to be Pgp substrates and many of them are not predicted to be Pgp inhibitors (Table S11). Furthermore, hydantoins **5a**–**i** are estimated to not penetrate the BBB (BBB_filter) and have low retention in brain (logBB, Table S11), and hence it is very likely that they do not enter the brain [75]. Regarding distribution in the body, they are predicted to be transported by plasma proteins (Table S11, low hum_fup% values). Considering metabolism, the web server admetSAR is commonly used to estimate the probability that the compounds are substrates and inhibitors of cytochrome P450 (CYP450) oxidoreductases. According to admetSAR, all hydantoins **5a**–**i** should be substrates only for CYP3A4 and most of them are also predicted to be CYP3A4 inhibitors (Table S11). In terms of toxicity, hydantoins **5f**–**i** are classified as cardiotoxic (hERG_Filter). All synthesized hydantoins **5a**–**i** are predicted to be non-mutagenic and non-carcinogenic to rats or mice (Table S11).

The software PASS 2020 [55] and the online server SwissTargetPrediction [56] were used to predict biological activities of the new 3,5-disubstituted hydantoins **5a**–**i**. The PASS 2020 simultaneously predicts 1945 recommended biological activities, including pharmacological effects, mechanisms of action, interaction with drug-metabolizing enzymes and transporters, toxic and adverse effects, influence on gene expression, etc., using built-in machine-learning models. The results consist of Pa and Pi values, which are computed probabilities that a compound is active and inactive, respectively, for each type of activity from the biological activity spectrum. The Pa and Pi values range from 0.0 to 1.0. Considering only the activities predicted with Pa > Pi and Pa > 0.5, all synthesized hydantoins **5a**–**i** should have antiarthritic activity (Pa ≥ 0.727). In addition, only the compound syn/anti-**5d** is predicted to inhibit thioredoxin glutathione reductase (Pa = 0.665), beta lactamase (Pa = 0.554), DNA-directed DNA polymerase (Pa = 0.553), and DNA polymerase beta (Pa = 0.542). In contrast to PASS, SwissTargetPrediction estimated protein targets based on the structural similarity of hydantoins **5a**–**i** with 327,719 already known actives on 2092 human proteins. No high-confidence predictions were found indicating substantial novelty in the structures of the synthesized 3,5-disubstituted hydantoins.

## 3. Materials and Methods

### 3.1. Chemistry 

#### 3.1.1. General 

All required chemicals were purchased from commercial suppliers, including Sigma-Aldrich (Munich, Germany), Merck (Darmstadt, Germany), Fluka (Buchs, Switzerland), and Kemika (Zagreb, Croatia). Prior to use, the solvents dichloromethane, ethanol, and acetonitrile were subjected to the required drying procedures according to established protocols. The HPLC-grade solvents, acetonitrile, 2-propanol, and *n*-hexane, were purchased from Honeywell (Seelze, Germany). The reactions were monitored by thin-layer chromatography (TLC), which was performed on 0.25 mm silica gel plates (50F-254, DC-Alufolien-Kieselgel F254, Sigma Aldrich, Merck KGaA, Darmstadt, Germany). The TLC plates were observed under ultraviolet light (254 nm) or stained with ninhydrin. Flash column chromatography was performed with silica gel Si50 (particle size 0.04–0.053 mm, 230–400 mesh, Sigma Aldrich, Munich, Germany).

The determination of melting points was conducted using an Olympus BX51 polarizing microscope (Olympus Corporation, Tokyo, Japan), which was equipped with a Linkam TH500 hot stage and a PR500 temperature controller (Technical Manufacturing Corporation, Peabody, Massachusetts, USA). Images were captured using the Olympus C5050 ZOOM digital camera.

FTIR-ATR spectra were obtained using a PerkinElmer UATR Two spectrometer (PerkinElmer Inc., Waltham, MA, USA), operating within a range of 450 cm^−1^ to 4000 cm^−1^.

All solvents used for NMR sample preparation were purchased from EurIsotop (Saint-Aubin, France). ^1^H and ^13^C NMR spectra were recorded on a Brucker AV 300 or AV 500 (1H 300 or 500 MHz and ^13^C 75 or 151 MHz) spectrometers (Bruker Technologies, Ettlingen and Leipzig, Germany) in chloroform (CDCl_3_) or acetonitrile (CD_3_CN) at ambient temperature. All solvents utilized for the preparation of NMR samples were obtained from EurIsotop (Saint-Aubin, France). The ^1^H and ^13^C NMR spectra were recorded on Brucker AV 300 or AV 500 spectrometers (^1^H 300 or 500 MHz and ^13^C 75 or 151 MHz) (Bruker Technologies, Ettlingen and Leipzig, Germany), utilizing chloroform (CDCl_3_) or acetonitrile (CD_3_CN) at ambient temperature. The chemical shifts (*δ*) were expressed in parts per milion (ppm) relative to tetramethylsilane (TMS), which was used as an internal standard. All coupling constants (*J*) are given in hertz (Hz). The splitting patterns were reported as follows: s for singlet; d for doublet; t for triplet; p for pentet; h for heptet; m for multiplet; bs for broad singlet; dd for a doublet of doublets; and tdd for a triplet of a doublet of doublets.

An Agilent 1290 Infinity II/5550 Q-TOF instrument (Agilent Technologies, Waldbronn, Germany) with electrospray ionization (ESI) quadrupole-time-of-flight (Q-TOF) mass spectrometry with high-resolution was used as the analytical technique to determine the molecular weight of the synthesized compounds.

RP-HPLC studies were performed with the aim of determining the *cis*/*trans* ratio of 3-phthalimido-β-lactam **2** and determining the purity of compound 3-amino-β-lactam **3**. The Symmetry C18 column (150 × 4.6 mm, 5 μm, Waters, Milford, MA, USA) was used for these analyses. The analyses were carried out on an Agilent 1200 Series System (Agilent Technologies, Waldbronn, Germany), which was equipped with a vacuum degasser, a quaternary pump, a thermostatically controlled column compartment, an autosampler, and a variable wavelength detector. The methodology used was as follows: water served as eluent A, while acetonitrile constituted eluent B. The gradient for the first 0–15 min went from 30% to 70% eluent B. From 15–18 min, an isocratic phase with 70% eluent B was applied. From 18.01 to 21 min, an additional isocratic phase with 30% B was introduced. The flow rate was maintained at 1 mL/min, the detection wavelength was set to 254 nm, the column temperature was set to 30 °C, and the injection volume was set to 20 μL.

*Trans*-(±)-β-lactam ureas **4a**–**i** were analyzed by RP-HPLC using a Synergi Polar-RP 80A column (150 mm × 4.6 mm, 4 μm, Phenomenex, Torrance, CA, USA) with a gradient elution setup consisting of (A) water + 0.1% trifluoroacetic acid and (B) acetonitrile. The elution program was as follows: 0–10 min, 50–100% B; 10–13 min, 100% B; and 13.01–17 min, 50% B. The flow rate was maintained at 1.0 mL/min. The column temperature was maintained at 30 °C, and the sample injection volume was 20 μL. UV detection was performed at a wavelength of 254 nm. 

The *syn*/*anti* ratio of hydantoins was determined by HPLC on an Agilent 1200 series system using a Zorbax Extend-C18 column (250 × 4.6 mm, 5 μm, Agilent Technologies, Milford, MA, USA). The flow rate was set to 0.8 mL/min, the column temperature was maintained at 30 °C. A sample volume of 20 μL was injected and UV detection was performed at a wavelength of 254 nm. A gradient elution of two solvents was used—Solvent A (water) and Solvent B (acetonitrile). The mobile phase gradient elutions used in the three methods were as follows: For Method A (for **5a**, **5d**, **5e**), the gradient program was as follows: 0–60 min, 35–48% B; 60–63 min, 48% B; and 63.01–67 min, 35% B. For Method B (for **5c**, **5h**, **5i**), the gradient program was as follows: 0–60 min, 35–60% B; 60–63 min, 60% B; and 63.01–67 min, 35% B. For Method C (for **5b**, **5f**, **5g**), the gradient program was as follows: 0–60 min, 45 58% B; 60–63 min, 58% B; and 63.01–67 min, 45% B. The preparative HPLC was carried out on an Agilent 1260 Infinity II HPLC system, which was equipped with an Agilent MWD detector. A Zorbax Extend C-18 PrepHT preparative column (250 mm × 21.2 mm i. d., 5-µm particle size) was used, and the gradient elution program with water (phase A) and acetonitrile (phase B) was delivered at 17 mL/min. The chromatograms were monitored at 254 nm. In addition, the injection volume was 500 μL. The mobile phase gradient elutions used in the three methods were as follows: For Method D (for **5a**, **5d**, **5e**), the gradient program was as follows: 0–35.98 min, 35–48% B; 35.98–37.78 min, 48% B; and 37.79–40.18 min, 35% B. For Method E (for **5c**, **5h**, **5i**), the gradient program was as follows: 0–35.98 min, 35–60% B; 35.98–37.78 min, 60% B; and 37.78–40.18 min, 35% B. For Method F (for **5b**, **5f**, **5g**), the gradient program was as follows: 0–35.98 min, 45–58% B; 35.98–37.78 min, 58% B; and 37.78–40.18, min 45% B.

The chiral separation of *syn*- and *anti*-allyl hydantoin enantiomers **5a** was carried out on the CHIRAL ART Amylose-SA semi-preparative column (250 mm × 8.0 mm i. d., 10-µm particle size). An isocratic elution was performed with *n*-hexane/PrOH (90/10, *v*/*v*) at a flow rate of 5 mL/min. The chromatograms were monitored at 254 nm and the injection volume was 500 μL. The 10 μm bulk immobilized amylose-based chiral stationary phase CHIRAL ART Amylose-SA S-10 μm used in the HPLC column was purchased from YMC (Kyoto, Japan) and packed in house using empty stainless steel HPLC columns (dimensions: 250 mm × 4.6 mm i. d. and 250 mm × 8.0 mm i. d.) from Knauer GmbH (Berlin, Germany).

The enantioseparation of the hydantoins *syn*-**5a**–**i** and *anti*-**5a**–**i** was carried out on an Agilent 1200 Series HPLC System (Agilent Technologies, Waldbronn, Germany). The system included a vacuum degasser, a quaternary pump, a thermostatically controlled column compartment, an autosampler, and a variable wavelength detector. The mobile phase was a mixture of *n*-hexane and 2-PrOH (90/10, *v*/*v*). All analyses were performed with a flow rate of 1.0 mL/min and at a column temperature of 30 °C. An injection volume of 20 μL was employed.

ECD spectra were recorded using a J-815 spectrometer (Jasco, Tokyo, Japan) at room temperature in spectroscopic grade acetonitrile. Solutions with concentrations in the range of 0.25 to 0.27 mM were measured in quartz cells with a path length of 1 and 0.1 cm. All spectra were carried out in the range from 380 to 180 nm using a scanning speed of 100 nm/min, a step size of 0.2 nm, a bandwidth of 1 nm, a response time of 0.5 s, and an accumulation of 5 scans. The spectra were background-corrected using acetonitrile as the solvent, and they were recorded under the same measurement conditions. 

VCD spectra were obtained using an FVS-6000 VCD spectrometer (Jasco, Tokyo, Japan) with a resolution of 4 cm^−1^ within the 2000–850 cm^−1^ range in spectroscopic grade CD_3_CN for 2 h. Solutions with concentrations in the range of 0.2–0.15 M in a BaF_2_ cell with a path length of 200 μm were placed on a rotating holder. Baseline correction was made by subtracting the spectrum of CD_3_CN recorded under identical conditions. 

#### 3.1.2. Synthesis of (4-Fluorophenyl)[(4-methoxyphenyl)methylene]amine (**1**)

To a solution containing 4-methoxybenzaldehyde (6 mL, 49.313 mmol) in anhydrous dichloromethane (30 mL) maintained at room temperature, 4-fluoroaniline (4.67 mL, 49.313 mmol) was then added. This was followed by the addition of activated 4 Å molecular sieve powder (2 g). The resulting reaction mixture was stirred at room temperature for 20 h. The mixture was then filtered through a short layer of Celite, which had previously been rinsed with dichloromethane, to remove the molecular sieve powder. The filtrate was concentrated under reduced pressure, yielding a crude imine in the form of an oil. After recrystallization from a solvent mixture of ethyl acetate and *n*-hexane, pure imine **1** (8.1 g, 73%) was isolated as white crystals. m.p. 68–70 °C. FTIR (ATR, cm^−1^): 2925, 2847, 1626, 1604, 1512, 1458, 1308, 1253, 1208, 1179, 1159, 1021, 841. ^1^H NMR (300 MHz, CDCl_3_), *δ*/ppm: 8.35 (s, 1H), 7.83 (d, *J* = 8.7 Hz, 2H), 7.21−7.12 (m, 2H), 7.06 (t, *J* = 8.7 Hz, 2H), 6.97 (d, *J* = 8.8 Hz, 2H), 3.86 (s, 3H). ^13^C NMR (150 MHz, CDCl_3_), *δ*/ppm: 162.7, 161.0 (d, *J* = 244.1 Hz), 159.6, 148.5 (d, *J* = 2.7 Hz), 130.6, 129.3, 122.3 (d, *J* = 8.1 Hz), 115.9 (d, *J* = 22.4 Hz), 114.3, 55.6. C_14_H_12_FNO (229.25): calcd. C 73.35, H 5.28, N 6.11; found C 73.06, H 5.40, N 6.12.

#### 3.1.3. *Cis*/*trans*-(±)-1-(4-fluorophenyl)-2-(4-methoxyphenyl)-4-oxoazetidin-3-yl-isoindoline-1,3 dione (**2**)

A suspension containing phthalimidoacetic acid (4.03 g, 19.529 mmol) in anhydrous dichloromethane (150 mL) was cooled to 0 °C under an inert argon atmosphere. 2-Chloro-1-methylpyridinium iodide (6.69 g, 39.258 mmol) and triethylamine (10.9 mL, 78.516 mmol) were added to this solution. The resulting reaction mixture was stirred for 2 h to 0 °C. After that, a solution of imine **1** (3.0 g, 13.086 mmol) in anhydrous dichloromethane (25 mL) was added dropwise and stirring was then continued for a further 2 h, during which the temperature rose from 0 °C to room temperature. The mixture was then refluxed for 20 h and progress was monitored by TLC and HPLC. After the completion of the reaction, the reaction mixture was washed with a solution of saturated sodium bicarbonate (150 mL), a solution of saturated sodium chloride (150 mL), and deionized water (150 mL). The organic layer was then dried over anhydrous sodium sulfate, filtered, and concentrated under reduced pressure, yielding the crude product. The *cis*/*trans* ratio of the crude product was determined to be 1:5. The crude product was purified by column chromatography over silica gel in a chloroform/dichloromethane/ethyl acetate mixture (7/1/1, *v*/*v*/*v*). The pure forms of the *cis*- and *trans*-3-phthalimido-β-lactams were obtained as a white solid. *R*_f_ (*cis*-**2**) = 0.59, *R*_f_ (*trans*-**2**) = 0.71 (chloroform/dichloromethane/ethyl acetate = 7/1/1, *v*/*v*/*v*).

##### Cis-**2**

White solid (600 mg, 11%), m.p. 194.1−198.8 °C. FTIR (ATR, cm^−1^): 2895, 1781, 1756, 1721, 1611, 1510, 1467, 1442, 1385, 1254, 1175, 1115, 1053, 956, 832, 814, 750, 716. ^1^H NMR (600 MHz, CDCl_3_), *δ*/ppm: 7.77−7.61 (m, 4H), 7.48−7.38 (m, 2H), 7.17 (d, *J* = 8.7 Hz, 2H), 7.02 (t, *J* = 8.7 Hz, 2H), 6.72 (d, *J* = 8.8 Hz, 2H), 5.65 (d, *J* = 5.5 Hz, 1H), 5.42 (d, *J* = 5.5 Hz, 1H), 3.66 (s, 3H). ^13^C NMR (151 MHz, CDCl_3_), *δ*/ppm: 166.9, 160.9, 159.8, 159.5 (d, *J* = 244.2 Hz), 134.4, 133.8 (d, *J* = 2.7 Hz), 131.3, 128.7, 123. 7, 119.0 (d, *J* = 7.9 Hz), 116.1 (d, *J* = 22.8 Hz), 114.2, 61.0, 59.3, 55.3. HR-MS (ESI-QTOF) *m*/*z*: calcd. for C_24_H_17_FN_2_O_4_, [M + H]^+^, 417.1251; found 417.1255. 

##### Trans-**2**

White solid (4.1 g, 75%), m.p. 197.3−198.8 °C. FTIR (ATR, cm^−1^): 3007, 2932, 2836, 1778, 1754, 1713, 1612, 1507, 1467, 1369, 1311, 1253, 1229, 1180, 1148, 1120, 1105, 1034, 970, 941, 833, 717, 530. ^1^H NMR (600 MHz, CDCl_3_), *δ*/ppm: 7.91−7.84 (m, 2H), 7.80−7.73 (m, 2H), 7.36−7.27 (m, 4H), 7.01−6.88 (m, 4H), 5.31 (d, *J* = 2.6 Hz, 1H), 5.28 (d, *J* = 2.6 Hz, 1H), 3.81 (s, 3H). ^13^C NMR (151 MHz, CDCl_3_), *δ*/ppm: 166.9, 162.1, 160.5, 159.5 (d, *J* = 244.1 Hz), 134.7, 133.6 (d, *J* = 2.8 Hz), 131.8, 127.7, 127.4, 124.0, 119.3 (d, *J* = 8.0 Hz), 116.1 (d, *J* = 22.8 Hz), 115.0, 63.1, 61.3, 55.5. HR-MS (ESI-QTOF) *m*/*z*: calcd. for C_24_H_17_FN_2_O_4_, [M + H]^+^, 417.1251; found 417.1248.

#### 3.1.4. Trans-3-amino-1-(4-fluorophenyl)-4-(4-methoxyphenyl)-2-azetidinone (**3**)

To a suspension of *trans*-3-phthalimido-β lactam **2b** (3.78 g, 9.078 mmol) in dry ethanol (100 mL) was added ethylenediamine (1.21 mL, 18.156 mmol), and the reaction mixture was stirred at 65 °C under an argon atmosphere. The progress of the reaction was monitored by TLC. After one hour, the ethanol was evaporated in vacuo and the resulting crude product was dissolved in ethyl acetate (200 mL). The solution was then washed with a solution of saturated sodium chloride (100 mL) and deionized water (100 mL). The organic layer was dried over anhydrous sodium sulfate, filtered and concentrated under reduced pressure to give the crude product which was then purified by SiO_2_ flash column chromatography with ethyl acetate as an eluent to give compound **3** (1.73 g, 67%) as a white solid. *R*_f_ = 0.39 (ethyl acetate), m.p. 98.7−101.2 °C. FTIR (ATR, cm^−1^): 3384, 3341, 3177, 2933, 2840, 1732, 1611, 1510, 1483, 1380, 1246, 1229, 1154, 1115, 1029, 965, 832, 793, 717. ^1^H NMR (600 MHz, CDCl_3_), *δ*/ppm: 7.30−7.20 (m, 4H), 6.98−6.84 (m, 4H), 4.60 (d, *J* = 2.2 Hz, 1H), 4.04 (d, *J* = 2.2 Hz, 1H), 3.80 (s, 3H), 1.82 (bs, 2H). ^13^C NMR (151 MHz, CDCl_3_), *δ*/ppm: 168.0, 160.0, 159.2 (d, *J* = 243.6 Hz), 133.8 (d, *J* = 2.8 Hz), 128.6, 127.3, 119.0 (d, *J* = 7.9 Hz), 116.0 (d, *J* = 22.6 Hz), 114.7, 70.2, 66.6, 55.5. HR-MS (ESI-QTOF) *m*/*z*: calcd. for C_16_H_15_FN_2_O_2_, [M + H]^+^, 287.1191; found 287.1183.

#### 3.1.5. General Procedure for the Preparation of (±)-*trans*-β-lactam Ureas **4a**–**i**

To a solution of *trans*-3-amino-β-lactam **3** (1.0 equiv.) in dry acetonitrile (5 mL) was added the appropriate isocyanate (1.5 equiv.) and the resulting reaction mixture was stirred for 20 h at room temperature. Acetonitrile was then concentrated under reduced pressure and the obtained crude product was purified by SiO_2_ flash column chromatography with *n*-hexane/ethyl acetate (4/1, *v*/*v*). The pure *trans*-β-lactam urea was finally obtained by trituration with *n*-hexane.

##### Allyl-3-[(±)-*trans*-1-(4-fluorophenyl)-2-(4-methoxyphenyl)-4-oxoazetidin-3-yl]urea (**4a**)

Compound **4a** was prepared from **3** (50 mg, 0.175 mmol) and allyl isocyanate (23.2 μL, 0.263 mmol) according to the general synthetic procedure, producing a white solid (57.7 mg, 89%). *R*_f_ = 0.22 (chloroform/ethyl acetate = 4/1, *v*/*v*), m.p. 86.1−88.5 °C. FTIR (ATR, cm^−1^): 3346, 2933, 2838, 1745, 1636, 1612, 1560, 1507, 1426, 1288, 1247, 1224, 1175, 1137, 1102, 923, 830. ^1^H NMR (600 MHz, CDCl_3_), *δ*/ppm: 7.25−7.17 (m, 4H), 6.92−6.84 (m, 4H), 6.04 (d, *J* = 6.8 Hz, 1H), 5.84−5.74 (m, 1H), 5.37 (t, *J* = 5.8 Hz, 1H), 5.16 (dd, *J* = 17.2, 1.6 Hz, 1H), 5.07 (dd, *J* = 10.3, 1.5 Hz, 1H), 5.01 (d, *J* = 2.3 Hz, 1H), 4.42 (dd, *J* = 6.8, 2.3 Hz, 1H), 3.79 (s, 3H), 3.78−3.67 (m, 2H). ^13^C NMR (151 MHz, CDCl_3_), *δ*/ppm: 166.3, 160.1, 159.4 (d, *J* = 244.5 Hz), 157.5, 135.1, 133.6 (d, *J* = 2.7 Hz), 128.2, 127.6, 119.3 (d, *J* = 7.9 Hz), 116.0 (d, *J* = 22.8 Hz), 115.9, 114.8, 67.2, 63.9, 55.5, 42.9. HR-MS (ESI-QTOF) *m*/*z*: calcd. for C_20_H_20_FN_3_O_3_, [M + H]^+^, 370.1567; found 370.1579.

##### 1-Hexyl-3-[(±)-*trans*-1-(4-fluorophenyl)-2-(4-methoxyphenyl)-4-oxoazetidin-3-yl]urea (**4b**)

Compound **4b** was prepared from **3** (50 mg, 0.175 mmol) and hexyl isocyanate (38.2 μL, 0.263 mmol) according to the general synthetic procedure, producing a white solid (64.3 mg, 89%). R_f_ = 0.32 (chloroform/ethyl acetate = 4/1, *v*/*v*), m.p. 58.3−62.4 °C. FTIR (ATR, cm^−1^): 3352, 2956, 2929, 2858, 1748, 1639, 1563, 1507, 1386, 1247, 1226, 1175, 1136, 1031, 830. ^1^H NMR (600 MHz, CDCl_3_), δ/ppm: 7.25−7.18 (m, 4H), 6.91−6.85 (m, 4H), 5.95 (d, *J* = 6.7 Hz, 1H), 5.22 (t, *J* = 5.6 Hz, 1H), 5.01 (d, *J* = 2.2 Hz, 1H), 4.40 (dd, *J* = 6.7, 2.3 Hz, 1H), 3.79 (s, 3H), 3.16−3.00 (m, 2H), 1.43 (p, *J* = 7.2 Hz, 2H), 1.32−1.19 (m, 6H), 0.90−0.83 (m, 3H). ^13^C NMR (151 MHz, CDCl_3_), δ/ppm: 166.5, 160.0, 159.4 (d, *J* = 244.0 Hz), 157.5, 133.6 (d, *J* = 2.7 Hz), 128.3, 127.6, 119.2 (d, *J* = 7.8 Hz), 116.0 (d, *J* = 22.7 Hz), 114.7, 67.3, 64.0, 55.4, 40.6, 31.7, 30.2, 26.7, 22.7, 14.2. HR-MS (ESI-QTOF) m/z: calcd. for C_23_H_28_FN_3_O_3_, [M + H]^+^, 414.2193; found 414.2158.

##### 1-Cyclopentyl-3-[(±)-*trans*-1-(4-fluorophenyl)-2-(4-methoxyphenyl)-4-oxoazetidin 3-yl]urea (**4c**)

Compound **4c** was prepared from **3** (50 mg, 0.175 mmol) and cyclopentyl isocyanate (29.6 μL, 0.263 mmol) according to the general synthetic procedure, producing a white solid (59.5 mg, 86%). R_f_ = 0.30 (chloroform/ethyl acetate = 4/1, *v*/*v*), m.p. 138.7−143.7 °C. FTIR (ATR, cm^−1^): 3342, 2959, 1747, 1633, 1556, 1509, 1385, 1289, 1250, 1226, 1175, 1131, 1101, 1031, 830. ^1^H NMR (600 MHz, CDCl_3_), δ/ppm: 7.28−7.19 (m, 4H), 6.93−6.85 (m, 4H), 5.74 (d, *J* = 6.5 Hz, 1H), 5.09 (d, *J* = 7.3 Hz, 1H), 4.99 (d, *J* = 2.2 Hz, 1H), 4.43 (dd, *J* = 6.6, 2.2 Hz, 1H), 3.97 (h, *J* = 6.8 Hz, 1H), 3.79 (s, 3H), 1.90 (tdd, *J* = 13.2, 10.1, 6.2 Hz, 2H), 1.67−1.58 (m, 2H), 1.48−1.58 (m, 2H), 1.40−1.28 (m, 2H). ^13^C NMR (151 MHz, CDCl_3_), δ/ppm: 166.2, 160.1, 159.4 (d, *J* = 244.0 Hz), 157.1, 133.7 (d, *J* = 2.4 Hz), 128.3, 127.6, 119.2, 116.0 (d, *J* = 22.6 Hz), 114.7, 67.3, 64.1, 55.5, 52.3, 33.6, 33.5, 23.7. HR MS (ESI-QTOF) m/z: calcd. for C_22_H_24_FN_3_O_3_, [M + H]^+^, 398.1880; found 398.1871.

##### 1-[(±)-*Trans*-1-(4-fluorophenyl)-2-(4-methoxyphenyl)-4-oxoazetidin-3-yl)-3-(furan-2 ylmethyl]urea (**4d**)

Compound **4d** was prepared from **3** (50 mg, 0.175 mmol) and furfuryl isocyanate (28.2 μL, 0.263 mmol) according to the general synthetic procedure, producing a white solid (64.4 mg, 90%). R_f_ = 0.22 (chloroform/ethyl acetate = 4/1, *v*/*v*), m.p. 188.8−189.9 °C. FTIR (ATR, cm*^−^*^1^): 3333, 1732, 1650, 1611, 1556, 1508, 1427, 1388, 1426, 1247, 1218, 1176, 1146, 1104, 1076, 1028, 1010, 833, 743. ^1^H NMR (600 MHz, CDCl_3_), δ/ppm: 7.28−7.27 (m, 1H), 7.23−7.17 (m, 4H), 6.91−6.84 (m, 4H), 6.26 (dd, *J* = 3.2, 1.8 Hz, 1H), 6.17 (d, *J* = 2.9 Hz, 1H), 5.99 (d, *J* = 6.7 Hz, 1H), 5.60 (t, *J* = 5.7 Hz, 1H), 5.00 (d, *J* = 2.2 Hz, 1H), 4.40 (dd, *J* = 6.7, 2.3 Hz, 1H), 4.32 (dd, *J* = 15.6, 5.8 Hz, 1H), 4.25 (dd, *J* = 15.6, 5.5 Hz, 1H), 3.79 (s, 3H). ^13^C NMR (151 MHz, CDCl_3_), δ/ppm: 166.2, 160.1, 159.4 (d, *J* = 244.3 Hz), 157.2, 152.2, 142.2, 133.5 (d, *J* = 2.5 Hz), 128.1, 127.6, 119.3 (d, *J* = 7.9 Hz), 116.0 (d, *J* = 22.8 Hz), 114.8, 110.6, 107.2, 67.2, 63.9, 55.5, 37.4. HR-MS (ESI-QTOF) m/z: calcd. for C_22_H_20_FN_3_O_4_, [M + H]^+^, 410.1516; found 410.1509.

##### 1-Benzyl-3-[(±)-*trans*-1-(4-fluorophenyl)-2-(4-methoxyphenyl)-4-oxoazetidin-3-yl]urea (**4e**)

Compound **4e** was prepared from **3** (50 mg, 0.175 mmol) and benzyl isocyanate (32.5 μL, 0.263 mmol) according to the general synthetic procedure, producing a white solid (65.9 mg, 90%). R_f_ = 0.28 (chloroform/ethyl acetate = 4/1, *v*/*v*), m.p. 164.1−167.3 °C. FTIR (ATR, cm^−1^): 3358, 3264, 3035, 2965, 2929, 1735, 1646, 1555, 1506, 1425, 1384, 1243, 1176, 1155, 1105, 1022, 841, 748, 706. ^1^H NMR (600 MHz, CDCl_3_), δ/ppm: 7.30−7.24 (m, 2H), 7.25−7.14 (m, 7H), 6.89−6.83 (m, 4H), 5.97 (d, *J* = 6.6 Hz, 1H), 5.57 (t, *J* = 5.8 Hz, 1H), 4.97 (d, *J* = 2.3 Hz, 1H), 4.38 (dd, *J* = 6.7, 2.3 Hz, 1H), 4.30 (dd, *J* = 14.7, 5.8 Hz, 1H), 4.23 (dd, *J* = 14.9, 5.6 Hz, 1H), 3.78 (s, 3H). ^13^C NMR (151 MHz, CDCl_3_), δ/ppm: 166.2, 160.1, 159.4 (d, *J* = 244.0 Hz), 157.5, 139.0, 133.5 (d, *J* = 2.6 Hz), 128.7, 128.1, 127.6, 127.4, 119.2 (d, *J* = 7.9 Hz), 116.0 (d, *J* = 22.8 Hz), 114.7, 67.2, 63.9, 55.5, 44.4. HR-MS (ESI-QTOF) m/z: calcd. for C_24_H_22_FN_3_O_3_, [M + H]^+^, 420.1723; found 420.1700.

##### 1-(4-(Tert-Butyl)phenyl)-3-[(±)-*trans*-1-(4-fluorophenyl)-2-(4-methoxyphenyl)-4-oxoazetidin-3-yl]urea (**4f**)

Compound **4f** was prepared from **3** (50 mg, 0.175 mmol) and 4-tert-butyl isocyanate (46.7 μL, 0.263 mmol) according to the general synthetic procedure, producing a white solid (73.7 mg, 91%). R_f_ = 0.47 (chloroform/ethyl acetate = 4:1, *v*/*v*), m.p. 195.4−196.0 °C. FTIR (ATR, cm^−1^): 3332, 2961, 1748, 1657, 1603, 1542, 1507, 1387, 1292, 1249, 1228, 1176, 1138, 1102, 1032, 831. ^1^H NMR (600 MHz, CDCl_3_), δ/ppm: 7.35 (s, 1H), 7.23−7.18 (m, 6H), 7.18−7.13 (m, 2H), 6.90−6.84 (m, 4H), 6.18 (d, *J* = 6.8 Hz, 1H), 5.06 (d, *J* = 2.3 Hz, 1H), 4.46 (dd, *J* = 6.8, 2.3 Hz, 1H), 3.78 (s, 3H), 1.25 (s, 9H). ^13^C NMR (151 MHz, CDCl_3_), δ/ppm: 166.2, 160.1, 159.5 (d, *J* = 244.5 Hz), 155.5, 147.1, 135.4, 133.5 (d, *J* = 2.6 Hz), 128.0, 127.7, 126.1, 121.0, 119.4 (d, *J* = 8.0 Hz), 116.0 (d, *J* = 22.8 Hz), 114.8, 67.1, 63.8, 55.5, 34.4, 31.5. HR-MS (ESI-QTOF) m/z: calcd. for C_27_H_28_FN_3_O_3_, [M + H]^+^, 462.2193; found 462.2183.

##### 1-(3-Chloro-4-methylphenyl)-3-[(±)-*trans*-1-(4-fluorophenyl)-2-(4-methoxyphenyl)-4-oxoazetidin-3-yl]urea (**4g**)

Compound **4g** was prepared from **3** (50 mg, 0.175 mmol) and 3-chloro-4-methyl isocyanate (36.0 μL, 0.263 mmol) according to the general synthetic procedure, producing a white solid (69.1 mg, 87%). R_f_ = 0.41 (chloroform/ethyl acetate = 4/1, *v*/*v*), m.p. 174.2−178.8 °C. FTIR (ATR, cm^−1^): 3327, 2931, 1741, 1658, 1588, 1542, 1508, 1384, 1290, 1225, 1175, 1136, 1102, 1030, 830. ^1^H NMR (600 MHz, CDCl_3_), δ/ppm: 7.62 (s, 1H), 7.27 (d, *J* = 2.0 Hz, 1H), 7.23−7.15 (m, 4H), 6.85−6.82 (m, 6H), 6.48 (d, *J* = 6.9 Hz, 1H), 5.12 (d, *J* = 2.3 Hz, 1H), 4.39 (dd, *J* = 6.9, 2.3 Hz, 1H), 3.79 (s, 3H), 2.20 (s, 3H). ^13^C NMR (151 MHz, CDCl_3_), δ/ppm: 166.8, 160.4, 160.2, 159.6 (d, *J* = 244.7 Hz), 155.1, 137.2, 134.4, 133.3 (d, *J* = 2.6 Hz), 130.9, 130.7, 127.7, 127.6, 120.2, 119.5 (d, *J* = 7.9 Hz), 118.2, 116.1 (d, *J* = 22.7 Hz), 114.8, 66.9, 63.7, 55.5, 19.4. HR-MS (ESI-QTOF) m/z: calcd. for C_24_H_21_ClFN_3_O_3_, [M + H]^+^, 454.1333; found 454.1257.

##### 1-(3,5-Dimethylphenyl)-3-[(±)-*trans*-1-(4 fluorophenyl)-2-(4-methoxyphenyl)-4-oxoazetidin 3-yl]urea (**4h**)

Compound **4h** was prepared from **3** (50 mg, 0.175 mmol) and 3,5-dimethylphenyl isocyanate (37.0 μL, 0.263 mmol) according to the general synthetic procedure, producing a white solid (68.3 mg, 90%). *R*_f_ = 0.44 (chloroform/ethyl acetate = 4/1, *v*/*v*), m.p. 163.2−164.2 °C. FTIR (ATR, cm^−1^): 3320, 2918, 2851, 1753, 1645, 1612, 1566, 1508, 1387, 1279, 1247, 1224, 1175, 1153, 1102, 1031, 831. ^1^H NMR (600 MHz, CDCl_3_), *δ*/ppm: 7.40 (s, 1H), 7.32−7.06 (m, 4H), 6.94−6.75 (m, 6H), 6.61 (s, 1H), 6.32 (d, *J* = 6.9 Hz, 1H), 5.08 (d, *J* = 2.3 Hz, 1H), 4.40 (dd, *J* = 6.8, 2.3 Hz, 1H), 3.78 (s, 3H), 2.15 (s, 6H). ^13^C NMR (151 MHz, CDCl_3_), *δ*/ppm: 166.6, 160.1, 159.5 (d, *J* = 244.2 Hz), 155.4, 138.8, 138.1, 133.4 (d, *J* = 2.7 Hz), 128.0, 127.6, 125.5, 119.4 (d, *J* = 8.0 Hz), 118.3, 116.0 (d, *J* = 22.8 Hz), 114.7, 67.0, 63.7, 55.4, 21.4. HR-MS (ESI-QTOF) *m*/*z*: calcd. for C_25_H_24_FN_3_O_3_, [M +H]^+^, 434.1880; found 434.1871.

##### 1-(2,6-Dimethylphenyl)-3-[(±)-*trans*-1-(4-fluorophenyl)-2-(4-methoxyphenyl)-4-oxoazetidin 3-yl]urea (**4i**)

Compound **4i** was prepared from **3** (50 mg, 0.175 mmol) and 2,6-dimethylphenyl isocyanate (36.6 μL, 0.263 mmol) according to the general synthetic procedure, producing a white solid (67.2 mg, 89%). *R*_f_ = 0.30 (chloroform/ethyl acetate = 4/1, *v*/*v*), m.p. 127.2−128.9 °C. FTIR (ATR, cm^−1^): 3311, 2961, 1753, 1638, 1612, 1548, 1508, 1384, 1248, 1226, 1175, 1134, 1101, 1030, 831. ^1^H NMR (600 MHz, CDCl_3_), *δ*/ppm: 7.25−7.17 (m, 4H), 7.16−7.03 (m, 3H), 6.92−6.82 (m, 3H), 6.39 (s, 1H), 4.96 (d, *J* = 2.3 Hz, 1H), 4.44 (dd, *J* = 6.8, 2.3 Hz, 1H), 3.77 (s, 3H), 2.29 (s, 6H). ^13^C NMR (151 MHz, CDCl_3_), *δ*/ppm: 166.0, 160.1, 159.3 (d, *J* = 243.7 Hz), 156.2, 133.7 (d, *J* = 2.6 Hz), 128.9, 128.2, 127.5, 119.1 (d, *J* = 7.8 Hz), 115.9 (d, *J* = 22.8 Hz), 114.8, 67.2, 63.9, 55.5, 18.5. HR-MS (ESI-QTOF) *m*/*z*: calcd. for C_25_H_24_FN_3_O_3_, [M + H]^+^, 434.1880; found 434.1859.

#### 3.1.6. General Procedure for the Preparation of *Syn*/*anti*-(±)-3,5-disubstituted Hydantoins **5a**–**i**

To a stirred solution of corresponding β-lactam urea **4a**–**i** (1.0 mmol) in dry methanol at 65 °C was added a solution of 25% sodium methoxide in methanol (1.1 mmol). The reaction mixture was allowed to reflux for 1 h (the progress of the reaction was monitored by TLC) and the resulting reaction mixture was then treated with brine and extracted with chloroform (3 × 15 mL). The organic layer was dried over anhydrous sodium sulfate, filtered and concentrated in vacuo to give a residue. The residue was then purified by SiO_2_ flash column chromatography with chloroform/ethyl acetate (2:1 or 4:1) as the eluent solvent. The *syn*/*anti*-ratio in the product was determined by RP-HPLC and ^1^H NMR spectroscopy. The mixture of diastereomeric hydantoins was then fractionated by preparative RP-HPLC using a Zorbax Extend C18 PrepHT column (250 × 9.4 mm I.D., 5-μm particle size, 300 Å pore size, Agilent Technologies). A linear gradient AB (method D–F) was used at a flow rate of 17 mL/min, with mobile phase A consisting of water and mobile phase B of acetonitrile. The chromatograms were monitored at 254 nm. Collected fractions of *syn*- and *anti*-hydantoins were extracted with chloroform (3 × 20 mL). The purity of the *syn*- and *anti*-isomers fraction was verified by analytical RP-HPLC as described in the Materials and Methods section. The organic phase was dried over anhydrous sodium sulfate and filtered, and the resulting filtrate was concentrated in vacuo to obtain the pure *syn*- and *anti*-isomers. 

##### *Syn*/*anti*-3-allyl-5-{[(4-fluorophenyl)amino](4-methoxyphenyl)methyl}-imidazolidine-2,4-dione (**5a**)

Compound *syn*/*anti*-**5a** was prepared from *trans*-β-lactam urea **4a** (40 mg, 0.108 mmol) and a 25 wt.% solution of sodium methoxide in methanol (24.8 μL, 0.119 mmol) according to the general synthetic procedure, producing a white solid (34.3 mg, 85.8%). The diastereomeric ratio of *syn*/*anti*-isomers (52.5:47.5) was determined by HPLC (retention time: *anti*-isomer 29.5 min, *syn*-isomer 31.3 min, method A) of the crude reaction mixture. The diastereomeric mixture of the *syn*/*anti*-isomer **5a** was further separated by preparative HPLC (retention time: *anti*-isomer 18.0 min, *syn*-isomer 19.6 min, method D) to obtain isomers *syn*-**5a** (10 mg) and *anti*-**5a** (11 mg). *R*_f_ (*syn*/*anti*-**5a**) = 0.42 (chloroform/ethyl acetate = 2/1, *v*/*v*). 

*Syn*-**5a**: m.p. 168.4–170.7 °C. FTIR (ATR, cm^−1^): 3351, 2933, 2837, 1774, 1702, 1610, 1508, 1445, 1409, 1344, 1249, 1216, 1177, 1108, 989, 930, 823. ^1^H NMR (600 MHz, CD_3_CN), *δ*/ppm: 7.28 (d, *J* = 8.8 Hz, 2H), 6.89 (d, *J* = 8.8 Hz, 2H), 6.81 (t, *J* = 8.8 Hz, 2H), 6.61 (dd, *J* = 8.8, 4.8 Hz, 2H), 6.22 (br s, 1H), 5.71 (ddt, *J* = 17.1, 10.4 Hz, 5.0 Hz, 1H), 5.26 (d, *J* = 10.6 Hz, 1H), 5.04 (dq, *J* = 10.5, 1.4 Hz, 1H), 5.02 (dq, *J* = 17.3, 1.6 Hz, 1H), 4.91 (dd, *J* = 10.6, 3.3 Hz, 1H), 4.38 (dd, *J* = 3.3, 2.2 Hz, 1H), 3.98 (ddt, *J* = 16.5, 4.8, 1.8 Hz, 1H), 3.93 (ddt, *J* = 16.5, 4.8 Hz, 1.8 Hz, 1H), 3.75 (s, 3H). ^13^C NMR (151 MHz, CD_3_CN), *δ*/ppm: 173.2, 160.3, 158.2, 156.7 (d, *J* = 233,6 Hz), 144.4 (d, *J* = 1,7 Hz), 132.9, 131.5, 129.4, 116.6, 116.3 (d, *J* = 22.6 Hz), 116.0 (d, *J* = 7.5 Hz), 115.0, 63.3, 57.4, 56.0, 41.0. HR-MS (ESI-QTOF) *m*/*z*: calcd. for C_20_H_20_FN_3_O_3_, [M + H]^+^, 370.1567; found 370.1555. 

*Anti*-**5a**: m.p. 155.0–156.9 °C. FTIR (ATR, cm^−1^): 3351, 2933, 2837, 1774, 1702, 1610, 1508, 1445, 1409, 1344, 1249, 1216, 1177, 1108, 989, 930, 823. ^1^H NMR (600 MHz, CD_3_CN), *δ*/ppm: 7.26 (d, *J* = 8.8 Hz, 2H), 6.84 (t, *J* = 9.0 Hz, 2H), 6.84 (d, *J* = 8.4 Hz, 2H), 6.62 (dd, *J* = 8.8, 4.4 Hz, 2H), 6.38 (s, 1H), 5.43 (ddt, *J* = 17.1, 10.5, 4.9 Hz, 1H), 5.00 (d, *J* = 7.3 Hz, 1H), 4.85 (dq, *J* = 10.3, 1.1 Hz, 1H), 4.80 (dd, *J* = 6.4, 3.9 Hz, 1H), 4.52 (dd, *J* = 3.7, 1.8 Hz, 1H), 4.52 (dq, *J* = 17.2, 1.5 Hz, 1H), 3.82 (ddt, *J* = 16.1, 5.1, 1.5 Hz, 1H), 3.74 (s, 3H), 3.73 (ddt, *J* = 16.2, 5.1, 1.8 Hz, 1H). ^13^C NMR (151 MHz, CD_3_CN), *δ*/ppm: 172.5, 160.7, 157.6, 156.9 (d, *J* = 233,6 Hz), 144.4 (d, *J* = 1,7 Hz), 132.6, 130.4, 129.6, 116.5, 116.4 (d, *J* = 22.6 Hz), 116.0 (d, *J* = 7.5 Hz), 114.6, 62.1, 59.5, 56.0, 40.8. HR-MS (ESI QTOF) *m*/*z*: calcd. for C_20_H_20_FN_3_O_3_, [M + H]^+^, 370.1567; found 370.1555.

##### *Syn*/*anti*-{[(4-fluorophenyl)amino](4-methoxyphenyl)methyl}-3-hexylimidazolidine-2,4-dione (**5b**)

Compound *syn*/*anti*-**5b** was prepared from *trans*-β-lactam urea **4b** (40 mg, 0.097 mmol) and a 25 wt.% solution of sodium methoxide in methanol (24.5 μL, 0.107 mmol) according to the general synthetic procedure, producing a white solid (36.7 mg, 91.8%). The diastereomeric ratio of *syn*/*anti* isomers (50.1:49.9) was determined by HPLC (retention time: *anti*-isomer 40.5 min, *syn*-isomer 41.9 min, method C) of the crude reaction mixture. The diastereomeric mixture of the *syn*/*anti*-isomer **5b** was further separated by preparative HPLC (retention time: *anti*-isomer 29.8 min, *syn*-isomer 30.6 min, method F) to obtain isomers *syn*-**5b** (15 mg) and *anti*-**5b** (13 mg). *R*_f_ (*syn*/*anti*-**5b**) = 0.39 (chloroform/ethyl acetate = 4/1, *v*/*v*). 

*Syn*-**5b**: m.p. 137.4–139.3 °C. FTIR (ATR, cm^−1^): 3387, 3359, 2950, 2929, 2856, 1762, 1709, 1612, 1581, 1506, 1455, 1422, 1359, 1256, 1178, 1113, 1030, 985, 820. ^1^H NMR (600 MHz, CD_3_CN), *δ*/ppm: 7.25 (d, *J* = 8.7 Hz, 2H), 6.84–6.77 (m, 4H), 6.60–6.57 (m, 2H), 6.15 (s, 1H), 5.25 (d, *J* = 10.5 Hz, 1H), 4.88 (dd, *J* = 10.7, 3.2 Hz, 1H), 4.32 (dd, *J* = 3.2, 2.1 Hz, 1H), 3.74 (s, 3H), 3.40–3.33 (m, 1H), 3.33–3.27 (m, 1H), 1.45–1.37 (m, 2H), 1.25–1.02 (m, 6H), 0.86–0.83 (m, 3H). ^13^C NMR (151 MHz, CD_3_CN), *δ*/ppm: 172.5, 160.1, 158.4, 156.4 (d, *J* = 235.0 Hz), 142.7 (d, *J* = 2.1 Hz), 131.2, 129.2, 116.0 (d, *J* = 22.5 Hz), 115.7 (d, *J* = 7.4 Hz), 114.6, 62.7, 59.2, 55.7, 38.8, 32.0, 28.5, 26.8, 23.0, 14.1. HR-MS (ESI-QTOF) *m*/*z*: calcd. for C_23_H_28_FN_3_O_3_, [M + H]^+^, 414.2193; found 414.2184. 

*Anti*-**5b**: m.p. 164.2–167.5 °C. FTIR (ATR, cm^−1^): 3387, 3359, 2950, 2929, 2856, 1762, 1709, 1612, 1581, 1506, 1455, 1422, 1359, 1256, 1178, 1113, 1030, 985, 820. ^1^H NMR (600 MHz, CD_3_CN), *δ*/ppm: 7.27 (d, *J* = 8.7 Hz, 2H), 6.89–6.44 (m, 4H), 6.67–6.60 (m, 2H), 6.34 (s, 1H), 4.98 (d, *J* = 7.5 Hz, 1H), 4.79 (dd, *J* = 7.9, 3.6 Hz, 1H), 4.47 (dd, *J* = 3.6, 1.7 Hz, 1H), 3.72 (s, 3H), 3.23–3.16 (m, 1H), 3.14–3.07 (m, 1H), 1.25–1.02 (m, 6H), 0.95–0.86 (m, 2H), 0.86–0.83 (m, 3H). ^13^C NMR (151 MHz, CD_3_CN), *δ*/ppm: 173.3, 160.3, 157.9, 156.8 (d, *J* = 235.8 Hz), 142.1 (d, *J* = 2.1 Hz), 130.1, 129.3, 116.3 (d, *J* = 22.5 Hz), 115.9 (d, *J* = 7.4 Hz), 114.2, 61.6, 57.3, 55.6, 38.6, 31.9, 28.2, 26.7, 22.9, 14.1. HR-MS (ESI-QTOF) *m*/*z*: calcd. for C_23_H_28_FN_3_O_3_, [M + H]^+^, 414.2193; found 414.2184.

##### *Syn*/*anti*-3-cyclopentyl-5-[[(4-fluorophenyl)amino](4-methoxyphenyl)methyl}-imidazolidine-2,4-dione (**5c**)

Compound *syn*/*anti*-**5c** was prepared from *trans*-β-lactam urea **4c** (40 mg, 0.101 mmol) and a 25 wt.% solution of sodium methoxide in methanol (25.4 μL, 0.111 mmol) according to the general synthetic procedure, producing a white solid (31.8 mg, 79.5%). The diastereomeric ratio of *syn*/*anti* isomers (51.8:48.2) was determined by HPLC (retention time: *anti*-isomer 41.2 min, *syn*-isomer 42.3 min, method B) of the crude reaction mixture. The diastereomeric mixture of the *syn*/*anti*-isomer **5c** was further separated by preparative HPLC (retention time: *anti*-isomer 23.2 min, *syn*-isomer 24.1 min, method F) to obtain isomers *syn*-**5c** (14 mg) and *anti*-**5c** (13 mg). *R*_f_ (*syn*/*anti*-**5c**) = 0.41 (chloroform/ethyl acetate = 4/1, *v*/*v*). 

*Syn*-**5c**: m.p. 211.0–211.8 °C. FTIR (ATR, cm^−1^): 3347, 3231, 2959, 1762, 1699, 1613, 1510, 1432, 1352, 1308, 1282, 1258, 1221, 1131, 1157, 1111, 965, 832. ^1^H NMR (600 MHz, CD_3_CN), *δ*/ppm: 7.26 (d, *J* = 8.8 Hz, 2H), 6.87 (d, *J* = 8.8 Hz, 2H), 6.81 (t, *J* = 8.9 Hz, 2H), 6.61–6.57 (m, 2H), 6.15 (s, 1H), 5.26 (d, *J* = 10.5 Hz, 1H), 4.84 (dd, *J* = 10.5, 3.3 Hz, 1H), 4.26 (dd, *J* = 3.4, 2.2 Hz, 1H), 4.10 4.02 (m, 1H), 3.74 (s, 3H), 1.90–1.75 (m, 4H), 1.66–1.57 (m, 2H), 1.57–1.49 (m, 2H). ^13^C NMR (151 MHz, CD_3_CN), *δ*/ppm: 173.5, 160.3, 158.4, 156.6 (d, *J* = 233.2 Hz), 144.4 (d, *J* = 1.6 Hz), 131.3, 129.5, 116.2 (d, *J* = 22.5 Hz), 115.9 (d, *J* = 7.4 Hz), 114.8, 62.1, 59.7, 57.8, 52.2, 29.6, 29.5, 25.8. HR-MS (ESI-QTOF) *m*/*z*: calcd. for C_22_H_24_FN_3_O_3_, [M + H]^+^, 398.1880; found 398.1870. 

*Anti*-**5c**: m.p. 141.1–142.5 °C. FTIR (ATR, cm^−1^): 3347, 3231, 2959, 1762, 1699, 1613, 1510, 1432, 1352, 1308, 1282, 1258, 1221, 1131, 1157, 1111, 965, 832. ^1^H NMR (600 MHz, CD_3_CN), *δ*/ppm: 7.24 (d, *J* = 8.7 Hz, 2H), 6.86–6.81 (m, 4H), 6.64 6.60 (m, 2H), 6.32 (s, 1H), 4.99 (d, *J* = 7.0 Hz, 1H), 4.77 (dd, *J* = 7.3, 3.6 Hz, 1H), 4.39 (dd, *J* = 3.7, 1.6 Hz, 1H), 4.29–4.15 (m, 1H), 3.73 (s, 3H), 1.75–1.66 (m, 4H), 1.57–1.42 (m, 2H), 1.50–1.42 (m, 2H). ^13^C NMR (151 MHz, CD_3_CN), *δ*/ppm: 172.8, 160.6, 158.0, 156.9 (d, *J* = 233.6 Hz), 144.4 (d, *J* = 1.7 Hz), 130.3, 129.5, 116.3 (d, *J* = 22.5 Hz), 116.0 (d, *J* = 7.6 Hz), 114.5, 61.3, 57.8, 56.0, 52.0, 29.5, 29.3, 25.7. HR-MS (ESI-QTOF) *m*/*z*: calcd. for C_22_H_24_FN_3_O_3_, [M + H]^+^, 398.1880; found 398.1870.

##### *Syn*/*anti*-5-{[(4-fluorophenyl)amino](4-methoxyphenyl)methyl)-3-(furan-2 ylmethyl}imidazolidine-2,4-dione (**5d**)

Compound *syn*/*anti*-**5d** was prepared from *trans*-β-lactam urea **4d** (40 mg, 0.098 mmol) and a 25 wt.% solution of sodium methoxide in methanol (24.7 μL, 0.108 mmol) according to the general synthetic procedure, producing a white solid (32.6 mg, 81.5%). The diastereomeric ratio of *syn*/*anti*-isomers (57.1:42.9) was determined by HPLC (retention time: *anti*-isomer 37.7 min, *syn*-isomer 40.3 min, method A) of the crude reaction mixture. The diastereomeric mixture of the *syn*/*anti*-isomer **5d** was further separated by preparative HPLC (retention time: *anti*-isomer 23.2 min, *syn*-isomer 25.2 min, method D) to obtain isomers *syn*-**5d** (13 mg) and *anti*-**5d** (11 mg). *R*_f_ (*syn*/*anti*-**5d**) = 0.43 (chloroform/ethyl acetate = 4/1, *v*/*v*). 

*Syn*-**5d**: m.p. 161.0–163.9 °C. FTIR (ATR, cm^−1^): 3356, 1776, 1705, 1610, 1508, 1441, 1348, 1248, 1215, 1178, 1129, 1031, 1010, 937, 822. ^1^H NMR (600 MHz, CD_3_CN), *δ*/ppm: 7.37 (dd, *J* = 1.8 Hz, 0.9 Hz, 1H), 7.26 (d, *J* = 8.6 Hz, 2H), 6.87–6.75 (m, 6H), 6.31 (dd, *J* = 3.2, 1.9 Hz, 1H), 6.25 (s, 1H), 6.16 (dd, *J* = 3.2, 0.8 Hz, 1H), 5.21 (d, *J* = 10.6 Hz, 1H), 4.89 (dd, *J* = 10.6, 3.2 Hz, 1H), 4.55 (d, *J* = 15.9 Hz, 1H), 4.49 (d, *J* = 15.5 Hz, 1H), 4.39 (dd, *J* = 3.3, 2.1 Hz, 1H), 3.74 (s, 3H). ^13^C NMR (151 MHz, CD_3_CN), *δ*/ppm: 172.8, 160.2, 157.7, 156.6 (d, *J* = 233.5 Hz), 150.6, 144.3 (d, *J* = 1.4 Hz), 143.3, 131.3, 129.3, 116.2 (d, *J* = 22.4 Hz), 115.9 (d, *J* = 7.4 Hz), 114.9, 111.4, 108.8, 63.1, 57.4, 55.9, 35.7. HR-MS (ESI-QTOF) *m*/*z*: calcd. for C_22_H_20_FN_3_O_4_, [M + H]^+^, 410.1516; found 410.1514. 

*Anti*-**5d**: m.p. 165.8–168,9 °C. FTIR (ATR, cm^−1^): 3356, 1776, 1705, 1610, 1508, 1441, 1348, 1248, 1215, 1178, 1129, 1031, 1010, 937, 822. ^1^H NMR (600 MHz, CD_3_CN), *δ*/ppm: 7.28 (dd, *J* = 1.8, 0.9 Hz, 1H), 7.20 (d, *J* = 8.7 Hz, 2H), 6.61–6.55 (m, 6H), 6.40 (s, 1H), 6.23 (dd, *J* = 3.3, 1.8 Hz, 1H), 5.80 (dd, *J* = 3.3, 0.9 Hz, 1H), 4.97 (d, *J* = 7.3 Hz, 1H), 4.80 (dd, *J* = 7.4, 3.7 Hz, 1H), 4.52 (dd, *J* = 3.8, 1.7 Hz, 1H), 4.38 (d, *J* = 15.0 Hz, 1H), 4.30 (d, *J* = 15.8 Hz, 1H), 3.73 (s, 3H). ^13^C NMR (151 MHz, CD_3_CN), *δ*/ppm: 172.2, 160.4, 157.2, 156.8 (d, *J* = 233.5 Hz), 150.3, 144.3 (d, *J* = 1.5 Hz), 143.2, 131.3, 130.1, 116.2 (d, *J* = 22.4 Hz), 115.9 (d, *J* = 7.4 Hz), 114.5, 111.2, 108.6, 62.0, 59.3, 55.8, 35.4. HR-MS (ESI-QTOF) *m*/*z*: calcd. for C_22_H_20_FN_3_O_4_, [M + H]^+^, 410.1516; found 410.1514.

##### *Syn*/*anti*-3-benzyl-5-{[(4-fluorophenyl)amino](4-methoxyphenyl)methyl}imidazolidine-2,4-dione (**5e**)

Compound *syn*/*anti*-**5e** was prepared from *trans*-β-lactam urea **4e** (47 mg, 0.112 mmol) and a 25 wt.% solution of sodium methoxide in methanol (28.1 μL, 0.123 mmol) according to the general synthetic procedure, producing a white solid (40.6 mg, 86.4%). The diastereomeric ratio of *syn*/*anti*-isomers (52.2:47.8) was determined by HPLC (retention time: *anti*-isomer 51.5 min, *syn*-isomer 54.4 min, method A) of the crude reaction mixture. The diastereomeric mixture of the *syn*/*anti*-isomer **5e** was further separated by preparative HPLC (retention time: *anti*-isomer 32.3 min, *syn*-isomer 33.8 min, method E) to obtain isomers *syn*-**5e** (16 mg) and *anti*-**5e** (14 mg). *R*_f_ (*syn*/*anti*-**5e**) = 0.46 (chloroform/ethyl acetate = 2/1, *v*/*v*). 

*Syn*-**5e**: m.p. 187.3–191.0 °C. FTIR (ATR, cm^−1^): 3359, 1771, 1706, 1611, 1509,1446, 1251, 1217, 1179, 10301, 822. ^1^H NMR (600 MHz, CD_3_CN), *δ*/ppm: 7.42–7.37 (m, 1H), 7.28 (d, *J* = 8.7 Hz, 2H), 7.21–7.10 (m, 3H), 6.96–6.73 (m, 8H), 6.67–6.53 (m, 2H), 6.36–6.30 (m, 1H), 6.21–6,15 (m, 1H), 6.30 (s, 1H), 4.91 (d, *J* = 7.5 Hz, 1H), 4.84 (dd, *J* = 10.8, 3.2 Hz, 1H), 4.45 (dd, *J* = 3.2, 2.1 Hz, 1H), 4.63 (d, *J* = 15.5 Hz, 1H), 4.52 (d, *J* = 15.5 Hz, 1H), 3.77 (s, 3H). ^13^C NMR (151 MHz, CD_3_CN), *δ*/ppm: 174.0, 160.9, 158.9, 157.4 (d, *J* = 233.3 Hz), 144.9 (d, *J* = 1.6 Hz), 138.2, 131.9, 130.1, 130.0, 128.8, 128.9, 116.9 (d, *J* = 22.7 Hz), 116.5 (d, *J* = 7.4 Hz), 115.50, 63.9, 58.0, 56.5, 43.0. HR MS (ESI-QTOF) *m*/*z*: calcd. for C_24_H_22_FN_3_O_3_, [M + H]^+^, 420.1723; found 420.1715. 

*Anti*-**5e**: m.p. 154.3–156.1 °C. FTIR (ATR, cm^−1^): 3359, 1771, 1706, 1611, 1509,1446, 1251, 1217, 1179, 10301, 822. ^1^H NMR (600 MHz, CD_3_CN), *δ*/ppm: 7.24 (d, *J* = 8.8 Hz, 2H), 7.21–7.10 (m, 3H), 6.90–6.74 (m, 4H), 6.74–6.67 (m, 2H), 6.66–6.58 (m, 2H), 6.54 (s, 1H), 5.21 (d, *J* = 7.5 Hz, 1H), 4.76 (dd, *J* = 7.5, 3.2 Hz, 1H), 4.60 (dd, *J* = 3.5, 1.8 Hz, 1H), 4.45 (d, *J* = 15.5 Hz, 1H), 4.29 (d, *J* = 14.8 Hz, 1H), 3.75 (s, 3H). ^13^C NMR (151 MHz, CD_3_CN), *δ*/ppm: 173.2, 161.2, 158.1, 157.6 (d, *J* = 233.8 Hz), 144.9 (d, *J* = 1.6 Hz), 137.8, 131.9, 131.0, 129.9, 128.8, 128.4, 116.9 (d, *J* = 22.4 Hz), 116.6 (d, *J* = 7.5 Hz), 115.2, 62.8, 59.8, 56.4, 42.7. HR-MS (ESI-QTOF) *m*/*z*: calcd. for C_24_H_22_FN_3_O_3_, [M + H]^+^, 420.1723; found 420.1715.

##### *Syn*/*anti*-3-[4-(tert-butyl)phenyl]-5-{[(4-fluorophenyl)amino](4-methoxyphenyl)methyl}imidazoli-dine-2,4-dione (**5f**)

Compound *syn*/*anti*-**5f** was prepared from *trans*-β-lactam urea **4f** (40 mg, 0.087 mmol) and a 25 wt.% solution of sodium methoxide in methanol (22.0 μL, 0.096 mmol) according to the general synthetic procedure, producing a white solid (35.5 mg, 88.8%). The diastereomeric ratio of *syn*/*anti*-isomers (66.0:34.0) was determined by HPLC (retention time: *anti*-isomer 50.6 min, *syn*-isomer 52.6 min, method C) of the crude reaction mixture. The diastereomeric mixture of the *syn*/*anti*-isomer **5f** was further separated by preparative HPLC (retention time: *anti*-isomer 31.9 min, *syn*-isomer 33.6 min, method F) to obtain isomers *syn*-**5f** (13 mg) and *anti*-**5f** (8 mg). *R*_f_ (*syn*/*anti*-**5f**) = 0.61 (chloroform/ethyl acetate = 4/1, *v*/*v*). 

*Syn*-**5f**: m.p. 233.9–234.6 °C. FTIR (ATR, cm^−1^): 3356, 3237, 2963, 1770, 1716, 1610, 1509, 1462, 1364, 1253, 1223, 1178, 1109, 1031, 828. ^1^H NMR (600 MHz, CD_3_CN), *δ*/ppm: 7.49 (d, *J* = 8.6 Hz, 2H), 7.42 (d, *J* = 8.6 Hz, 2H), 7.11 (d, *J* = 8.6 Hz, 2H), 6.94–6.88 (m, 2H), 6.68–6.62 (m, 2H), 6.48 (s, 1H), 5.39 (d, *J* = 10.5 Hz, 1H), 4.98 (dd, *J* = 10.5, 3.1 Hz, 1H), 4.50 (dd, *J* = 3.4, 2.0 Hz, 1H), 3.76 (s, 3H), 1.32 (s, 9H). ^13^C NMR (151 MHz, CD_3_CN), *δ*/ppm: 172.7, 160.4, 157.5, 156.7 (d, *J* = 233.5 Hz), 152.5, 144.3 (d, *J* = 1.5 Hz), 131.2, 129.5, 129.4, 127.3, 126.9, 116.3 (d, *J* = 22.5 Hz), 116.0 (d, *J* = 7.4 Hz), 114.6, 61.9, 59.8, 56.0, 31.5. HR-MS (ESI-QTOF) *m*/*z*: calcd. for C_27_H_28_FN_3_O_3_, [M + H]^+^, 462.2193; found 462.2187. 

*Anti*-**5f**: m.p. 87.5–90.5 °C. FTIR (ATR, cm^−1^): 3356, 3237, 2963, 1770, 1716, 1610, 1509, 1462, 1364, 1253, 1223, 1178, 1109, 1031, 828. ^1^H NMR (600 MHz, CD_3_CN), *δ*/ppm: 7.36–7.29 (m, 4H), 6.92–6.88 (m, 4H), 6.84–6.76 (m, 4H), 6.69 (s, 1H), 5.08 (d, *J* = 7.0 Hz, 1H), 4.89 (dd, *J* = 7.1, 3.4 Hz, 1H), 4.64 (dd, *J* = 3.5, 1.4 Hz, 1H), 3.76 (s, 3H), 1.30 (s, 9H). ^13^C NMR (151 MHz, CD_3_CN), *δ*/ppm: 172.1, 160.7, 157.1, 156.9 (d, *J* = 233.8 Hz), 152.52, 144.4 (d, *J* = 1.5 Hz), 130.5, 130.3, 130.2, 127.4, 126.9, 116.3 (d, *J* = 22.5 Hz), 116.0 (d, *J* = 7.4 Hz), 114.9, 63.0, 57.9, 55.9, 31.4. HR MS (ESI-QTOF) *m*/*z*: calcd. for C_27_H_28_FN_3_O_3_, [M + H]^+^, 462.2193; found 462.2187.

##### *Syn*/*anti*-3-(3-chloro-4-methylphenyl)-5{[(4-fluorophenyl)amino](4-methoxyphenyl)methyl}imidazolidine-2,4-dione (**5g**)

Compound *syn*/*anti*-**5g** was prepared from *trans*-β-lactam urea **4g** (42 mg, 0.093 mmol) and a 25 wt.% solution of sodium methoxide in methanol (23.3 μL, 0.102 mmol) according to the general synthetic procedure, producing a white solid (30.1 mg, 75.3%). The diastereomeric ratio of *syn*/*anti*-isomers (48.3:51.7) was determined by HPLC (retention time: *anti*-isomer 36.8 min, *syn*-isomer 38.3 min, method C) of the crude reaction mixture. The diastereomeric mixture of the *syn*/*anti*-isomer **5g** was further separated by preparative HPLC (retention time: *anti*-isomer 23.2 min, *syn*-isomer 24.4 min, method F) to obtain isomers *syn*-**5g** (11 mg) and *anti*-**5g** (12 mg). *R*_f_ (*syn*/*anti*-**5g**) = 0.37 (chloroform/ethyl acetate = 2/1, *v*/*v*). 

*Syn*-**5g**: m.p. = 207.3–208.9 °C. FTIR (ATR, cm^−1^): 3370, 1761, 1708, 1610, 1509, 1419, 1252, 1252, 1223, 1177, 1108, 1029, 819. ^1^H NMR (600 MHz, CD_3_CN), *δ*/ppm: 7.31–7.26 (m, 3H), 7.19 (d, *J* = 2.0 Hz, 1H), 7.09 (dd, *J* = 8.1, 2.0 Hz, 1H), 6.84 6.76 (m, 4H), 6.68–6.63 (m, 2H), 6.50 (s, 1H), 5.34 (d, *J* = 10.7 Hz, 1H), 4.98 (dd, *J* = 10.6, 3.1 Hz, 1H), 4.51 (dd, *J* = 3.3, 2.1 Hz, 1H), 3.76 (s, 3H), 2.34 (s, 3H). ^13^C NMR (151 MHz, CD_3_CN), *δ*/ppm: 171.7, 160.8, 157.0, 156.7 (d, *J* = 233.3 Hz), 144.2 (d, *J* = 1.8 Hz), 137.4, 134.6, 132.2, 131.5, 131.1, 130.3, 127.7, 126.0, 116.3 (d, *J* = 22.4 Hz), 116.1 (d, *J* = 7.6 Hz), 114.9, 63.0, 57.9, 55.9, 19.8. HR MS (ESI-QTOF) *m*/*z*: calcd. for C_24_H_21_ClFN_3_O_3_, [M + H]^+^, 454.1333; found 454.1326. 

*Anti*-**5g**: m.p. 128.1–131.6 °C. FTIR (ATR, cm^−1^): 3370, 1761, 1708, 1610, 1509, 1419, 1252, 1252, 1223, 1177, 1108, 1029, 819. ^1^H NMR (600 MHz, CD_3_CN), *δ*/ppm: 7.38–7.29 (m, 3H), 6.95–6.91 (m, 2H), 6.91–6.84 (m, 4H), 6.69 (s, 1H), 6.69–6.64 (m, 2H), 5.05 (d, *J* = 7.3 Hz, 1H), 4.88 (dd, *J* = 7.3, 3.6 Hz, 1H), 4.64 (dd, *J* = 3.6, 1.5 Hz, 1H), 3.76 (s, 3H), 2.37 (s, 3H). ^13^C NMR (151 MHz, CD_3_CN), *δ*/ppm: 172.3, 160.4, 156.5, 156. 9 (d, *J* = 233.8 Hz), 144.3 (d, *J* = 1.7 Hz), 137.3, 134.6, 132.24, 131.8, 129.4, 129.30, 127.7, 126.1, 116.3 (d, *J* = 22.3 Hz), 116.1 (d, *J* = 7.6 Hz), 114.6, 61.9, 59.8, 56.0, 19.9. HR-MS (ESI-QTOF) *m*/*z*: calcd. for C_24_H_21_ClFN_3_O_3_, [M + H]^+^, 454.1333; found 454.1326.

##### *Syn*/*anti*-3-(3,5-dimethylphenyl)-5-{[(4-fluorophenyl)amino](4-methoxyphenyl)methyl}imidazolidine-2,4-dione (**5h**)

Compound *syn*/*anti*-**5h** was prepared from *trans*-β-lactam urea **4h** (40 mg, 0.092 mmol) and a 25 wt.% solution of sodium methoxide in methanol (23.1 μL, 0.101 mmol) according to the general synthetic procedure, producing a white solid (23.5 mg, 58.8%). The diastereomeric ratio of *syn*/*anti*-isomers (64.3:35.7) was determined by HPLC (retention time: *anti*-isomer 45.0 min, *syn*-isomer 46.5 min, method B) of the crude reaction mixture. The diastereomeric mixture of the *syn*/*anti*-isomer **5h** was further separated by preparative HPLC (retention time: *anti*-isomer 31.0 min, *syn*-isomer 32.3 min, method E) to obtain isomers *syn*-**5h** (9 mg) and *anti*-**5h** (8 mg). *R*_f_ (*syn*/*anti*-**5h**) = 0.39 (chloroform/ethyl acetate = 2/1, *v*/*v*). 

*Syn*-**5h**: m.p. 202.3–206.4 °C. FTIR (ATR, cm^−1^): 3353, 3248, 1771, 1706, 1611, 1509, 1438, 1411, 1358, 1279, 1254, 1221, 1173, 1108, 1029, 824. ^1^H NMR (600 MHz, CD_3_CN), *δ*/ppm: 7.33 (dd, *J* = 8.7, 5.4 Hz, 4H), 7.04 (s, 1H), 6.75 (s, 2H), 6.68 6.63 (m, 4H), 6.41 (s, 1H), 5.37 (d, *J* = 10.6 Hz, 1H), 4.97 (dd, *J* = 10.6, 3.2 Hz, 1H), 4.49 (dd, *J* = 3.2, 2.1 Hz, 1H), 3.77 (s, 3H), 2.30 (s, 6H). ^13^C NMR (151 MHz, CD_3_CN), *δ*/ppm: 172.7, 160.4, 157.5, 156.7 (d, *J* = 233.5 Hz), 144.4 (d, *J* = 1.7 Hz), 139.9, 133.0, 131.2, 130.8, 129.5, 125.5, 116.3 (d, *J* = 22.3 Hz), 115.5 (d, *J* = 7.5 Hz), 114.9, 62.9, 57.9, 56.0, 21.2. HR-MS (ESI-QTOF) *m*/*z*: calcd. for C_25_H_24_FN_3_O_3_, [M + H]^+^, 434.1880; found 434.1876. 

*Anti*-**5h**: m.p. 101.4–104.0 °C. FTIR (ATR, cm^−1^): 3353, 3248, 1771, 1706, 1611, 1509, 1438, 1411, 1358, 1279, 1254, 1221, 1173, 1108, 1029, 824. ^1^H NMR (600 MHz, CD_3_CN), *δ*/ppm: 6.92 (dd, *J* = 8.7, 5.2 Hz, 4H), 6.99 (s, 1H), 6.85 (dt, *J* = 19.8, 8.9 Hz, 4H), 6.61 (s, 1H), 6.40 (s, 2H), 5.05 (d, *J* = 7.3 Hz, 1H), 4.88 (dd, *J* = 7.4, 3.6 Hz, 1H), 4.63 (dd, *J* = 3.6, 1.5 Hz, 1H), 3.77 (s, 3H), 2.25 (s, 6H). ^13^C NMR (151 MHz, CD_3_CN), *δ*/ppm: 172.0, 160.8, 157.1, 156.9 (d, J = 233.6 Hz), 144.3 (d, *J* = 1.8 Hz), 139.8, 132.7, 130.8, 129.4, 130.4, 125.5, 116.3 (d, *J* = 22.4 Hz), 115.0 (d, *J* = 7.5 Hz), 114.6, 61.9, 59.8, 55.9, 21.1. HR-MS (ESI-QTOF) *m*/*z*: calcd. for C_25_H_24_FN_3_O_3_, [M + H]^+^, 434.1880; found 434.1876.

##### *Syn*/*anti*-3-(2,6-Dimethylphenyl)-5-{[(4-fluorophenyl)amino](4-methoxyphenyl)methyl}imidazolidine-2,4-dione (**5i**)

Compound *syn*/*anti*-**5i** was prepared from *trans*-β-lactam urea **4i** (45 mg, 0.104 mmol) and a 25 wt.% solution of sodium methoxide in methanol (26.1 μL, 0.114 mmol) according to the general synthetic procedures, producing a white solid (34.1 mg, 75.8%). The diastereomeric ratio of *syn*/*anti*-isomers (58.8:41.2) was determined by HPLC (retention time: *anti*-isomer 41.7 min, *syn*-isomer 43.1 min, method B) of the crude reaction mixture. The diastereomeric mixture of the *syn*/*anti*-isomer **5i** was further separated by preparative HPLC (retention time: *anti*-isomer 28.8 min, *syn*-isomer 30.1 min, method F) to obtain isomers *syn*-**5i** (14 mg) and *anti*-**5i** (14 mg). *R*_f_ (*syn*/*anti*-**5i**) = 0.56 (chloroform/ethyl acetate = 4/1, *v*/*v*). 

*Syn*-**5i**: m.p. 168.6–170.8 °C. FTIR (ATR, cm^−1^): 3346, 2929, 1781, 1712, 1611, 1508, 1479, 1442, 1404, 1250, 1177, 1216, 1109, 1031, 823. ^1^H NMR (600 MHz, CD_3_CN), *δ*/ppm: 7.37 (d, *J* = 7.4 Hz, 2H), 7.24 (t, *J* = 7.6 Hz, 1H), 7.12 (t, *J* = 7.5 Hz, 2H), 6.93 (d, *J* = 7.4 Hz, 2H), 6.88 (d, *J* = 8.5 Hz, 2H), 6.69–6.62 (m, 2H), 6.75 (s, 1H), 5.42 (d, *J* = 11.2 Hz, 1H), 5.04 (dd, *J* = 10.3 Hz, 2.9 Hz, 1H), 4.66 (dd, *J* = 2.9, 2.1 Hz, 1H), 3.77 (s, 3H), 2.13 (s, 3H), 1.98 (s, 3H). ^13^C NMR (151 MHz, CD_3_CN), *δ*/ppm: 171.5, 160.9, 156.9 (d, *J* = 233.9 Hz), 156.7, 144.2 (d, *J* = 1.8 Hz), 138.0, 137.9, 131.5, 130.3, 129.5, 129.4, 129.2, 129.1, 116.3 (d, *J* = 22.5 Hz), 116.0 (d, *J* = 7.5 Hz), 115.0, 64.1, 57.3, 55.9, 18.0. HR-MS (ESI-QTOF) *m*/z: calcd. for C_25_H_24_FN_3_O_3_, [M + H]^+^, 434.1880; found 434.1872. 

*Anti*-**5i**: m.p. 115.7–117.3 °C. FTIR (ATR, cm^−1^): 3346, 2929, 1781, 1712, 1611, 1508, 1479, 1442, 1404, 1250, 1177, 1216, 1109, 1031, 823. ^1^H NMR (600 MHz, CD_3_CN), *δ*/ppm: 7.35 (d, *J* = 8.8 Hz, 2H), 7.18 (dd, *J* = 7.6, 3.2 Hz, 2H), 7.00 (ddt, *J* = 7.6, 1.5, 0.7 Hz, 1H), 6.88–6.80 (m, 4H), 6.69–6.62 (m, 2H), 6.50 (s, 1H), 5.02 (d, *J* = 7.4 Hz, 1H), 4.95 (dd, *J* = 7.9, 3.5 Hz, 1H), 4.86 (dd, *J* = 3.5, 1.8 Hz, 1H), 3.75 (s, 3H), 2.17 (s, 3H), 2.08 (s, 3H). ^13^C NMR (151 MHz, CD_3_CN), *δ*/ppm: 171.5, 160.9, 156.9 (d, *J* = 233.9 Hz), 156.7, 144.2 (d, *J* = 1.8 Hz), 138.0, 137.9, 131.2, 130.9, 130.8, 130.20, 129.1, 129.0, 116.3 (d, *J* = 22.5 Hz), 115.1 (d, *J* = 7.6 Hz), 114.9, 62.3, 59.1, 55.9, 16.7. HR-MS (ESI QTOF) *m*/*z*: calcd. for C_25_H_24_FN_3_O_3_, [M + H]^+^, 434.1872; found 434.1872.

#### 3.1.7. Computational Section

Conformational searches and preliminary DFT calculations were run with Spartan’20 (Wavefunction, Irvine, CA, USA) with default parameters, default grids, and convergence criteria. Geometry optimizations and frequency calculations, and TD-DFT calculations, were run with Gaussian’16 (Revision C.01) [76] with default grids and convergence criteria. The conformational search was run with the Monte Carlo algorithm implemented in Spartan’20 using Merck molecular force field. All structures thus obtained were pre-optimized with DFT at the B3LYP-D3/6-31G(d) level in vacuo. The structures were then reoptimized at B3LYP-D3BJ/6-311+G(d,p) in vacuo, and final energies were estimated at the same level including the PCM solvent model for acetonitrile. ECD calculations were run with TD-DFT at the CAM-B3LYP/def2-TZVP/PCM and B3LYP/def2-TZVP/PCM level, including the IEF-PCM solvent model for acetonitrile. The number of excited states (roots) was 40 for both functionals. VCD (frequency) calculations were run with DFT at the B3LYP-D3BJ/6-311+G(d,p)/PCM level, using geometries optimized at the same level of theory. All structures had only real (positive) frequencies. UV/ECD and IR/VCD spectra were plotted using the program SpecDis (version 1.71, Berlin, Germany, http://specdis-software.jimdo.com, accessed on 23 September 2024).

#### 3.1.8. Antiproliferative Activity of *Syn*/*anti*-(±)-3,5 Disubstituted Hydantoins **5a**–**i**

The antiproliferative activity of compounds **5a**–**i** was evaluated across three distinct human cell lines. The following cell lines were utilized in this study: HepG2 (liver hepatocellular carcinoma, HB 8055, from ATCC, Manassas, VA, USA), A2780 (ovarian carcinoma, 93112519, from ECACC, Porton Down, Wiltshire, UK), MCF7 (breast adenocarcinoma, HTB-22, from ATCC, Manassas, VA, USA), and one human non-tumor cell line, HFF1 (untransformed human fibroblasts, SCRC-1041, from ATCC, Manassas, VA, USA), employing the MTT assay [77,78]. The above cells were cultured as monolayers and maintained in various media, specifically Dulbecco’s modified Eagle’s medium (DMEM) or Roswell Park Memorial Institute (RPMI-1549) medium, which were enriched with differing concentrations of fetal bovine serum (FBS). Specifically, the A2780 cancer cells were cultured in RPMI-1540 medium (ATCC, Manassas, VA, USA) supplemented with 10% FBS. The MCF7 carcinoma cells were cultured in DMEM supplemented with 10% FBS and insulin (0.01 mg/mL). The HepG2 cells were cultured in DMEM supplemented with 10% FBS, whereas the HFF1 cells were cultured in DMEM containing 15% FBS. All cell lines were incubated in a humidified atmosphere with 5% CO_2_ at 37 °C. The growth inhibition activity was assessed according to the slightly modified procedure of the National Cancer Institute, Developmental Therapeutics Program [79]. On day 0, the cells were inoculated onto standard 96-well microtiter plates. The cell concentrations were calibrated based on the population doubling time (PDT). The cell concentrations were 9.6 × 10^3^ per mL for HepG2, 1.3 × 10^4^ per mL for A2780, 1.6 × 10^3^ per mL for MCF7, and 1.6 × 10^3^ per mL for HFF1. All compounds were prepared as stock solutions in DMSO at a concentration of 0.04 mol/L. On the subsequent day, the cells were treated with a range of concentrations of compounds **5a**–**i** (from 10⁻^8^ to 10⁻^4^ mol/L in duplicate), and were then incubated for an additional 72 h at 37 °C in a 5% CO_2_ environment. The working dilutions in DMSO were freshly generated on the day of testing. Following a 72 h incubation period, the growth rate of the cells was evaluated via the MTT assay, which quantifies dehydrogenase activity in viable cells. The MTT cell proliferation assay is a colorimetric assay system that quantifies the conversion of the tetrazolium compound (MTT) into an insoluble formazan product by the mitochondria of viable cells. For this analysis, the medium containing the substance was removed, and 40 µL of MTT reagent was added to each well at a concentration of 0.5 mg/mL. Subsequently, the precipitates were dissolved in 160 µL of DMSO following a four-hour incubation period. The absorbance (A) was determined using a microplate reader set at a wavelength of 595 nm (Hidex Chameleon V, Hidex, Turku, Finland). The percentage of growth (PG) for the cell lines was calculated using one of two subsequent expressions: Each experiment was conducted in quadruplicate across two independent trials.

If (mean A_test_ − mean A_tzero_) ≥ 0, then (1)

PG = 100 × (mean A_test_ − mean A_tzero_)/(mean A_ctrl_ − mean A_tzero_), (2)

and if (mean A_test_ − mean A_tzero_) < 0, then (3)

PG = 100 × (mean A_test_ − mean A_tzero_)/A_tzero_ (4)

where the mean A_tzero_ is the average of the optical density measurements before the exposure of the cells to the test compound, the mean A_test_ is the average of the optical density measurements after the desired period, and the mean A_ctrl_ is the average of the optical density measurements after the desired period with no exposure of the cells to the test compound. Each experimental trial was conducted in quadruplicate across two separate experiments. The results are expressed as IC_50_, which indicates the concentration required for 50% inhibition. The IC_50_ values for each compound are calculated from concentration–response curves using linear regression analysis by fitting the test concentrations that give PG values above and below the reference value (i.e., 50%). However, in instances where all tested concentrations for a specific cell line yield PGs that exceed the corresponding reference effect level (e.g., a PG value of 50), the highest tested concentration is designated as the default value, prefixed by a “>“ symbol.

#### 3.1.9. In Silico Drug-Likeness and Biological Profiling of 3,5-Disubstituted Hydantoins **5a**–**i**

The commercial program ADMET Predictor ver. 9.5 (Simulation Plus Inc., Lancaster, CA, USA) [53] was used to predict various simple structural features, physicochemical properties, and pharmacokinetic- and toxicity-related parameters of novel hydantoin derivatives. These were the topological polar surface area (TPSA) and numbers of hydrogen bond donating (HBD) and accepting (HBA) atoms, water solubility (Sw), lipophilicity coefficient (logP), human effective jejunal permeability (Peff), permeability to Madin-Darby canine kidney cells (MDCK), percent unbound to human blood plasma proteins (hum_fup%), percent unbound in human liver microsomes (fumic%), high/low classification of the probability of passing through the blood–brain barrier (BBB), brain/blood partition coefficient (logBB), likeness of P-glycoprotein efflux (Pgp sub) and inhibition (Pgp inh), rodent chronic toxicity Rat_ and Mouse_TD50 values estimating the oral dose (in mg kg^−1^ per day) of a compound required to induce a tumor in 50% of a rat/mouse population after exposure over a standard lifetime, yes/no likelihood of the hERG potassium channel inhibition (hERG_filter), and potential toxicity liabilities summarizing all ADMET risk models (Tox_Code). The web server admetSAR (https://lmmd.ecust.edu.cn/admetsar2/, accessed on 17 February 2020) was used to predict whether a compound may be an inhibitor (CYPs 3A4, 2D6, 2C19, 2C9, and 1A2) and a substrate (CYPs 3A4, 2D6, and 3A4) of the CYP enzymes [54]. 

Potential biological activities were screened in silico by using the commercial software PASS 2020 [55] and freely available web server SwissTargetPrediction [56]. 

## 4. Conclusions 

In summary, a series of novel drug-like (±)-3,5-disubstituted hydantoins *syn*/*anti*-**5a**–**i** were synthesized by a base-assisted intramolecular amidolysis of *trans*-β-lactams ureas, with yields from good to excellent. The analysis of the NMR spectra revealed significant differences in carbon chemical shifts of two diastereoisomers. In addition, the NOE interactions, coupled with molecular modelling calculations, helped determine the relative configuration of the two diastereoisomers. The absolute configuration of the four enantiomers of allyl hydantoin *syn*/*anti*-**5a** was assigned using TD-DFT calculations of ECD and VCD spectra. The biological potential of novel hydantoins was evaluated in vitro by testing on antiproliferative activities and in silico using the commercial programs PASS and ADMET Predictor and the free online servers SwissTargetPrediction and admetSAR. Most of the tested compounds **5a**–**i** showed moderate antiproliferative activity on the tested tumor cell lines A2780 and MCF7. Four hydantoins, *anti*-**5b**, *syn*-**5f**, *anti*-**5f,** and *anti*-**5g**, showed moderate activity against the liver cancer cell line HepG2. The hydantoin compounds *anti*-**5b**, *anti*-**5c**, *syn*-**5f**, *syn*-**5g**, *anti*-**5g**, *syn*-**5h,** and *anti*-**5h** showed a moderate cytotoxic effect, while the other hydantoins had less or no toxic effect on the HFF-1 healthy cells. Among the synthesized hydantoins, compound *anti*-**5c**, with a cyclopentyl group at the N-3 position of the hydantoin ring, exhibited a strong cytotoxic effect against human tumor cell line MCF7 (IC_50_ = 4.5 µmol/L) and non-tumor cell line HFF-1 (IC_50_ = 12.0 µmol/L). In silico ADMET profiling describes hydantoins **5a**–**i** as aqueous soluble and membrane permeable molecules that tend to be metabolized by CYP3A4 and eliminated by the Pgp pump. According to PASS, synthesized hydantoins **5a**–**i** have a high probability of antiarthritic activity (Pa ≥ 0.727). The hydantoin *syn*/*anti*-**5d**, with a furfuryl group at the N-3 position of the hydantoin ring, is also predicted to be an inhibitor of thioredoxin glutathione reductase, beta lactamase, DNA-directed DNA polymerase, and DNA polymerase beta. The SwissTargetPrediction tool did not predict significant biological targets for compounds **5a**–**i**, consistent with the novelty of the structure of the synthesized compounds.

## Figures and Tables

**Table 1:** Structures, yields, and diastereomeric ratio of 3,5-disubstituted hydantoins **5a**–**i.**

Entry	β*-*Lactam Urea	Hydantoin	R	η (%)	HPLC *Syn*:*Anti*
1	**4a**	**5a**	allyl	85.8	52.5:47.5
2	**4b**	**5b**	hexyl	91.8	50.1:49.9
3	**4c**	**5c**	cylopentyl	79.5	51.8:48.2
4	**4d**	**5d**	furfuryl	81.5	57.1:42.9
5	**4e**	**5e**	benzyl	86.4	52.2:47.8
6	**4f**	**5f**	4-*tert*-butylphenyl	88.8	66.0:34.0
7	**4g**	**5g**	3-chloro-4-methylphenyl	75.3	48.3:51.7
8	**4h**	**5h**	3,5-dimethylphenyl	58.8	64.3:35.7
9	**4i**	**5i**	2,6-dimethylphenyl	75.8	58.8:41.2

**Table 2:** Antiproliferative activities of hydantoins *syn*-**5a**–**i** and *anti*-**5a**–**i** in vitro (IC_50_, μmol/L).

Compound	HepG2 *	A2780 *	MCF7 *	HFF-1 *
*syn* **-5a**	>100	71 ± 34	>100	>100
*anti* **-5a**	>100	>100	>100	>100
*syn* **-5b**	>100	93 ± 123	55 ± 1.8	>100
*anti* **-5b**	35 ± 12	33 ± 12	34 ± 9	52 ± 15
*syn* **-5c**	>100	35 ± 7.9	41 ± 5.4	>100
*anti* **-5c**	>100	42 ± 57	4.5 ± 0.45	12 ± 13
*syn* **-5d**	>100	>100	>100	>100
*anti* **-5d**	>100	79 ± 5.4	>100	>100
*syn* **-5e**	>100	59 ± 17	>100	>100
*anti* **-5e**	>100	58 ± 7.7	55 ± 2.4	>100
*syn* **-5f**	15 ± 1.2	15 ± 1.7	20 ± 0.15	20 ± 0.48
*anti* **-5f**	33 ± 1.4	22 ± 5.9	39 ± 9.5	>100
*syn* **-5g**	>100	28 ± 13	21 ± 0.35	33 ± 18
*anti* **-5g**	30 ± 3.0	43 ± 13	27 ± 1.4	35 ± 55
*syn* **-5h**	>100	43 ± 4.0	27 ± 2.2	21 ± 49
*anti* **-5h**	>100	59 ± 2.4	48 ± 14	18 ± 27
*syn* **-5i**	>100	77 ± 3.0	87 ± 32	>100
*anti* **-5i**	>100	48 ± 10	52 ± 21	94 ± 11

* The human cancer cell lines used in this study were HepG2 (derived from liver), A2780 (derived from ovarian tissue), and MCF-7 (derived from breast tissue). The normal human cell line used was HFF1 (human fibroblasts). Cell proliferation was assessed after 72 h using the MTT assay, as delineated in the Materials and Methods section.

## Data Availability

Data is contained within the article and Supplementary Materials.

## Supplementary Materials

The following supporting information can be downloaded at: https://www.mdpi.com/article/10.3390/ph17101259/s1, Figure S1: ^1^H NMR (300 MHz; CDCl_3_) spectra of compound **1**; Figure S2: ^13^C NMR (75 MHz; CDCl_3_) spectra of compound **1**; Figure S3: ^1^H NMR (300 MHz, CDCl_3_) spectra of β-lactam protons H-3 and H-4 of the *cis*/*trans*-3-phthalimido-β-lactam mixture **2a**/**2b**; Figure S4: ^1^H NMR (600 MHz; CDCl_3_) spectra of compound **2a**; Figure S5: ^13^C NMR (151 MHz; CDCl_3_) spectra of compound **2a**; Figure S6: ^1^H NMR (600 MHz; CDCl_3_) spectra of compound **2b**; Figure S7: ^13^C NMR (151 MHz; CDCl_3_) spectra of compound **2b**; Figure S8: ^1^H NMR (600 MHz; CDCl_3_) spectra of compound **3**; Figure S9: ^13^C NMR (151 MHz; CDCl_3_) spectra of compound **3**; Figure S10: ^1^H NMR (600 MHz; CDCl_3_) spectra of compound **4a**; Figure S11: ^13^C NMR (151 MHz; CDCl_3_) spectra of compound **4a**; Figure S12: ^1^H NMR (600 MHz; CDCl_3_) spectra of compound **4b**; Figure S13: ^13^C NMR (151 MHz; CDCl_3_) spectra of compound **4b**; Figure S14: ^1^H NMR (600 MHz; CDCl_3_) spectra of compound **4c**; Figure S15: ^13^C NMR (151 MHz; CDCl_3_) spectra of compound **4c**; Figure S16: ^1^H NMR (600 MHz; CDCl_3_) spectra of compound **4d**; Figure S17: ^13^C NMR (151 MHz; CDCl_3_) spectra of compound **4d**; Figure S18: ^1^H NMR (600 MHz; CDCl_3_) spectra of compound **4e**; Figure S19: ^13^C NMR (151 MHz; CDCl_3_) spectra of compound **4e**; Figure S20**:** ^1^H NMR (600 MHz; CDCl_3_) spectra of compound **4f**; Figure S21: ^13^C NMR (151 MHz; CDCl_3_) spectra of compound **4f**; Figure S22: ^1^H NMR (600 MHz; CDCl_3_) spectra of compound **4g**; Figure S23: ^13^C NMR (151 MHz; CDCl_3_) spectra of compound **4g**; Figure S24: ^1^H NMR (600 MHz; CDCl_3_) spectra of compound **4h**; Figure S25: ^13^C NMR (151 MHz; CDCl_3_) spectra of compound **4h**; Figure S26: ^1^H NMR (600 MHz; CDCl_3_) spectra of compound **4i**; Figure S27: ^13^C NMR (151 MHz; CDCl_3_) spectra of compound **4i**; Figure S28: ^1^H NMR (600 MHz; CD_3_CN) spectra of compound **5a**; Figure S29: ^13^C NMR (151 MHz; CD_3_CN) spectra of compound **5a**; Figure S30: ^1^H NMR (600 MHz; CD_3_CN) spectra of compound **5b**; Figure S31: ^13^C NMR (151 MHz; CD_3_CN) spectra of compound **5b**; Figure S32: ^1^H NMR (600 MHz; CD_3_CN) spectra of compound **5c**; Figure S33: ^13^C NMR (151 MHz; CD_3_CN) spectra of compound **5c**; Figure S34: ^1^H NMR (600 MHz; CD_3_CN) spectra of compound **5d**; Figure S35: ^13^C NMR (151 MHz; CD_3_CN) spectra of compound **5d**; Figure S36: ^1^H NMR (600 MHz; CD_3_CN) spectra of compound **5e**; Figure S37: ^13^C NMR (151 MHz; CD_3_CN) spectra of compound **5e**; Figure S38: ^1^H NMR (600 MHz; CD_3_CN) spectra of compound **5f**; Figure S39: ^13^C NMR (151 MHz; CD_3_CN) spectra of compound **5f**; Figure S40: ^1^H NMR (600 MHz; CD_3_CN) spectra of compound **5g**; Figure S41: ^13^C NMR (151 MHz; CD_3_CN) spectra of compound **5g**; Figure S42: ^1^H NMR (600 MHz; CD_3_CN) spectra of compound **5h**; Figure S43: ^13^C NMR (151 MHz; CD_3_CN) spectra of compound **5h**; Figure S44: ^1^H NMR (600 MHz; CD_3_CN) spectra of compound **5i**; Figure S45: ^13^C NMR (151 MHz; CD_3_CN) spectra of compound **5i**; **Figure S46**: ^1^H spectrum, structure, numbering, and full assignment of Peak 1 in acetonitrile-d_3_ at 25 °C; **Figure S47**: ^13^C spectrum, structure, numbering, and full assignment of Peak 1 in acetonitrile-d_3_ at 25 °C; Figure S48: Fully assigned ^1^H-^1^H COSY spectra of Peak 1 in acetonitrile-d_3_ at 25 °C; Figure S49: Fully assigned ^1^H-^13^C HSQC spectrum of Peak 1 in acetonitrile-d_3_ at 25 °C; Figure S50: Fully assigned ^1^H-^13^C HMBC spectrum of Peak 1 in acetonitrile-d_3_ at 25 °C; Figure S51: Fully assigned ^1^H-^1^H NOESY spectrum of Peak 1 in acetonitrile-d_3_ at 25 °C; Figure S52: ^1^H spectrum, structure, numbering, and full assignment of Peak 2 in acetonitrile-d_3_ at 25 °C; Figure S53: ^13^C spectrum, structure, numbering, and full assignment of Peak 2 in acetonitrile-d_3_ at 25 °C; Figure S54: Fully assigned ^1^H-^1^H COSY spectrum of Peak 2 in acetonitrile-d_3_ at 25 °C; Figure S55: Fully assigned ^1^H-^13^C HSQC spectrum of Peak 2 in acetonitrile-d_3_ at 25 °C; Figure S56: Fully assigned ^1^H-^13^C HMBC spectrum of Peak 2 in acetonitrile-d_3_ at 25 °C; Figure S57: Fully assigned ^1^H-^1^H NOESY spectrum of Peak 2 in acetonitrile-d_3_ at 25 °C; Figure S58: RP-HPLC chromatogram of compound **2a**. Figure S59: RP-HPLC chromatogram of compound **2b**; Figure S60: RP-HPLC chromatogram of compound **3**; Figure S61: RP-HPLC chromatogram of compound **4a**; Figure S62: RP-HPLC chromatogram of compound **4b**; Figure S63: RP-HPLC chromatogram of compound **4c**; Figure S64: RP-HPLC chromatogram of compound **4d**; Figure S65: RP-HPLC chromatogram of compound **4e**; Figure S66: RP-HPLC chromatogram of compound **4f**; Figure S67: RP-HPLC chromatogram of compound **4g**; Figure S68: RP-HPLC chromatogram of compound **4h**; Figure S69: RP-HPLC chromatogram of compound **4i**; Figure S70: RP-HPLC chromatogram of compound *syn*/*anti*-**5a**; Figure S71: RP-HPLC chromatogram of compound *anti*-**5a**; Figure S72: RP-HPLC chromatogram of compound *syn*-**5a**; Figure S73: RP-HPLC chromatogram of compound *syn*/*anti*-**5b**; Figure S74: RP-HPLC chromatogram of compound *anti*-**5b**; Figure S75: RP-HPLC chromatogram of compound *syn*-**5b**; Figure S76: RP-HPLC chromatogram of compound *syn*/*anti*-**5c**, Figure S77: RP-HPLC chromatogram of compound *anti*-**5c**; Figure S78: RP-HPLC chromatogram of compound *syn*-**5c**; Figure S79: RP-HPLC chromatogram of compound *syn*/*anti*-**5d**; Figure S80: RP-HPLC chromatogram of compound *anti*-**5d**; Figure S81: RP-HPLC chromatogram of compound *syn*-**5d**; Figure S82: RP-HPLC chromatogram of compound *syn*/*anti*-**5e**; Figure S83: RP-HPLC chromatogram of compound *anti*-**5e**; Figure S84: RP-HPLC chromatogram of compound *syn*-**5e**; Figure S85: RP-HPLC chromatogram of compound *syn*/*anti*-**5f**; Figure S86: RP-HPLC chromatogram of compound *anti*-**5f**; Figure S87: RP-HPLC chromatogram of compound *syn*-**5f**; Figure S88: RP-HPLC chromatogram of compound *syn*/*anti*-**5g**; Figure S89: RP-HPLC chromatogram of compound *anti*-**5g**; Figure S90: RP-HPLC chromatogram of compound *syn*-**5g**; Figure S91: RP-HPLC chromatogram of compound *syn*/*anti*-**5h**; Figure S92: RP-HPLC chromatogram of compound *anti*-**5h**; Figure S93: RP-HPLC chromatogram of compound *syn*-**5h**; Figure S94: RP-HPLC chromatogram of compound *syn*/*anti*-**5i**; Figure S95: RP-HPLC chromatogram of compound *anti*-**5i**; Figure S96: RP-HPLC chromatogram of compound *syn*-**5i**; Figure S97: Chromatogram of *syn*/*anti*-**5a** on preparative Zorbax Extend C-18 PrepHT preparative column; Figure S98: Chromatogram of *anti*-**5a** (Peak 1) on analytical CHIRAL ART Amylose SA column; Figure S99: Chromatogram of *syn*-**5a** (Peak 2) on analytical CHIRAL ART Amylose SA column; Figure S100: Chromatogram of enantiomer **5a**-**ent1** (*anti*-**5a**, Peak 1); Figure S101: Chromatogram of **5a**-**ent2** (*anti*-**5a**, Peak 1); Figure S102: Chromatogram of **5a**-**ent3** (*syn*-**5a**, Peak 2). Figure S103: Chromatogram of **5a**-**ent4** (*syn*-**5a**, Peak 2); Figure S104: Chromatogram of *anti*-**5a** (Peak 1) on the semi-preparative CHIRAL ART Amylose SA column; Figure S105: Chromatogram of *syn*-**5a** (Peak 2) on the semi-preparative CHIRAL ART Amylose SA column; Figure S106: HR-MS (ESI-QTOF) spectra of compound **2a**; Figure S107: HR-MS (ESI-QTOF) spectra of compound **2b**; Figure S108: HR-MS (ESI-QTOF) spectra of compound **4a**; Figure S109: HR-MS (ESI-QTOF) spectra of compound **4b**; Figure S110: HR-MS (ESI-QTOF) spectra of compound **4c**; Figure S111: HR-MS (ESI-QTOF) spectra of compound **4d**; Figure S112: HR-MS (ESI-QTOF) spectra of compound **4e**; Figure S113: HR-MS (ESI-QTOF) spectra of compound **4f**; Figure S114: HR-MS (ESI-QTOF) spectra of compound **4g**; Figure S115: HR-MS (ESI-QTOF) spectra of compound **4h**; Figure S116: HR-MS (ESI-QTOF) spectra of compound **4i**; Figure S117: HR-MS (ESI-QTOF) spectra of compound *syn*/*anti*-**5a**; Figure S118: HR-MS (ESI-QTOF) spectra of compound *syn*/*anti*-**5b**; Figure S119: HR-MS (ESI-QTOF) spectra of compound *syn*/*anti*-**5c**; Figure S120: HR-MS (ESI-QTOF) spectra of compound *syn*/*anti*-**5d**; Figure S121: HR-MS (ESI-QTOF) spectra of compound *syn*/*anti*-**5e**; Figure S122: HR-MS (ESI-QTOF) spectra of compound *syn*/*anti*-**5f**; Figure S123: HR-MS (ESI-QTOF) spectra of compound *syn*/*anti*-**5g**; Figure S124: HR-MS (ESI-QTOF) spectra of compound *syn*/*anti*-**5h**; Figure S125: HR-MS (ESI-QTOF) spectra of compound *syn*/*anti*-**5i**; Figure S126: IR spectra of compound **2a**; Figure S127: IR spectra of compound **2b**; Figure S128: IR spectra of compound **3**; Figure S129: IR spectra of compound **4a**; Figure S130: IR spectra of compound **4b**; Figure S131: IR spectra of compound **4c**; Figure S132: IR spectra of compound **4d**; Figure S133: IR spectra of compound **4e**; Figure S134: IR spectra of compound **4f**; Figure S135: IR spectra of compound **4g**; Figure S136: IR spectra of compound **4h**; Figure S137: IR spectra of compound **4i**; Figure S138: IR spectra of compound *syn*/*anti*-**5a**; Figure S139: IR spectra of compound *syn*/*anti*-**5b**; Figure S140: IR spectra of compound *syn*/*anti*-**5c**; Figure S141: IR spectra of compound *syn*/*anti*-**5d**; Figure S142: IR spectra of compound *syn*/*anti*-**5e**; Figure S143: IR spectra of compound *syn*/*anti*-**5f**; Figure S144: IR spectra of compound *syn*/*anti*-**5g**; Figure S145: IR spectra of compound *syn*/*anti*-**5h**; Figure S146: IR spectra of compound *syn*/*anti*-**5i**; Figure S147: ECD spectra calculated for the truncated analogs of (5*S*,6*S*)-**5a** and (5*R*,6*S*)-**5a** (with the allyl group replaced by a methyl) at the TD-B3LYP/def2-TZVP/PCM//B3LYP-D3BJ/6-311 + G(d,p) level. Plotting parameters: UV shift + 15 nm, σ = 0.3 eV; Table S1: Proton chemical shifts comparison for Peak 1 and Peak 2 in acetonitrile-d_3_ at 25 °C, with major differences marked in red; Table S2: Carbon chemical shifts comparison for Peak 1 and Peak 2 in acetonitrile-d_3_ at 25 °C, with major differences marked in red; Table S3: Comparison of NOE interactions for Peak 1 in acetonitrile-d_3_ at 25 °C, with major differences marked in red, where s = strong, m = medium, and w = weak; Table S4: Comparison of coupling constants for Peak 1 and Peak 2 in acetonitrile-d_3_ at 25 °C, with major differences marked in red; Table S5: Structures, relative energy (kcal/mol), and Boltzmann populations of low-energy minima calculated for (5*S*,6*S*)-**5a** at the B3LYP-D3BJ/6-311+G(d,p)/PCM level; Table S6: Structures, relative energy (kcal/mol), and Boltzmann populations of low-energy minima calculated for (5*R*,6*S*)-**5a** at the B3LYP-D3BJ/6-311+G(d,p)/PCM level; Table S7: ECD similarity factors calculated for the range 190–290 nm, and VCD similarity factors calculated for the range 1500–1200 cm^−1^; Table S8: Structures, relative energy (kcal/mol), and Boltzmann populations of low-energy minima calculated for the truncated model of (5*S*,6*S*)-**5a** at the B3LYP-D3BJ/6-311+G(d,p)/PCM level; Table S9: Structures, relative energy (kcal/mol), and Boltzmann populations of low-energy minima calculated for the truncated model of (5*R*,6*S*)-**5a** at the B3LYP-D3BJ/6-311+G(d,p)/PCM level; Table S10: Prediction of Lipinski’s rule of five properties for the hydantoins **5a**–**i**; Table S11: ADMET properties of the hydantoins **5a**–**i** calculated by ADMET Predictor and admetSAR.
