# Supplementary material for: Synthesis, Absolute Configuration, Biological Profile and Antiproliferative Activity of New 3,5-Disubstituted Hydantoins
Source: Pharmaceuticals (Basel). 2024 Sep 24;17(10):1259. doi: 10.3390/ph17101259 (PMC11510626; doi:10.3390/ph17101259)
Supplement: Supplementary file 1 [file pharmaceuticals-17-01259-s001.zip › pharmaceuticals-3185561-supplementary.pdf]

Supplementary Materials

Synthesis, Absolute Configuration, Biological Profile and Antiproliferative Activity of New 3,5-Disubstituted Hydantoins

Mladenka Jurin[1], Ana Čikoš[2], Višnja Stepanić[3], Marcin Górecki[4], Gennaro Pescitelli[4,*], Darko Kontrec[1], Andreja Jakas[1], Tonko Dražić[6] and Marin Roje[1,*]

[1]Laboratory for Chiral Technologies, Division of Organic Chemistry and Biochemistry, Ruđer Bošković Institute, Bijenička cesta 54, 10000 Zagreb, Croatia

[2]NMR Centre, Ruđer Bošković Institute, Bijenička cesta 54, 10000 Zagreb, Croatia

[3]Laboratory for Machine Learning and Knowledge Representation, Division of Electronics, Ruđer Bošković Institute, Bijenička cesta 54, 10000 Zagreb, Croatia

[4]Institute of Organic Chemistry, Polish Academy of Sciences, 01-224 Warsaw, Poland

[5]Department of Chemistry and Industrial Chemistry, University of Pisa, Via Giuseppe Moruzzi 13, 56124 Pisa, Italy

[6]Laboratory for Biocolloids and Surface Chemistry, Division of Physical Chemistry, Ruđer Bošković Institute, Bijenička cesta 54, 10000 Zagreb, Croatia

*** Correspondence:**
Gennaro Pescitelli
gennaro.pescitelli@unipi.it

Marin Roje
marin.roje@irb.hr

Table of contents

1. ^{1}H and ^{13}C NMR spectra S1
2. NMR Analysis of allyl hydantoin *syn*-5a and *anti*-5a S24
3. HPLC chromatograms S38
4. HPLC enantioseparation of allyl hydantoin *syn*-5a and *anti*-5a S62
5. HRMS spectra S67
6. IR spectra S75
7. DFT and TD-DFT calculations S82
8. *In silico* physicochemical and biological profiling S86
9. References S88

1. ^{1}H and ^{13}C NMR spectra

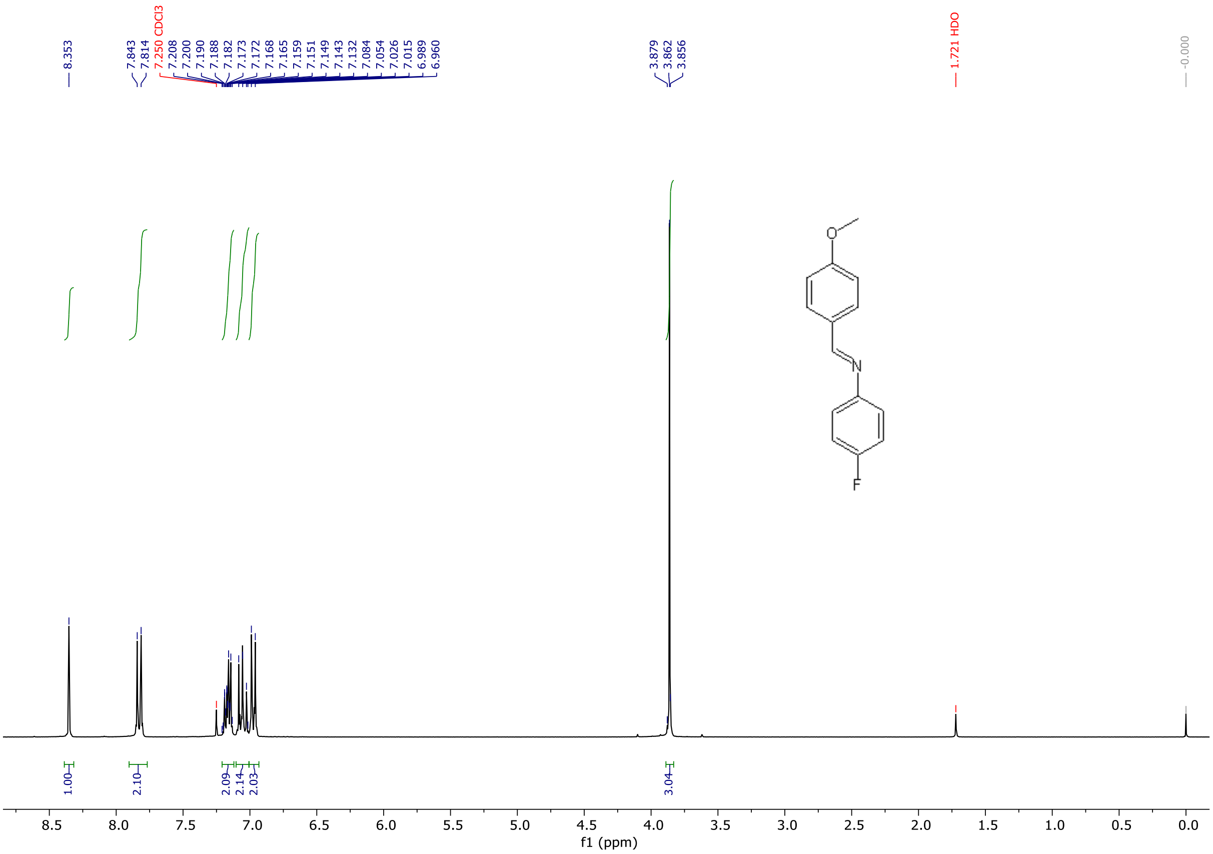

Figure S1. ^{1}H NMR (300 MHz; $CDCl_3$) spectra of compound **1**.

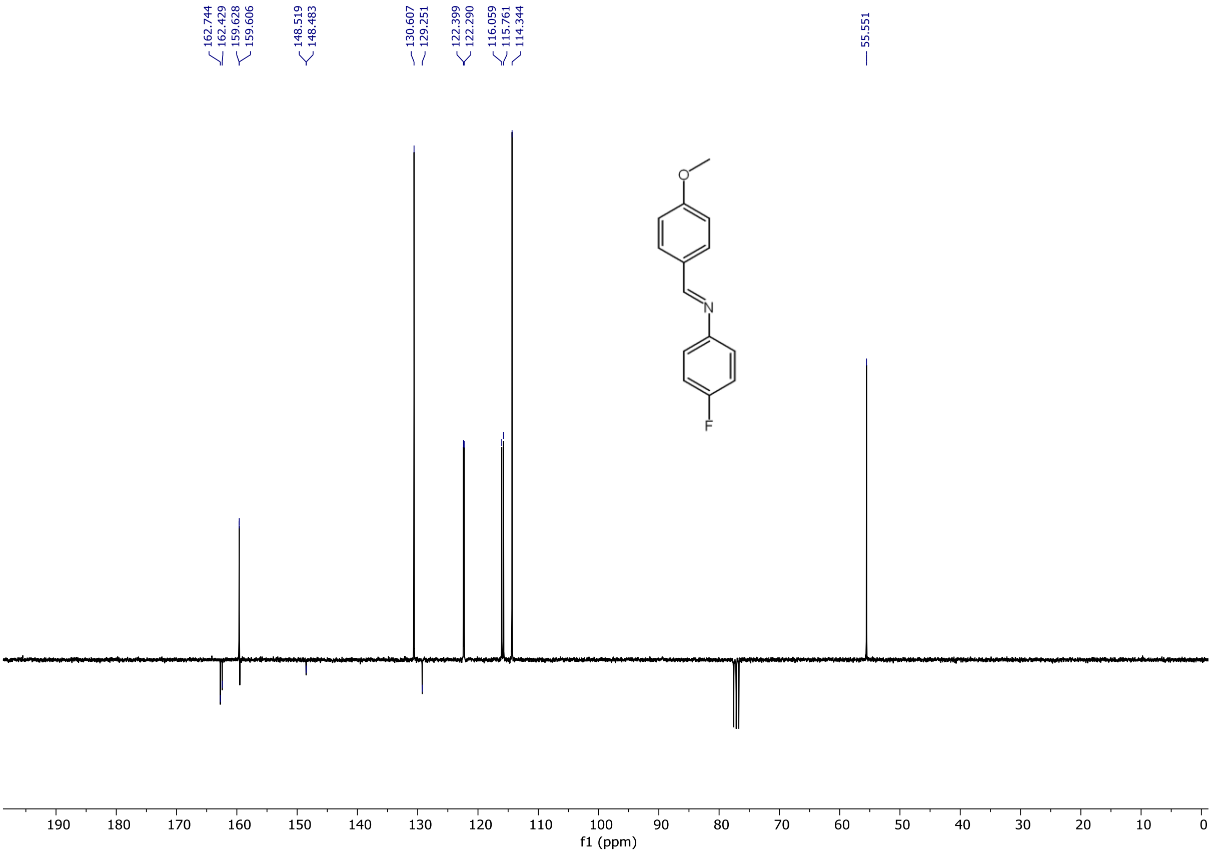

Figure S2. ^{13}C NMR (75 MHz; $CDCl_3$) spectra of compound **1**.

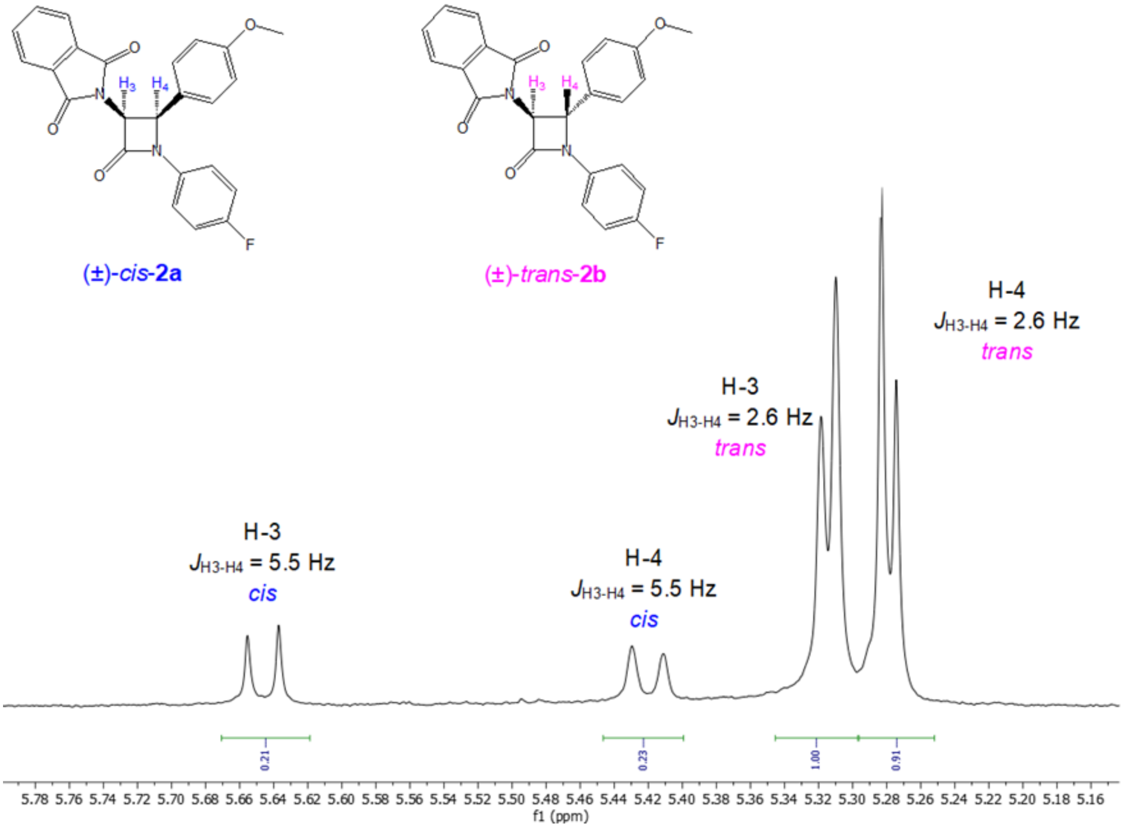

Figure S3. ^{1}H NMR (300 MHz, $CDCl_3$) spectra of β-lactam protons H-3 and H-4 of the *cis*/*trans*-3-phthalimido-β-lactam mixture **2a**/**2b**.

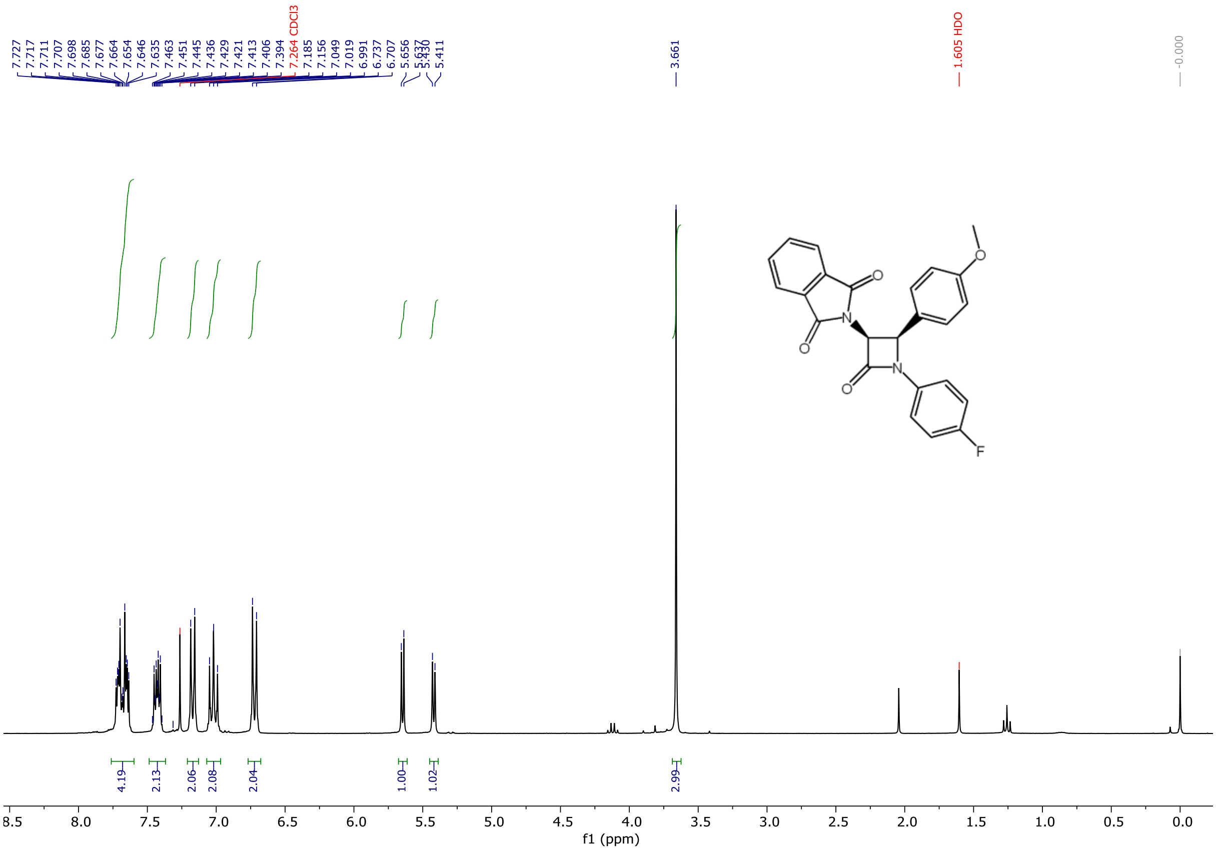

Figure S4. ^{1}H NMR (600 MHz; $CDCl_3$) spectra of compound **2a**.

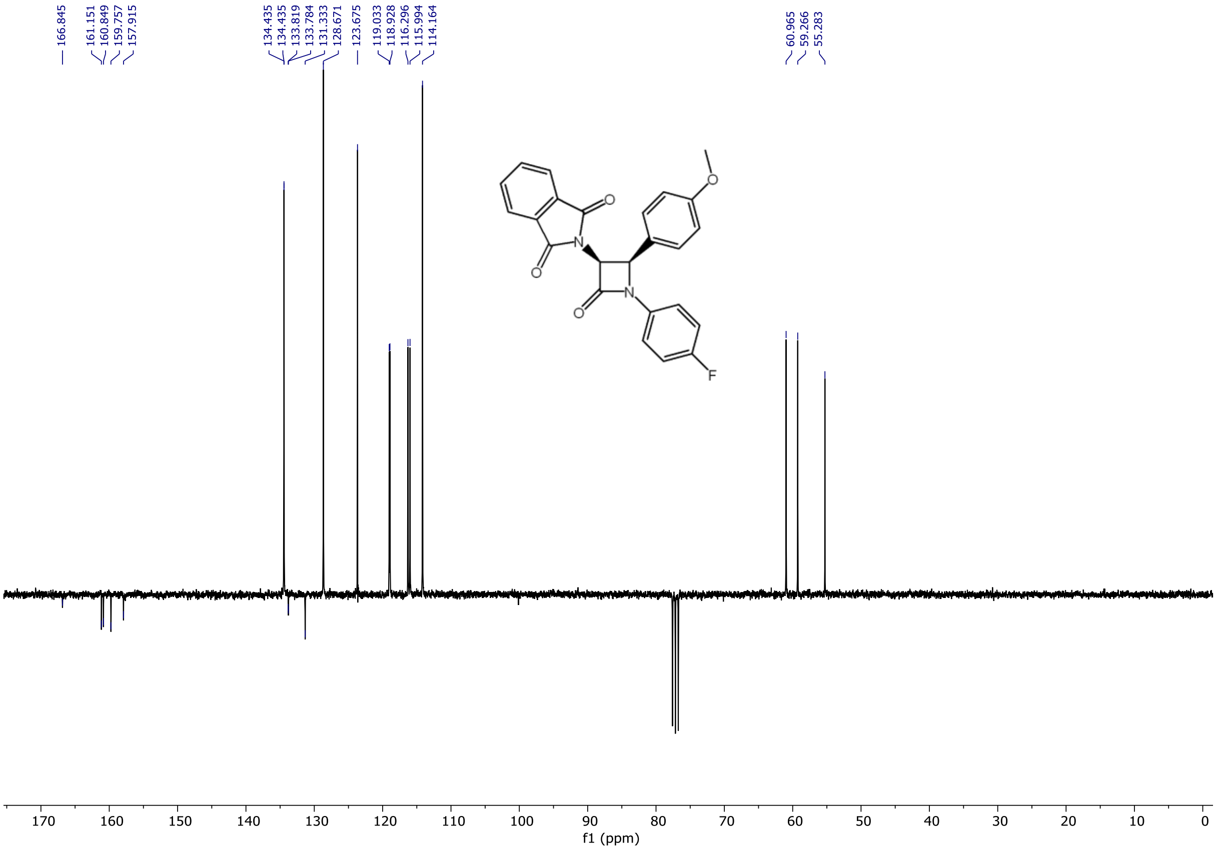

Figure S5. ^{13}C NMR (151 MHz; $CDCl_3$) spectra of compound **2a**.

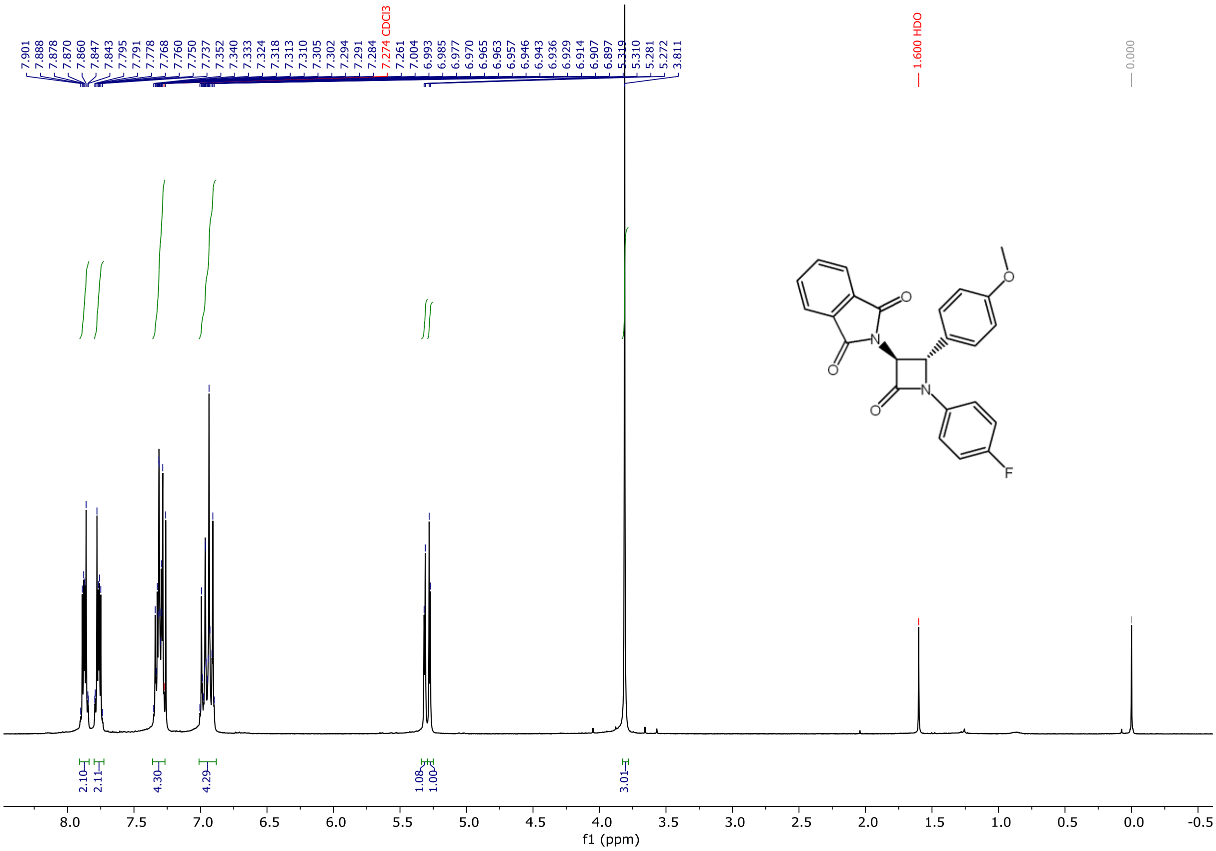

Figure S6. ^{1}H NMR (600 MHz; $CDCl_3$) spectra of compound **2b**.

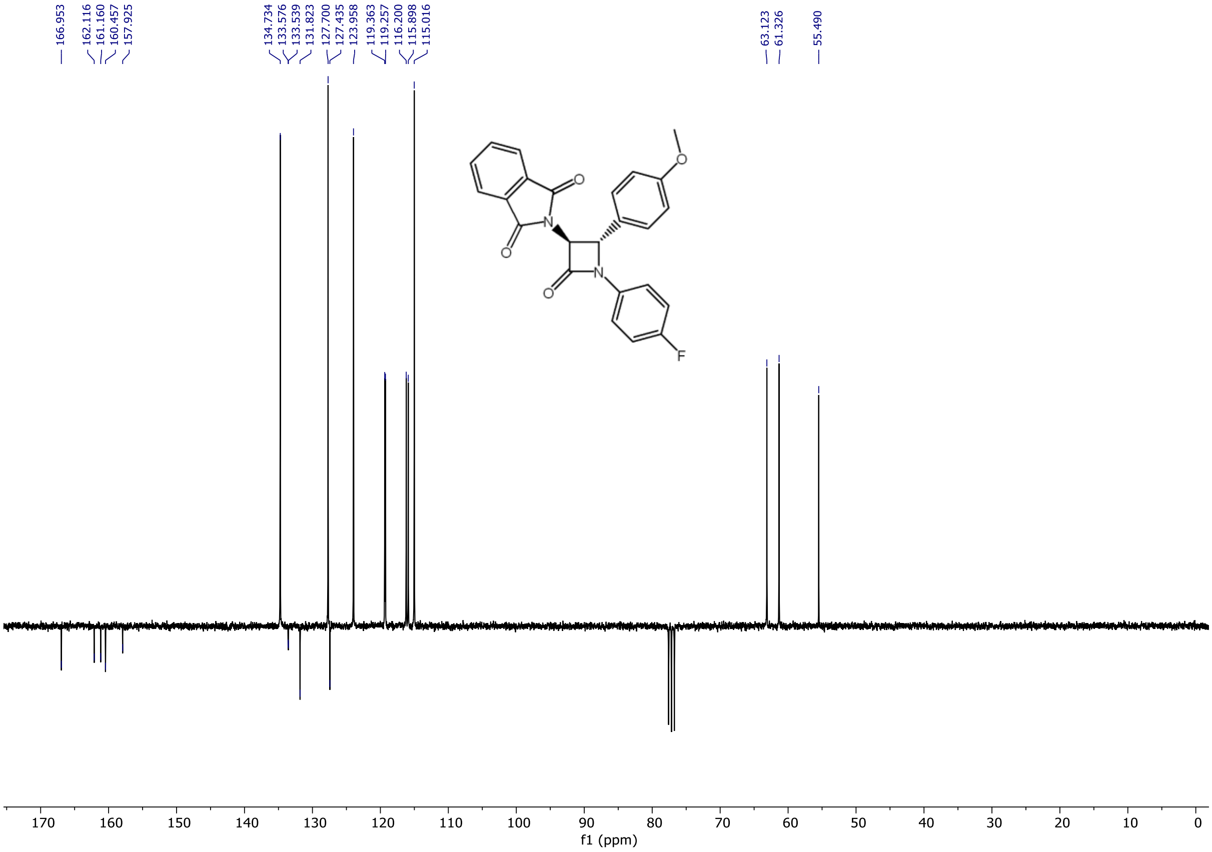

Figure S7. ^{13}C NMR (151 MHz; $CDCl_3$) spectra of compound **2b**.

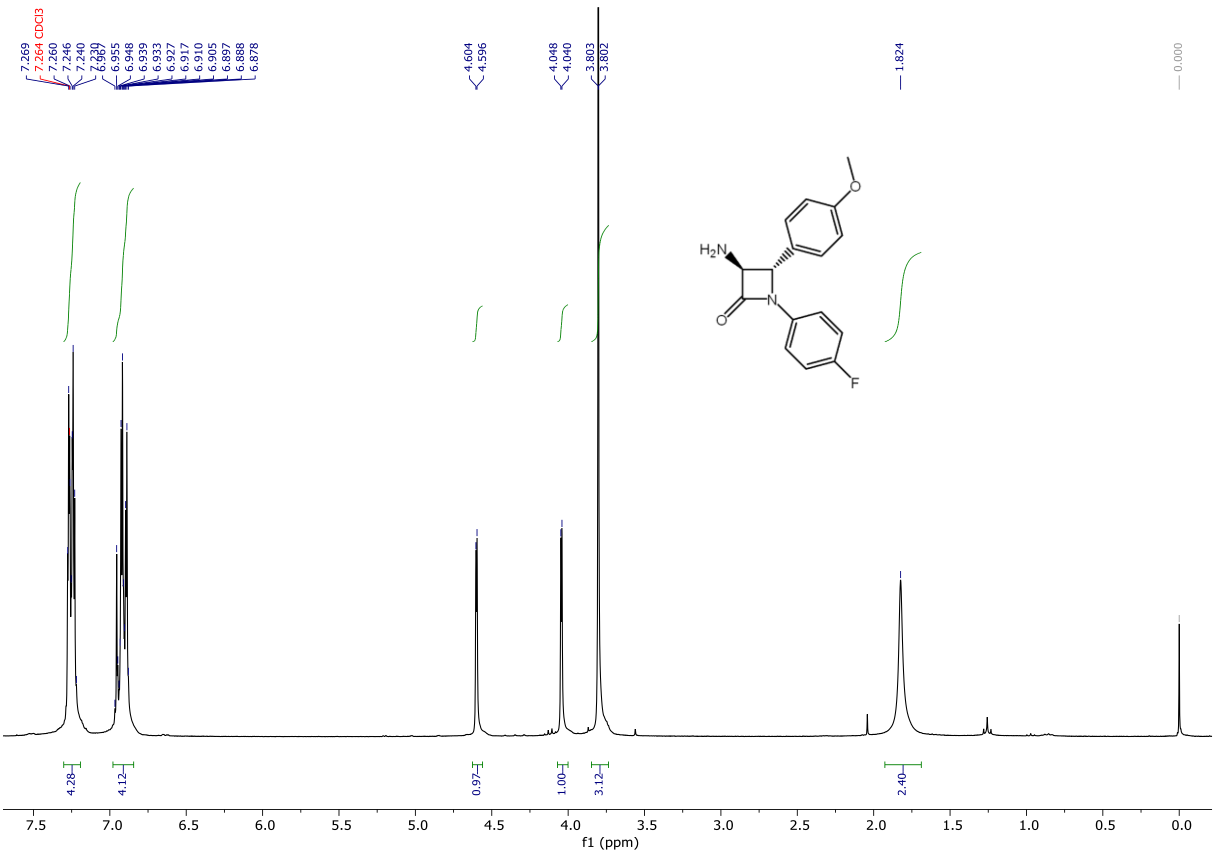

Figure S8. ^{1}H NMR (600 MHz; $CDCl_3$) spectra of compound **3**.

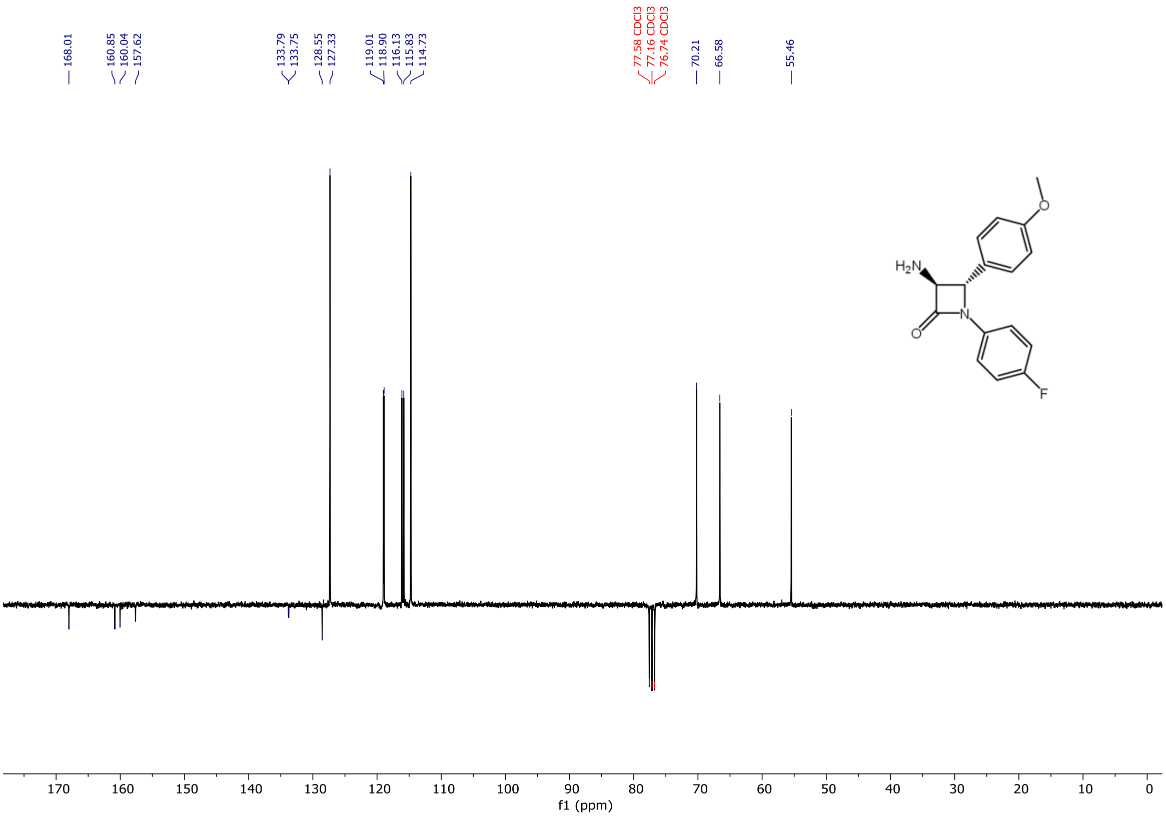

Figure S9. ^{13}C NMR (151 MHz; $CDCl_3$) spectra of compound **3**.

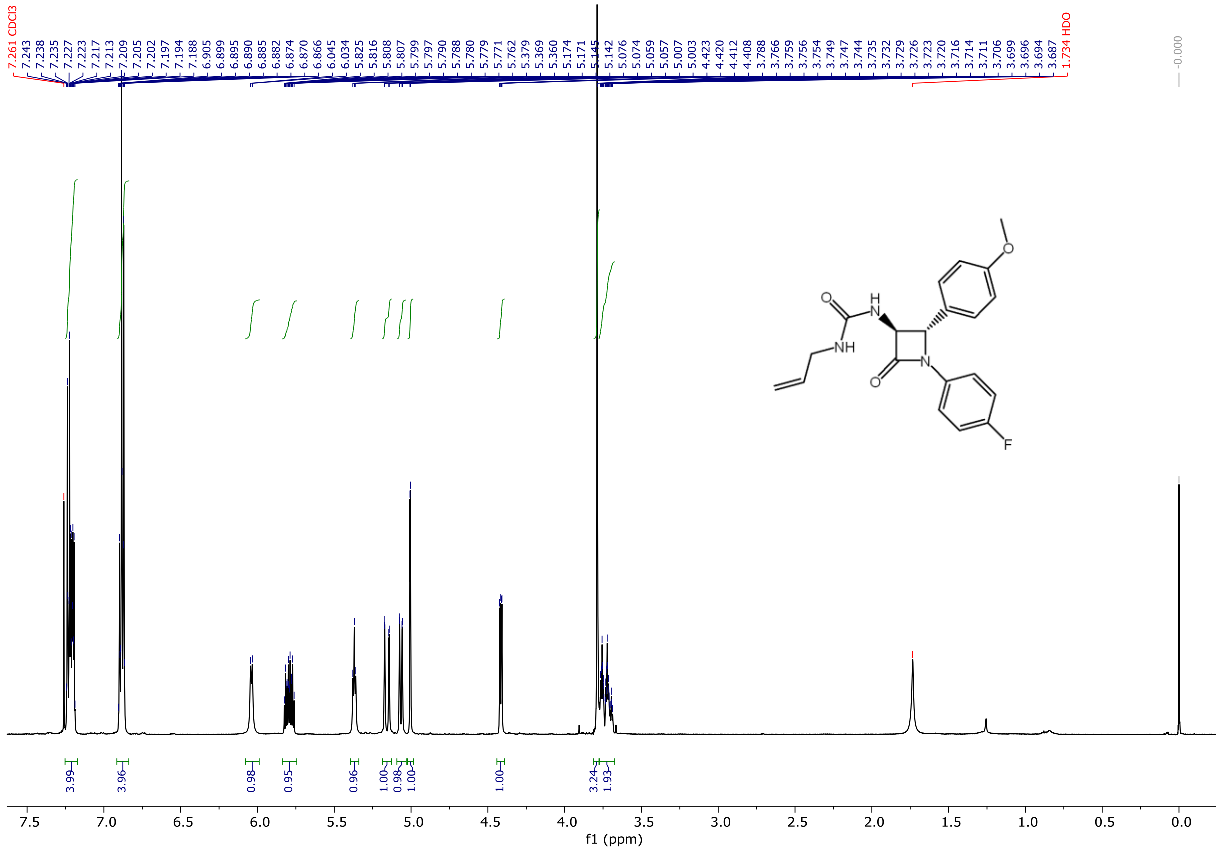

Figure S10. ^{1}H NMR (600 MHz; $CDCl_3$) spectra of compound **4a**.

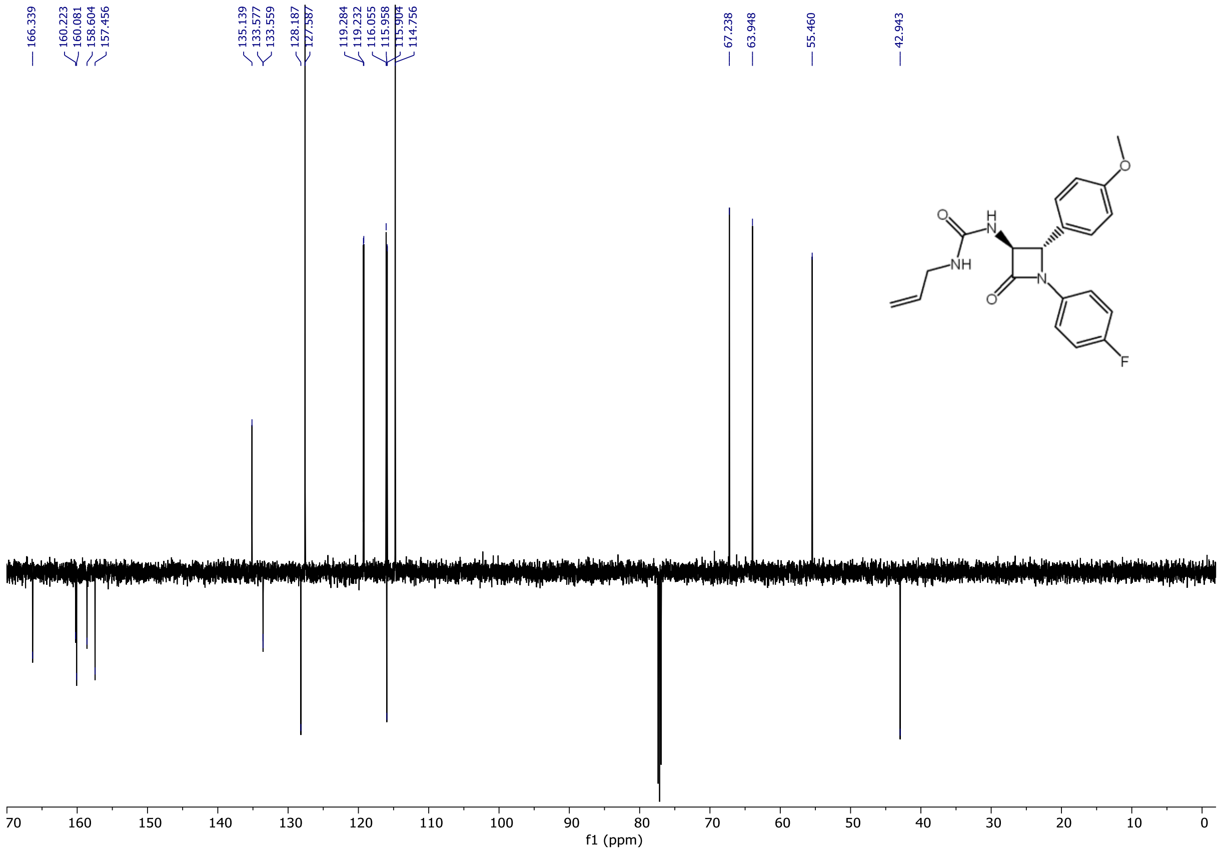

Figure S11. ^{13}C NMR (151 MHz; $CDCl_3$) spectra of compound **4a**.

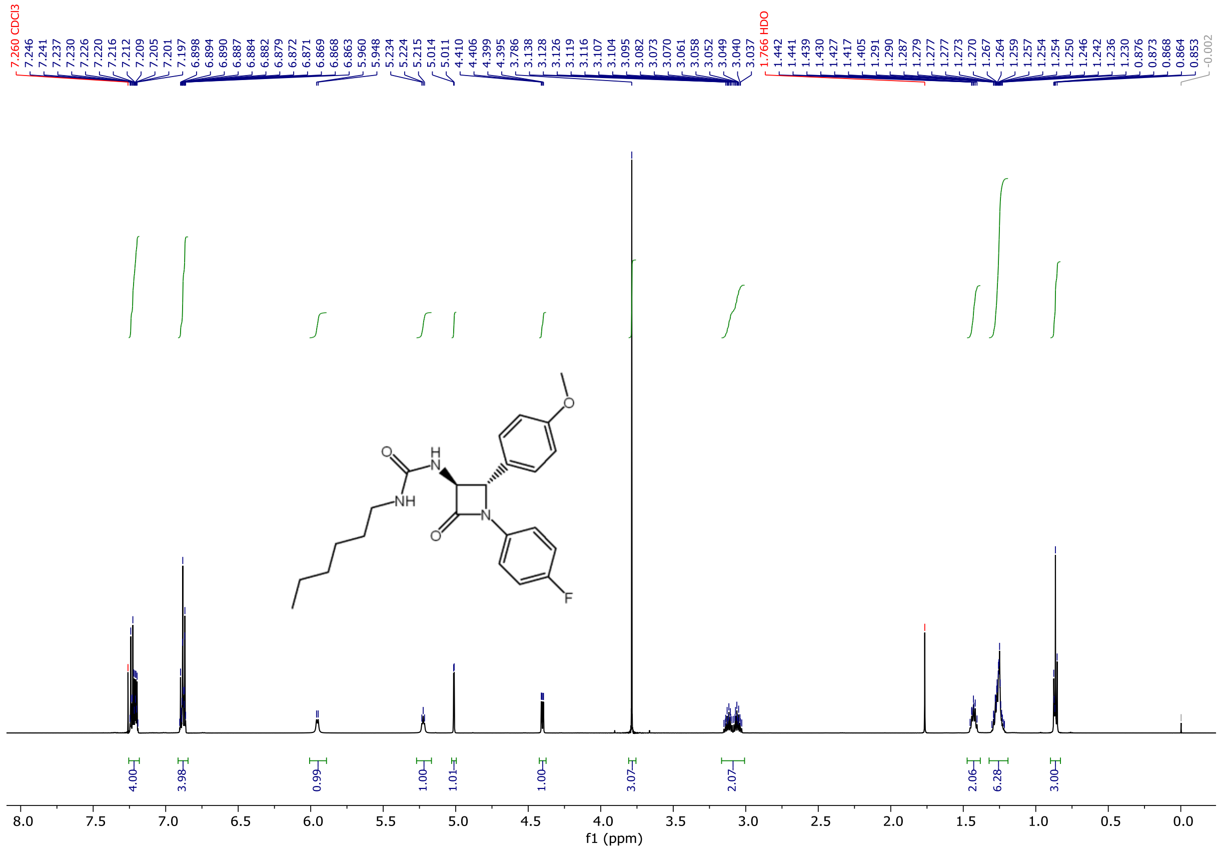

Figure S12. ^{1}H NMR (600 MHz; $CDCl_3$) spectra of compound **4b**.

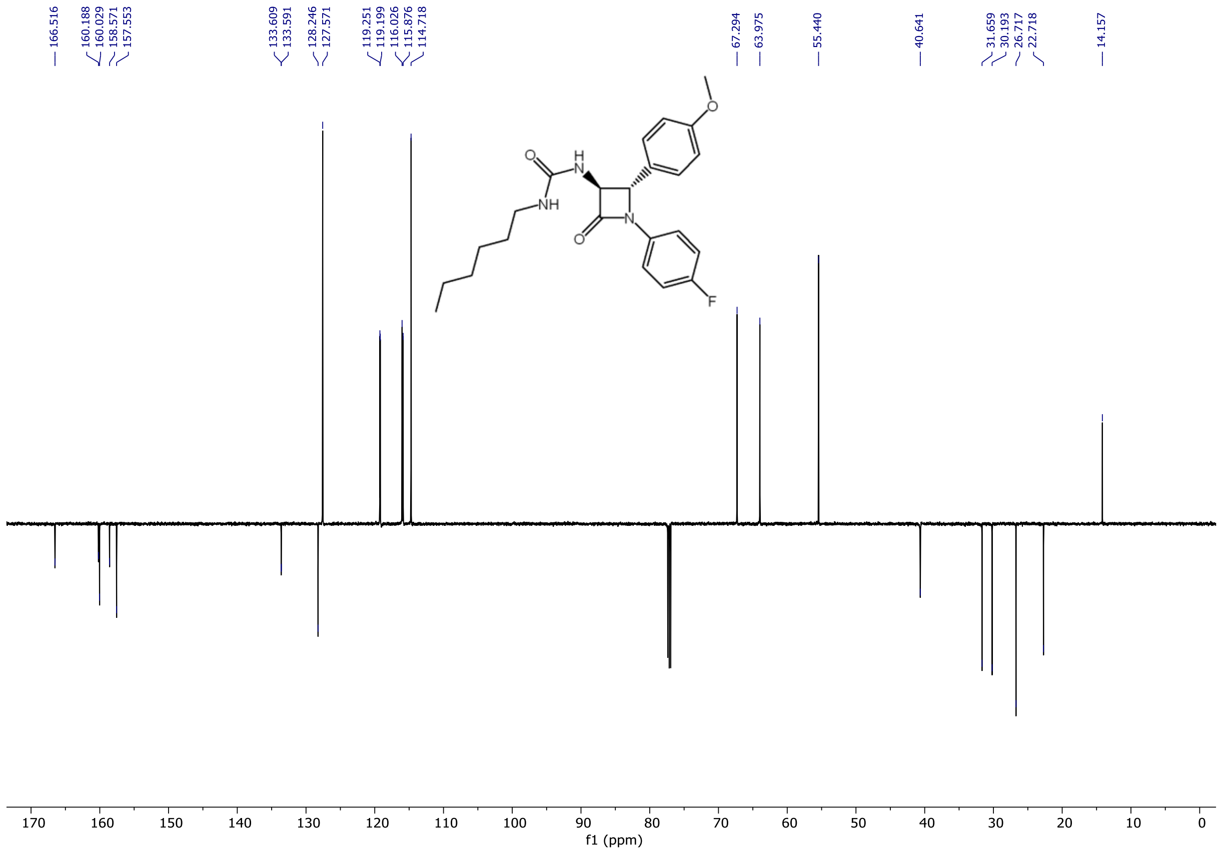

Figure S13. ^{13}C NMR (151 MHz; $CDCl_3$) spectra of compound **4b**.

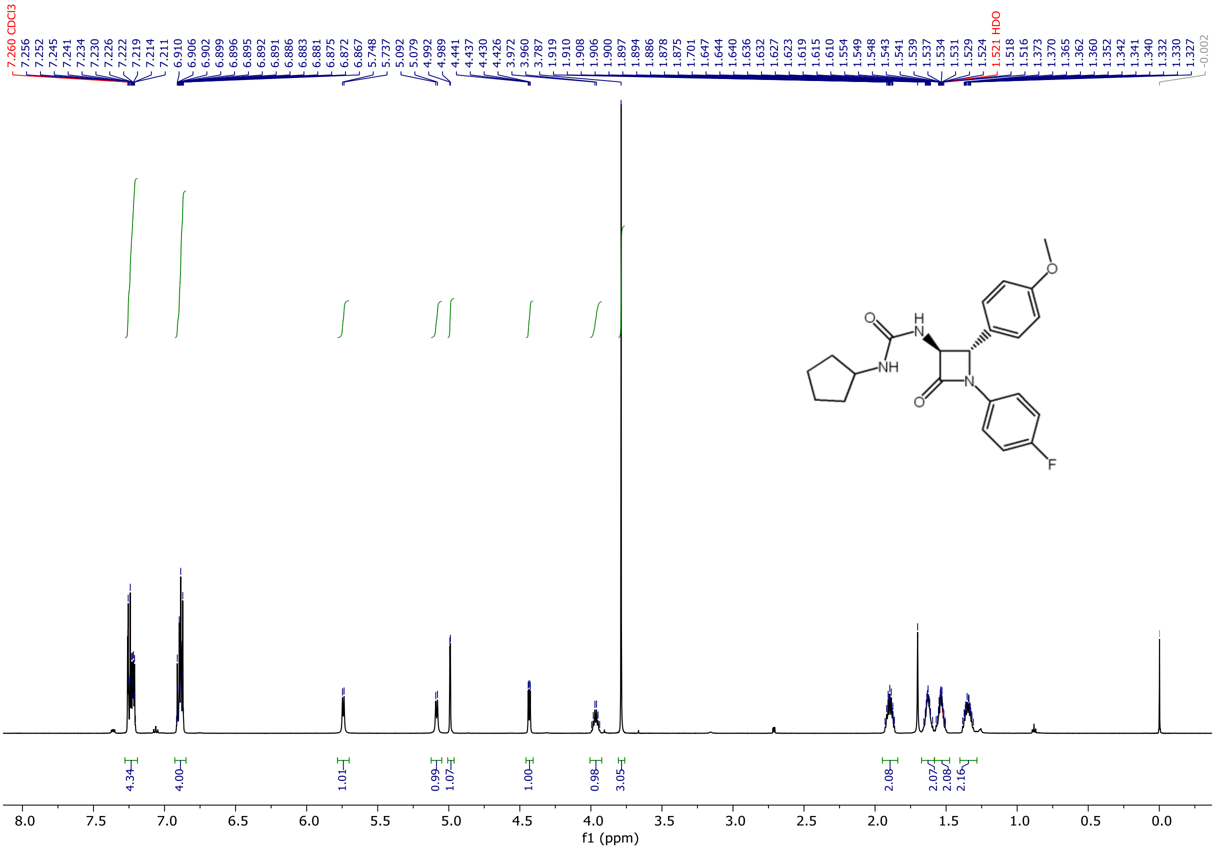

Figure S14. ^{1}H NMR (600 MHz; $CDCl_3$) spectra of compound **4c**.

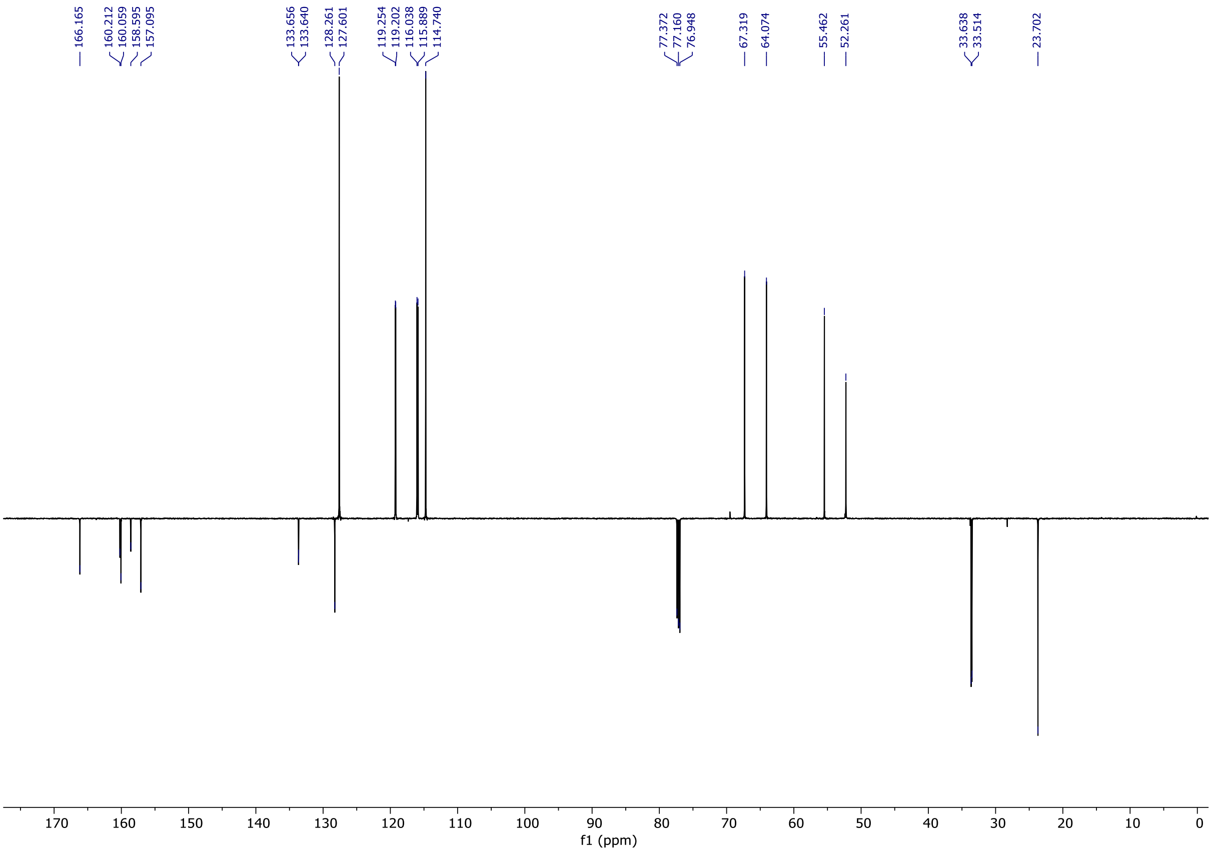

Figure S15. ^{13}C NMR (151 MHz; $CDCl_3$) spectra of compound **4c**.

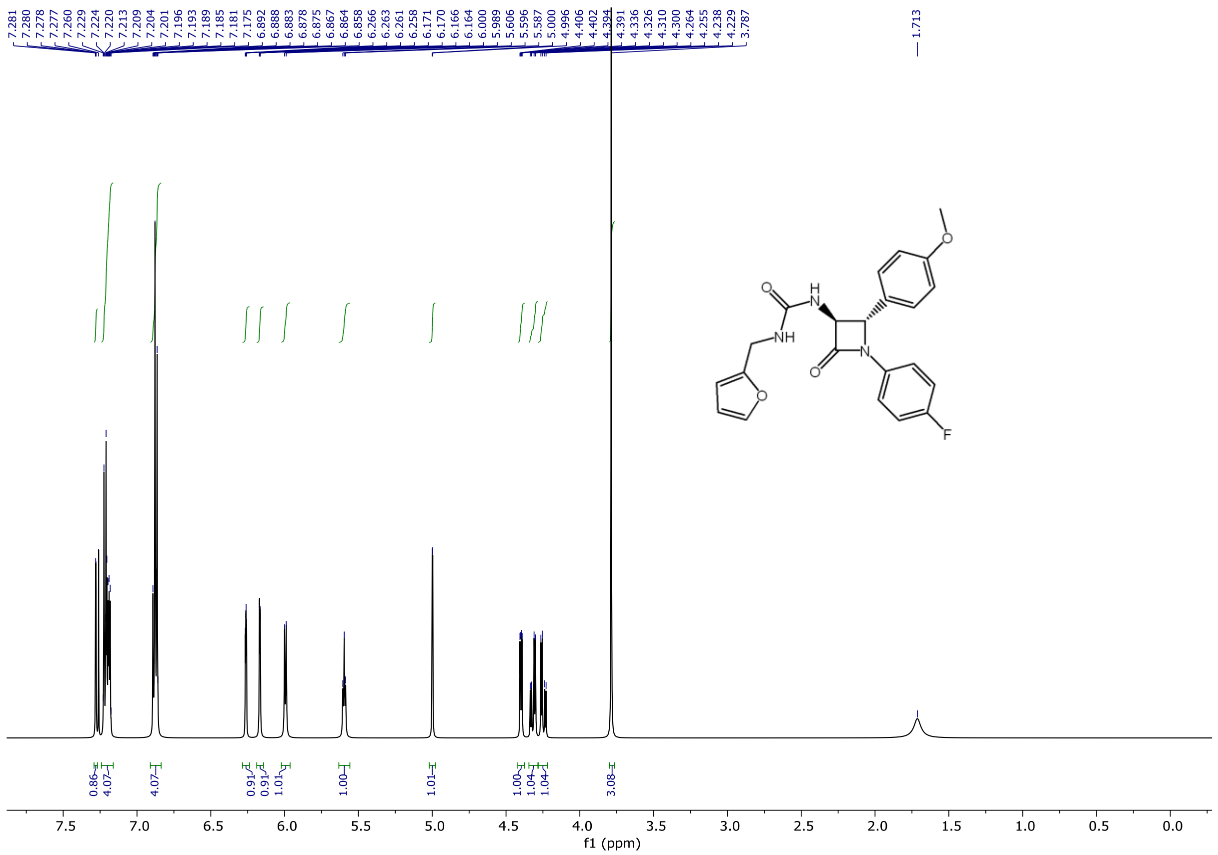

Figure S16. ^{1}H NMR (600 MHz; $CDCl_3$) spectra of compound **4d**.

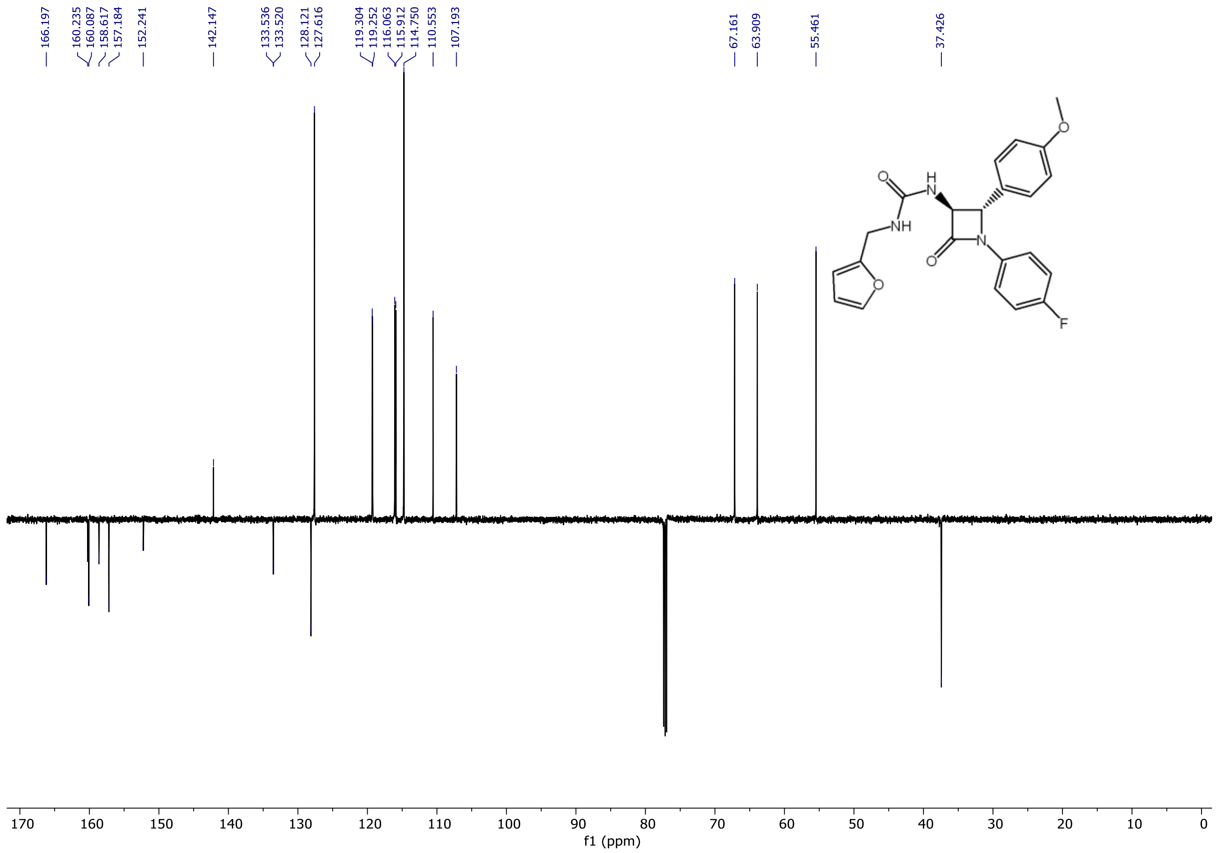

Figure S17. ^{13}C NMR (151 MHz; $CDCl_3$) spectra of compound **4d**.

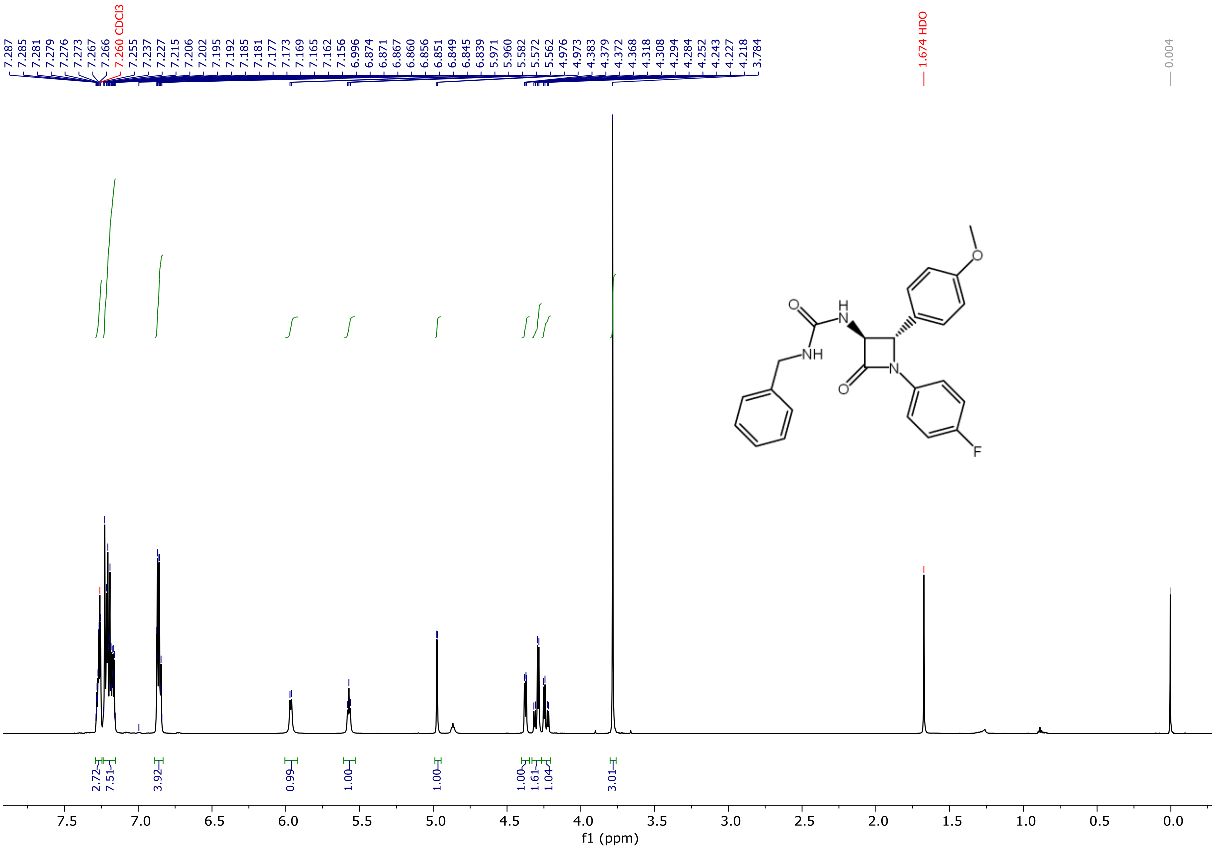

Figure S18. ^{1}H NMR (600 MHz; $CDCl_3$) spectra of compound **4e**.

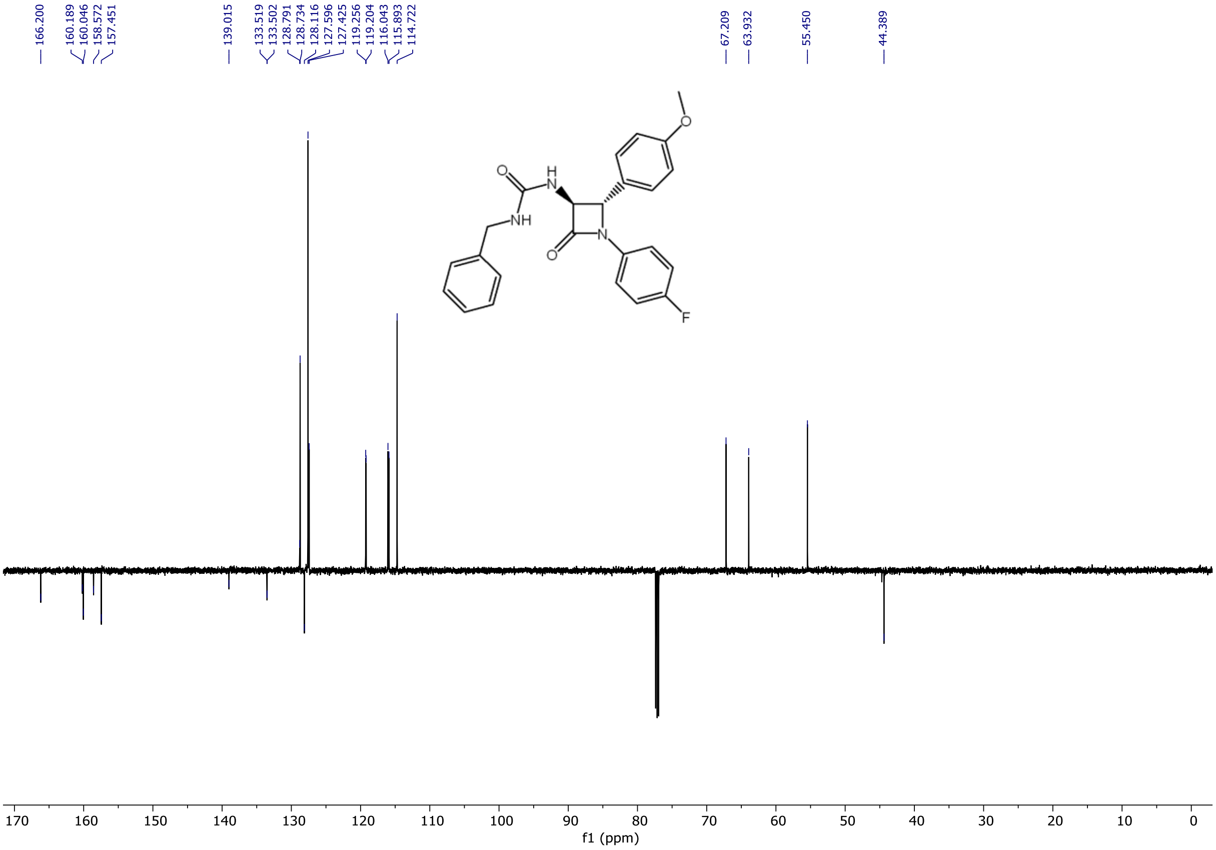

Figure S19. ^{13}C NMR (151 MHz; $CDCl_3$) spectra of compound **4e**.

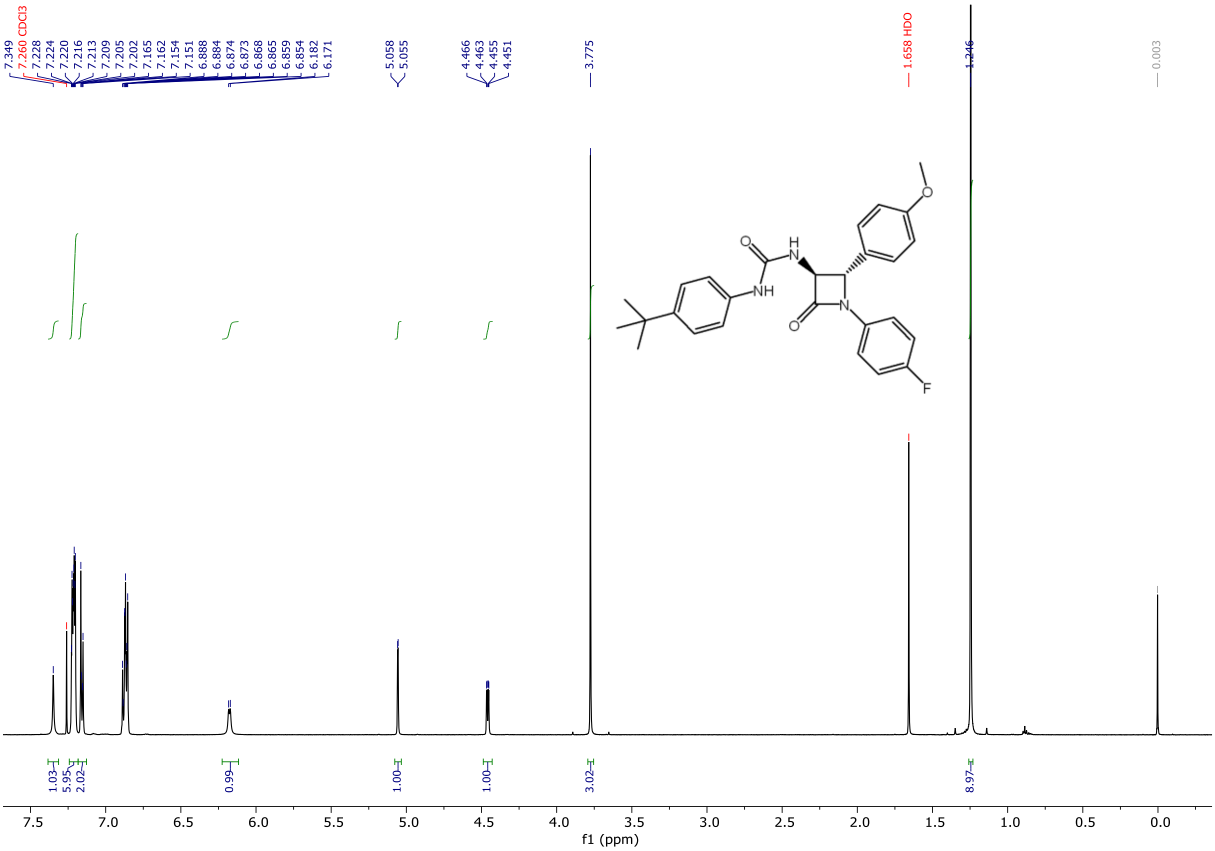

Figure S20. ^{1}H NMR (600 MHz; $CDCl_3$) spectra of compound **4f**.

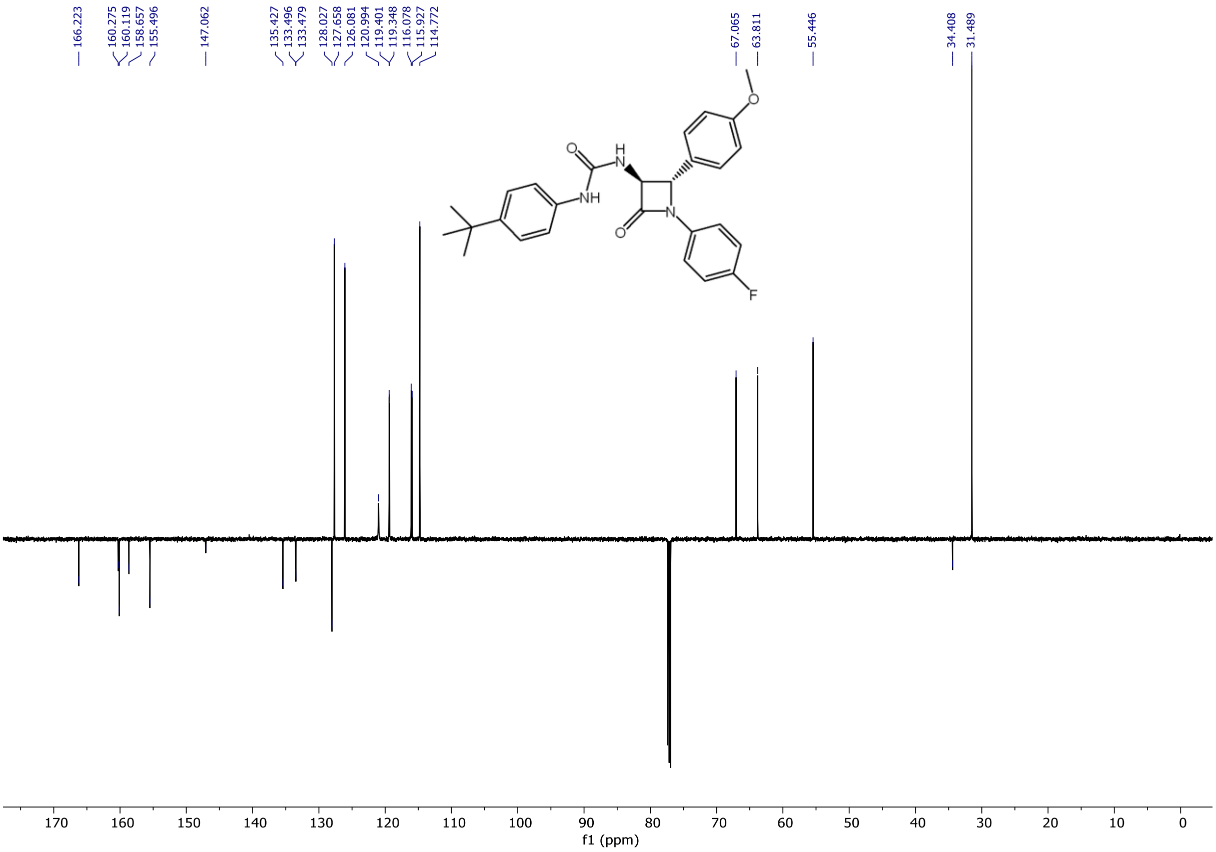

Figure S21. ^{13}C NMR (151 MHz; $CDCl_3$) spectra of compound **4f**.

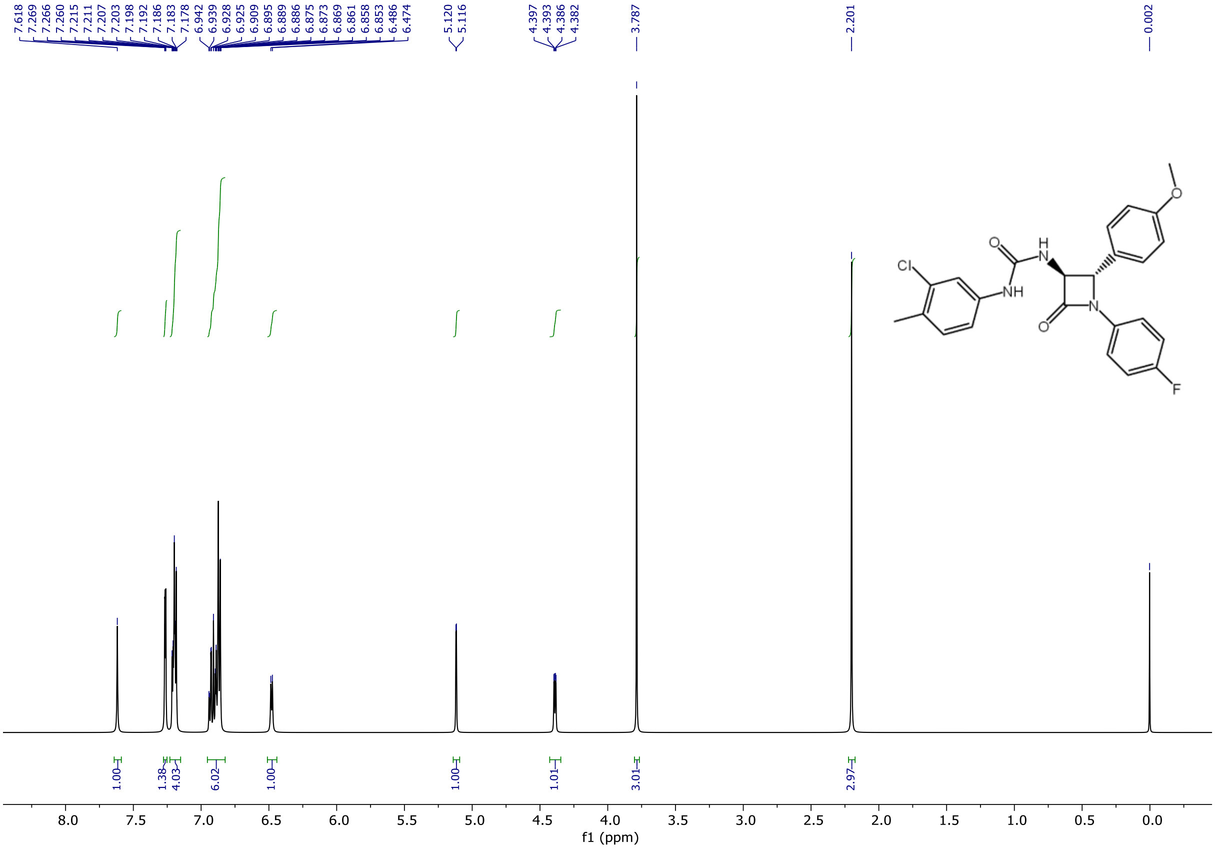

Figure S22. ^{1}H NMR (600 MHz; $CDCl_3$) spectra of compound **4g**.

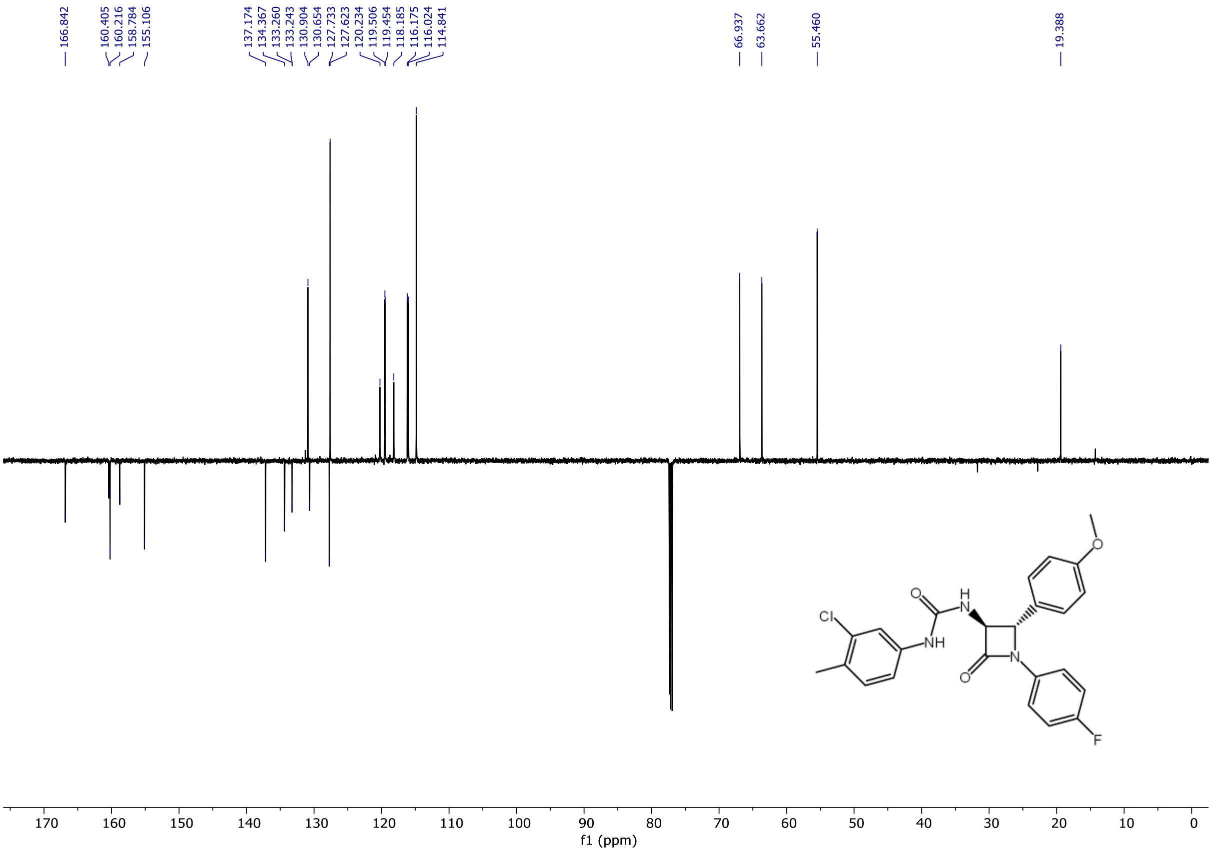

Figure S23. ^{13}C NMR (151 MHz; $CDCl_3$) spectra of compound **4g**.

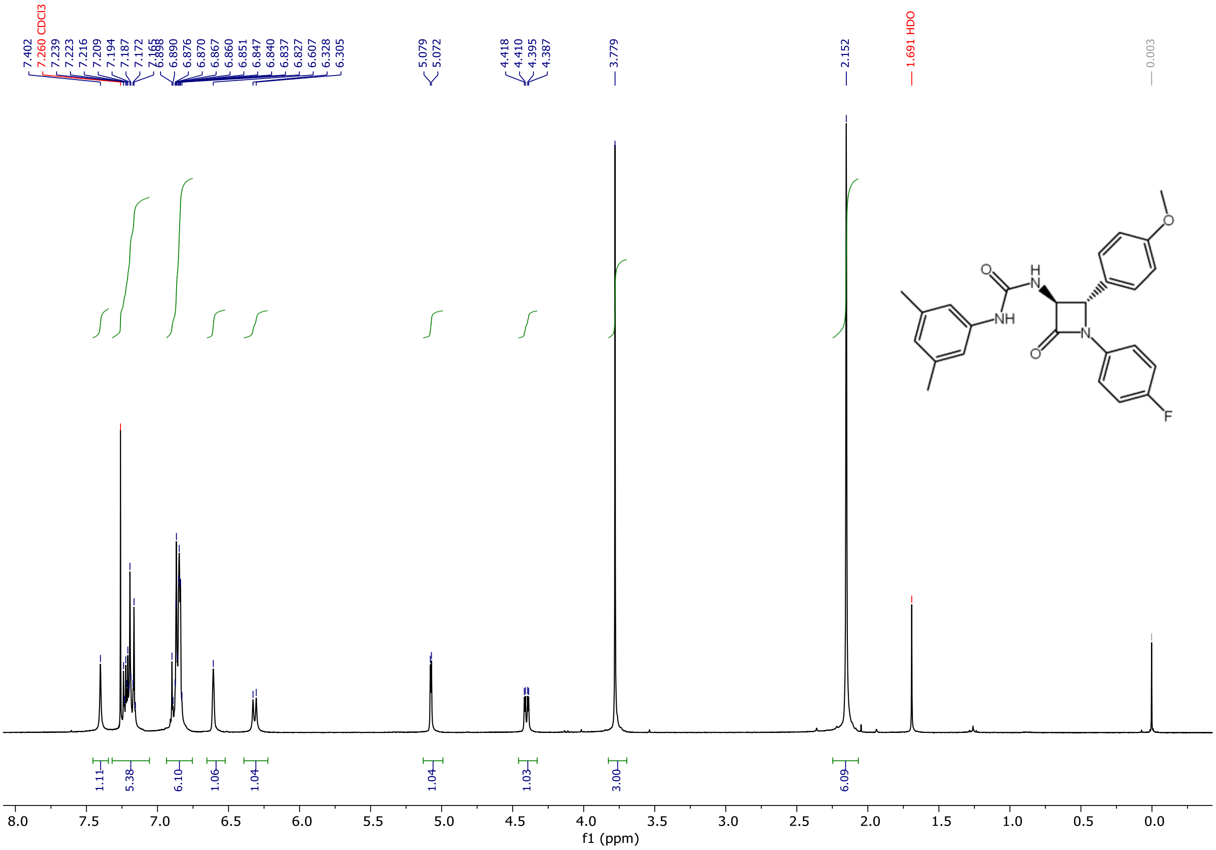

Figure S24. ^{1}H NMR (600 MHz; $CDCl_3$) spectra of compound **4h**.

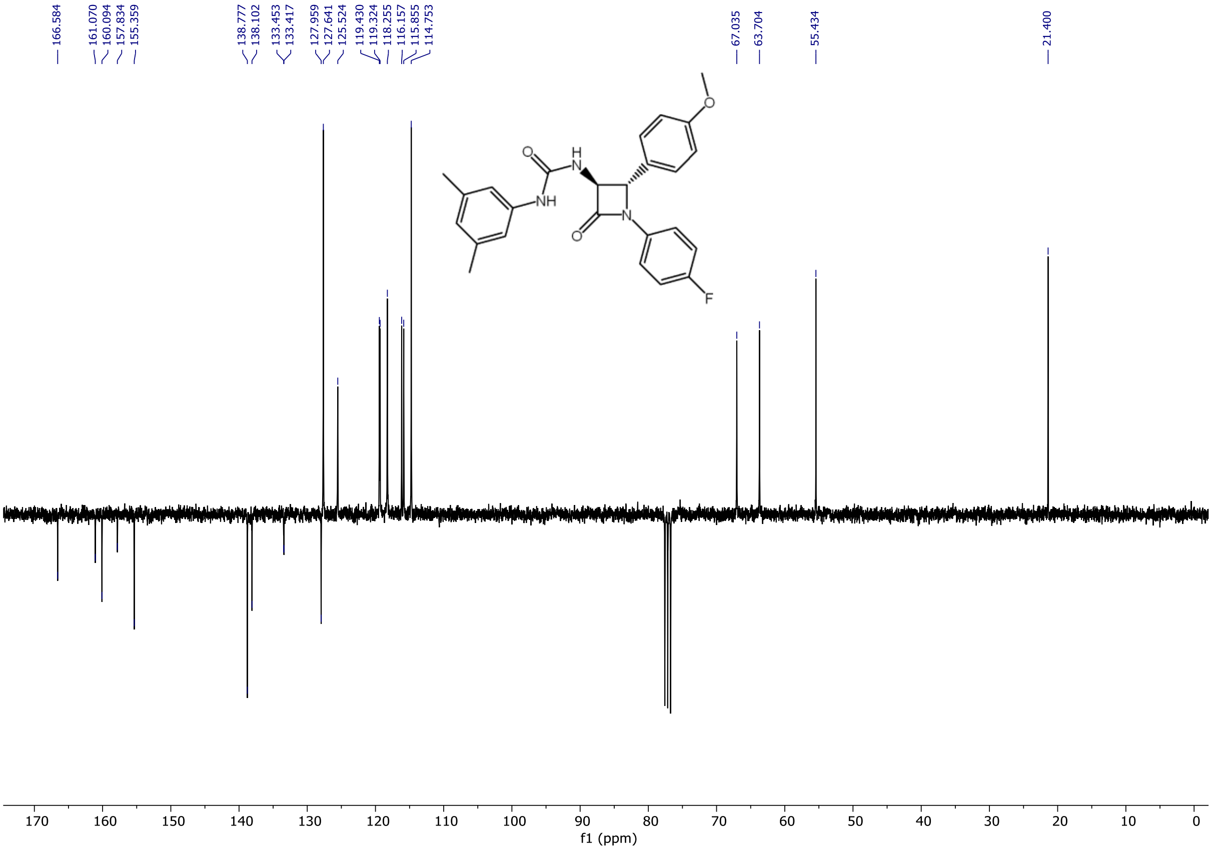

Figure S25. ^{13}C NMR (151 MHz; $CDCl_3$) spectra of compound **4h**.

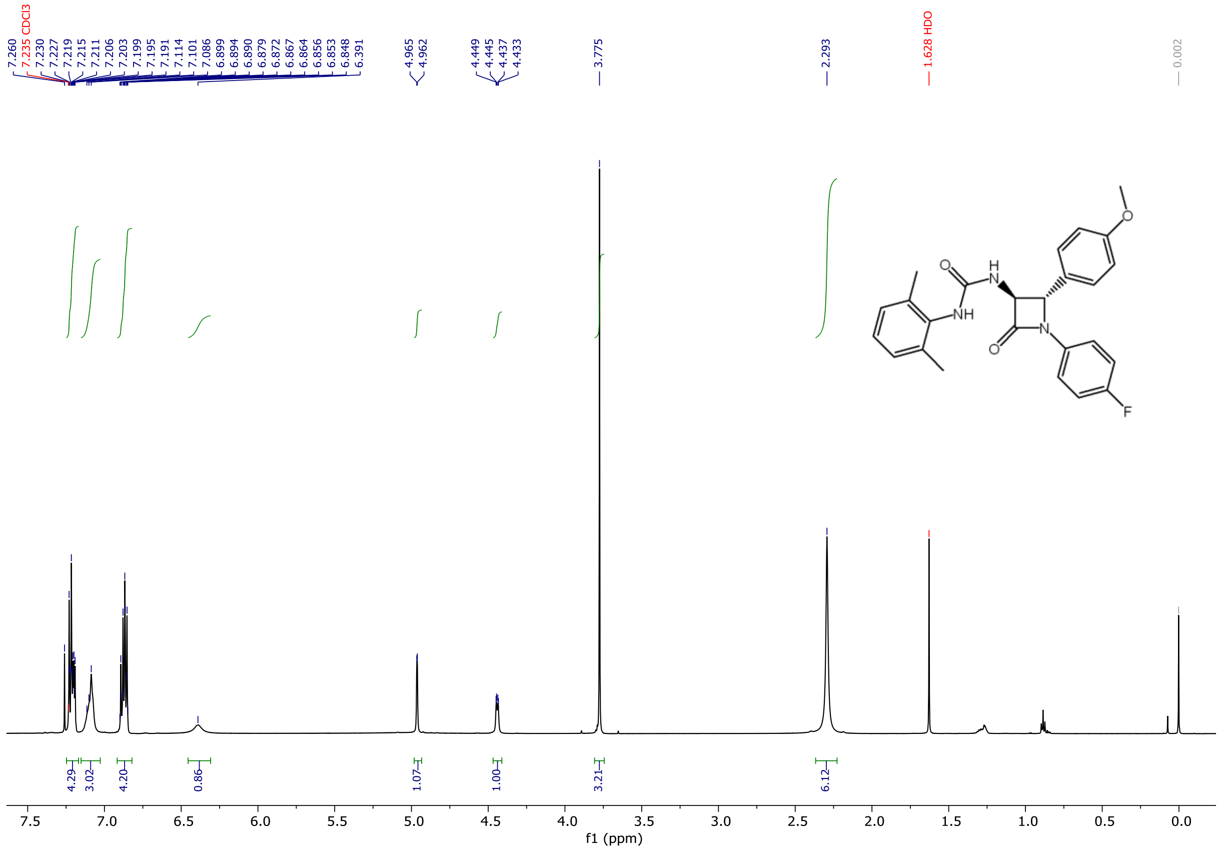

Figure S26. ^{1}H NMR (600 MHz; $CDCl_3$) spectra of compound **4i**.

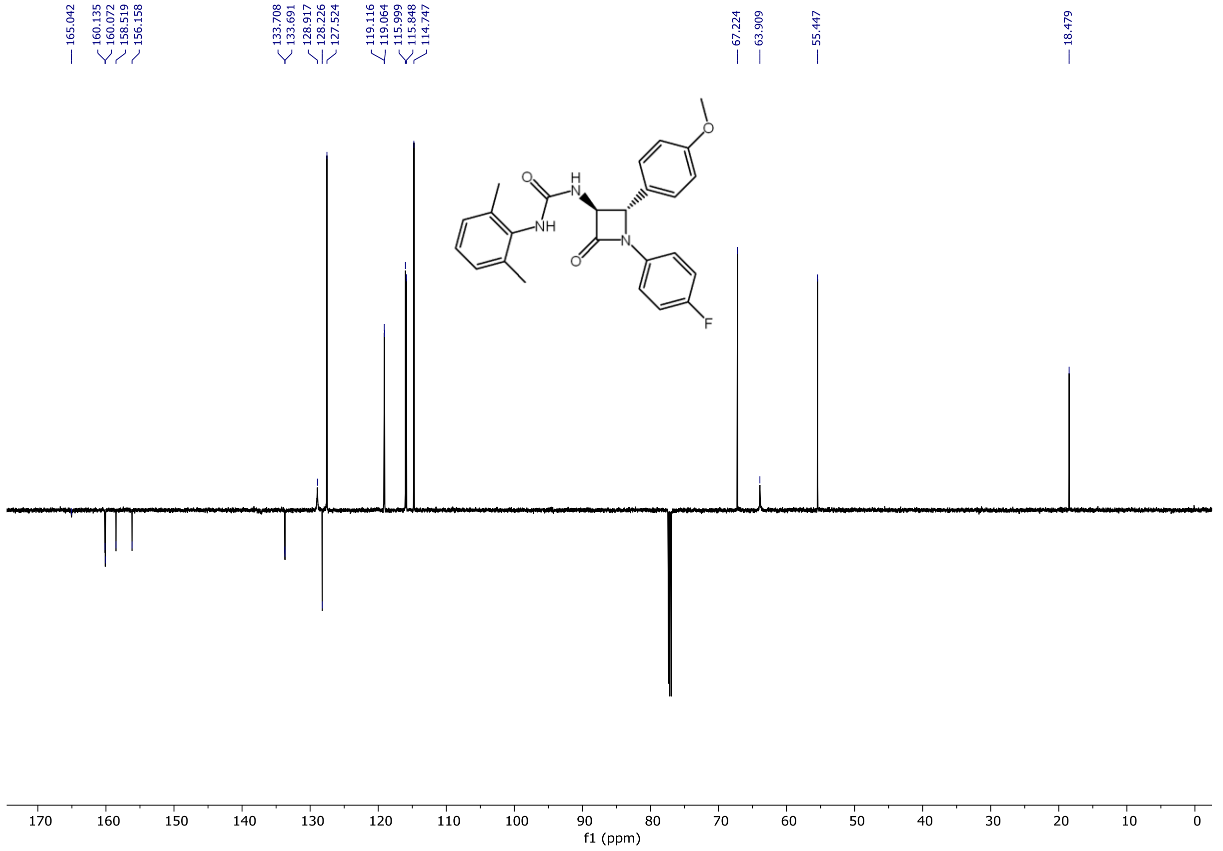

Figure S27. ^{13}C NMR (151 MHz; $CDCl_3$) spectra of compound **4i**.

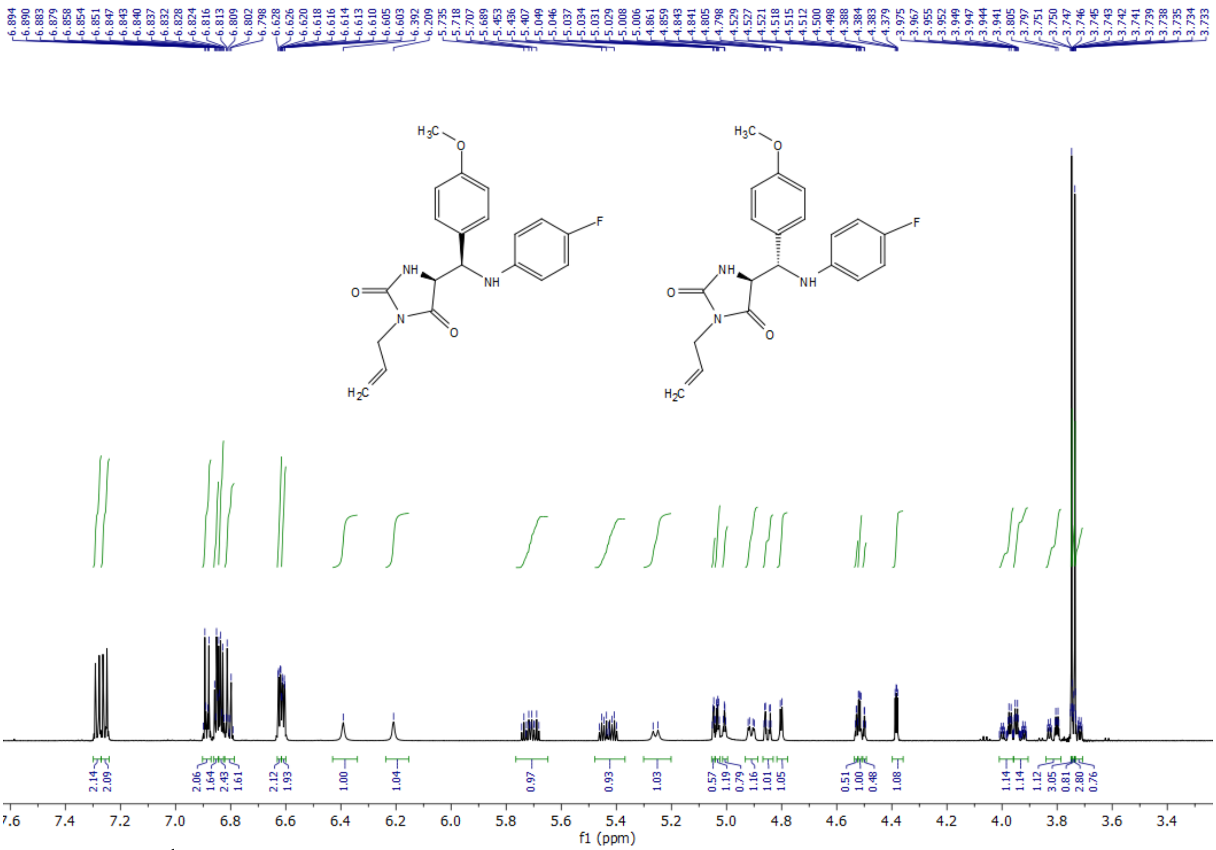

Figure S28. ^{1}H NMR (600 MHz; CD_3CN) spectra of compound **5a**.

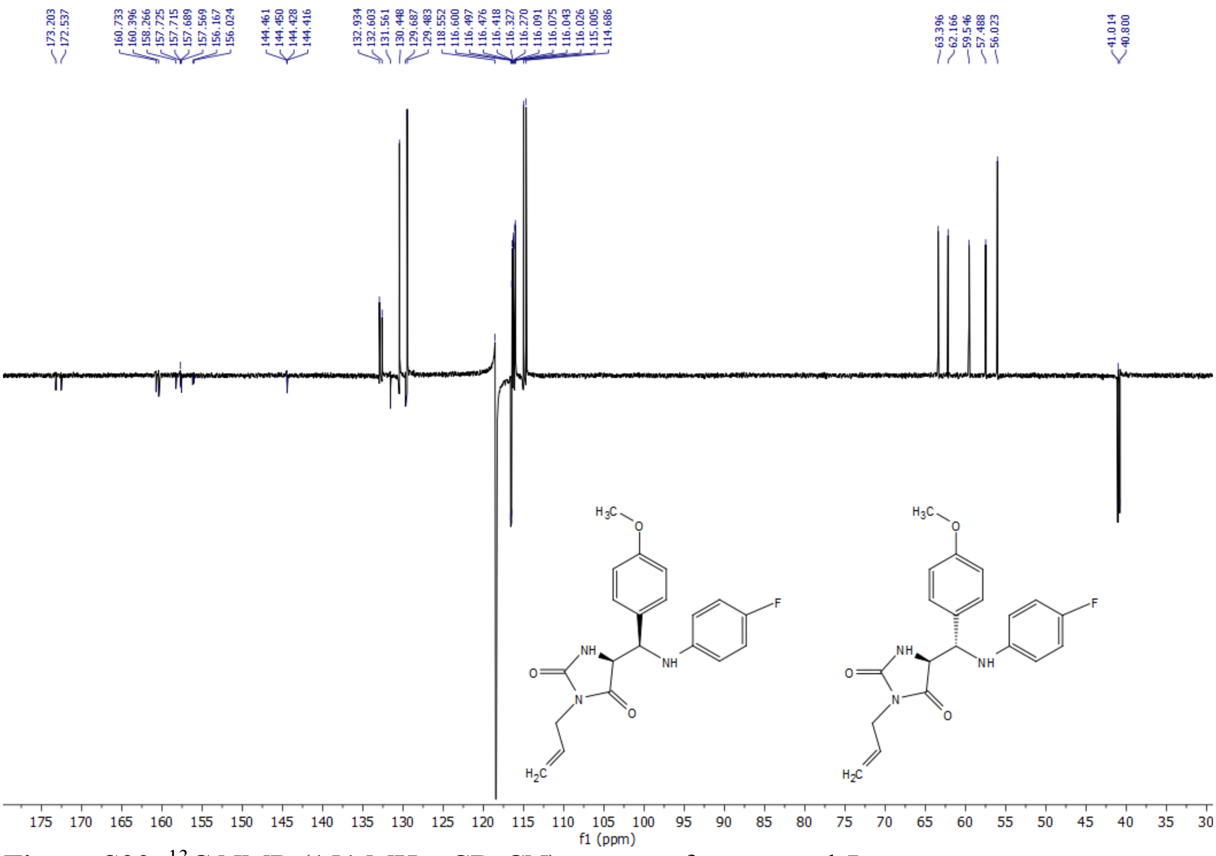

Figure S29. ^{13}C NMR (151 MHz; CD_3CN) spectra of compound **5a**.

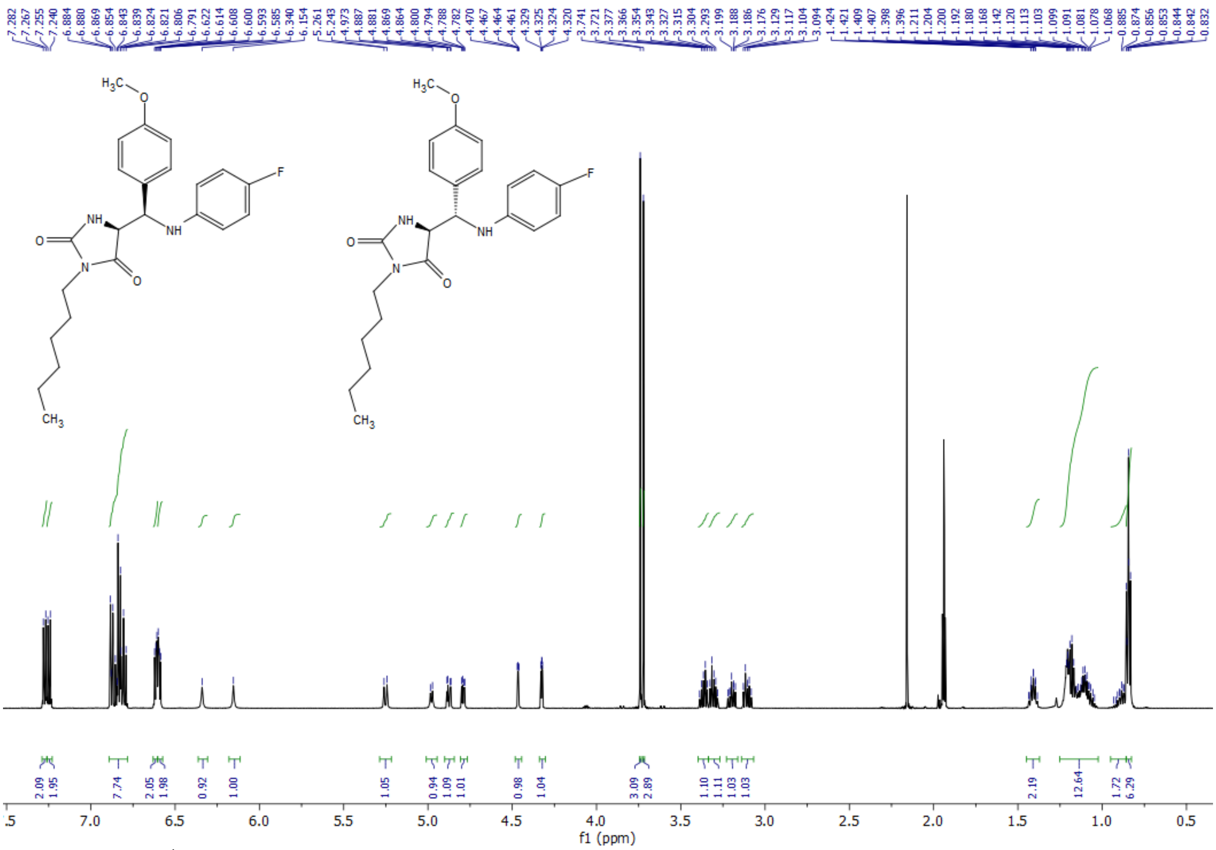

Figure S30. ^{1}H NMR (600 MHz; CD_3CN) spectra of compound **5b**.

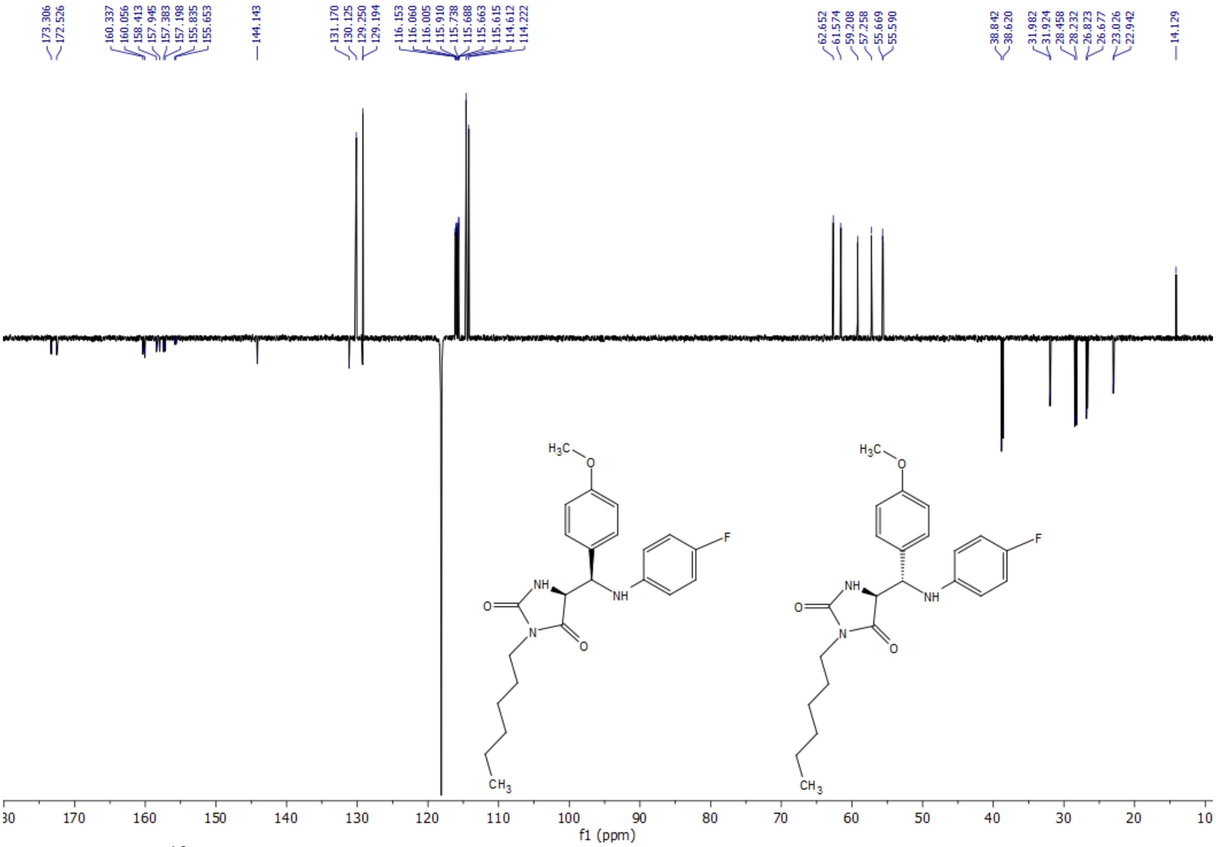

Figure S31. ^{13}C NMR (151 MHz; CD_3CN) spectra of compound **5b**.

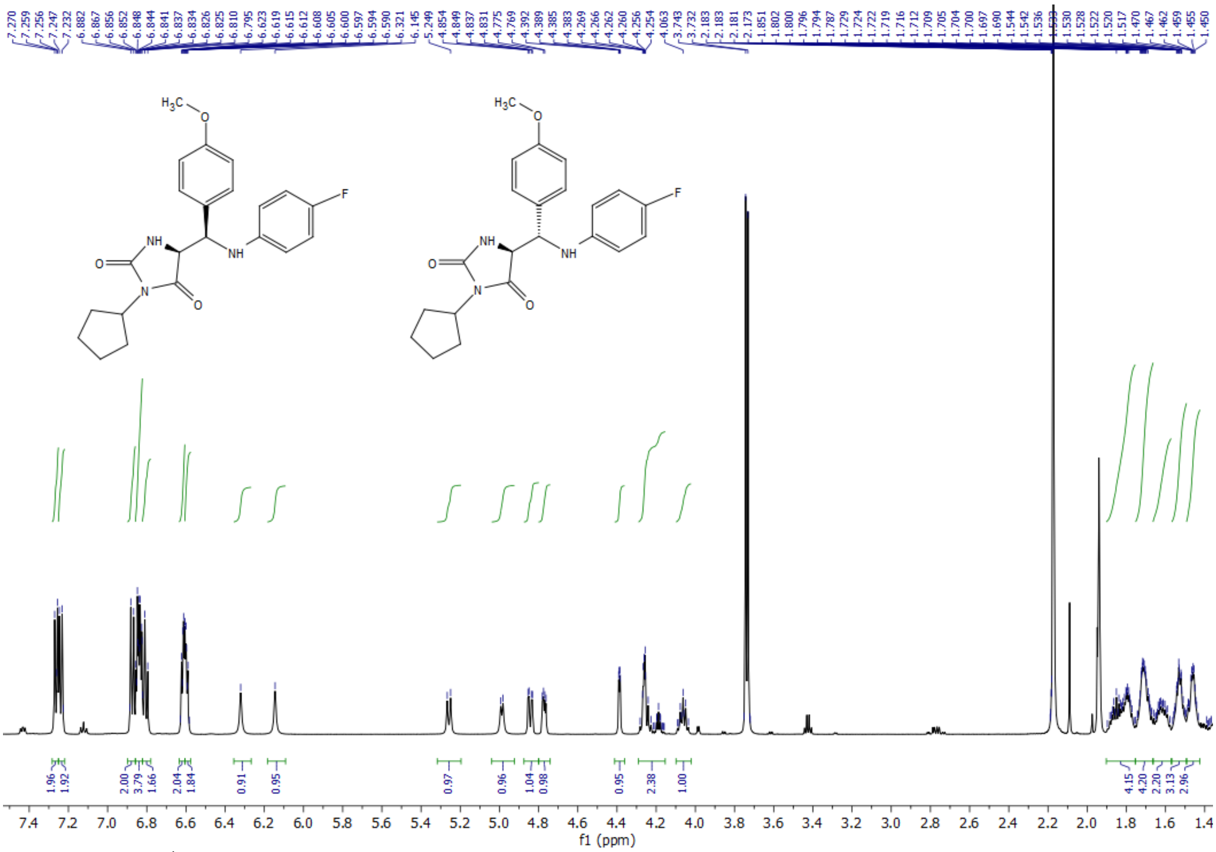

Figure S32. ^{1}H NMR (600 MHz; CD_3CN) spectra of compound **5c**.

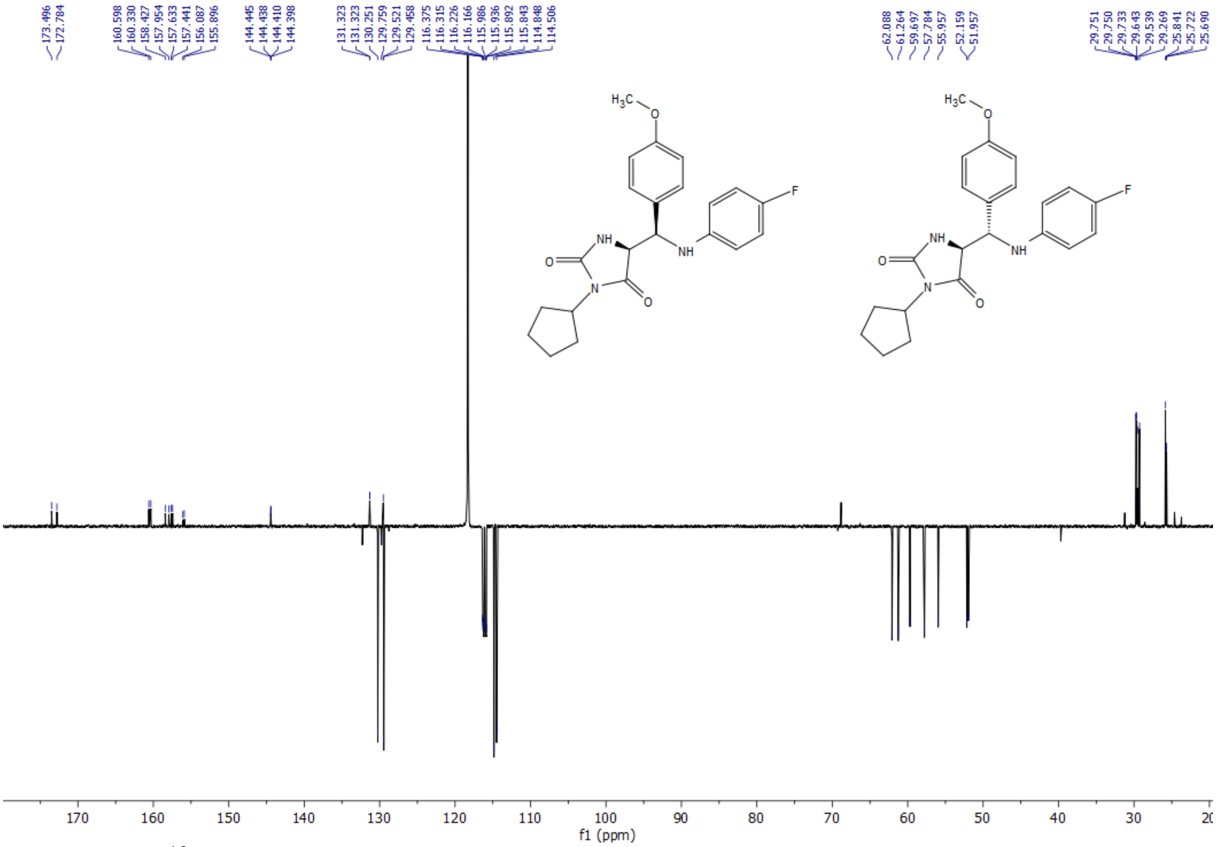

Figure S33. ^{13}C NMR (151 MHz; CD_3CN) spectra of compound **5c**.

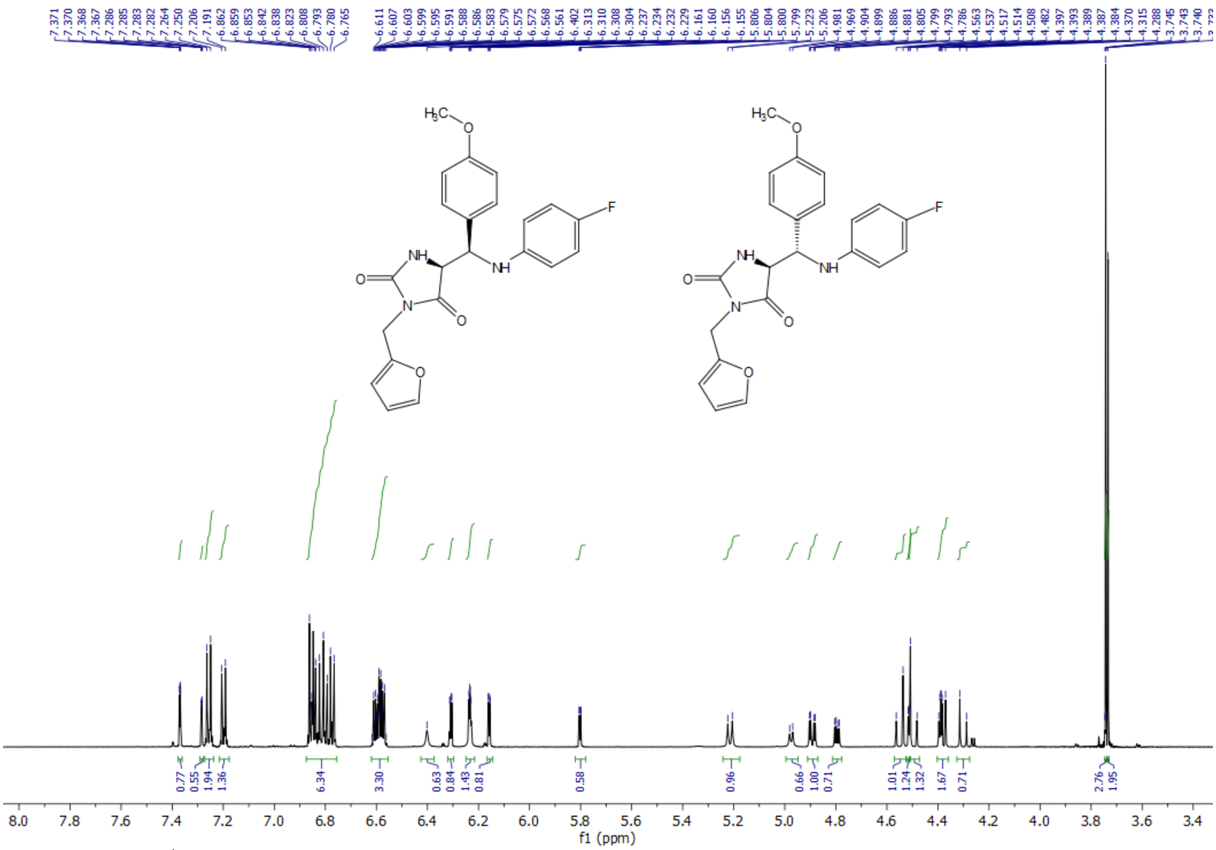

Figure S34. ^{1}H NMR (600 MHz; CD_3CN) spectra of compound **5d**.

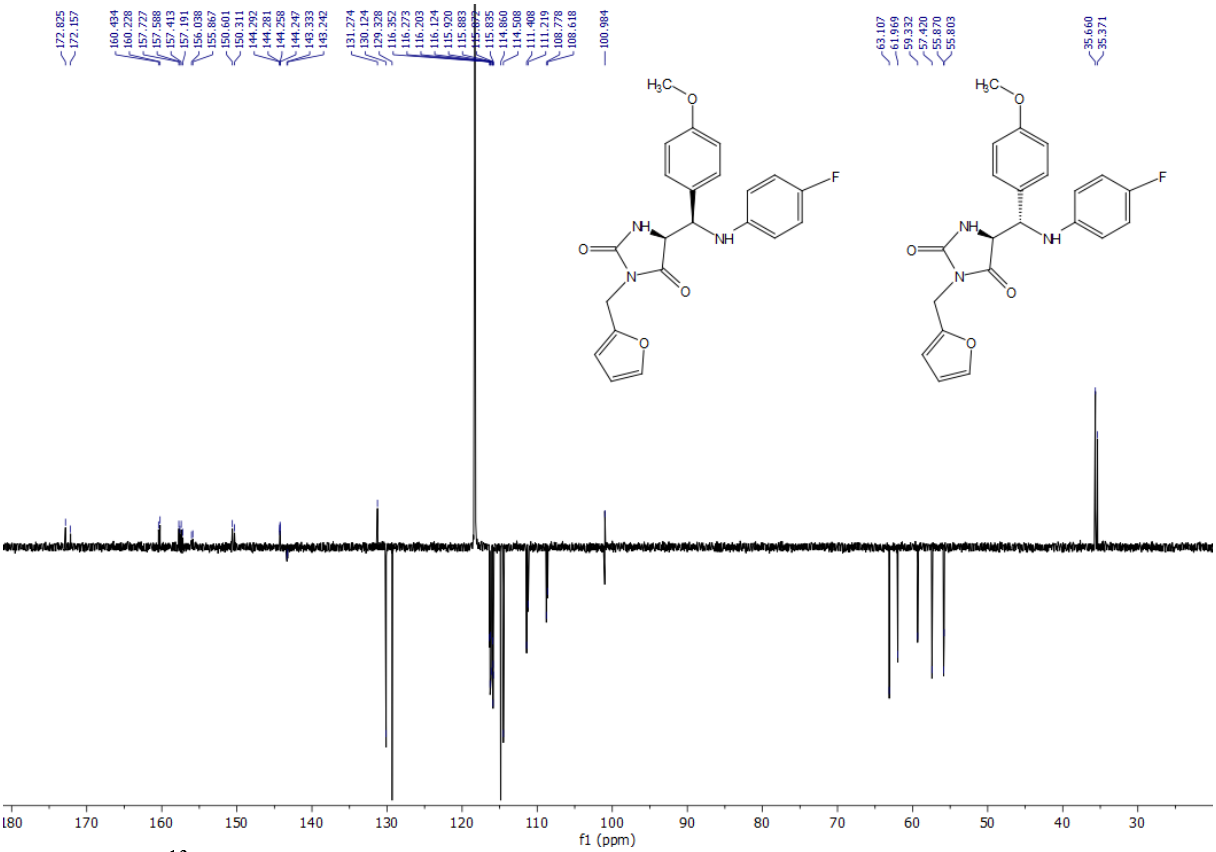

Figure S35. ^{13}C NMR (151 MHz; CD_3CN) spectra of compound **5d**.

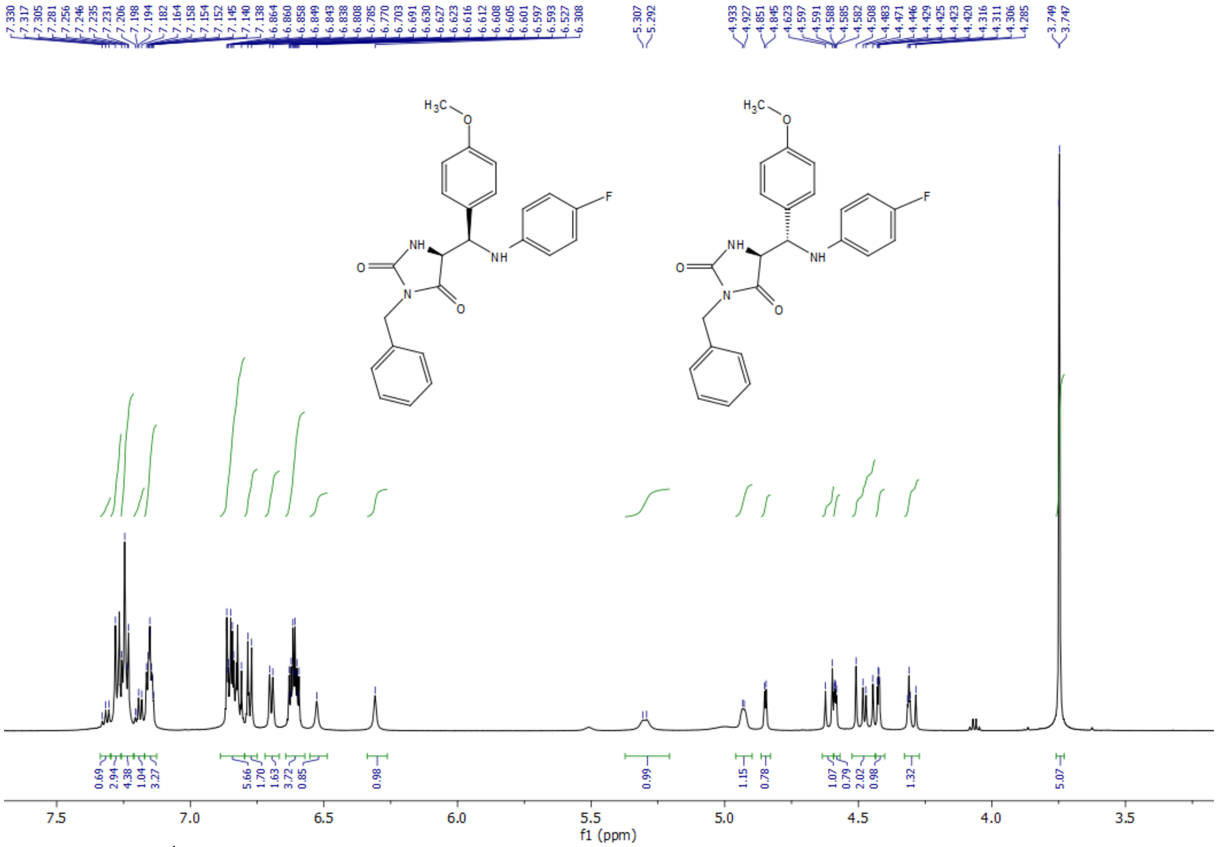

Figure S36. ^{1}H NMR (600 MHz; CD_3CN) spectra of compound **5e**.

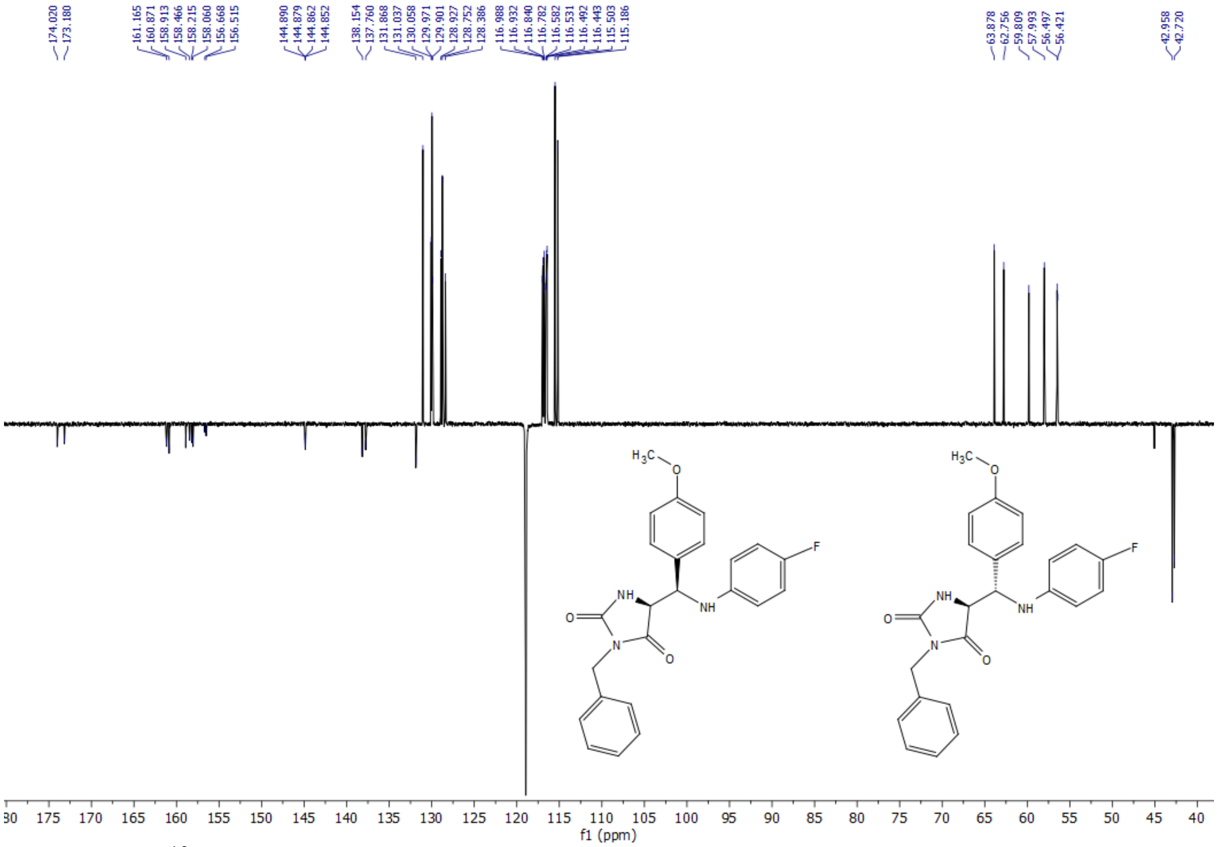

Figure S37. ^{13}C NMR (151 MHz; CD_3CN) spectra of compound **5e**.

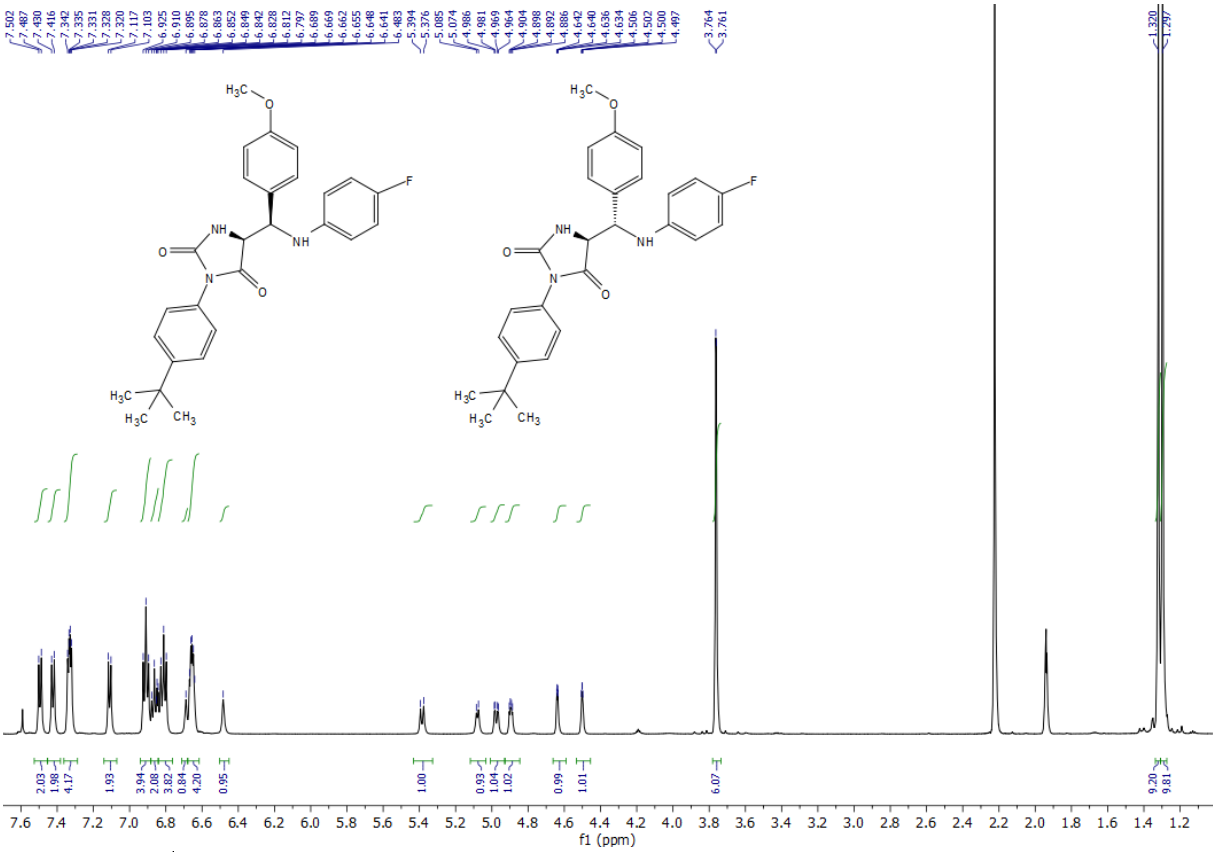

Figure S38. ^{1}H NMR (600 MHz; CD_3CN) spectra of compound **5f**.

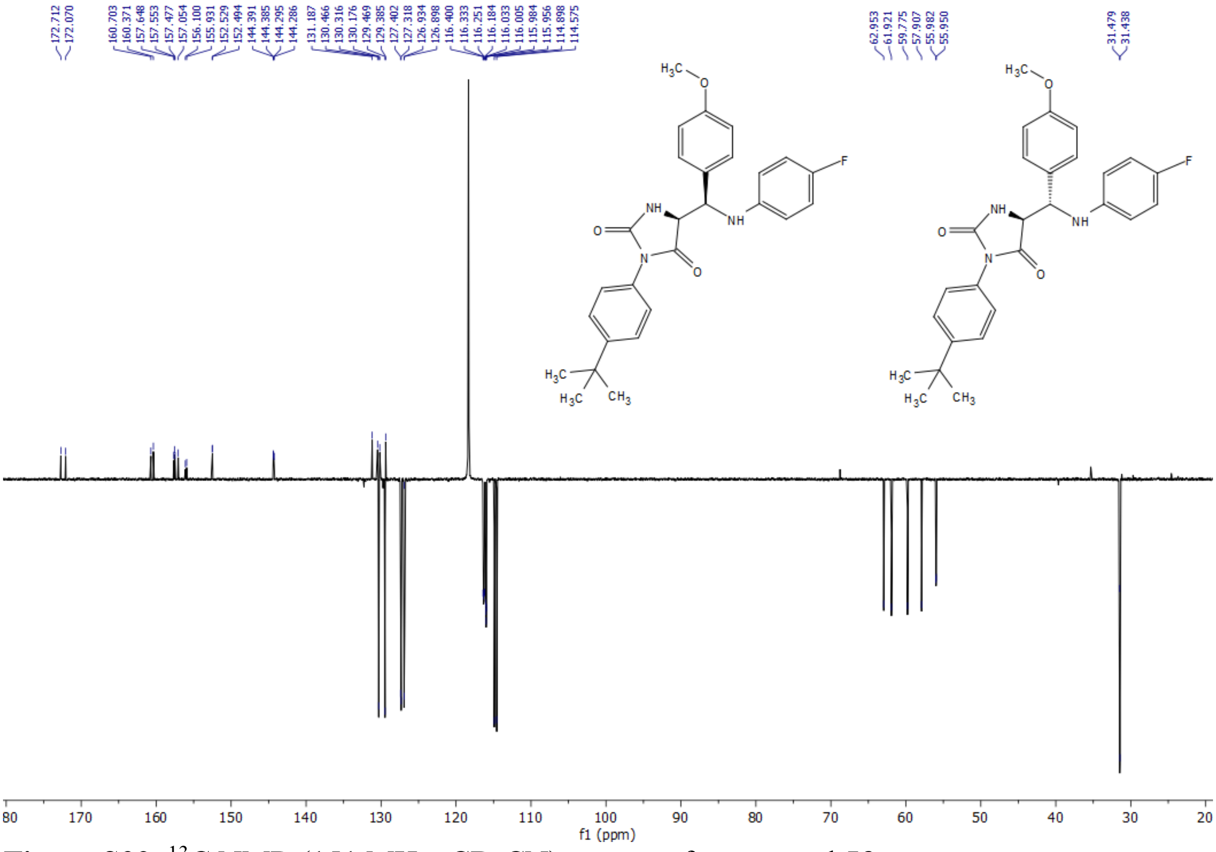

Figure S39. ^{13}C NMR (151 MHz; CD_3CN) spectra of compound **5f**.

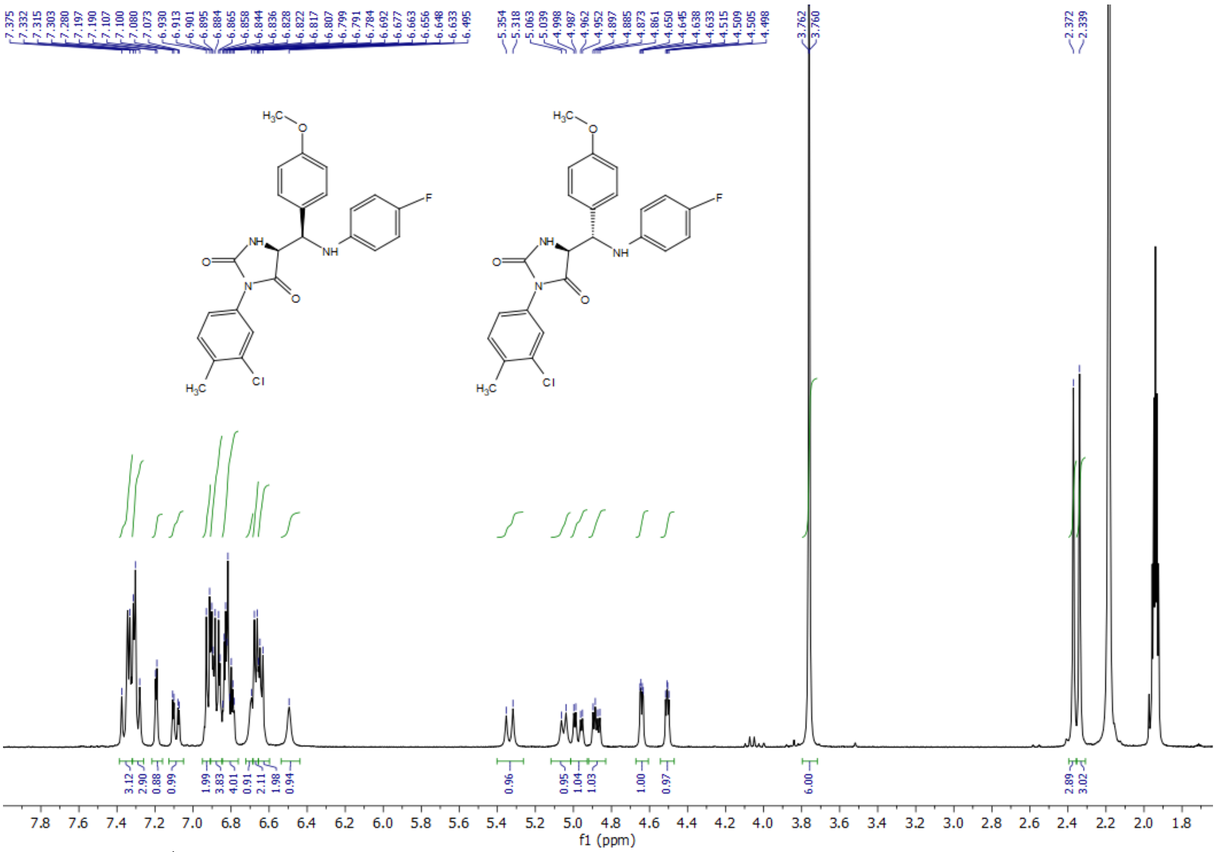

Figure S40. ^{1}H NMR (600 MHz; CD_3CN) spectra of compound **5g**.

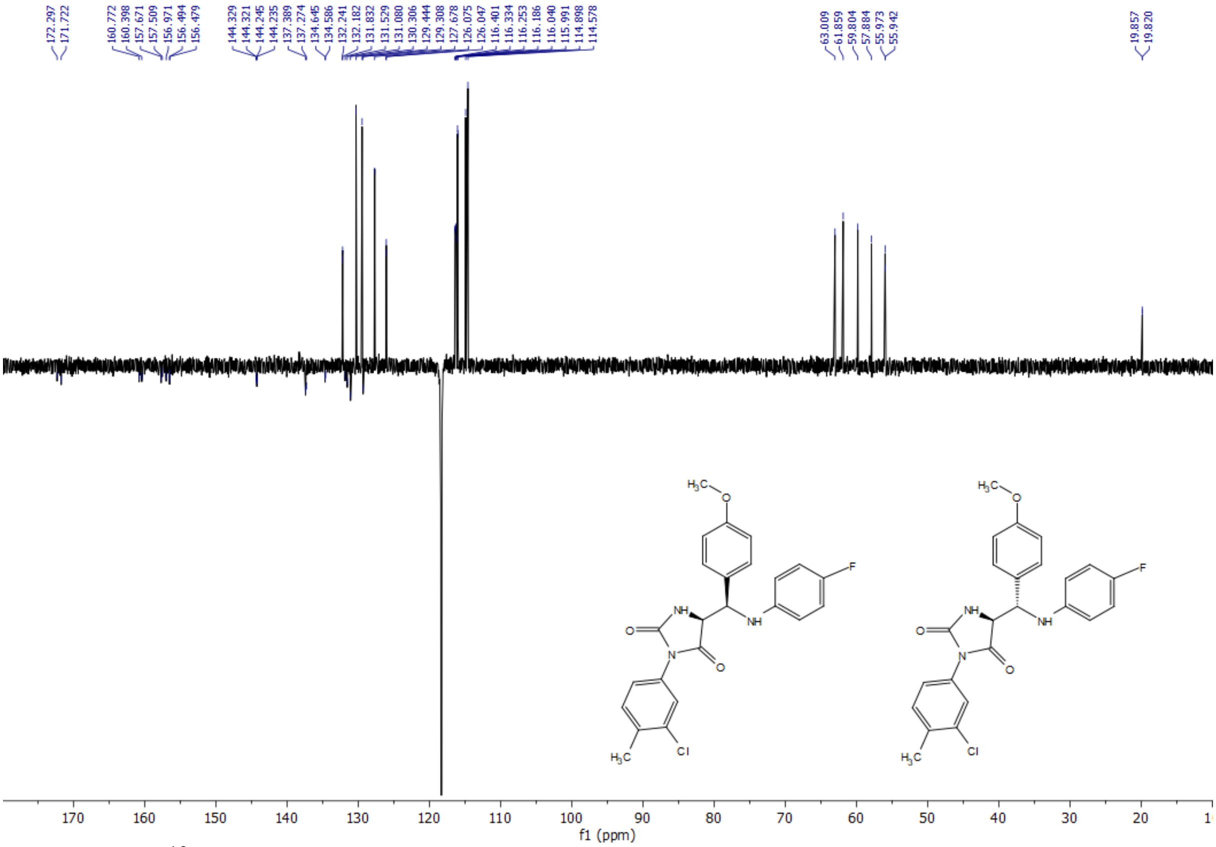

Figure S41. ^{13}C NMR (151 MHz; CD_3CN) spectra of compound **5g**.

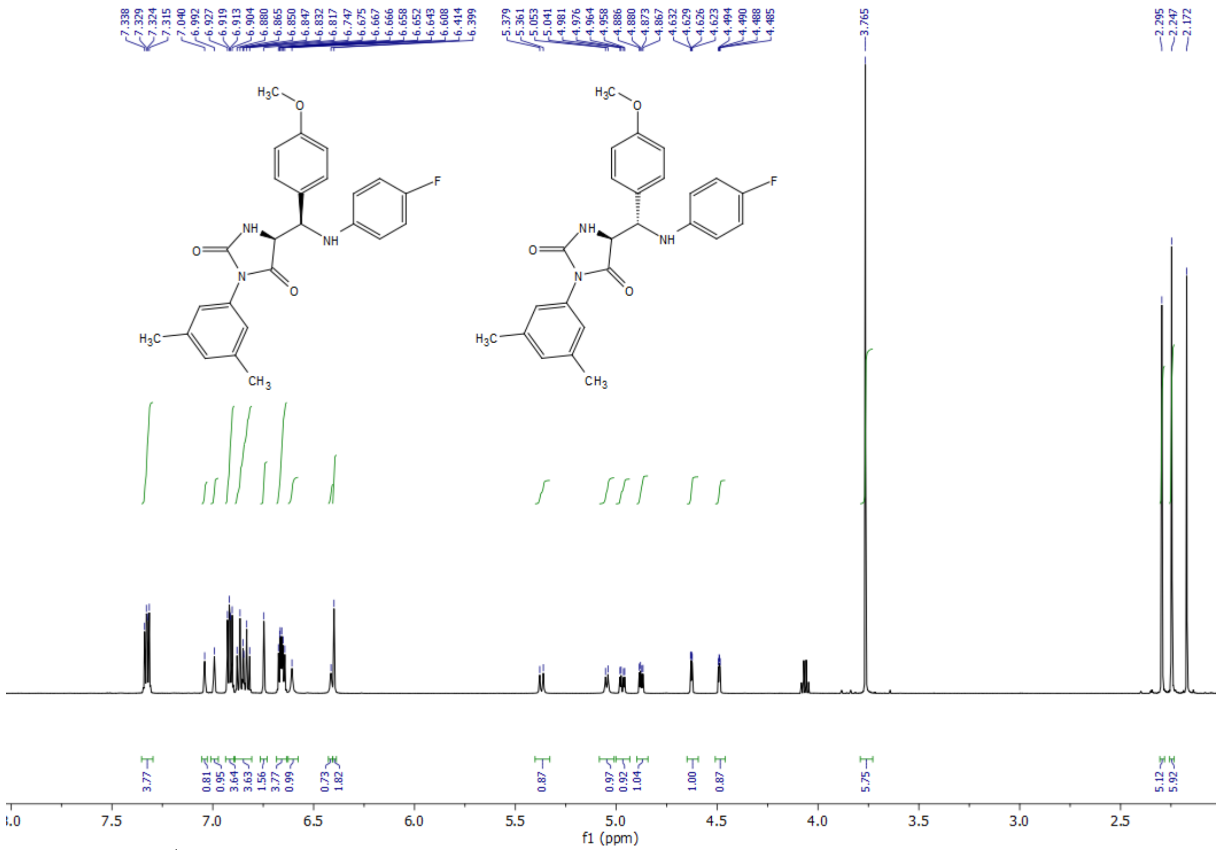

Figure S42. ^{1}H NMR (600 MHz; CD_3CN) spectra of compound **5h**.

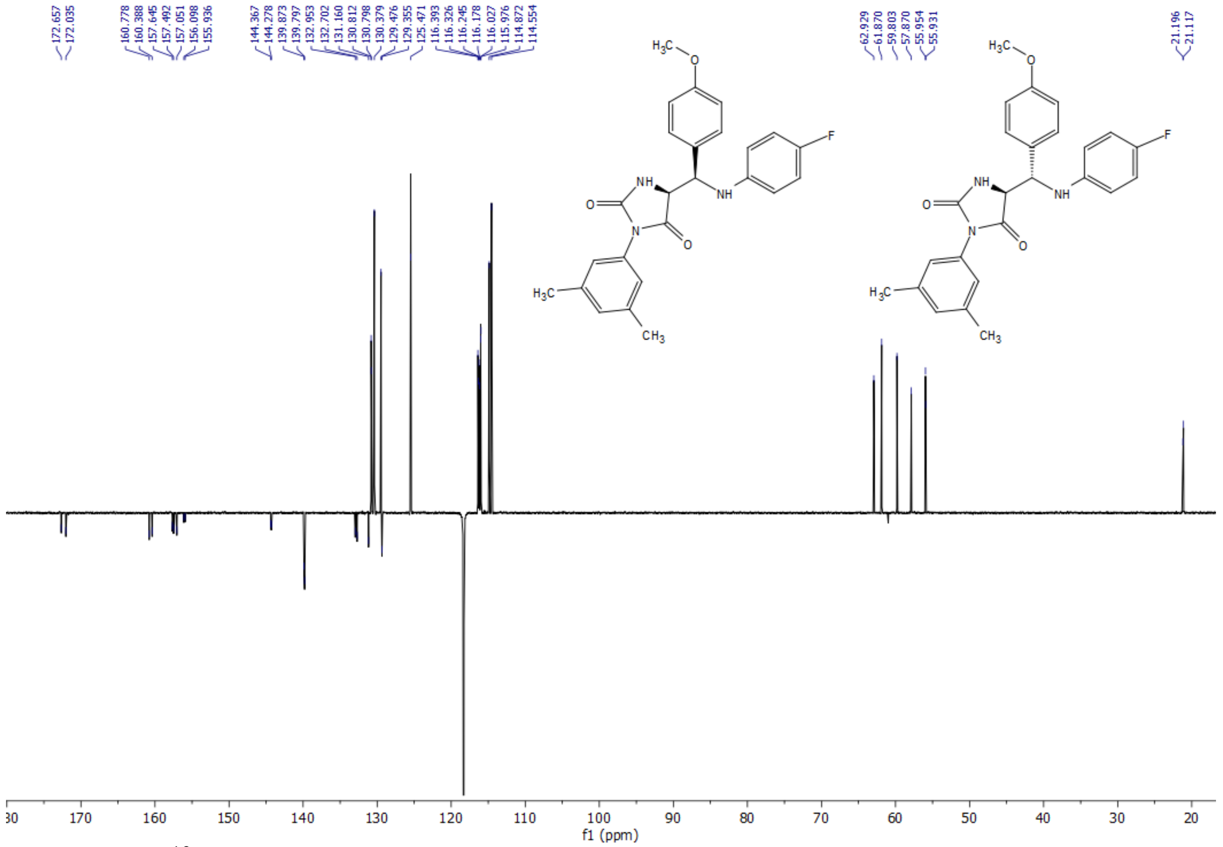

Figure S43. ^{13}C NMR (151 MHz; CD_3CN) spectra of compound **5h**.

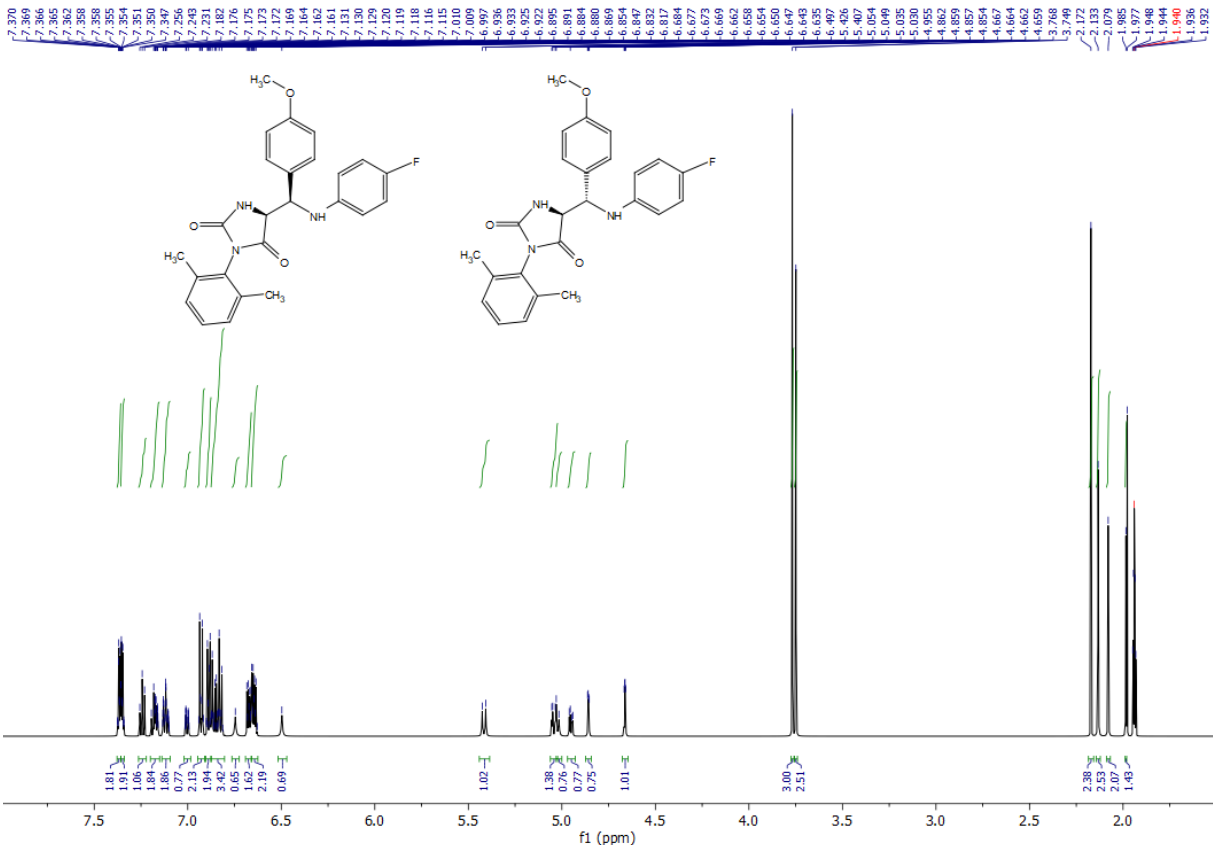

Figure S44. ^{1}H NMR (600 MHz; CD_3CN) spectra of compound **5i**.

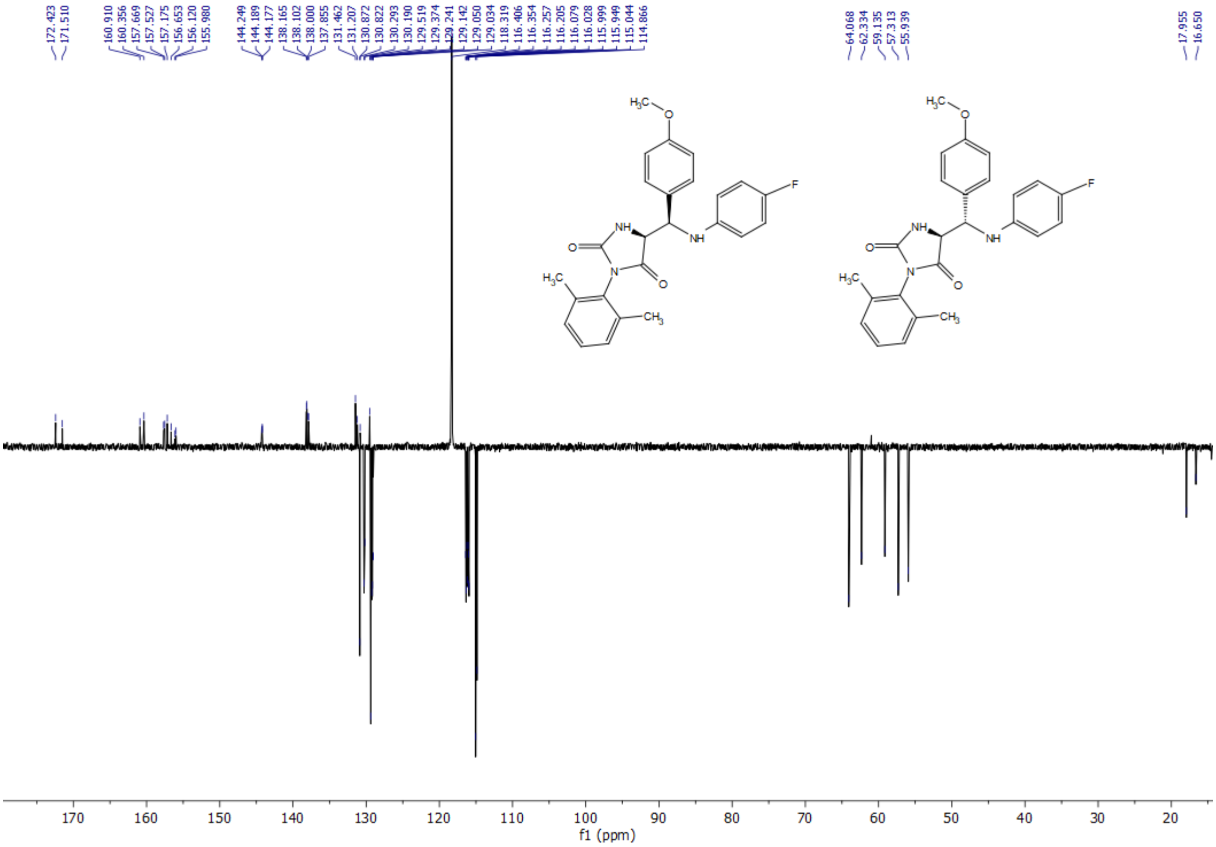

Figure S45. ^{13}C NMR (151 MHz; CD_3CN) spectra of compound **5i**.

2. NMR Analysis of allyl hydantoin *syn*-5a and *anti*-5a

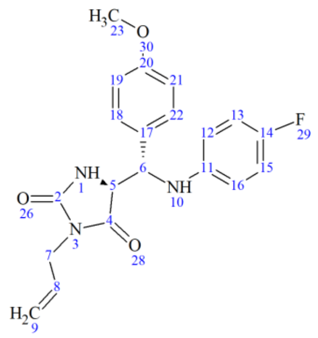

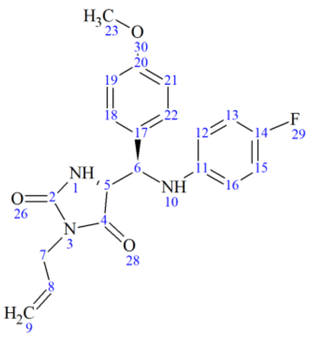

^{1}H NMR (600 MHz, ACETONITRILE-d$_3$): δ = 3.73 (ddt, J = 16.2, 5.1, 1.8 Hz, 1 H, 7<">), 3.74 (s, 3 H, 23), 3.82 (ddt, J = 16.1, 5.1, 1.5 Hz, 1 H, 7<'>), 4.52 (dq, J = 17.2, 1.5 Hz, 1 H, 9<b>), 4.52 (dd, J = 3.7, 1.8 Hz, 1 H, 5), 4.80 (dd, J = 6.4, 3.9 Hz, 1 H, 6), 4.85 (dq, J = 10.3, 1.1 Hz, 1 H, 9<a>), 5.00 (br d, J = 7.3 Hz, 1 H, 10), 5.43 (ddt, J = 17.1, 10.5, 4.9 Hz, 1 H, 8), 6.38 (br s, 1 H, 1), 6.62 (dd, J = 8.8, 4.4 Hz, 2 H, 16, 12), 6.84 (br d, J = 8.4 Hz, 2 H, 21, 19), 6.84 (br t, J = 9.0 Hz, 2 H, 15, 13), 7.26 (d, J = 8.8 Hz, 2 H, 22, 18) ppm

Shift1 (ppm)	H's	Type	J (Hz)	Atom1	Multiplet1
3.73	1.000	ddt	16.2, 5.1, 1.8	7<">	M03
3.74	3.000	s	-	23	M16
3.82	1.000	ddt	16.1, 5.1, 1.5	7<'>	M04
4.52	1.000	dd	3.7, 1.8	5	M15
4.52	1.000	dq	17.2, 1.5	9<b>	M05
4.80	1.000	dd	6.4, 3.9	6	M06
4.85	1.000	dq	10.3, 1.1	9<a>	M07
5.00	1.000	br d	7.3	10	M08
5.43	1.000	ddt	17.1, 10.5, 4.9	8	M09
6.38	1.000	br s	-	1	M10
6.62	2.000	dd	8.8, 4.4	16, 12	M11
6.84	2.000	br t	9.0	15, 13	M12
6.84	2.000	br d	8.4	21, 19	M14
7.26	2.000	d	8.8	22, 18	M13

Solvent	ACETONITRILE-d3
Pulse Sequence	zg30
Temperature (degree C)	25.000
Acquisition Time (sec)	1.3631
Number of Transients	64
Points Count	32768
Sweep Width (Hz)	12018.86
Spectrum Offset (Hz)	5388.5049
Original Points Count	16384
Nucleus	1H
Frequency (MHz)	600.1301

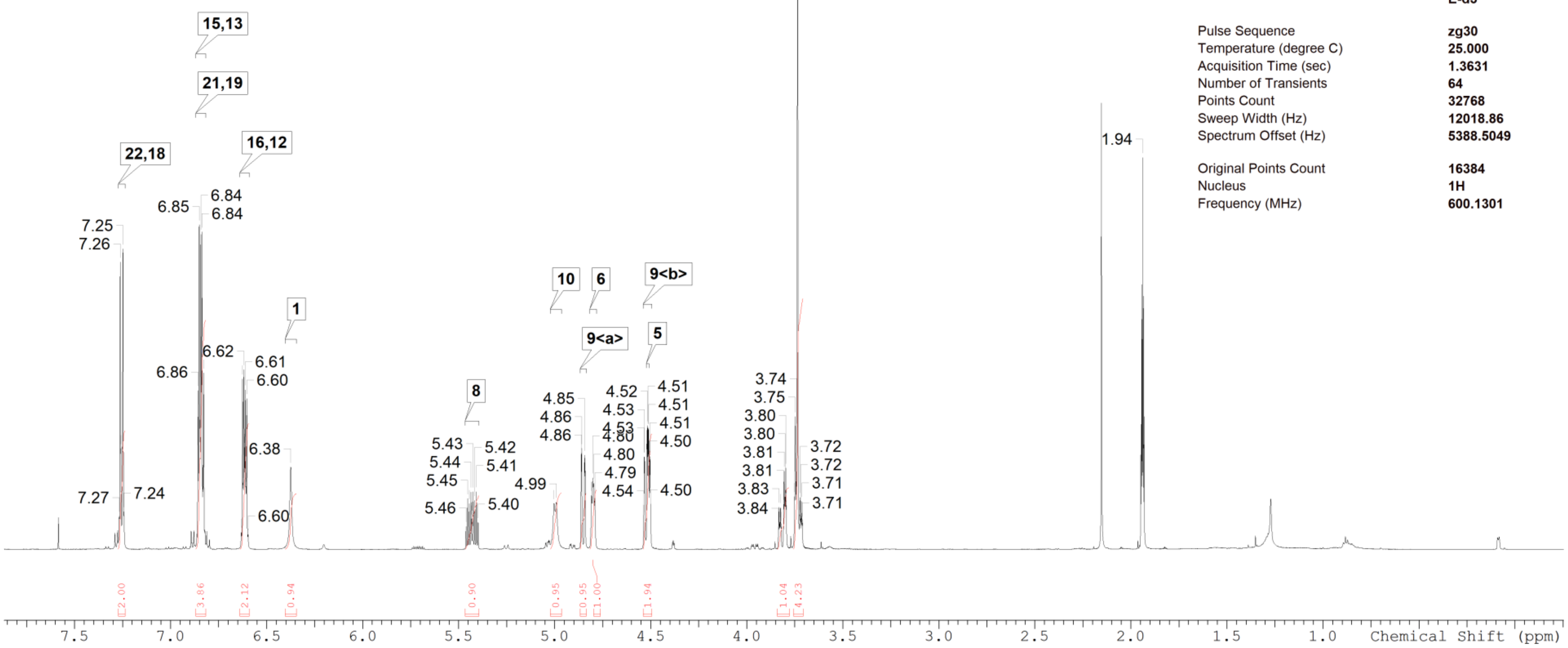

Figure S46. ^{1}H spectrum, structure, numbering and full assignment of Peak1 in acetonitrile-d$_3$ at 25 °C.

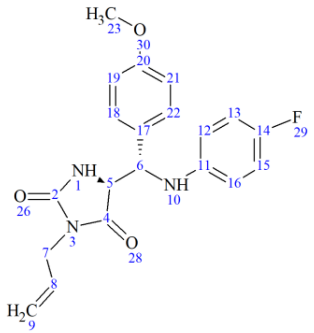

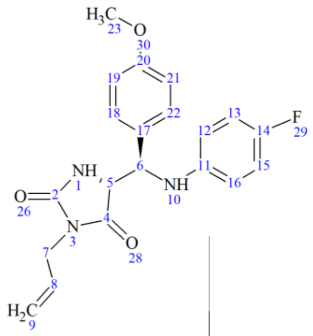

Shift1 (ppm)	C's	Type	J (Hz)	Atom1	Multiplet1
40.75	1.000	s	-	7	M03
55.98	1.000	s	-	23	M04
59.50	1.000	s	-	6	M05
62.12	1.000	s	-	5	M06
114.64	2.000	s	-	21, 19	M07
116.00	2.000	d	7.7	16, 12	M01
116.35	2.000	d	23.1	15, 13	M08
116.46	1.000	s	-	9	M02
129.65	1.000	s	-	17	M13
130.40	2.000	s	-	22, 18	M14
132.56	1.000	s	-	8	M15
144.41	1.000	d	2.2	11	M16
156.90	1.000	d	232.7	14	M19
157.65	1.000	s	-	2	M18
160.69	1.000	s	-	20	M20
172.49	1.000	s	-	4	M21

^{13}C NMR (151 MHz, ACETONITRILE-d$_3$): δ = 40.8 (7), 56.0 (23), 59.5 (6), 62.1 (5), 114.6 (21, 19), 116.0 (16, 12), 116.4 (15, 13), 116.5 (9), 129.6 (17), 130.4 (22, 18), 132.6 (8), 144.4 (11), 156.9 (14), 157.6 (2), 160.7 (20), 172.5 (4) ppm

Solvent	**ACETONITRILE -d3**
Pulse Sequence	**zgpg30**
Temperature (degree C)	**25.200**
Acquisition Time (sec)	**0.9110**
Number of Transients	**5760**
Points Count	**32768**
Sweep Width (Hz)	**35970.13**
Spectrum Offset (Hz)	**15251.7432**
Original Points Count	**32768**
Nucleus	**13C**
Frequency (MHz)	**150.9026**

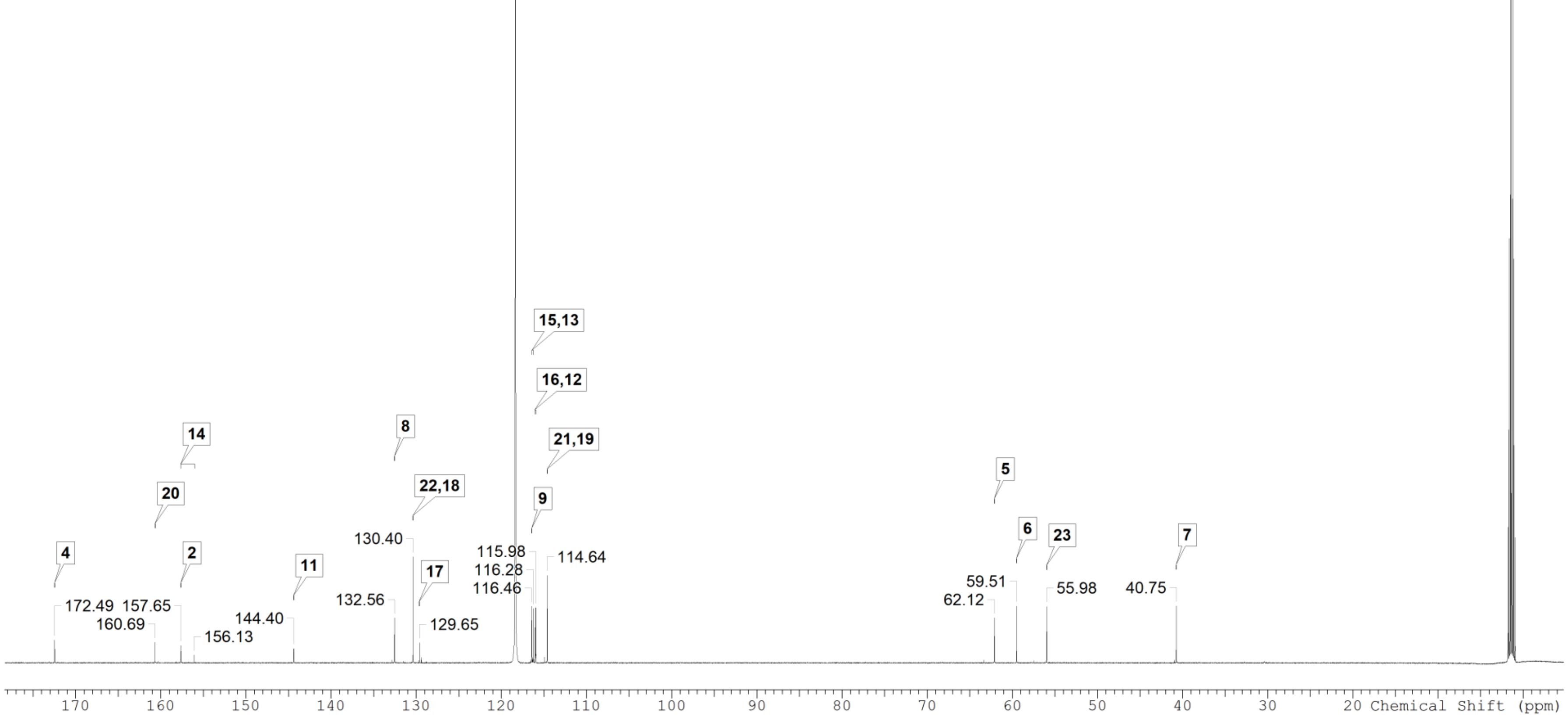

Figure S47. ^{13}C spectrum, structure, numbering and full assignment of Peak1 in acetonitrile-d$_3$ at 25 °C.

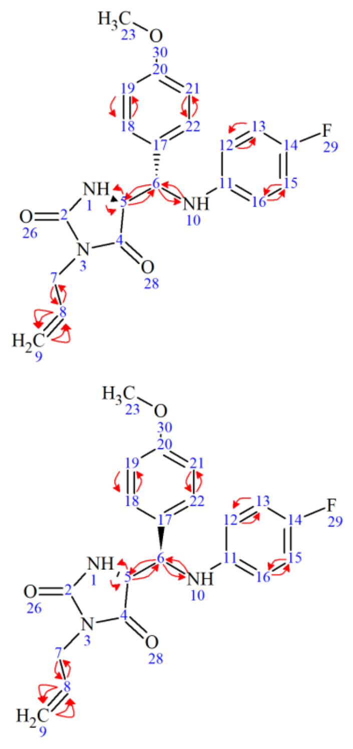

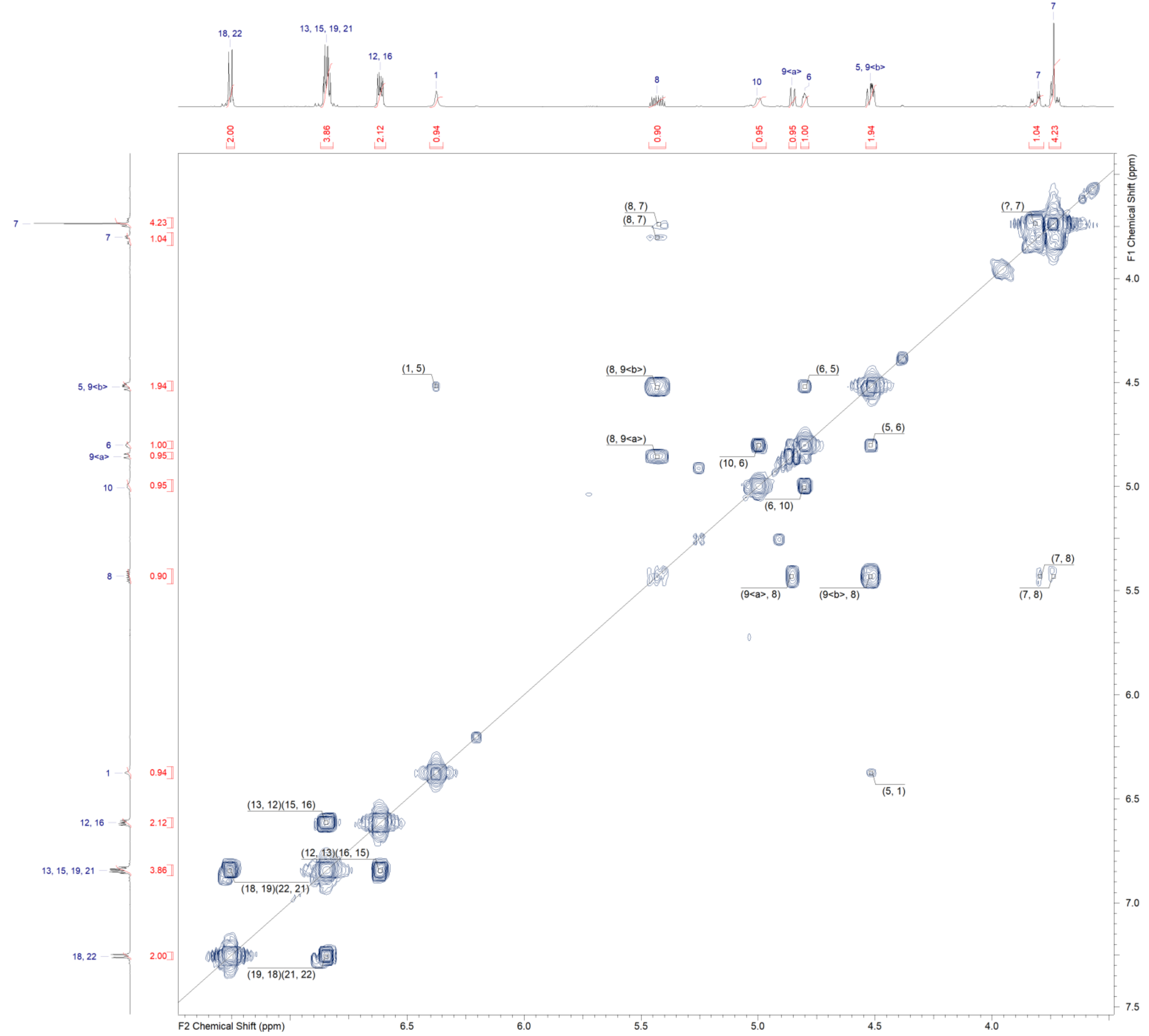

Acquisition Time (sec)	(0.1065, 0.0267)
Date	12 Jan 2022 17:49:34
Frequency (MHz)	(600.1300, 600.1300)
Nucleus	(1H, 1H)
Points Count	(2048, 2048)
Pulse Sequence	cosygpqf
Solvent	ACETONITRILE-d3
Sweep Width (Hz)	(9610.69, 9597.45)
Number of Transients	4
Spectrum Type	COSY
Temperature (degree C)	25.000

Figure S48. Fully assigned ^{1}H-^{1}H COSY spectrum of Peak1 in acetonitrile-d$_3$ at 25 °C.

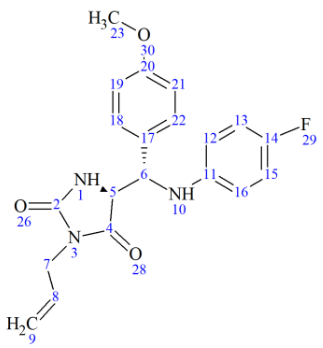

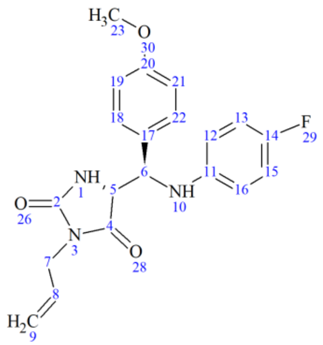

Acquisition Time (sec)	(0.1139, 0.0094)
Date	12 Jan 2022 18:30:02
Frequency (MHz)	(600.1300, 150.9026)
Nucleus	(1H, 13C)
Points Count	(1024, 1024)
Pulse Sequence	hsqcedetgpsisp.2
Solvent	ACETONITRILE-d3
Sweep Width (Hz)	(8984.02, 27138.29)
Number of Transients	16
Spectrum Type	HSQC-DEPT
Temperature (degree C)	25.100

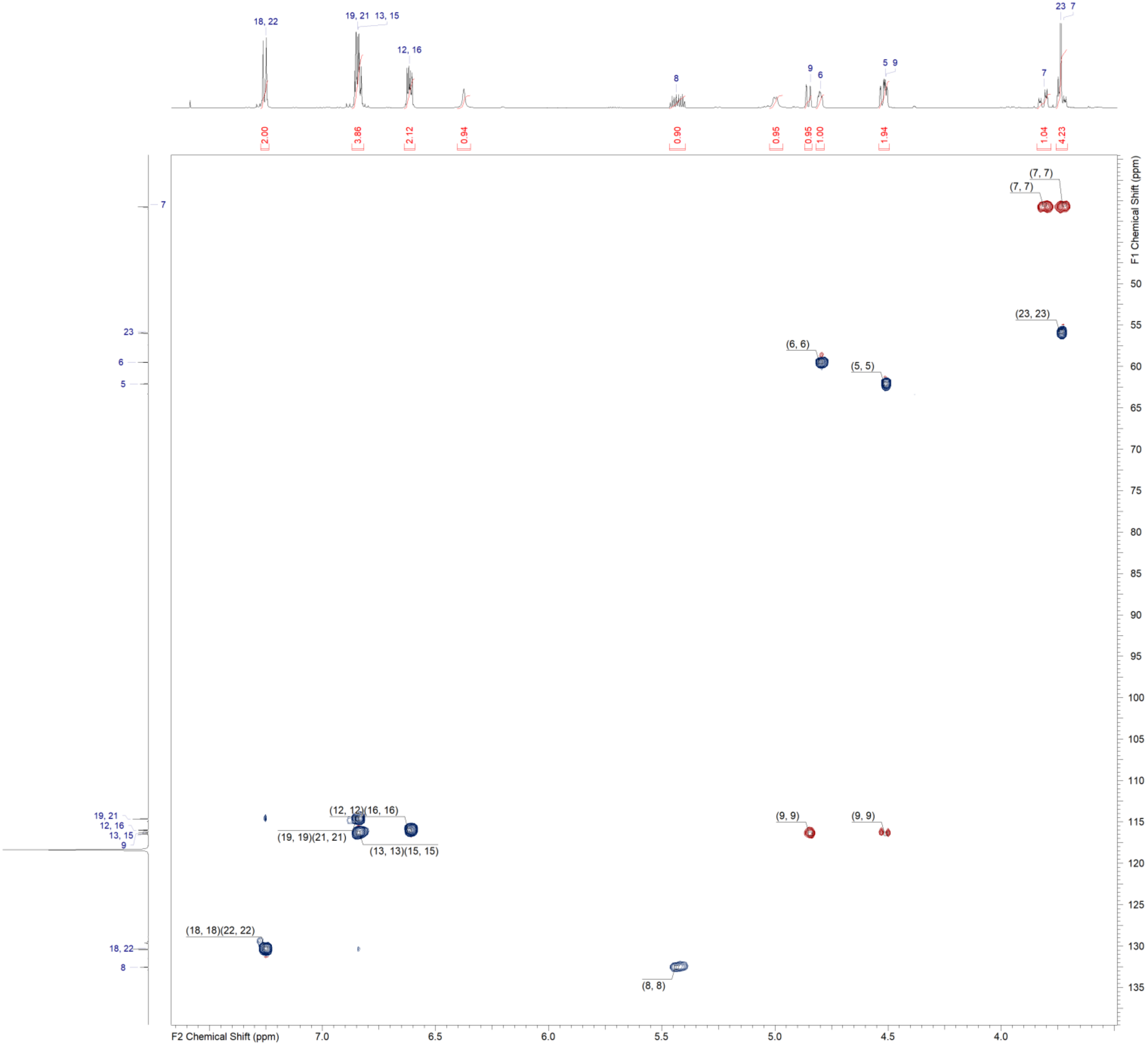

Figure S49. Fully assigned ^{1}H-^{13}C HSQC spectrum of Peak1 in acetonitrile-d_3 at 25 °C.

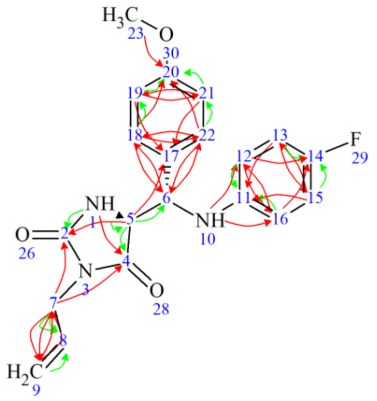

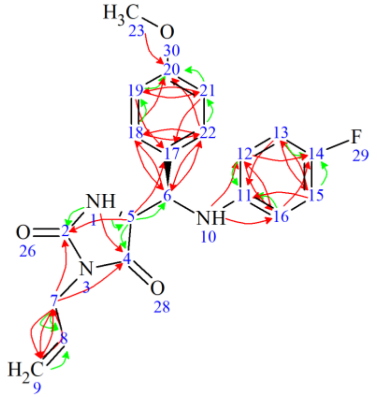

Acquisition Time (sec)	(0.1139, 0.0071)
Date	12 Jan 2022 17:49:42
Frequency (MHz)	(600.1300, 150.9027)
Nucleus	(1H, 13C)
Points Count	(2048, 1024)
Pulse Sequence	hmbcgplpndqf
Solvent	ACETONITRILE-d3
Sweep Width (Hz)	(8988.42, 36185.27)
Constant (Hz)	10.0
Number of Transients	32
Spectrum Type	HMBC
Temperature (degree C)	25.000

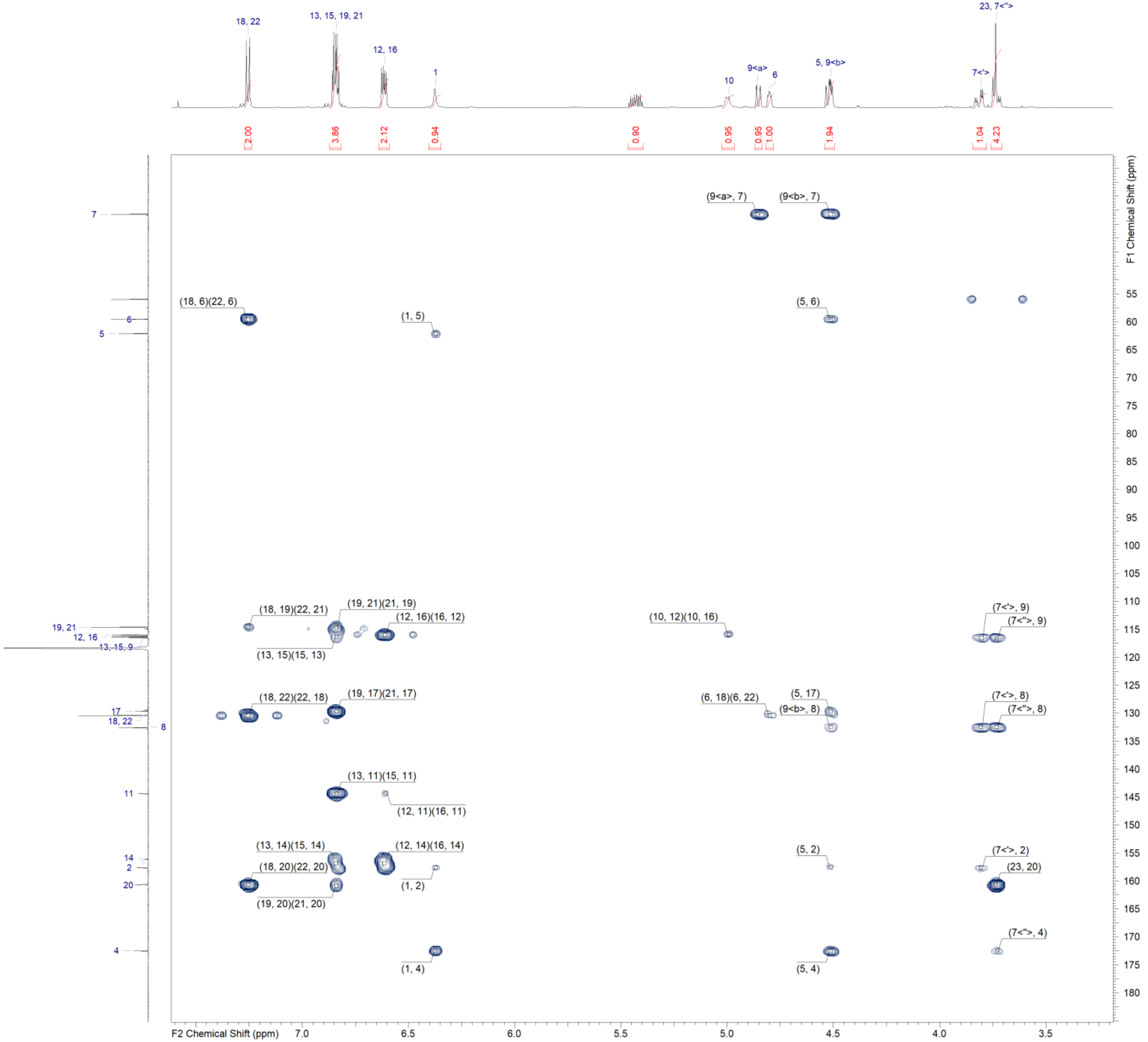

Figure S50. Fully assigned ^{1}H-^{13}C HMBC spectrum of Peak1 in acetonitrile-d$_3$ at 25 °C.

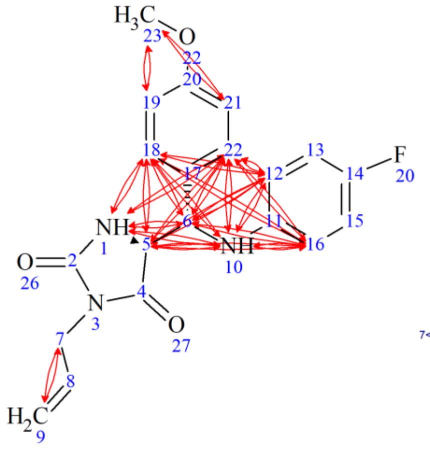

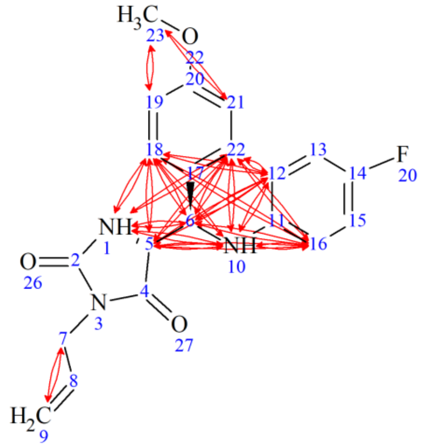

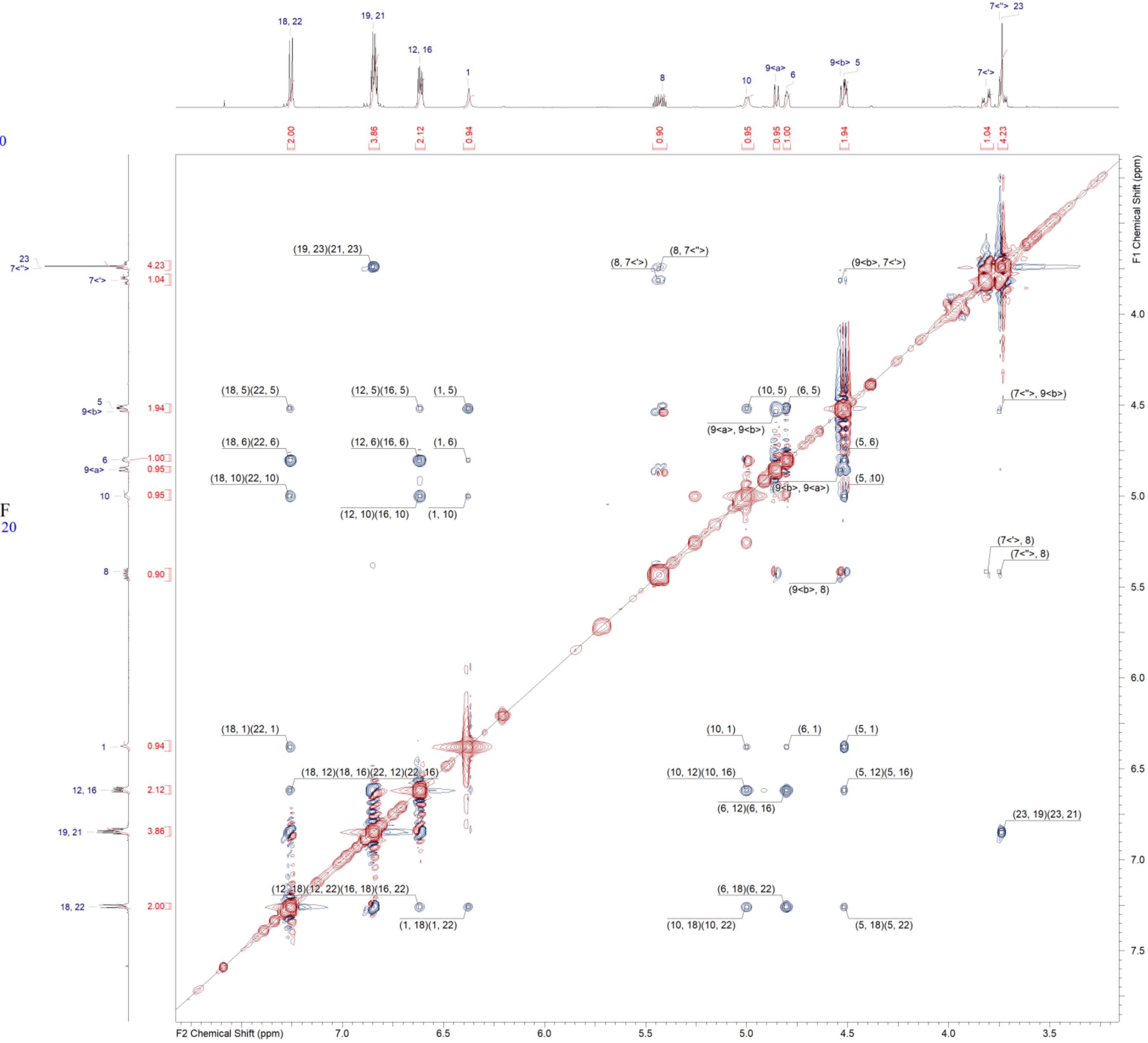

Acquisition Time (sec)	(0.1221, 0.0609)
Comment	MJ3-r5-pik1 13.1 mg
Frequency (MHz)	(600.1300, 600.1300)
Nucleus	(1H, 1H)
Origin	spect
Original Points Count	(1024, 512)
Points Count	(2048, 1024)
Pulse Sequence	noesygpphzs
Solvent	ACETONITRILE-d3
Sweep Width (Hz)	(8385.17, 8393.67)

Figure S51. Fully assigned ^{1}H-^{1}H NOESY spectrum of Peak1 in acetonitrile-d$_3$ at 25 °C

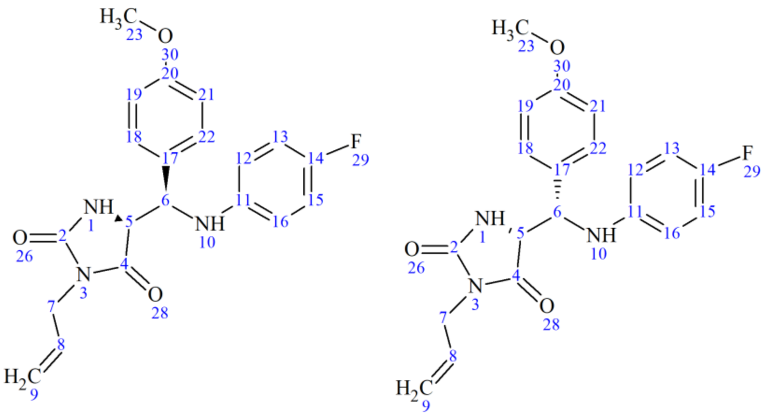

Shift1 (ppm)	H's	Type	J (Hz)	Atom1	Multiplet1
3.75	3.000	s	-	23	M03
3.93	1.000	ddt	16.5, 4.8, 1.8	7<">	M04
3.98	1.000	ddt	16.5, 4.8, 1.8	7<'>	M07
4.38	1.000	dd	3.3, 2.2	5	M05
4.91	1.000	dd	10.6, 3.3	6	M06
5.02	1.000	dq	17.3, 1.6	9<b>	M01
5.04	1.000	dq	10.5, 1.4	9<a>	M02
5.26	1.000	d	10.6	10	M08
5.71	1.000	ddt	17.1, 10.4, 5.0	8	M09
6.22	1.000	br s	-	1	M10
6.61	2.000	dd	8.8, 4.8	16, 12	M11
6.81	2.000	t	8.8	15, 13	M12
6.89	2.000	d	8.8	21, 19	M13
7.28	2.000	d	8.8	22, 18	M14

^{1}H NMR (600 MHz, ACETONITRILE-d_3): δ = 3.75 (s, 3 H, 23), 3.93 (ddt, J = 16.5, 4.8, 1.8 Hz, 1 H, 7<">), 3.98 (ddt, J = 16.5, 4.8, 1.8 Hz, 1 H, 7<'>), 4.38 (dd, J = 3.3, 2.2 Hz, 1 H, 5), 4.91 (dd, J = 10.6, 3.3 Hz, 1 H, 6), 5.02 (dq, J = 17.3, 1.6 Hz, 1 H, 9<b>), 5.04 (dq, J = 10.5, 1.4 Hz, 1 H, 9<a>), 5.26 (d, J = 10.6 Hz, 1 H, 10), 5.71 (ddt, J = 17.1, 10.4, 5.0 Hz, 1 H, 8), 6.22 (br s, 1 H, 1), 6.61 (dd, J = 8.8, 4.8 Hz, 2 H, 16, 12), 6.81 (t, J = 8.8 Hz, 2 H, 15, 13), 6.89 (d, J = 8.8 Hz, 2 H, 21, 19), 7.28 (d, J = 8.8 Hz, 2 H, 22, 18) ppm

Solvent	ACETONITRILE-d3
Pulse Sequence	zg30
Temperature (degree C)	25.000
Acquisition Time (sec)	1.3631
Number of Transients	64
Points Count	32768
Sweep Width (Hz)	12018.86
Spectrum Offset (Hz)	5388.3779
Original Points Count	16384
Nucleus	1H
Frequency (MHz)	600.1300

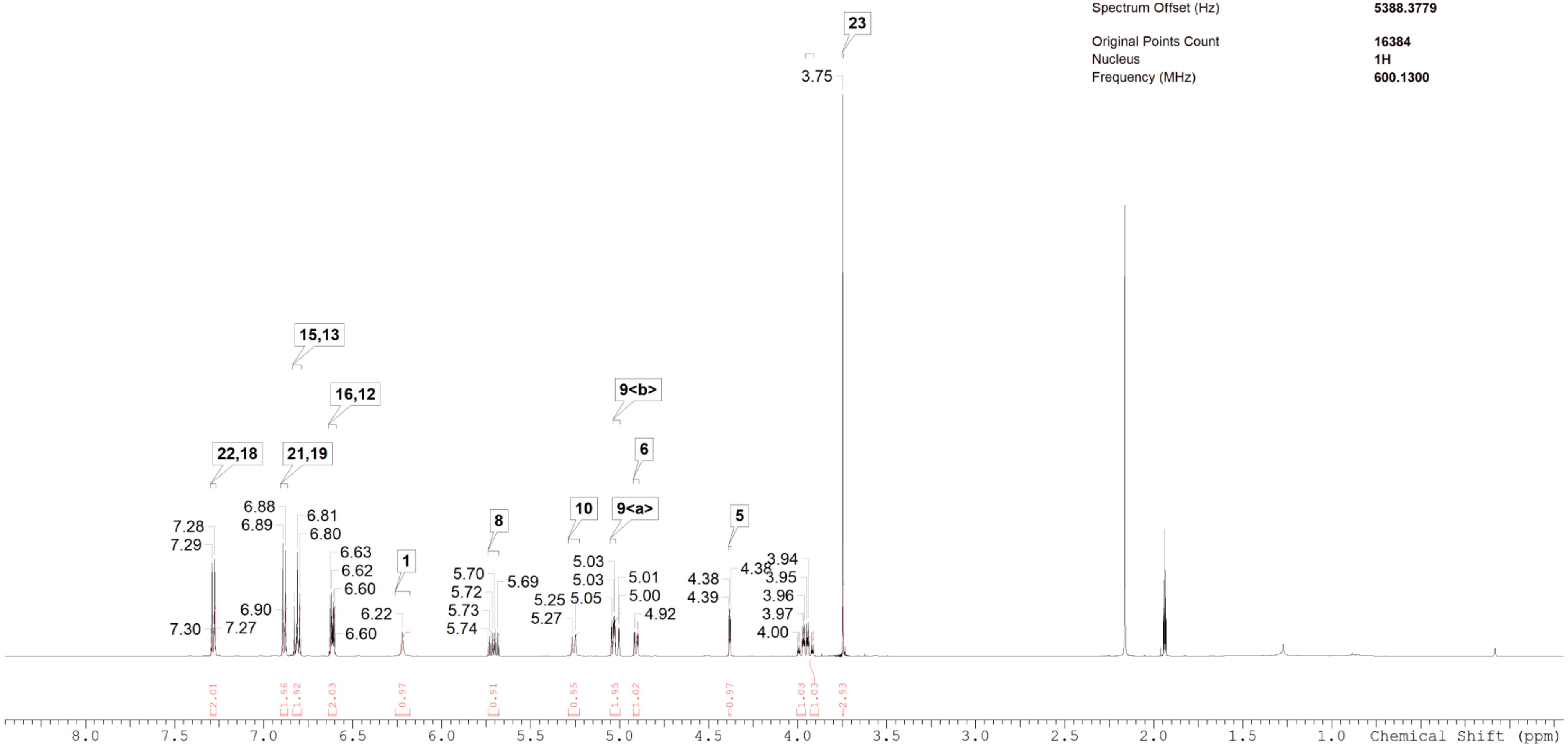

Figure S52. ^{1}H spectrum, structure, numbering and full assignment of Peak2 in acetonitrile-d_3 at 25 °C.

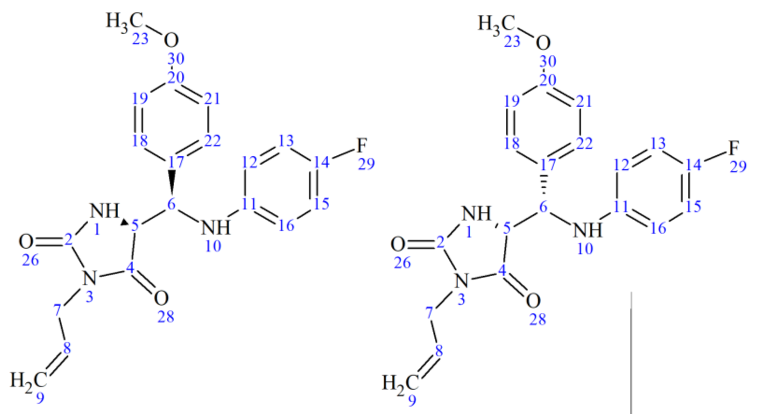

Atom	Exp. Shift (ppm)
7	41.0
23	56.0
6	57.4
5	63.3
21	115.0
19	115.0
16	116.0
12	116.0
15	116.3
13	116.3
9	116.6
22	129.4
18	129.4
17	131.5
8	132.9
11	144.4
14	156.7
2	158.2
20	160.3
4	173.2

^{13}C NMR (151 MHz, ACETONITRILE-d_3): δ = 41.0 (7), 56.0 (23), 57.4 (6), 63.3 (5), 115.0 (21, 19), 116.0 (16, 12), 116.3 (15, 13), 116.6 (9), 129.4 (22, 18), 131.5 (17), 132.9 (8), 144.4 (11), 156.7 (14), 158.2 (2), 160.3 (20), 173.2 (4) ppm

Solvent	ACETONITRILE-d3
Pulse Sequence	zgpg30
Temperature (degree C)	25.100
Acquisition Time (sec)	0.9110
Number of Transients	6400
Points Count	32768
Sweep Width (Hz)	35970.13
Spectrum Offset (Hz)	15250.5918
Original Points Count	32768
Nucleus	13C
Frequency (MHz)	150.9026

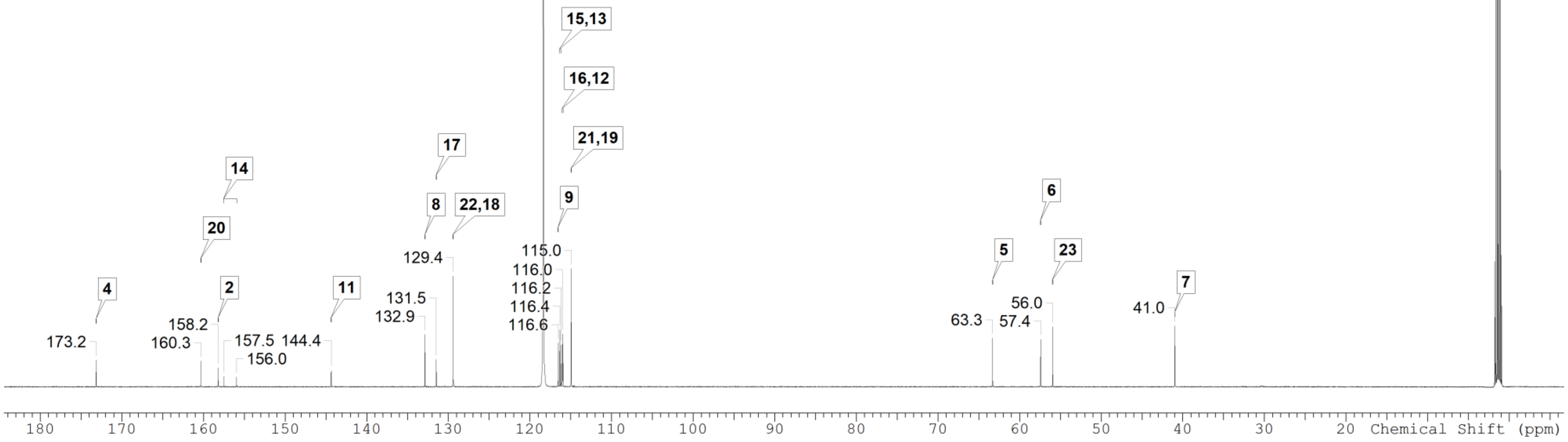

Figure S53. ^{13}C spectrum, structure, numbering and full assignment of Peak2 in acetonitrile-d3 at 25 °C.

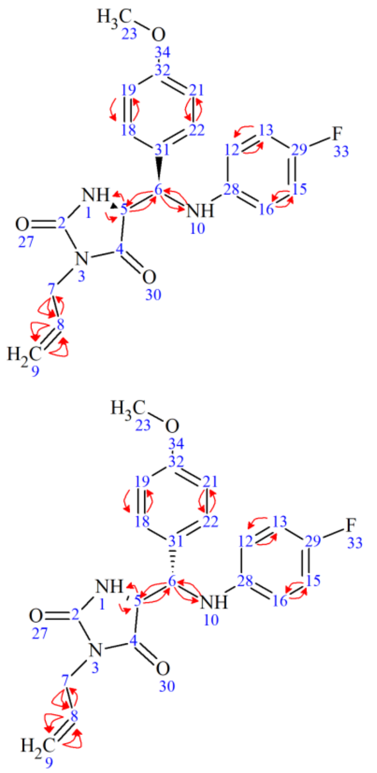

Acquisition Time (sec)	(0.1065, 0.0267)
Date	26 Jan 2022 23:28:28
Frequency (MHz)	(600.1301, 600.1301)
Nucleus	(1H, 1H)
Points Count	(2048, 2048)
Pulse Sequence	cosygpqf
Solvent	ACETONITRILE-d3
Sweep Width (Hz)	(9610.69, 9597.45)
Number of Transients	2
Spectrum Type	COSY
Temperature (degree C)	25.000

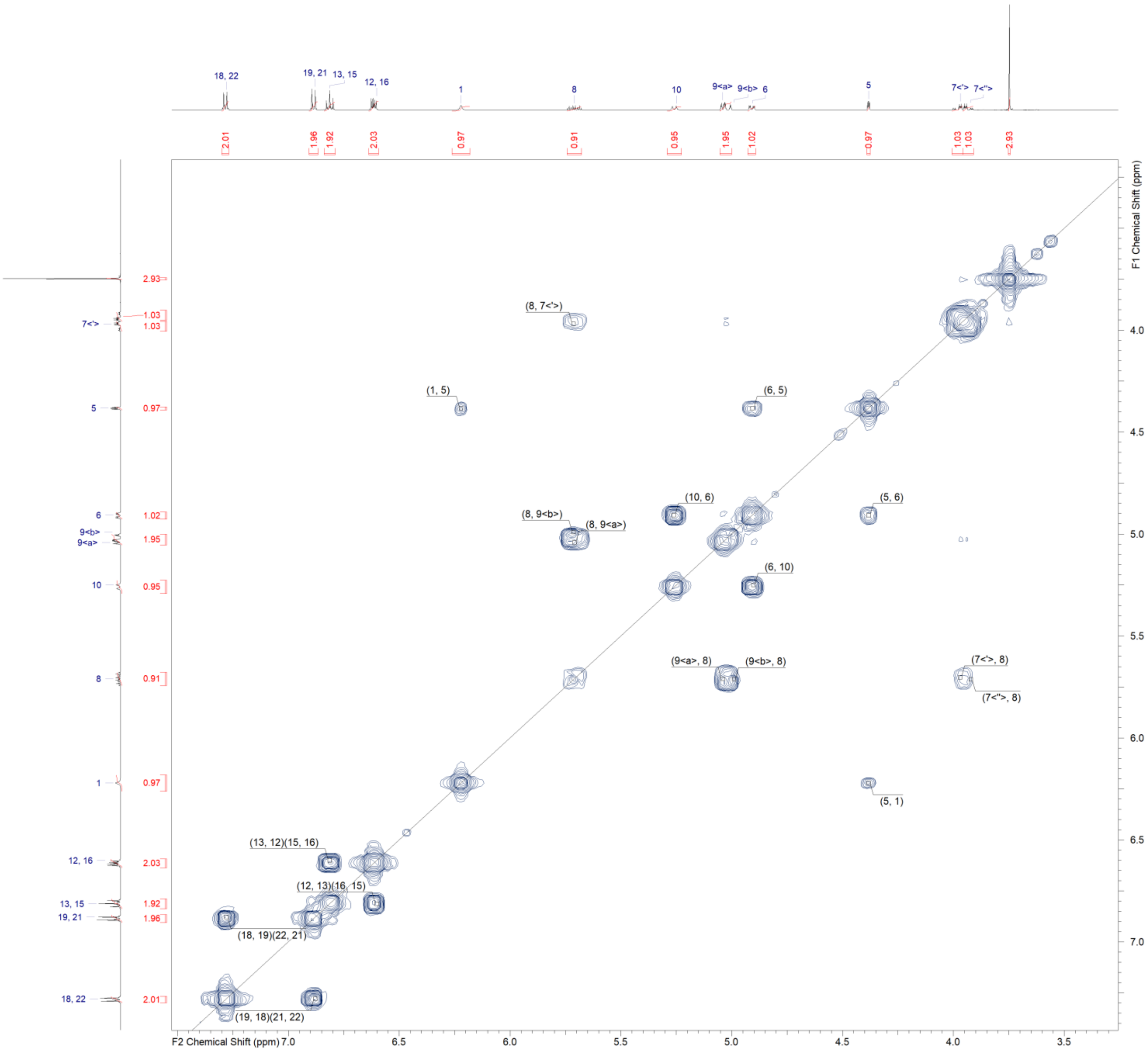

Figure S54. Fully assigned ^{1}H-^{1}H COSY spectrum of Peak2 in acetonitrile-d_3 at 25 °C.

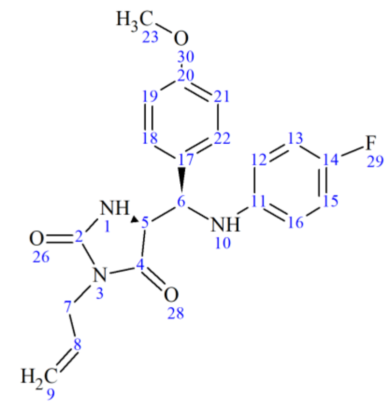

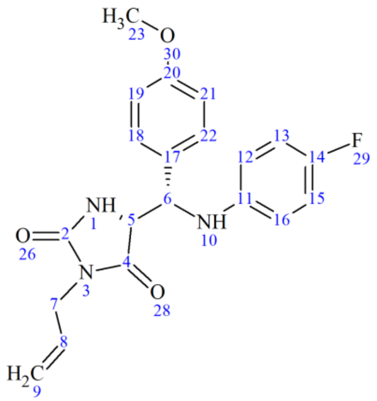

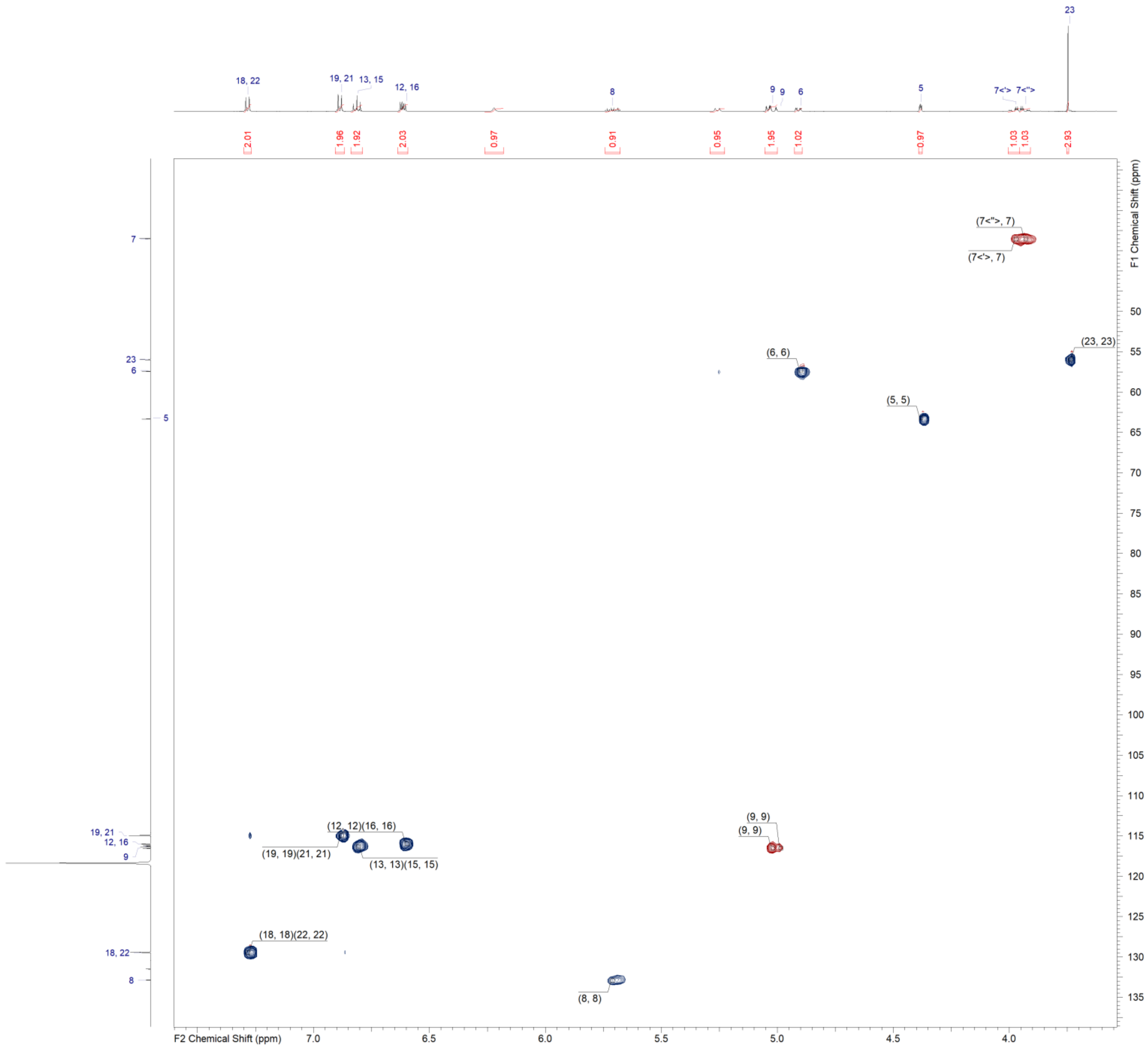

Acquisition Time (sec)	(0.1139, 0.0094)
Date	01 Feb 2022 21:21:48
Frequency (MHz)	(600.1300, 150.9027)
Nucleus	(1H, 13C)
Points Count	(1024, 1024)
Pulse Sequence	hsqcedetgpsisp.2
Solvent	ACETONITRILE-d3
Sweep Width (Hz)	(8984.02, 27138.29)
Number of Transients	16
Spectrum Type	HSQC-DEPT
Temperature (degree C)	25.100

Figure S55. Fully assigned ^{1}H-^{13}C HSQC spectrum of Peak2 in acetonitrile-d_3 at 25 °C.

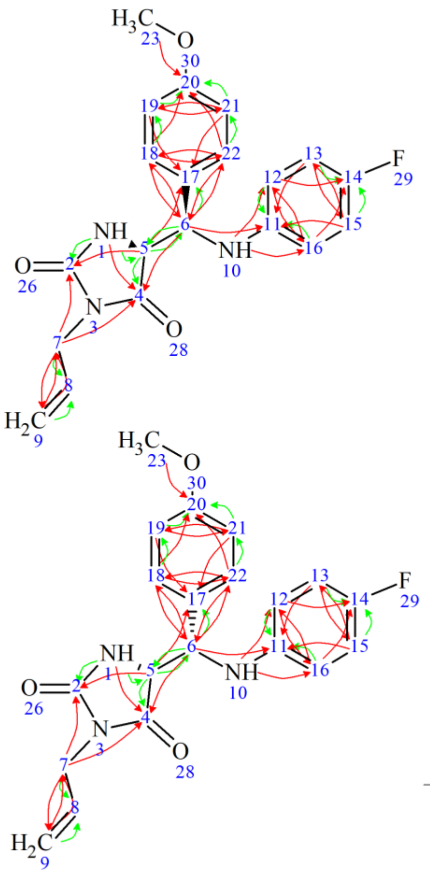

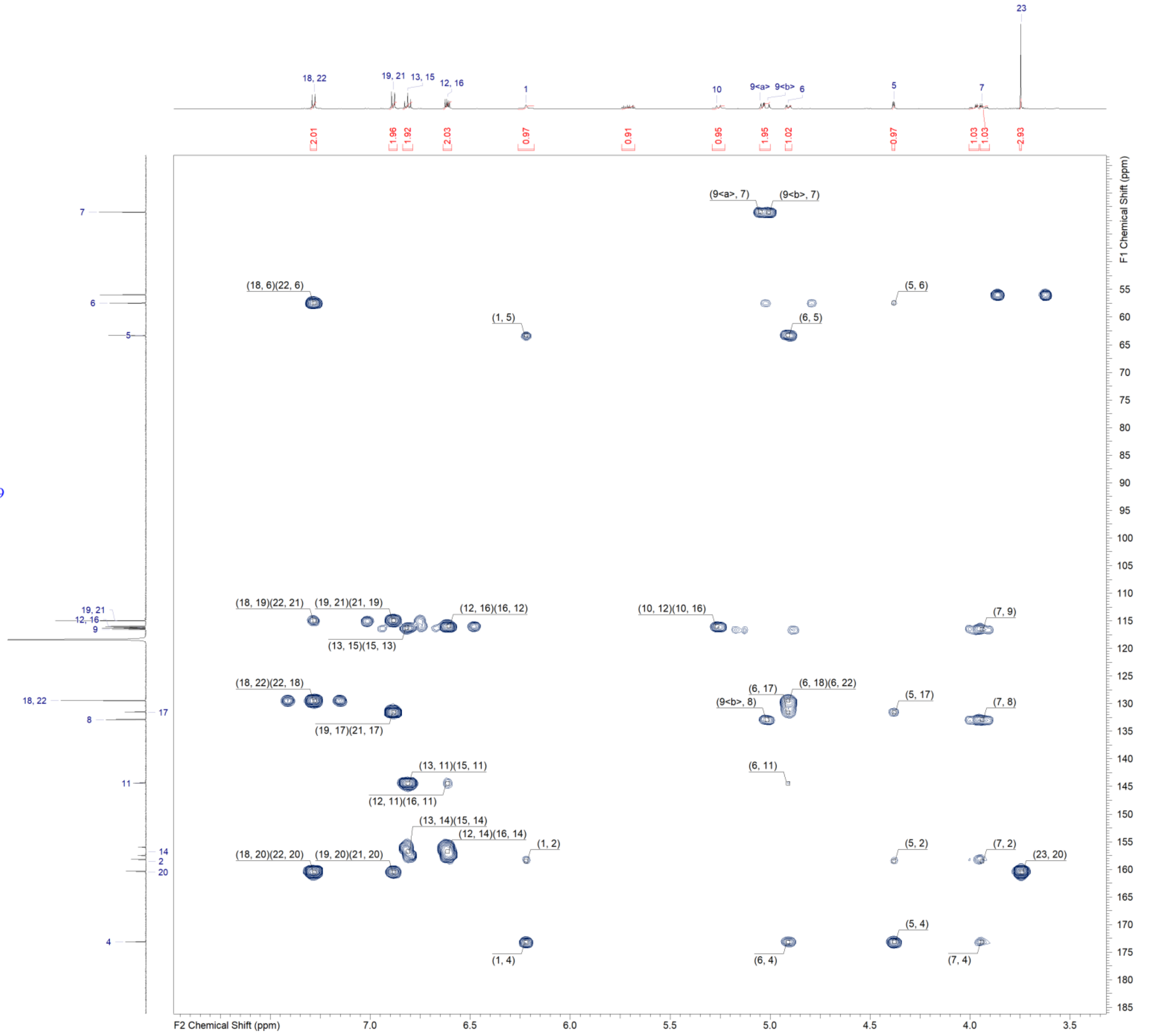

Acquisition Time (sec)	(0.1139, 0.0071)
Date	27 Jan 2022 05:14:42
Frequency (MHz)	(600.1300, 150.9026)
Nucleus	(1H, 13C)
Points Count	(2048, 1024)
Pulse Sequence	hmbcgplpndqf
Solvent	ACETONITRILE-d3
Sweep Width (Hz)	(8988.42, 36185.27)
Constant (Hz)	10.0
Number of Transients	32
Spectrum Type	HMBC
Temperature (degree C)	25.000

Figure S56. Fully assigned ^{1}H-^{13}C HMBC spectrum of Peak2 in acetonitrile-d₃ at 25 °C.

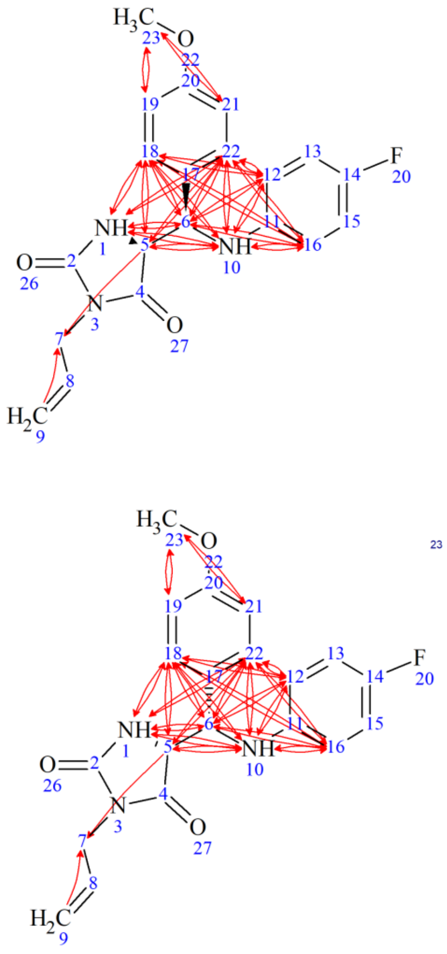

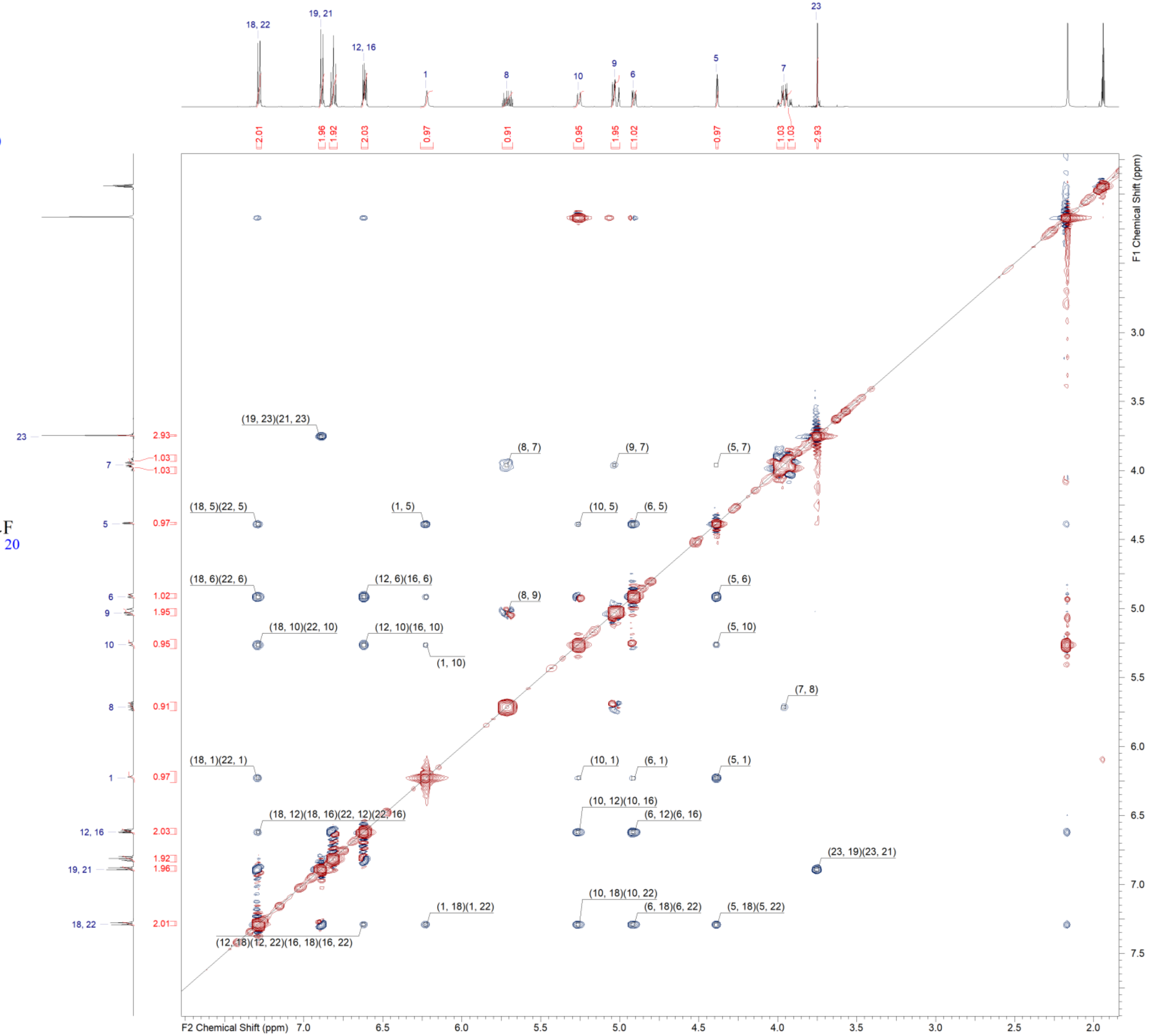

Acquisition Time (sec)	(0.1221, 0.0609)
Comment	MJ3-r5-pik2 acetonitrile 13.2 mg
Frequency (MHz)	(600.1301, 600.1301)
Nucleus	(1H, 1H)
Origin	spect
Original Points Count	(1024, 512)
Points Count	(2048, 1024)
Pulse Sequence	noesygpphzs
Solvent	ACETONITRILE-d3
Sweep Width (Hz)	(8385.17, 8393.67)

Figure S57. Fully assigned ^{1}H-^{1}H NOESY spectrum of Peak2 in acetonitrile-d_3 at 25 °C.

Table S1. Proton chemical shifts comparison for Peak1 and Peak2 in acetonitrile-d_3 at 25 °C, with major differences marked in red.

Proton	peak 1 δ/ppm	peak 2 δ/ppm	difference δ/ppm	absolute difference / ppm
7<">	3.731	3.932	-0.201	0.201
23	3.736	3.747	-0.011	0.011
7<'>	3.815	3.982	-0.167	0.167
5	4.516	4.383	0.133	0.133
9<b>	4.52	5.02	-0.5	0.5
6	4.8	4.91	-0.11	0.11
9<a>	4.853	5.038	-0.185	0.185
10	4.998	5.259	-0.261	0.261
8	5.431	5.71	-0.279	0.279
1	6.376	6.221	0.155	0.155
16, 12	6.615	6.615	0	0
15, 13	6.843	6.813	0.03	0.03
21, 19	6.845	6.886	-0.041	0.041
22, 18	7.255	7.284	-0.029	0.029

Table S2. Carbon chemical shifts comparison for Peak1 and Peak2 in acetonitrile-d_3 at 25 °C, with major differences marked in red.

Carbon	peak 1 δ/ppm	peak 2 δ/ppm	difference δ/ppm	absolute difference / ppm
7	40.8	41	-0.2	0.2
23	56	56	0	0
6	59.5	57.4	2.1	2.1
5	62.1	63.3	-1.2	1.2
21	114.6	115	-0.4	0.4
19	114.6	115	-0.4	0.4
16	116	116	0	0
12	116	116	0	0
15	116.4	116.3	0.1	0.1
13	116.4	116.3	0.1	0.1
9	116.5	116.6	-0.1	0.1
17	129.6	131.5	-1.9	1.9
22	130.4	129.4	1	1
18	130.4	129.4	1	1
8	132.6	132.9	-0.3	0.3
11	144.4	144.4	0	0
14	156.9	156.7	0.2	0.2
2	157.6	158.2	-0.6	0.6
20	160.7	160.3	0.4	0.4
4	172.5	173.2	-0.7	0.7

Table S3. Comparison of NOE interactions for Peak1 and Peak2 in acetonitrile-d_3 at 25 °C, with major differences marked in red, s=strong, m=medium, w=weak.

	5	1	7a	7b	8	9a	9b	6	10	12, 16	13, 15	18, 22	19, 21	23	**Peak 1**
5	●	s						s	m	w		w			
1	s	●						w	w			m			
7a	w		●		m		w								
7b				●	m		w								
8			m		●										
9a			w			●	s								
9b							●								
6	s	w						●		s		s			
10	w	w							●	s		s			
12, 16								s	s	●		m			
13, 15											●				
18, 22	m	m						s	s	m		●			
19, 21													●	s	
23													s	●	
**Peak 2**															

Table S4. Comparison of coupling constants for Peak1 and Peak2 in acetonitrile-d_3 at 25 °C, with major differences marked in red.

	**Peak 1**	**Peak 2**
**Atom**	**^xJ/Hz**	**^xJ/Hz**
**1NH, 5**	1.8	2.2
**5, 6**	3.7	3.3
**7a, 7b**	16.1	16.5
**7a, 8**	4.9	5.0
**7a, 9a**	1.5	1.4
**7a, 9b**	1.8	1.6
**7b, 8**	4.9	5.0
**7b, 9a**	1.5	1.4
**7b, 9b**	1.8	1.6
**8, 9a**	10.5	10.4
**8, 9b**	17.2	17.2
**9a, 9b**	1.5	1.4
**6, 10NH**	6.4	10.6
**12(16), 13(15)**	8.8	8.8
**18(22), 19(21)**	8.8	8.8

3. HPLC chromatograms

Chromatographic conditions for compounds (±)-**2a**, (±)-**2b** and (±)-**3**

Column: Symmetry C18 (150 × 4.6 mm, 5 µm, Waters, Milford, MA, USA)

Mobile phase A: Water

Mobile phase B: Acetonitrile

Method:

t/min	%MP A	%MP B
0	70	30
15	30	70
18	30	70
18.01	70	30
21	70	30

Flow: 1 mL/min

Detection: 254 nm

Column temperature: 30°C

Injection volume: 20 µL

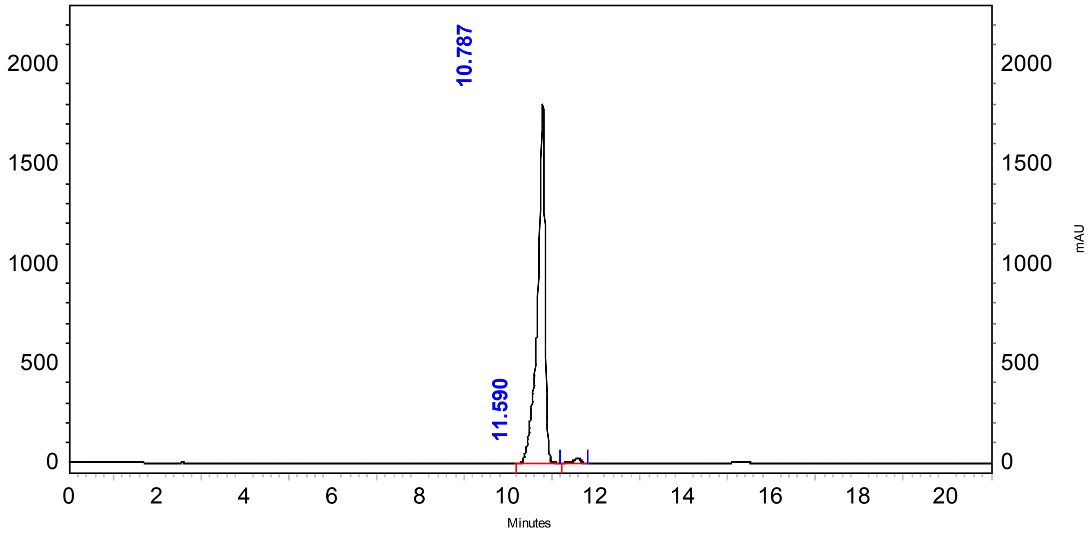

Compound	RT (min)	Area%
(±)-**2a**	10.787	98.67

Figure S58. RP-HPLC chromatogram of compound **2a**.

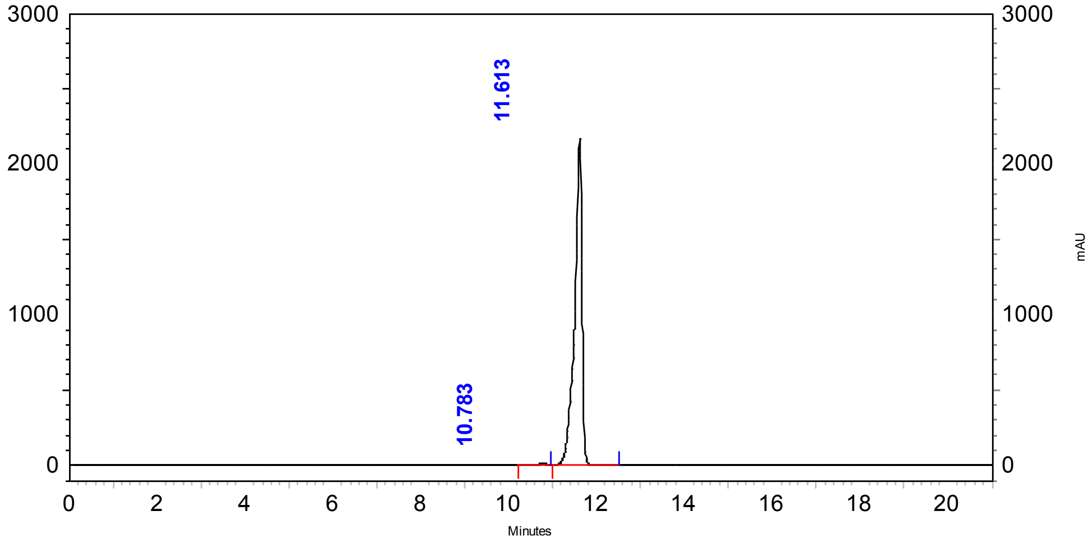

Peak No.	RT (min)	Area%
(±)-**2b**	11.613	99.40

Figure S59. RP-HPLC chromatogram of compound **2b**.

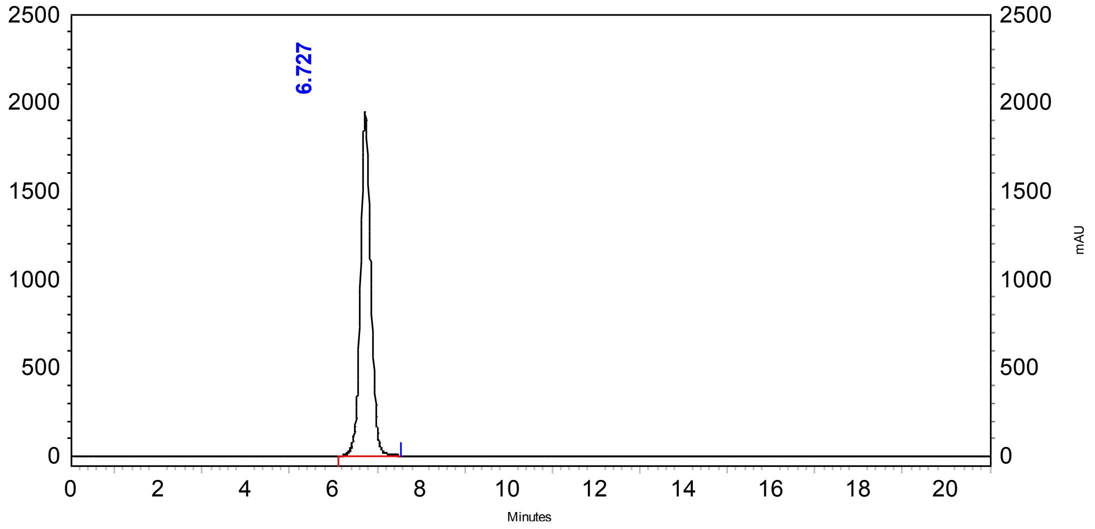

Peak No.	RT (min)	Area%
(±)-**3**	6.727	100

Figure S60. RP-HPLC chromatogram of compound **3**.

Chromatographic conditions for (±)-*trans*-β-lactam ureas **4a-i**

**Column:**	Synergi Polar-RP 80A (150 mm × 4.6 mm, 4 μm, Phenomenex, Torrance, CA, USA)		
**Mobile phase A:**	Water + 0.1% trifluoroacetic acid		
**Mobile phase B:**	Acetonitrile		
**Method:**	t/min	%MP A	%MP B
	0	50	50
	10	0	100
	13	0	100
	13.01	50	50
	17	50	50
**Flow:**	1 mL/min		
**Detection:**	254 nm		
**Column temperature:**	30 °C		
**Injection volume:**	20 μL		

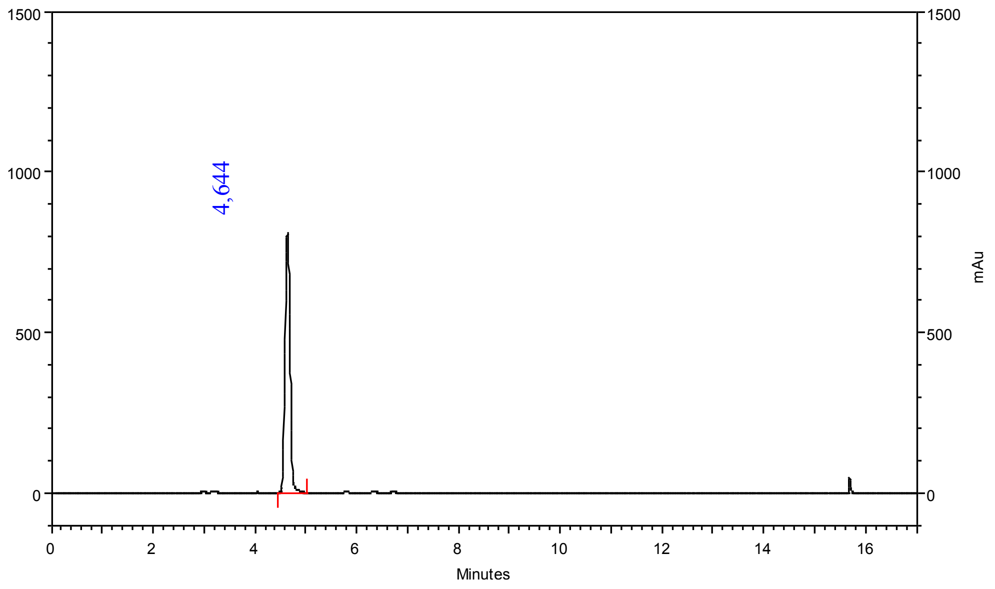

Peak No.	RT (min)	Area%
(±)-**4a**	4.644	100

Figure S61. RP-HPLC chromatogram of compound **4a**.

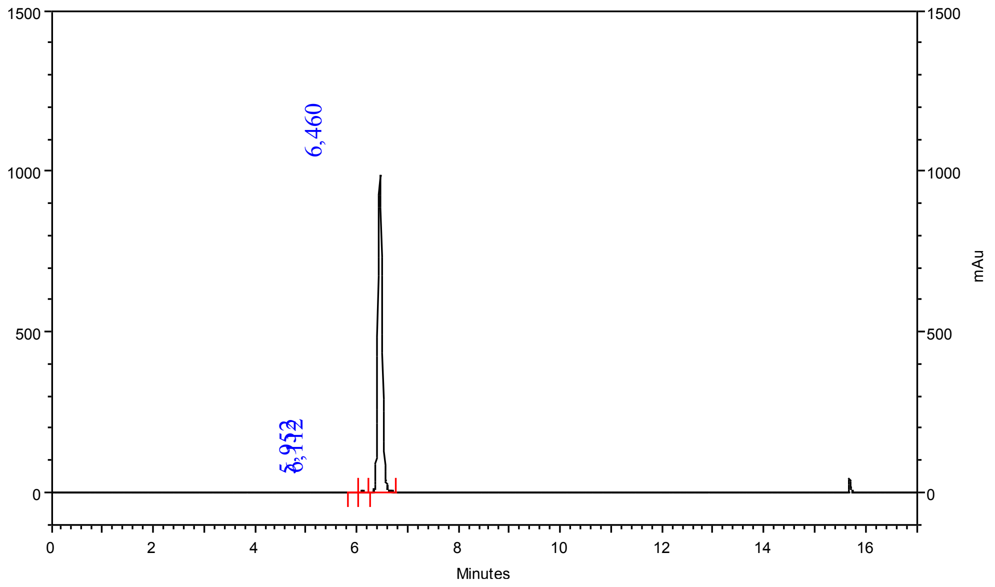

Peak No.	RT (min)	Area%
(±)-**4b**	6.460	99.76

Figure S62. RP-HPLC chromatogram of compound **4b**.

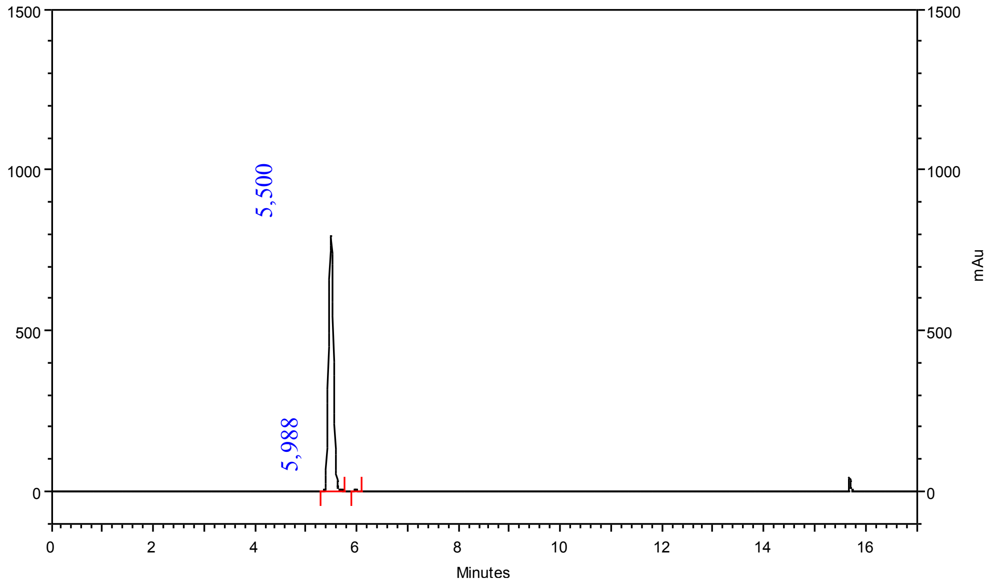

Peak No.	RT (min)	Area%
(±)-**4c**	5.500	99.88

Figure S63. RP-HPLC chromatogram of compound **4c**.

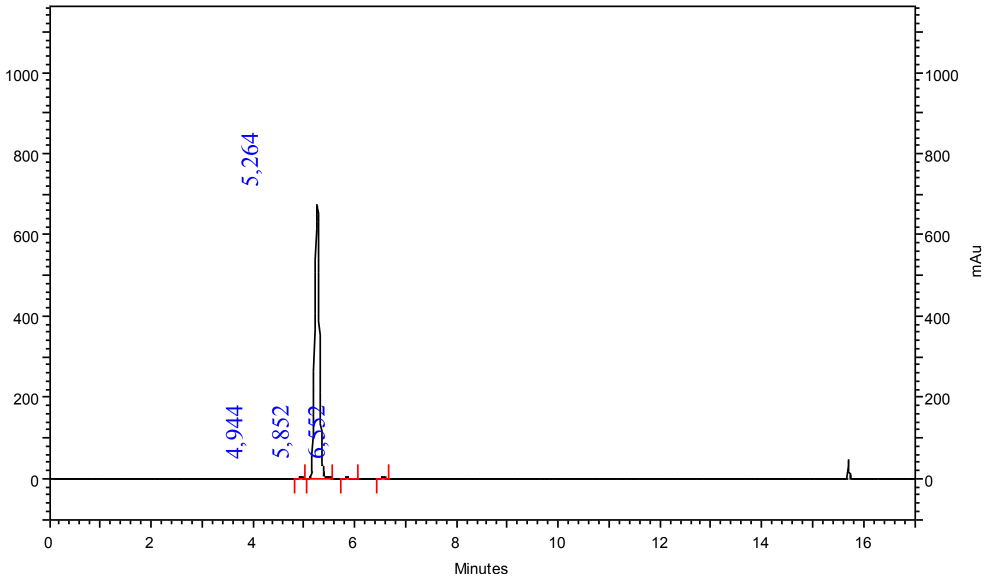

Peak No.	RT (min)	Area%
(±)-**4d**	5.264	99.49

Figure S64. RP-HPLC chromatogram of compound **4d**.

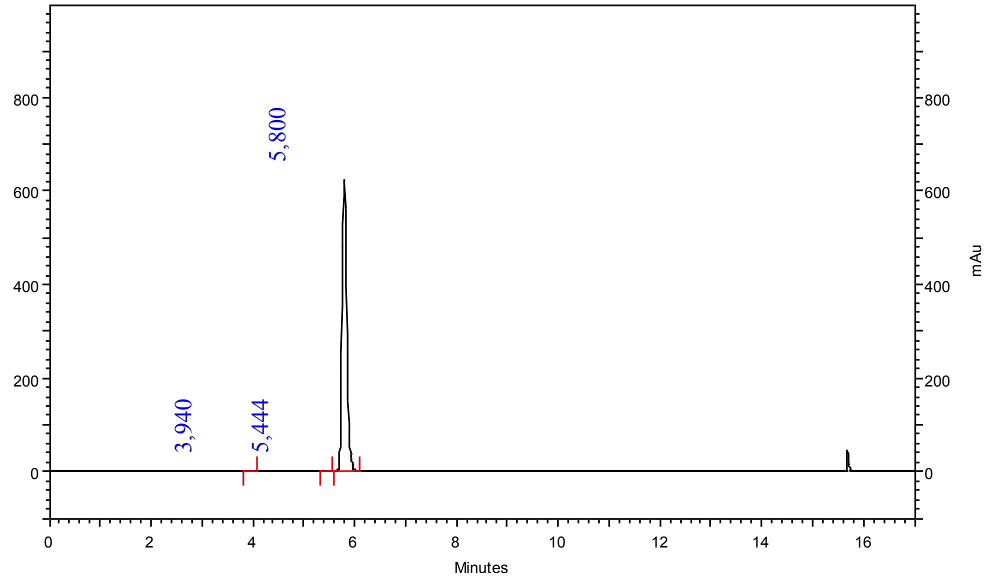

Peak No.	RT (min)	Area%
(±)-**4e**	5.800	99.43

Figure S65. RP-HPLC chromatogram of compound **4e**.

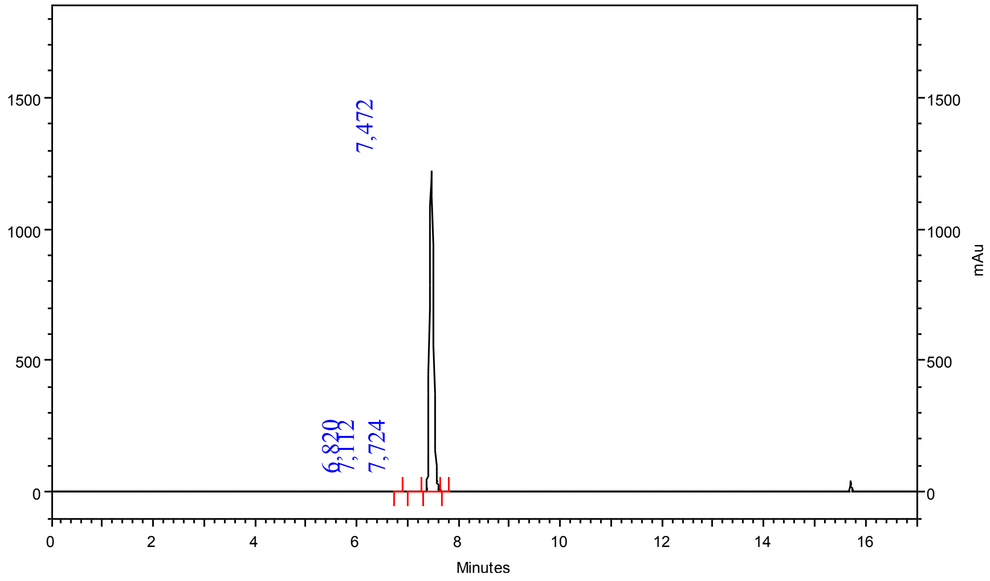

Peak No.	RT (min)	Area%
(±)-**4f**	7.472	96.65

Figure S66. RP-HPLC chromatogram of compound **4f**.

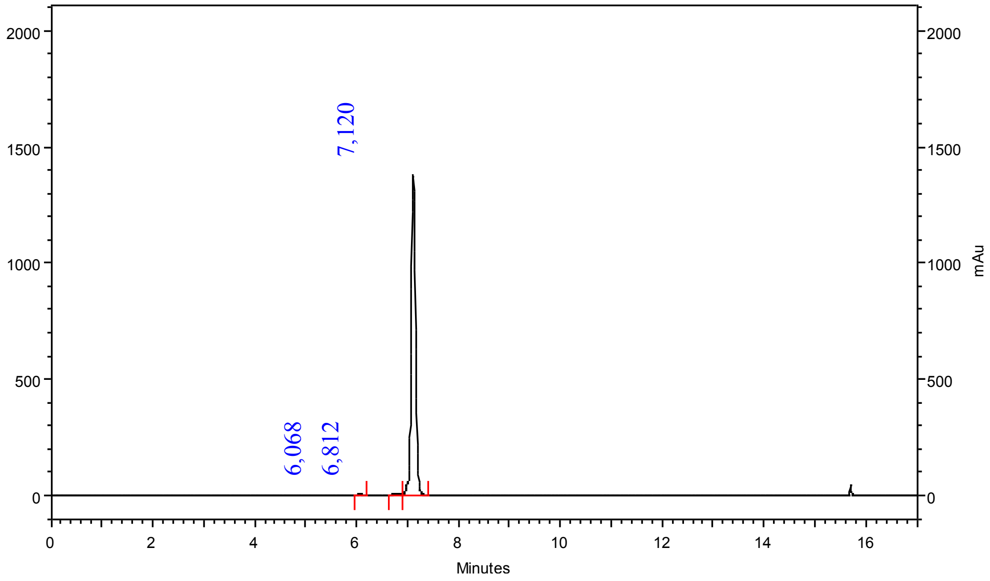

Peak No.	RT (min)	Area%
(±)-**4g**	7.120	99.15

Figure S67. RP-HPLC chromatogram of compound **4g**.

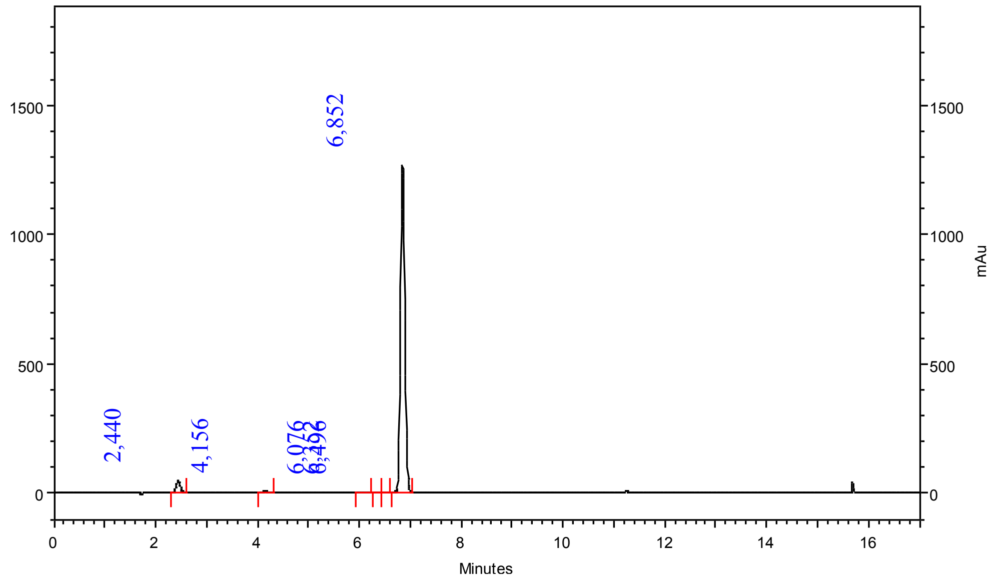

Peak No.	RT (min)	Area%
(±)-**4h**	6.852	95.06

Figure S68. RP-HPLC chromatogram of compound **4h**.

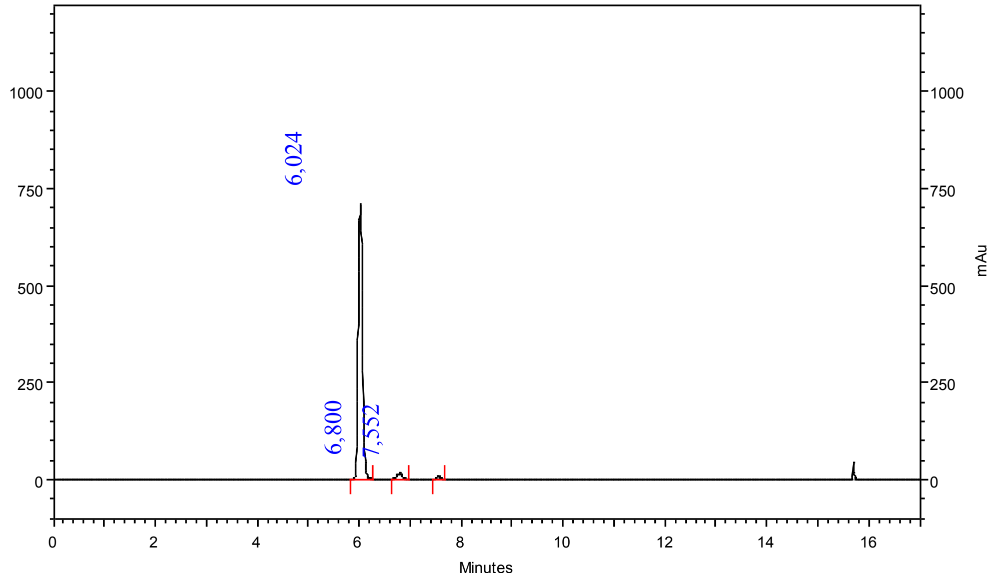

Peak No.	RT (min)	Area%
(±)-**4i**	6.024	96.70

Figure S69. RP-HPLC chromatogram of compound **4i**.

Chromatographic conditions for *syn*/*anti*-3,5-disubstituted hydantoins **5a-i**

**Column:**	Zorbax Extend-C18 column (250 × 4.6 mm, 5 µm, Agilent Technologies, Milford, MA, USA).
**Mobile phase A:**	Water
**Mobile phase B:**	Acetonitrile
**Flow:**	0.8 mL/min
**Detection:**	254 nm
**Column temperature:**	30 °C
**Injection volume:**	10 µL

**Method A** (for **5a**, **5d**, **5e**):	t/min	%MP A	%MP B
	0	65	35
	60	52	48
	63	52	48
	63.01	65	35
	67	65	35

**Method B** (for **5c**, **5h**, **5i**):	t/min	%MP A	%MP B
	0	65	35
	60	40	60
	63	40	60
	63.01	65	35
	67	65	35

**Method B** (for **5b**, **5f**, **5g**):	t/min	%MP A	%MP B
	0	55	45
	60	42	58
	63	42	58
	63.01	55	45
	67	55	45

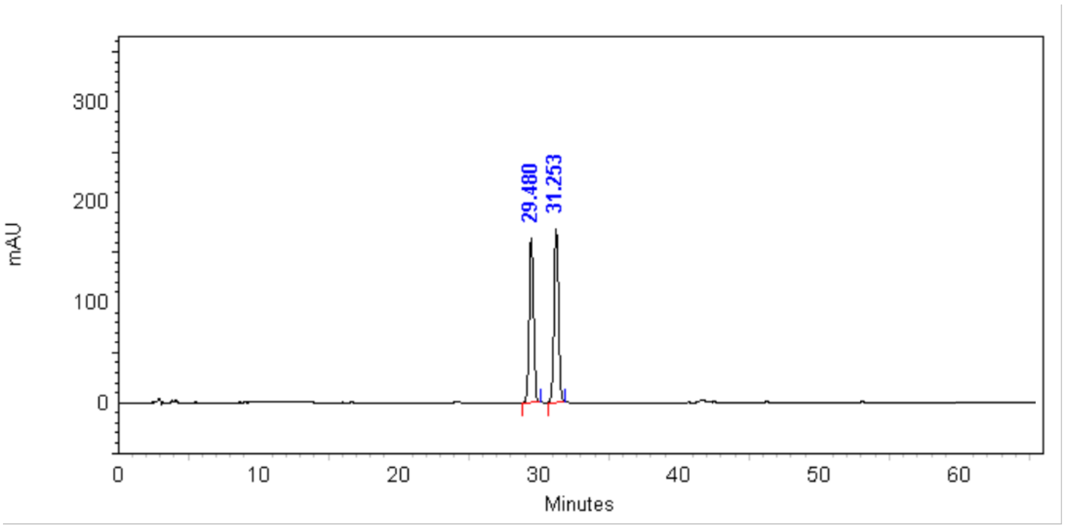

Peak No.	RT (min)	Area%
anti-**5a**	29.480	47.5
syn-**5a**	31.253	52.5

Figure S70. RP-HPLC chromatogram of compound *syn*/*anti*-**5a**.

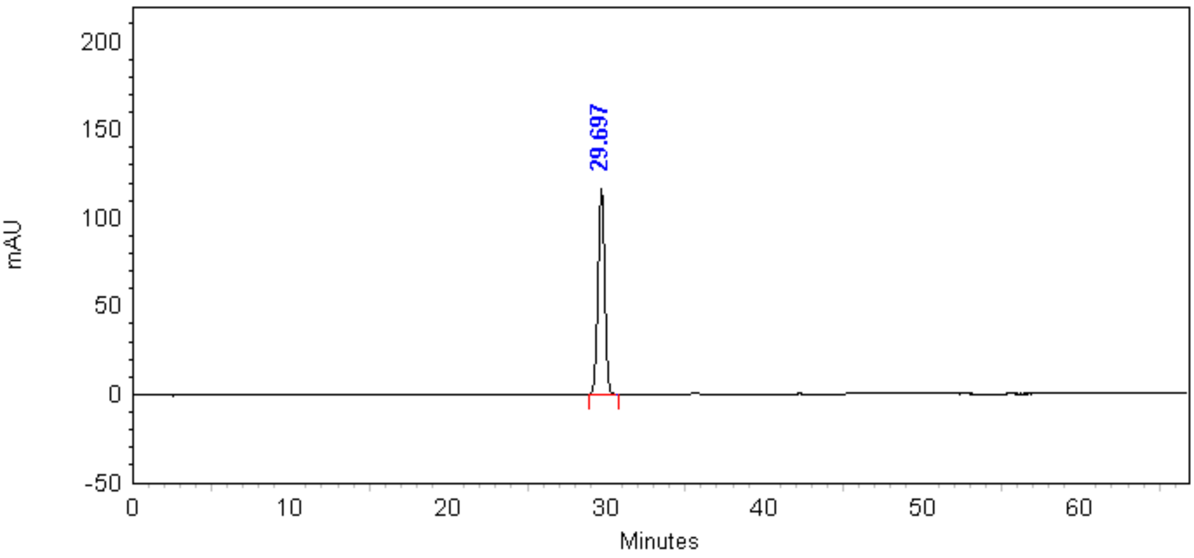

Peak No.	RT (min)	Area%
anti-**5a**	29.697	100

Figure S71. RP-HPLC chromatogram of compound *anti*-**5a**.

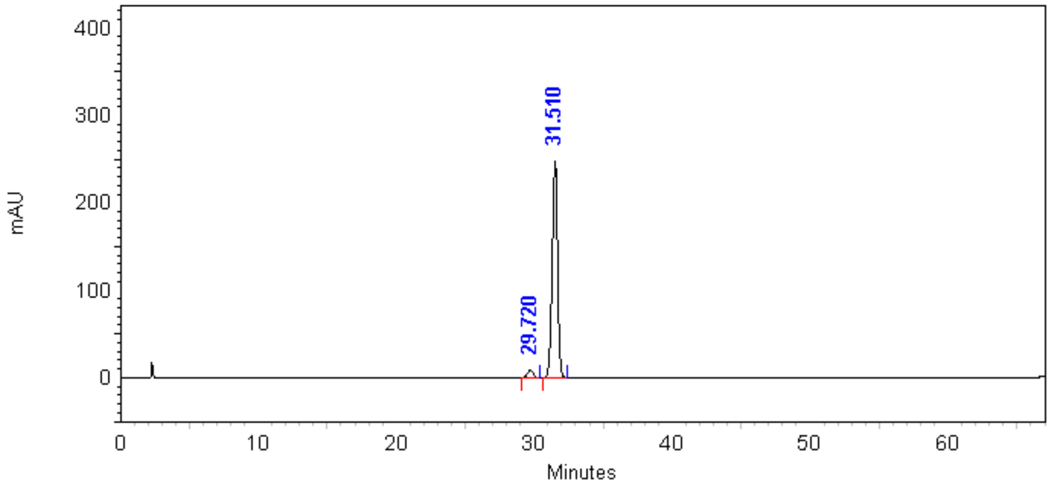

Peak No.	RT (min)	Area%
syn-**5a**	31.510	96.8

Figure S72. RP-HPLC chromatogram of compound *syn*-**5a**.

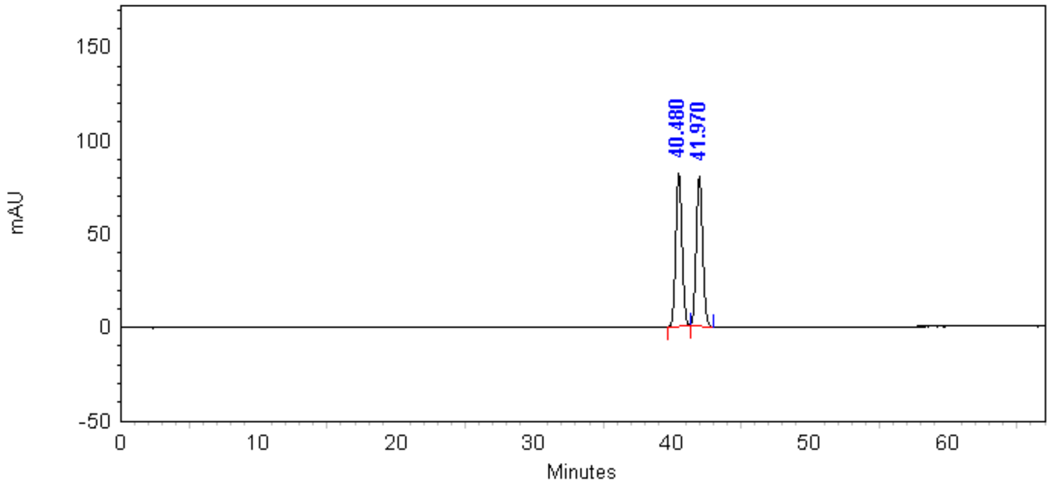

Peak No.	RT (min)	Area%
anti-**5b**	40.480	49.9
syn-**5b**	41.970	50.1

Figure S73. RP-HPLC chromatogram of compound *syn*/*anti*-**5b**.

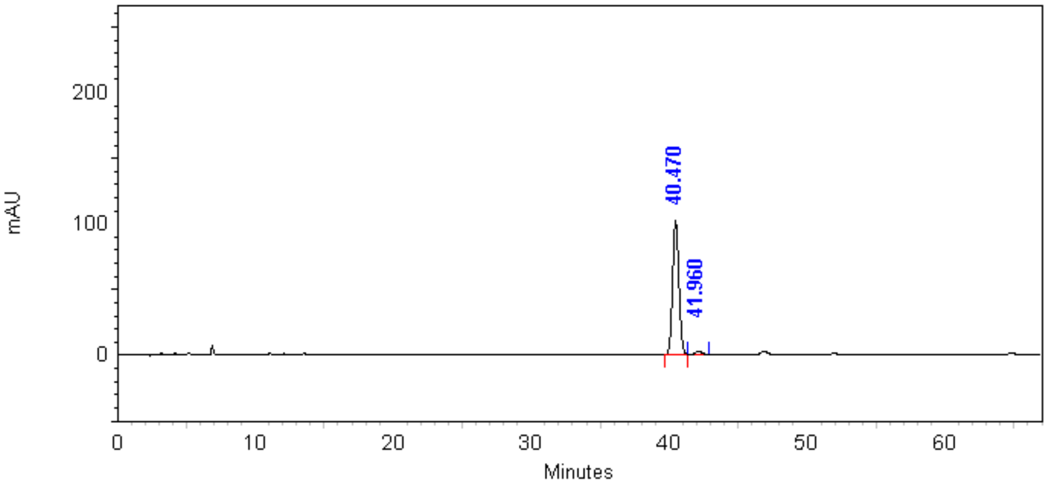

Peak No.	RT (min)	Area%
anti-**5b**	40.470	98.2

Figure S74. RP-HPLC chromatogram of compound *anti*-**5b**.

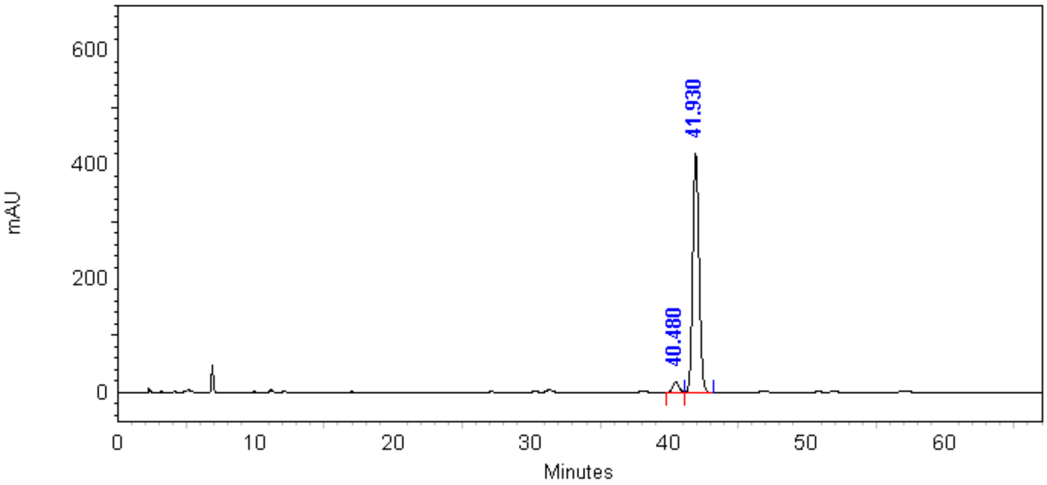

Peak No.	RT (min)	Area%
anti-**5b**	41.930	96.2

Figure S75. RP-HPLC chromatogram of compound *syn*-**5b**.

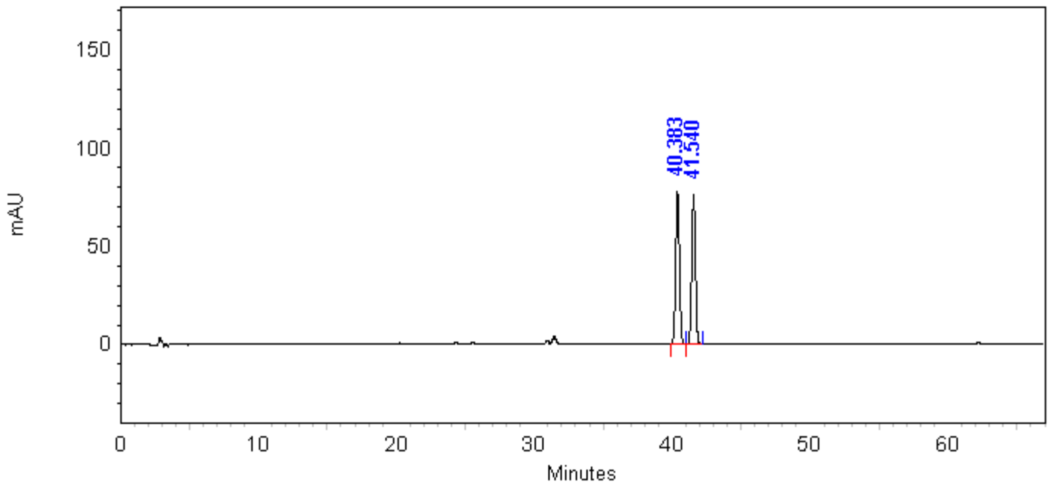

Peak No.	RT (min)	Area%
anti-**5c**	41.167	48.2
syn-**5c**	42.323	51.8

Figure S76. RP-HPLC chromatogram of compound *syn*/*anti*-**5c**.

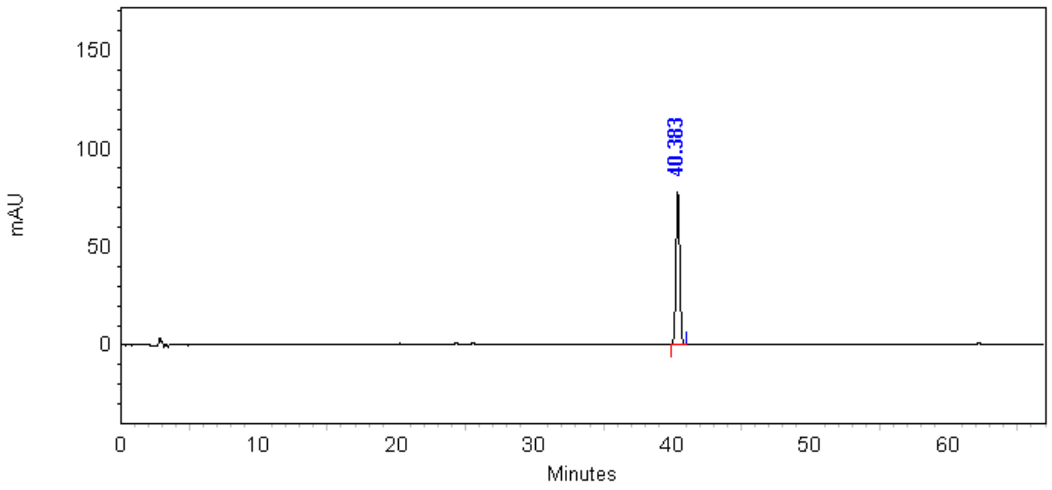

Peak No.	RT (min)	Area%
anti-**5c**	40.383	100.0

Figure S77. RP-HPLC chromatogram of compound *anti*-**5c**.

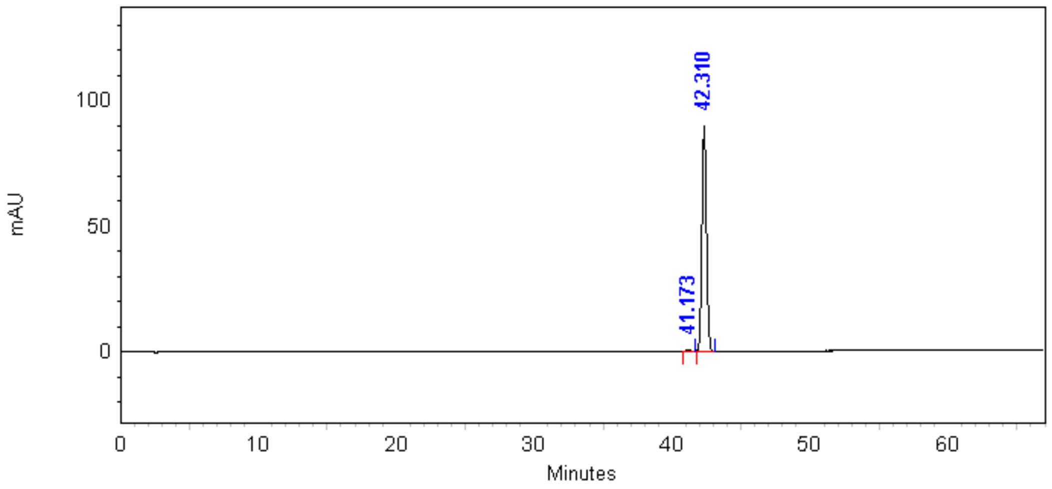

Peak No.	RT (min)	Area%
syn-**5c**	42.310	99.4

Figure S78. RP-HPLC chromatogram of compound *syn*-**5c**.

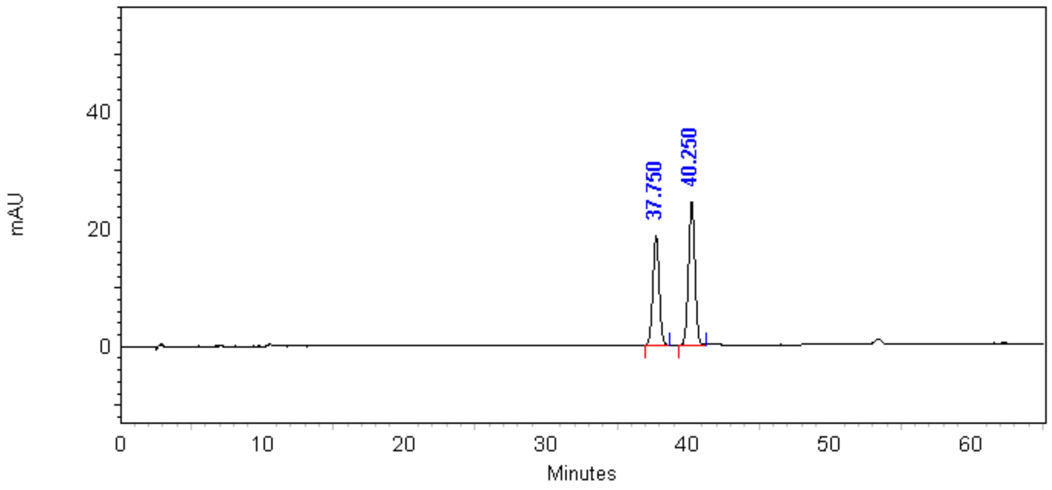

Peak No.	RT (min)	Area%
anti-**5d**	37.750	42.9
syn-**5d**	40.250	57.1

Figure S79. RP-HPLC chromatogram of compound *syn*/*anti*-**5d**.

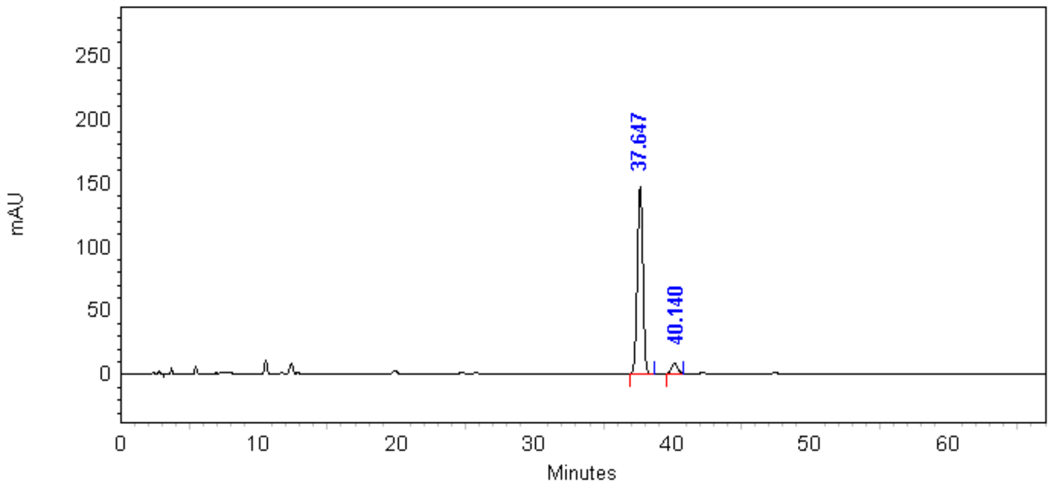

Peak No.	RT (min)	Area%
anti-**5d**	37.647	96.3

Figure S80. RP-HPLC chromatogram of compound *anti*-**5d**.

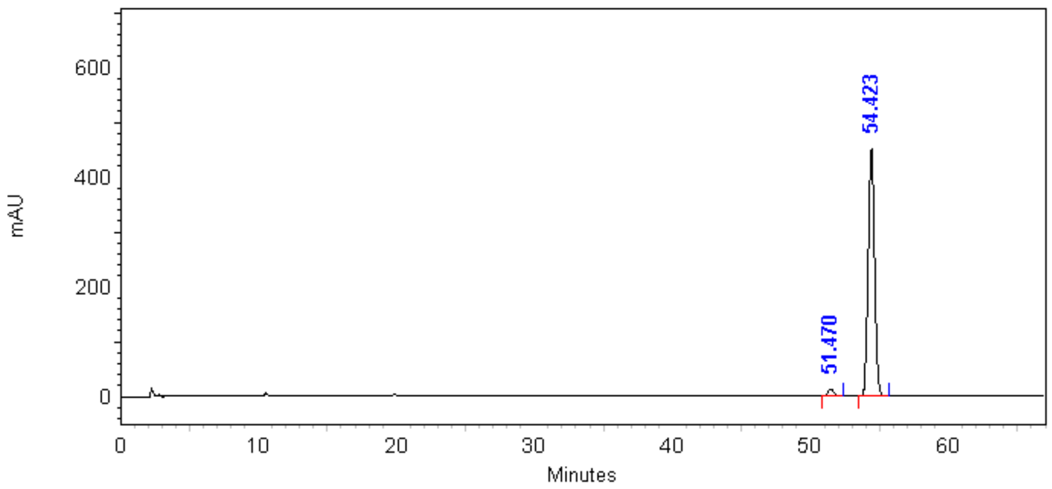

Peak No.	RT (min)	Area%
syn-**5d**	54.423	97.7

Figure S81. RP-HPLC chromatogram of compound *syn*-**5d**.

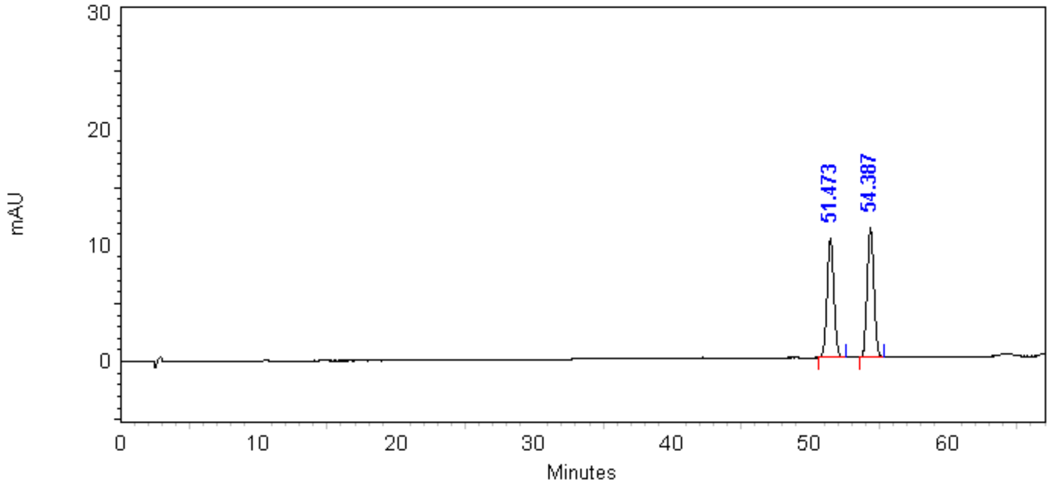

Peak No.	RT (min)	Area%
anti-**5e**	51.473	47.8
syn-**5e**	54.387	52.2

Figure S82. RP-HPLC chromatogram of compound *syn*/*anti*-**5e**.

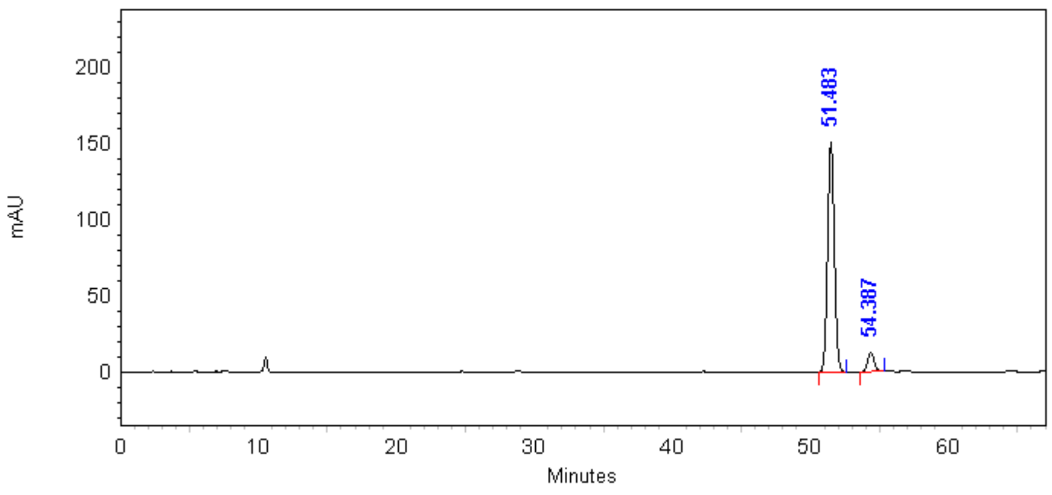

Peak No.	RT (min)	Area%
anti-**5e**	51.487	92.4

Figure S83. RP-HPLC chromatogram of compound *anti*-**5e**.

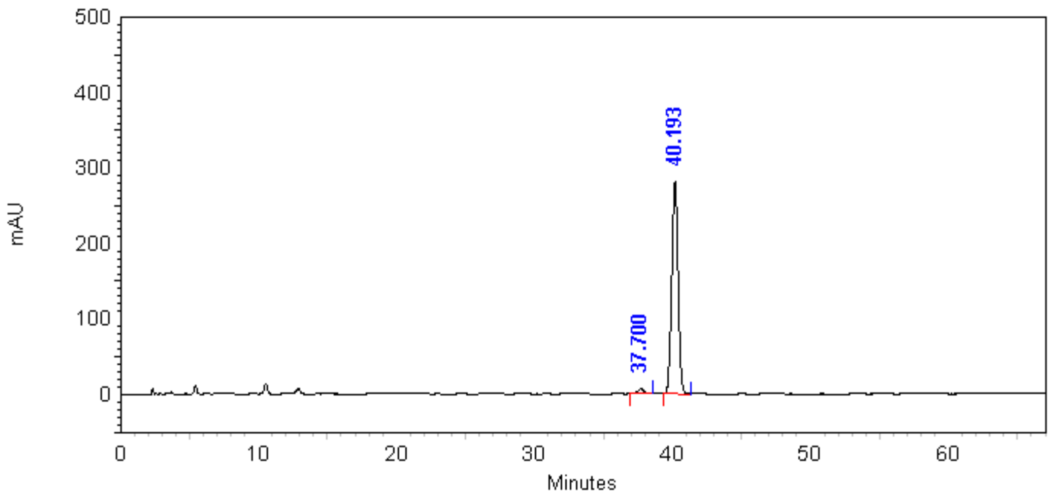

Peak No.	RT (min)	Area%
syn-**5e**	54.423	97.3

Figure S84. RP-HPLC chromatogram of compound *syn*-**5e**.

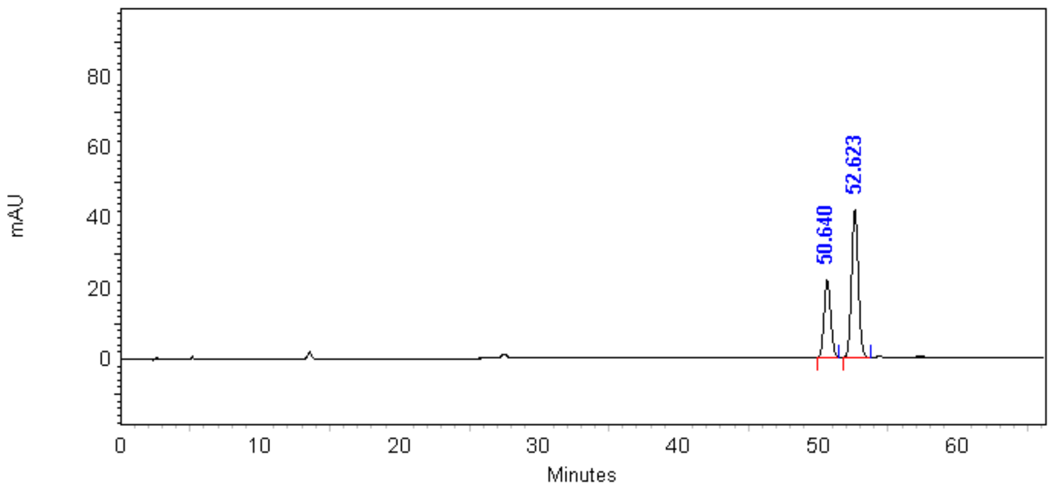

Peak No.	RT (min)	Area%
anti-**5f**	50.640	34.0
syn-**5f**	52.623	66.0

Figure S85. RP-HPLC chromatogram of compound *syn/anti*-**5f**.

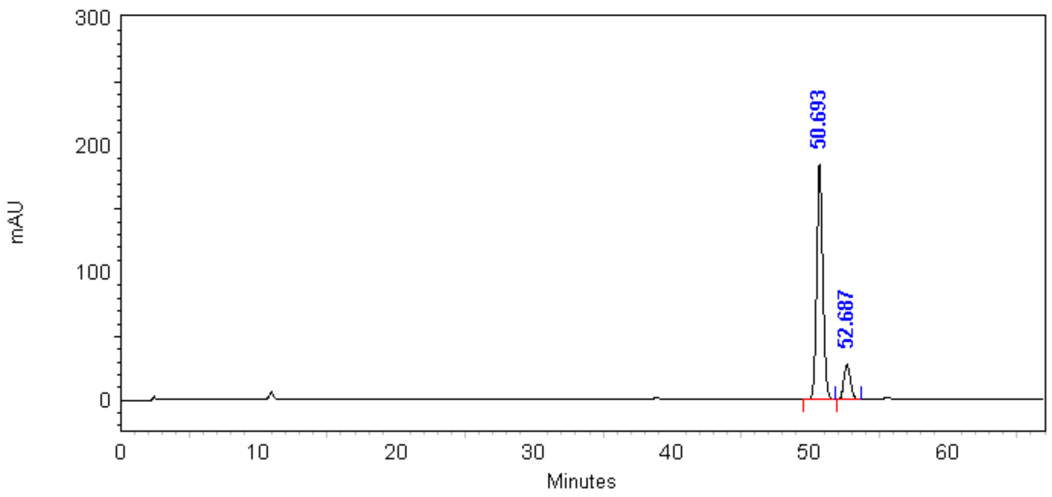

Peak No.	RT (min)	Area%
anti-**5f**	50.693	86.8

Figure S86. RP-HPLC chromatogram of compound *anti*-**5f**.

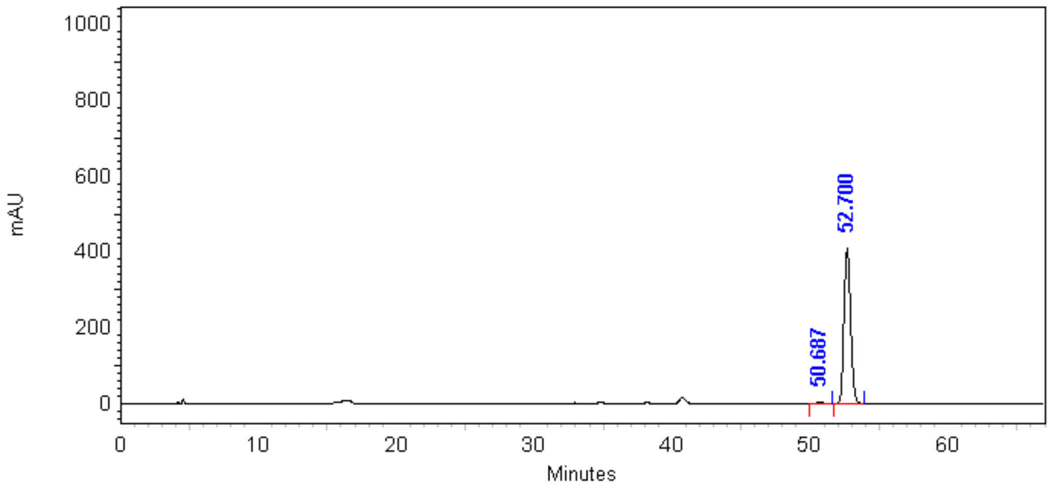

Peak No.	RT (min)	Area%
syn-**5f**	52.700	99.0

Figure S87. RP-HPLC chromatogram of compound *syn*-**5f**.

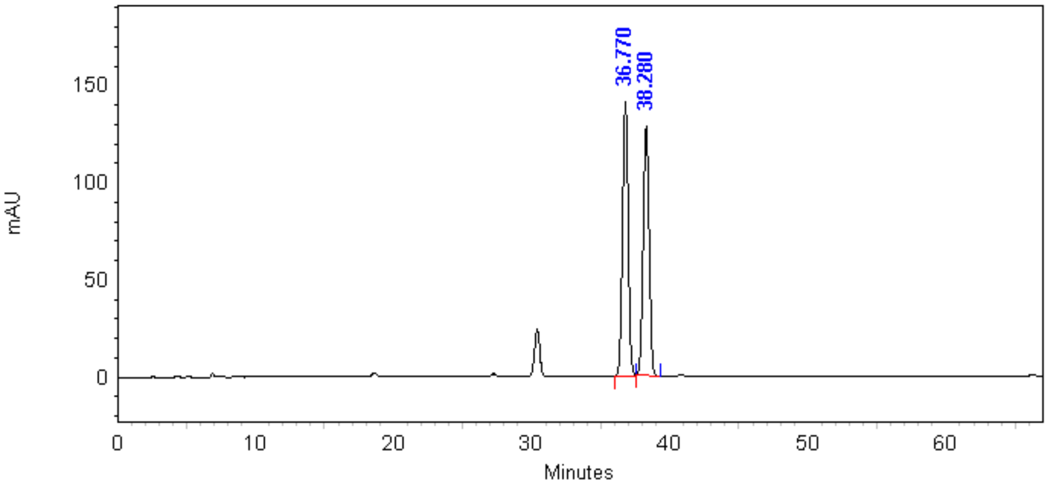

Peak No.	RT (min)	Area%
anti-**5g**	36.770	51.7
Syn-**5g**	38.280	48.3

Figure S88. RP-HPLC chromatogram of compound *syn*/*anti*-**5g**.

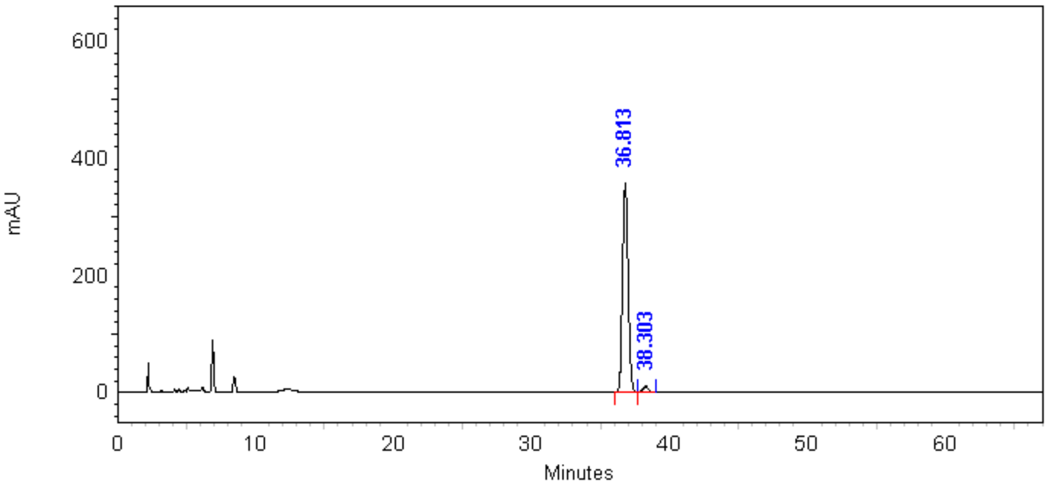

Peak No.	RT (min)	Area%
anti-**5g**	36.813	97.5

Figure S89. RP-HPLC chromatogram of compound *anti*-**5g**.

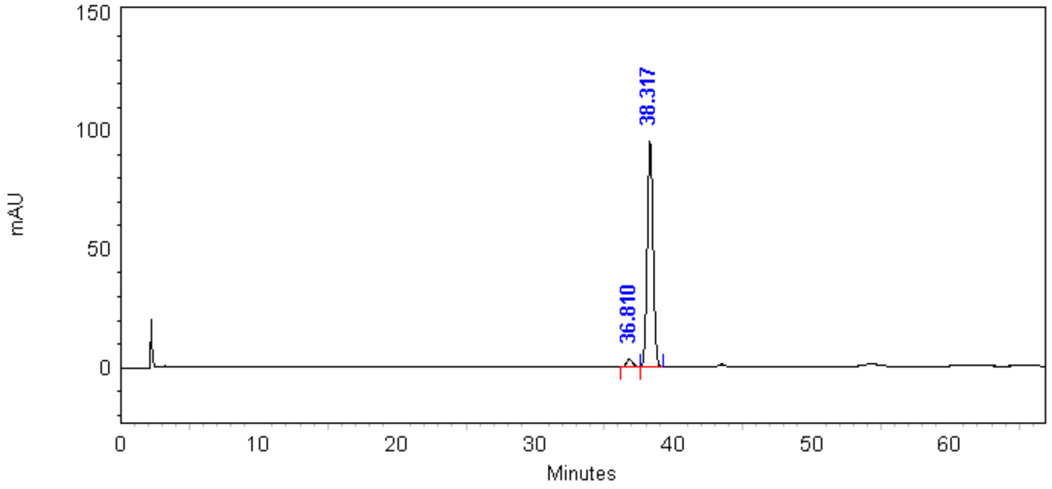

Peak No.	RT (min)	Area%
syn-**5g**	38.317	96.7

Figure S90. RP-HPLC chromatogram of compound *syn*-**5g**.

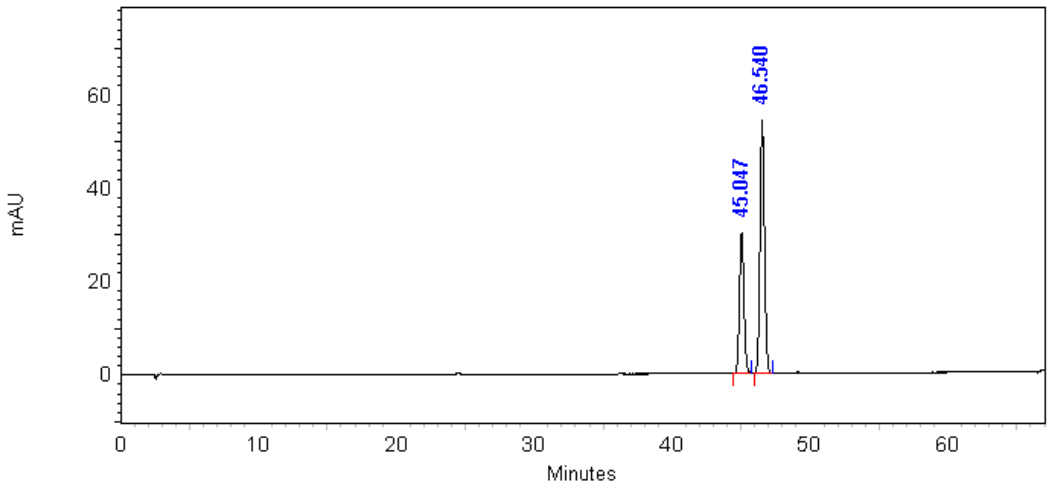

Peak No.	RT (min)	Area%
anti-**5h**	45.047	35.7
syn-**5h**	46.540	64.3

Figure S91. RP-HPLC chromatogram of compound *syn*/*anti*-**5h**.

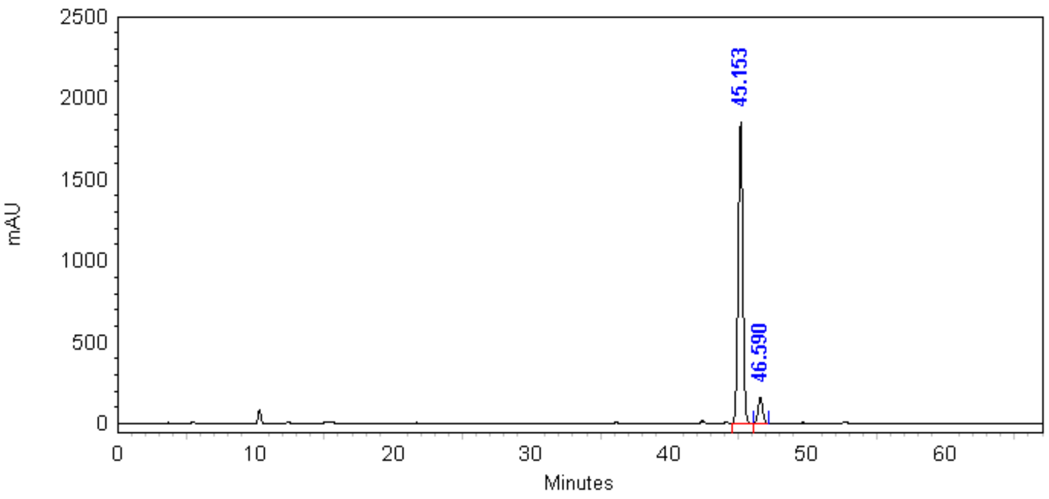

Peak No.	RT (min)	Area%
anti-**5h**	45.153	92.3

Figure S92. RP-HPLC chromatogram of compound *anti*-**5h**.

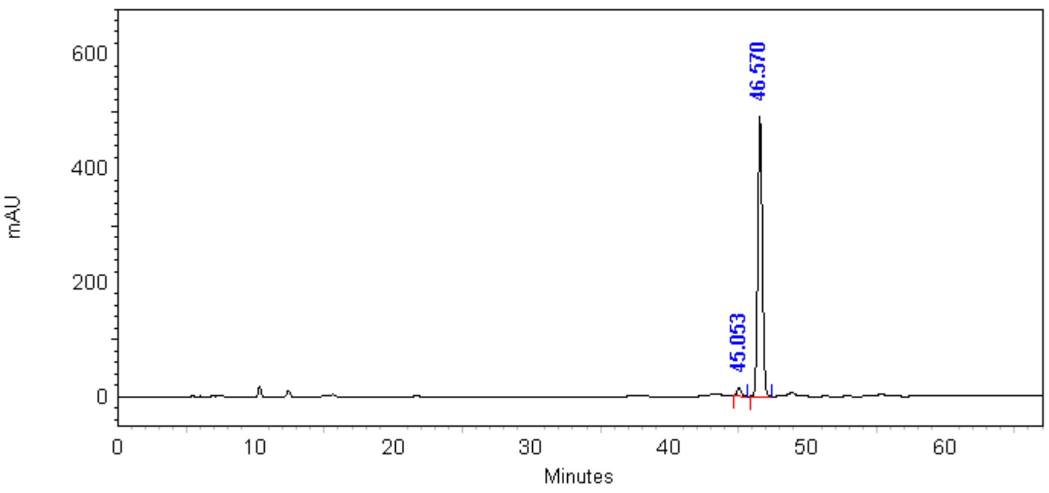

Peak No.	RT (min)	Area%
syn-**5h**	46.570	97.3

Figure S93. RP-HPLC chromatogram of compound *syn*-**5h**.

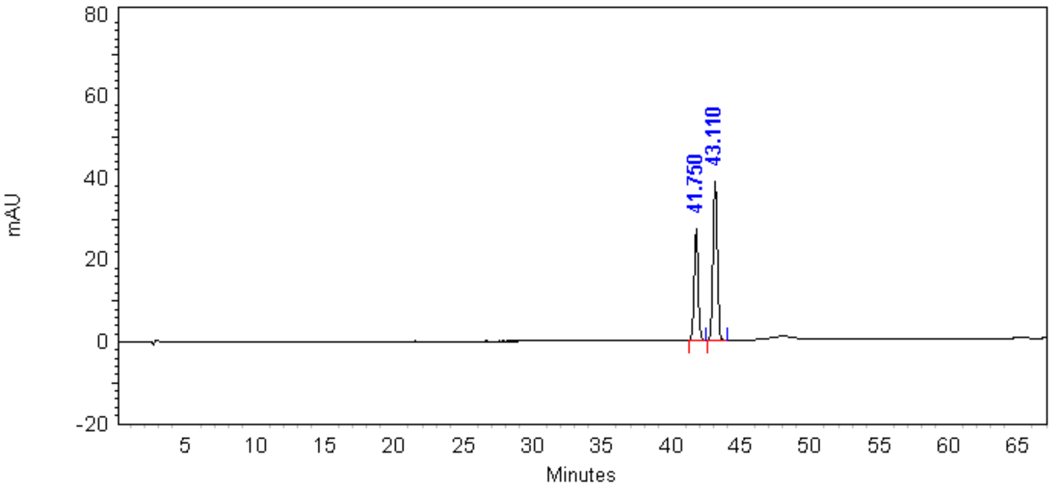

Peak No.	RT (min)	Area%
anti-**5i**	41.750	41.2
syn-**5i**	43.110	58.8

Figure S94. RP-HPLC chromatogram of compound *syn*/*anti*-**5i**.

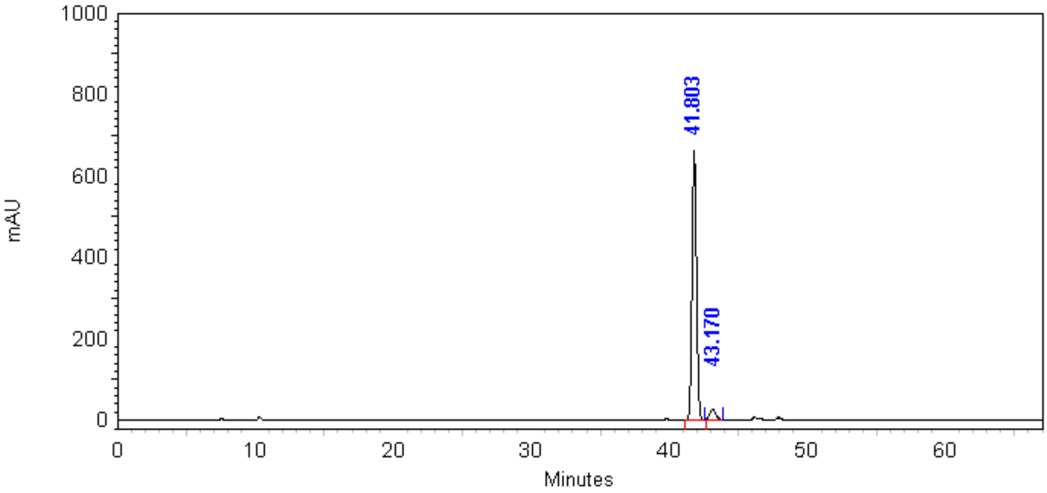

Peak No.	RT (min)	Area%
anti-**5i**	41.803	96.8

Figure S95. RP-HPLC chromatogram of compound *anti*-**5i**.

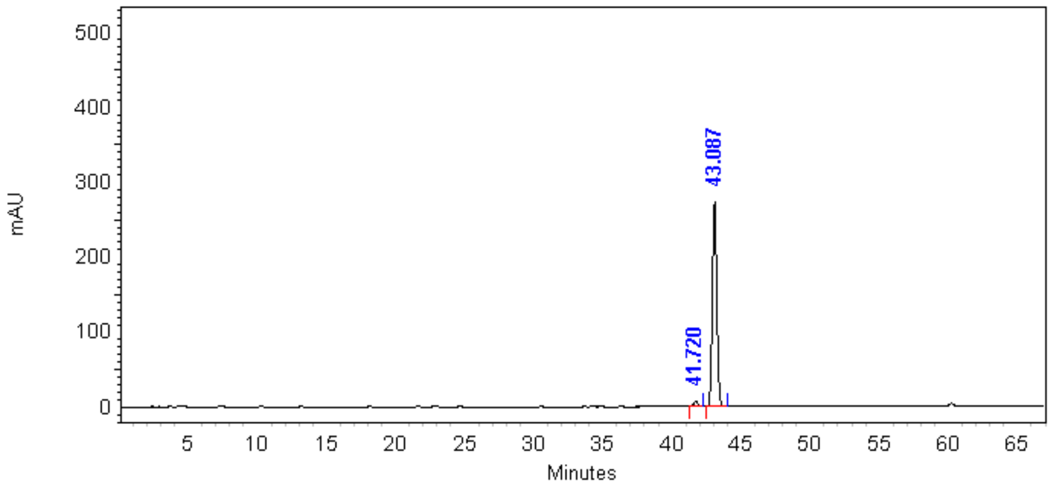

Peak No.	RT (min)	Area%
syn-**5i**	43.087	97.5

Figure S96. RP-HPLC chromatogram of compound *syn*-**5i**.

Chromatographic conditions for *syn*/*anti*-3,5-disubstituted hydantoins **5a-i**

**Column:**	Zorbax Extend C-18 PrepHT preparative column (250 mm × 21.2 mm i. d., 5-µm, Agilent Technologies, Milford, MA, USA).		
**Mobile phase A:**	Water		
**Mobile phase B:**	Acetonitrile		
**Flow:**	17 mL/min		
**Detection:**	254 nm		
**Column temperature:**	room temperature		
**Injection volume:**	500 µL		
**Method D:**	t/min	%MP A	%MP B
	0	65	35
	35.98	52	48
	37.78	52	48
	37.79	65	35
	40.18	65	35

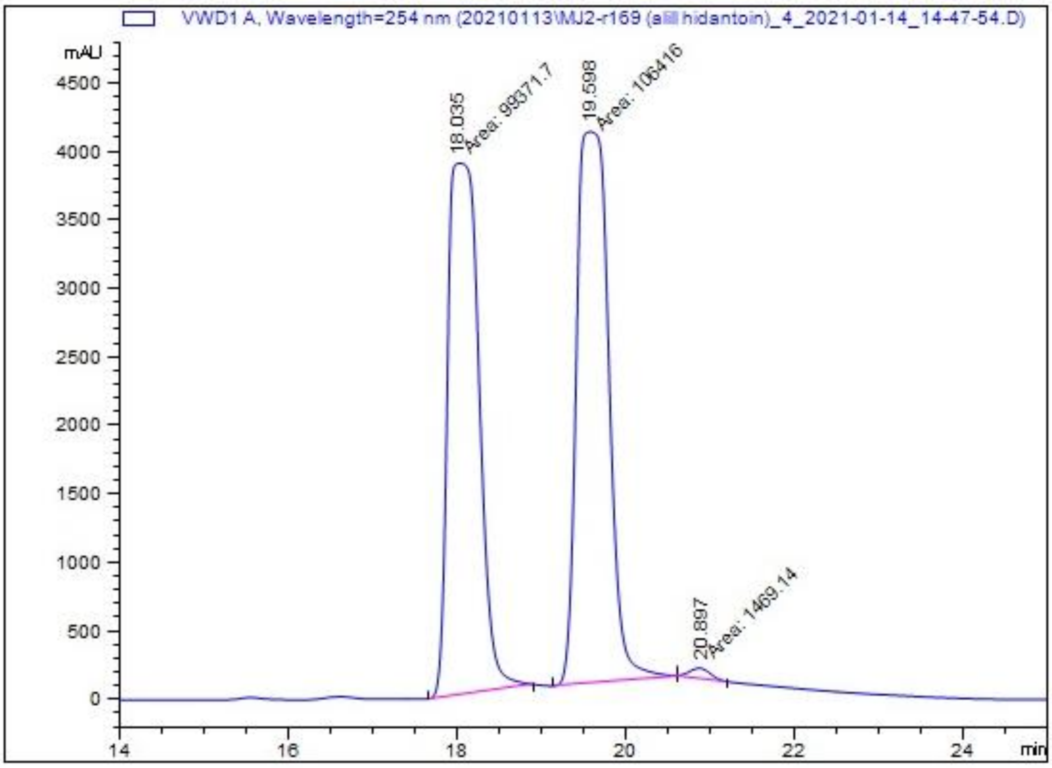

Figure S97. Chromatogram of *syn*/*anti*-**5a** on preparative Zorbax Extend C-18 PrepHT preparative column.

4. HPLC enantioseparation of allyl hydantoin *anti*-5a and *syn*-5a

Chromatographic conditions for analytical column

**Column:**	CHIRAL ART Amylose-SA (250 mm × 4.6 mm i. d., 10-µm)
**Mobile phase:**	*n*-hexane/2-PrOH (90/10, v/v)
**Flow:**	1,0 mL/min
**Detection:**	254 nm
**Column temperature:**	30 °C
**Injection volume:**	20 µL
**Run time:**	40 min

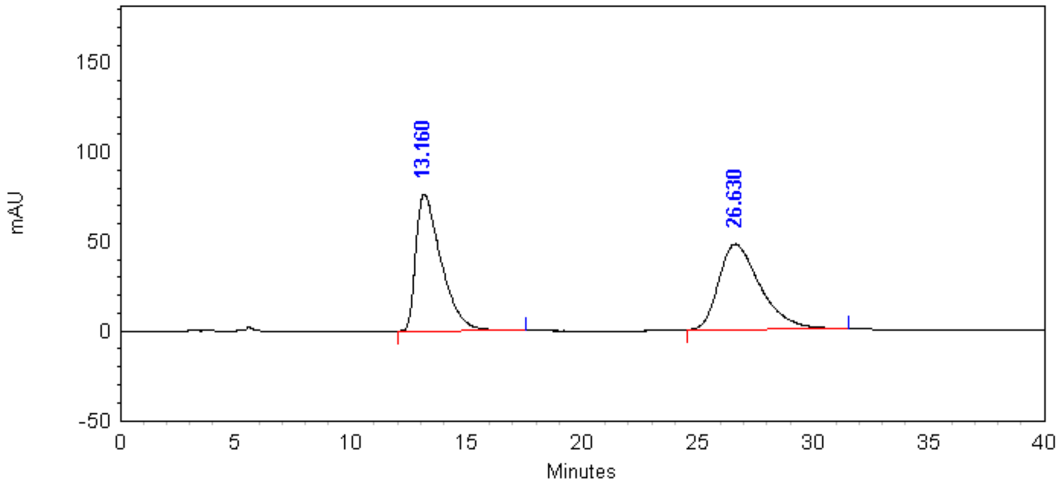

Figure S98. Chromatogram of *anti*-**5a** (Peak 1) on analytical CHIRAL ART Amylose SA column.

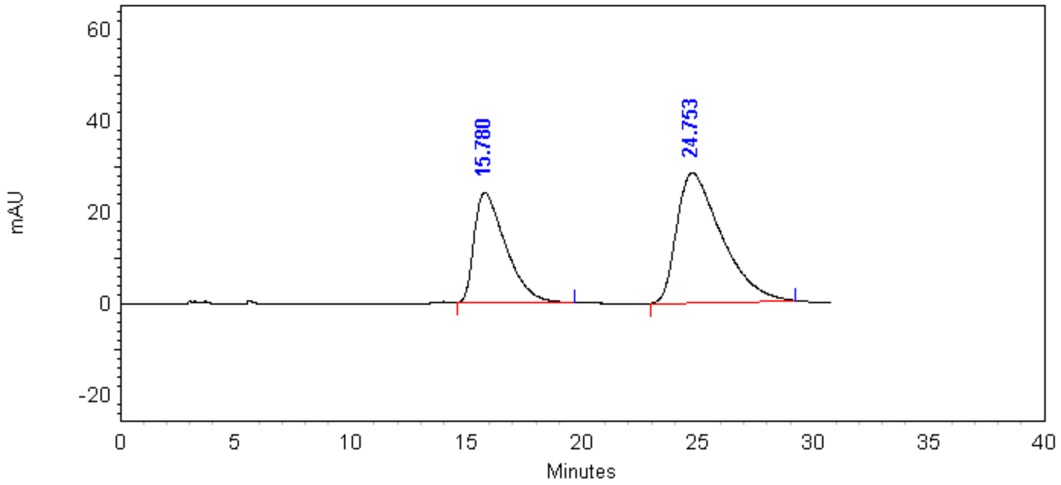

Figure S99. Chromatogram of *syn*-**5a** (Peak 2) on analytical CHIRAL ART Amylose SA column.

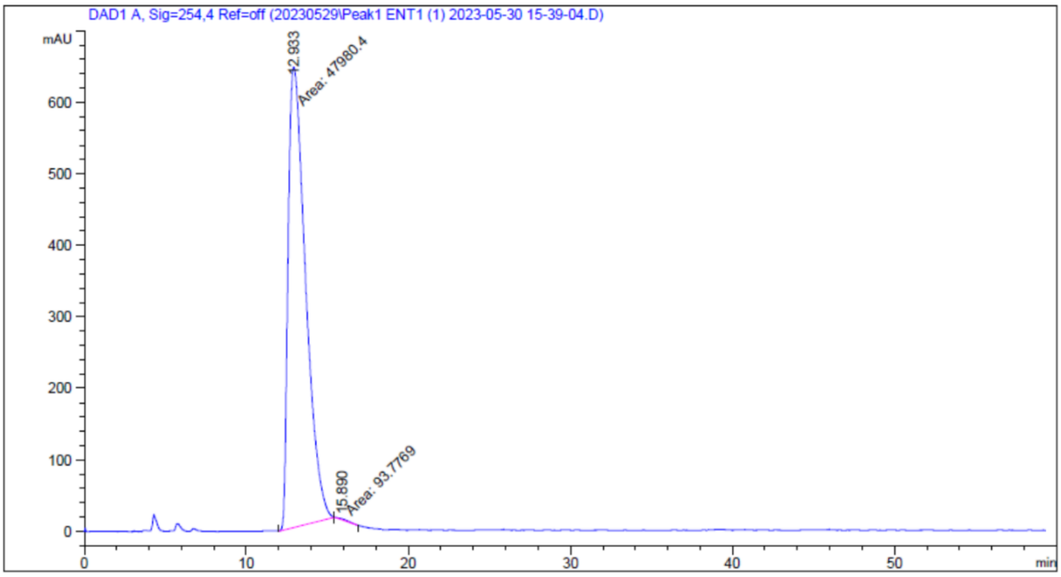

Figure S100. Chromatogram of enantiomer **5a-ent1** (*anti*-**5a**, Peak 1).

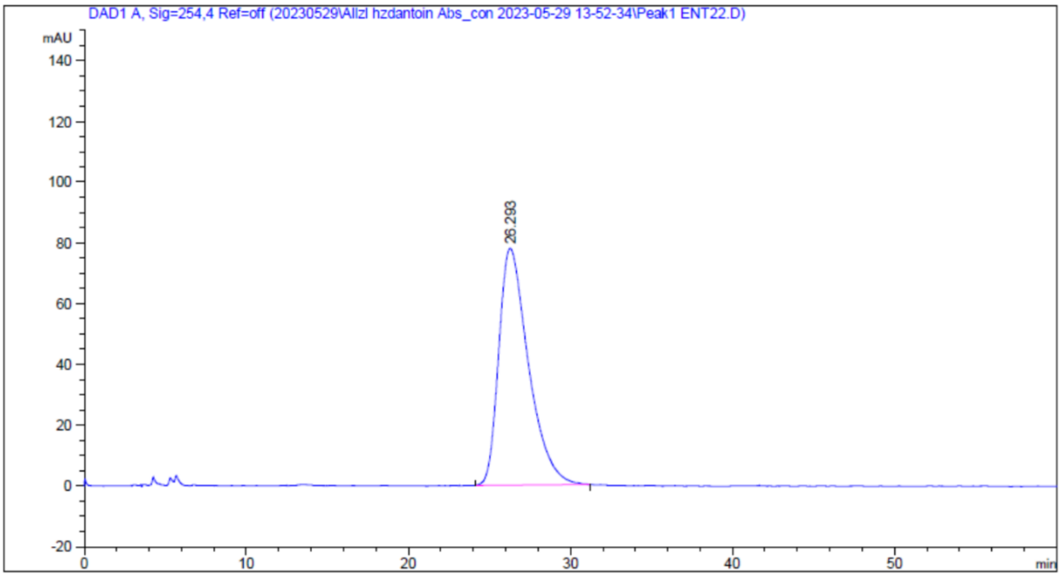

Figure S101. Chromatogram of **5a-ent2** (*anti*-**5a**, Peak 1).

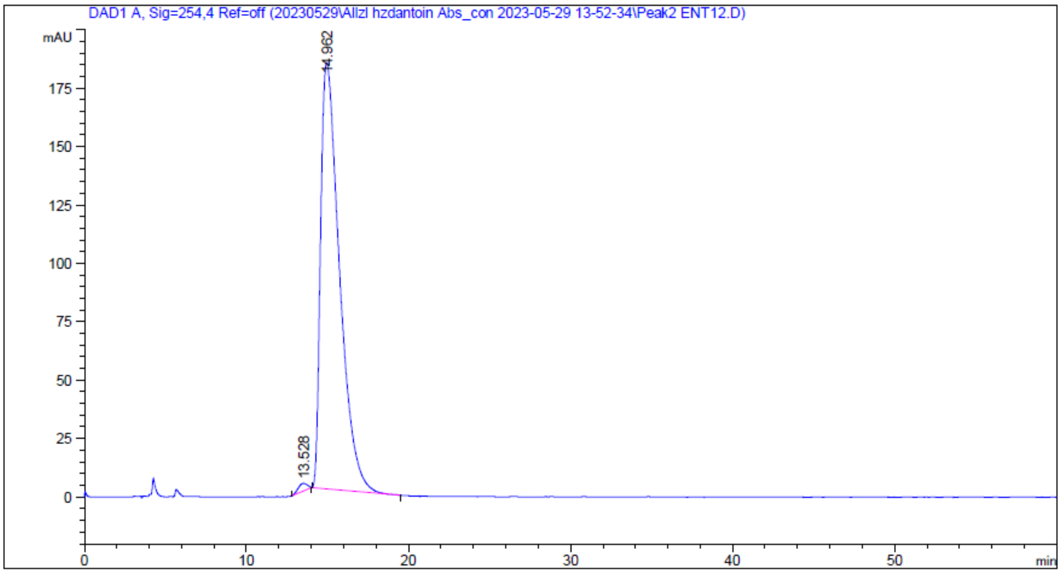

Figure S102. Chromatogram of **5a-ent3** (*syn*-**5a**, Peak 2).

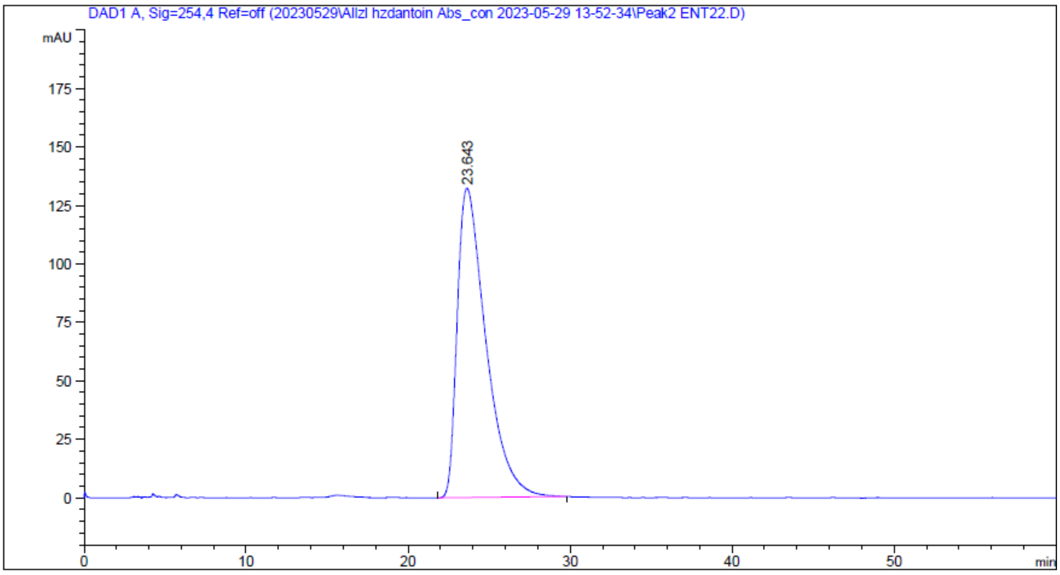

Figure S103. Chromatogram of **5a-ent4** (*syn*-**5a**, Peak 2).

Chromatographic conditions for semi-preparative column

**Column:**	CHIRAL ART Amylose-SA (250 mm × 8.0 mm i. d., 10-µm)
**Mobile phase:**	*n*-hexane/2-PrOH (90/10, v/v)
**Flow:**	5.0 mL/min
**Detection:**	254 nm
**Column temperature:**	room temperature
**Injection volume:**	500 µL
**Run time:**	30 min

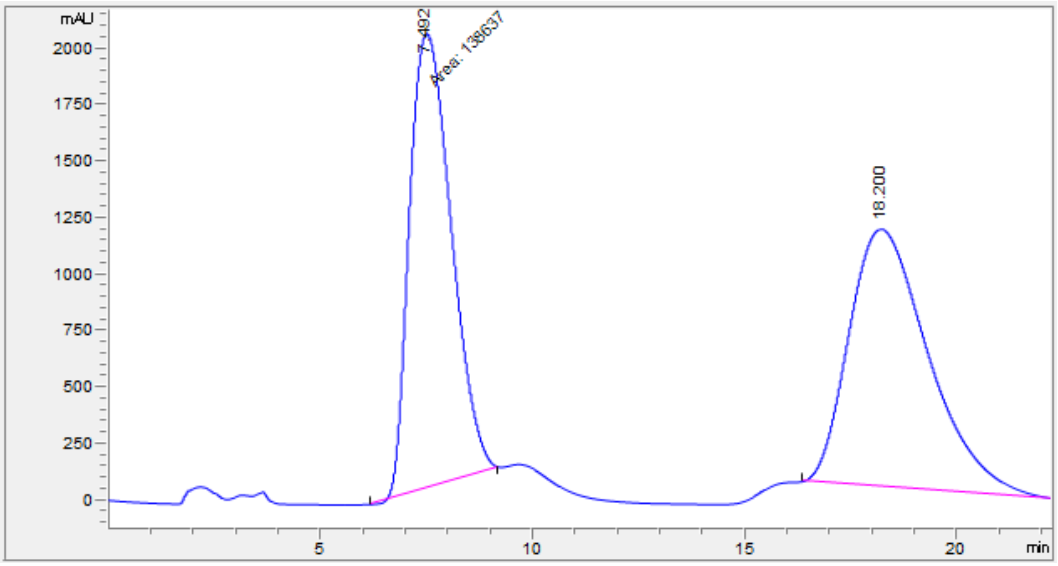

Figure S104. Chromatogram of *anti*-**5a** (Peak 1) on the semi-preparative CHIRAL ART Amylose SA column.

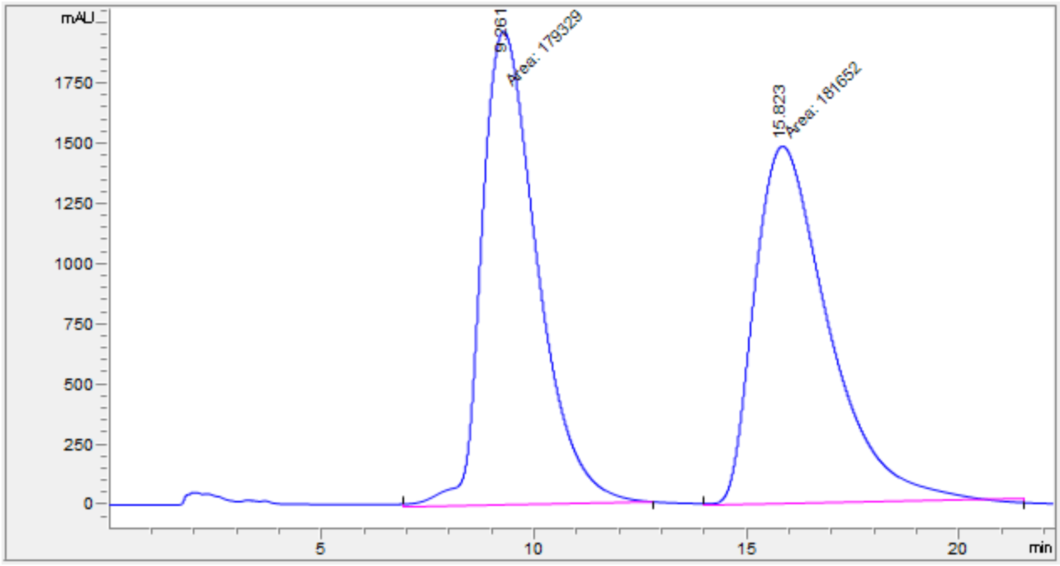

Figure S105. Chromatogram of *syn*-**5a** (Peak 2) on the semi-preparative CHIRAL ART Amylose SA column.

5. HRMS spectra

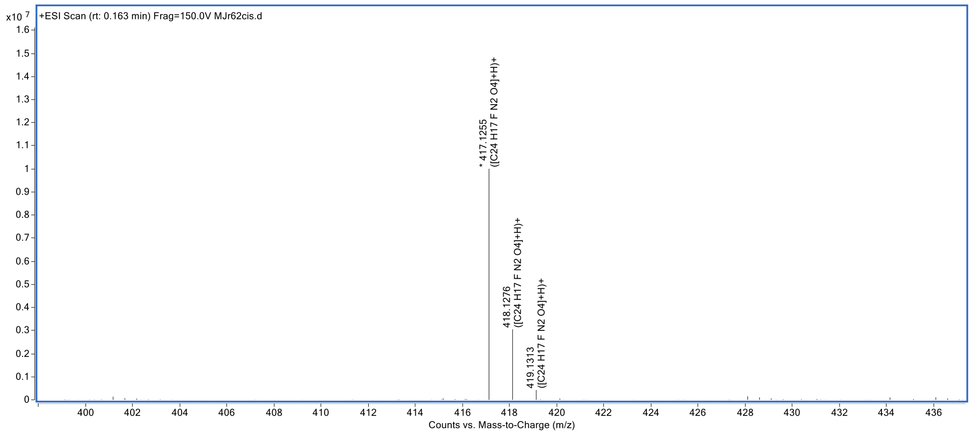

Figure S106. HR-MS (ESI-QTOF) spectra of compound **2a**.

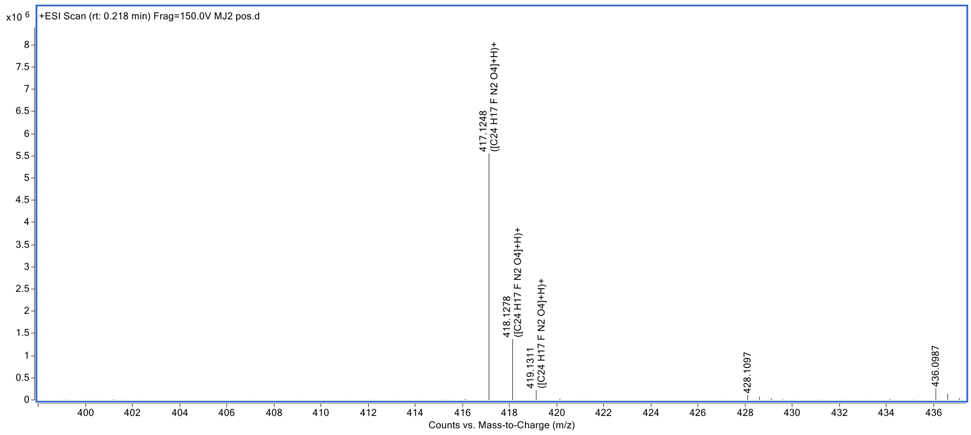

Figure S107. HR-MS (ESI-QTOF) spectra of compound **2b**.

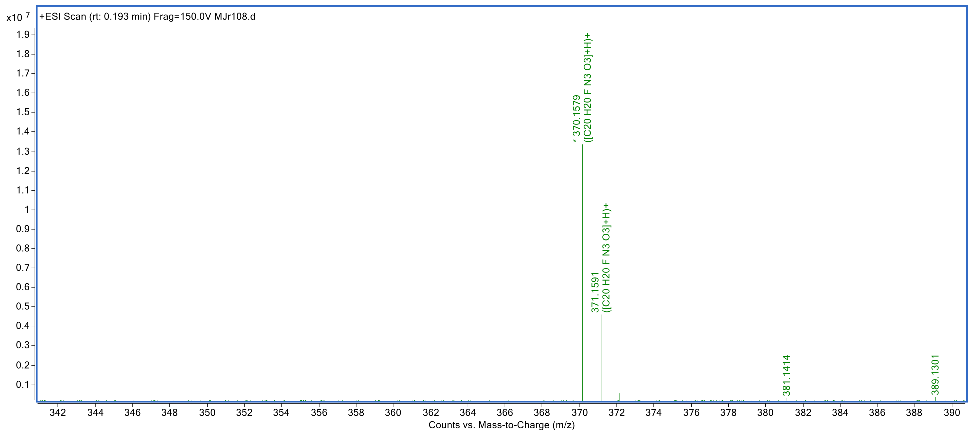

Figure S108. HR-MS (ESI-QTOF) spectra of compound **4a**.

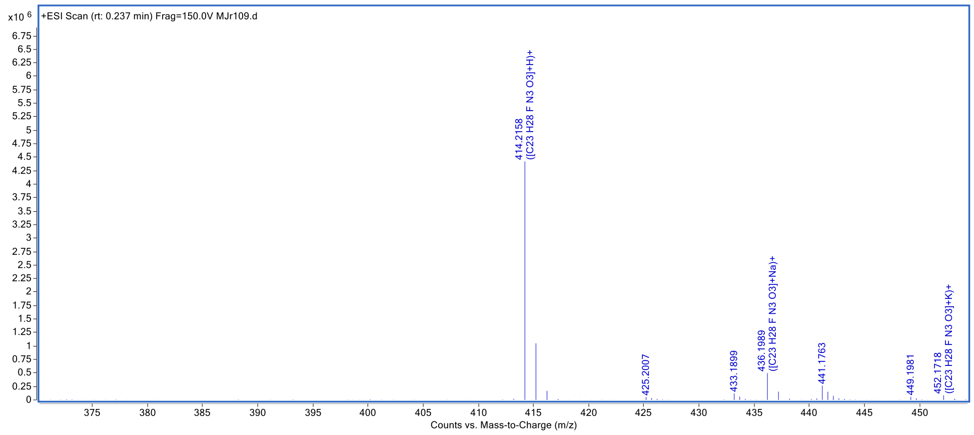

Figure S109. HR-MS (ESI-QTOF) spectra of compound **4b**.

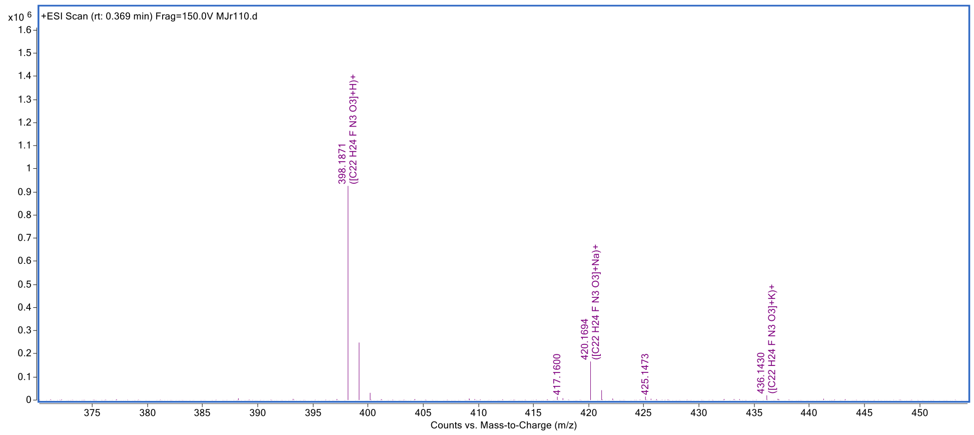

Figure S110. HR-MS (ESI-QTOF) spectra of compound **4c**.

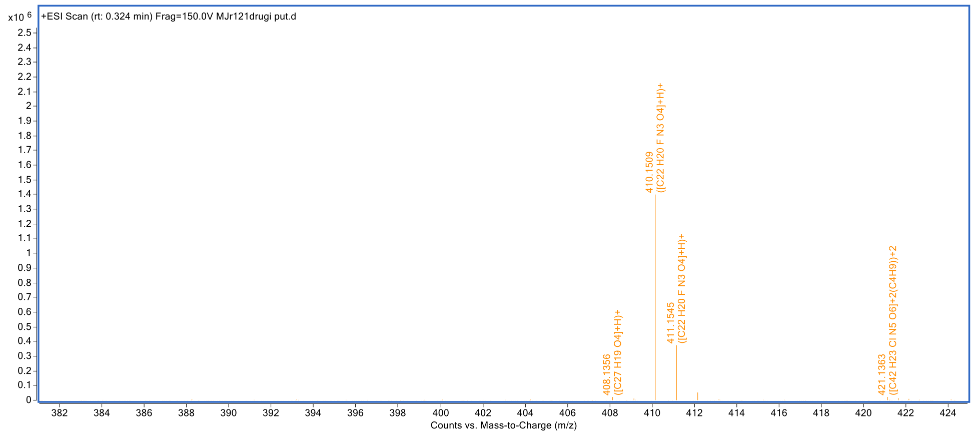

Figure S111. HR-MS (ESI-QTOF) spectra of compound **4d**.

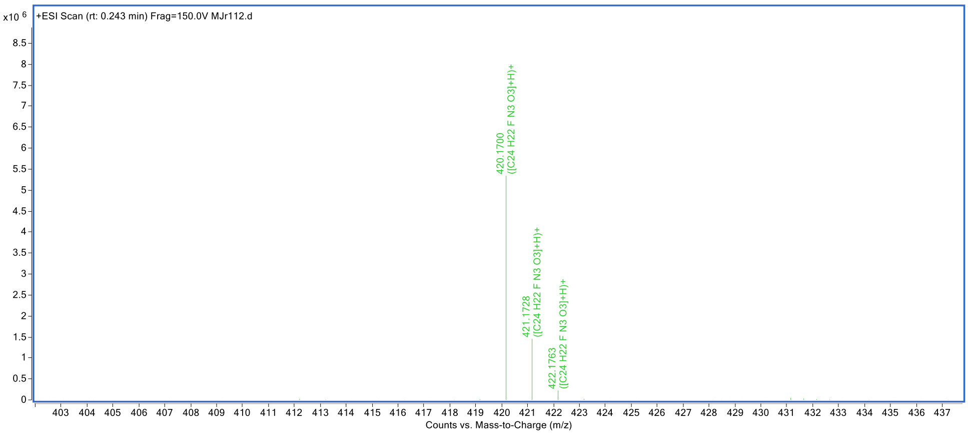

Figure S112. HR-MS (ESI-QTOF) spectra of compound **4e**.

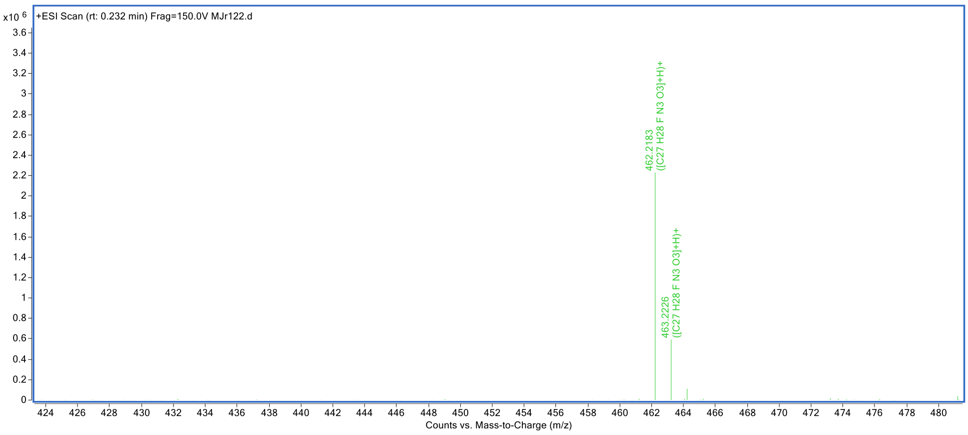

Figure S113. HR-MS (ESI-QTOF) spectra of compound **4f**.

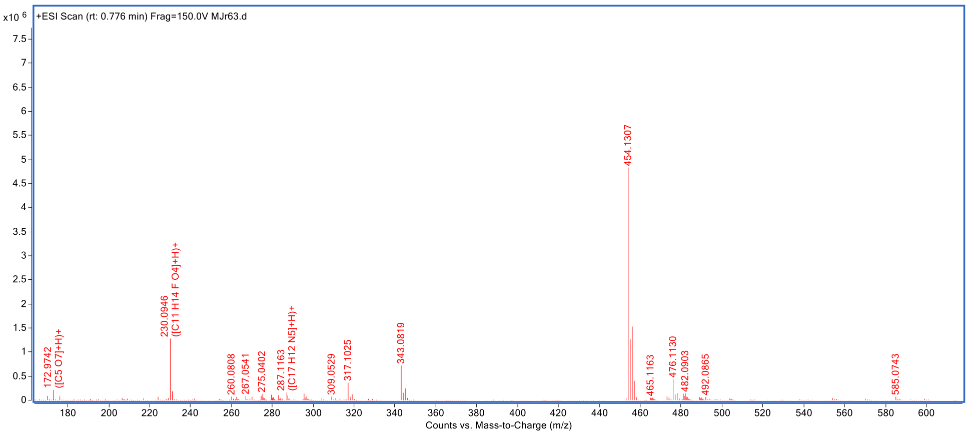

Figure S114. HR-MS (ESI-QTOF) spectra of compound **4g**.

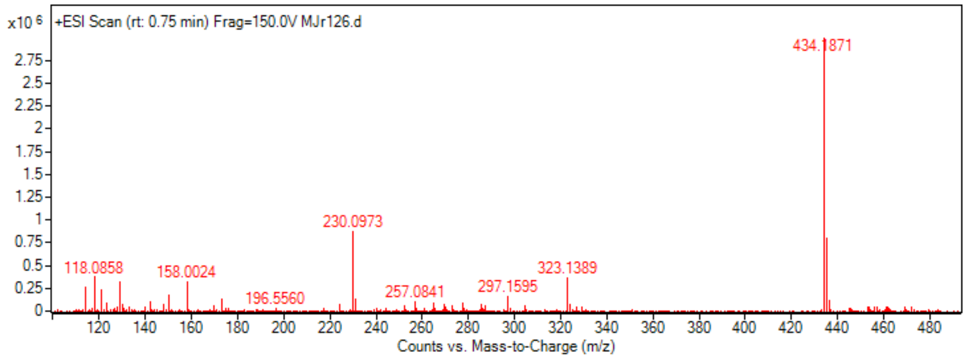

Figure S115. HR-MS (ESI-QTOF) spectra of compound **4h**.

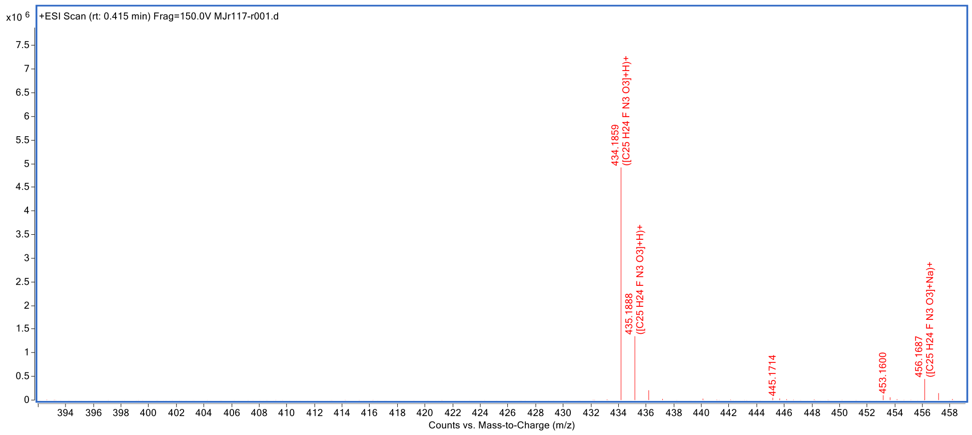

Figure S116. HR-MS (ESI-QTOF) spectra of compound **4i**.

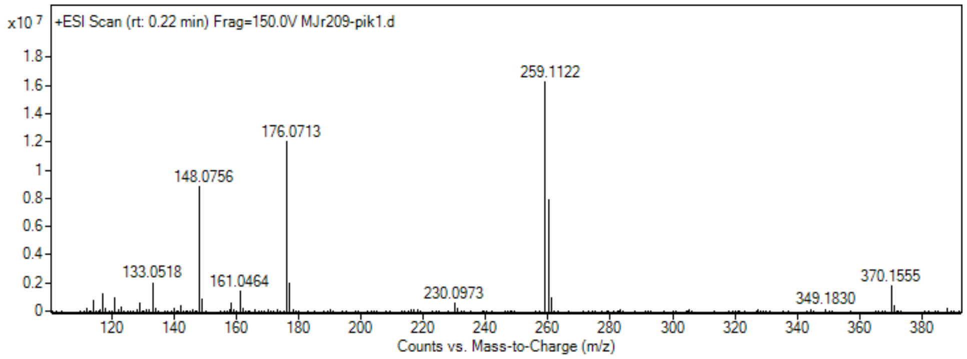

Figure S117. HR-MS (ESI-QTOF) spectra of compound *syn*/*anti*-**5a**.

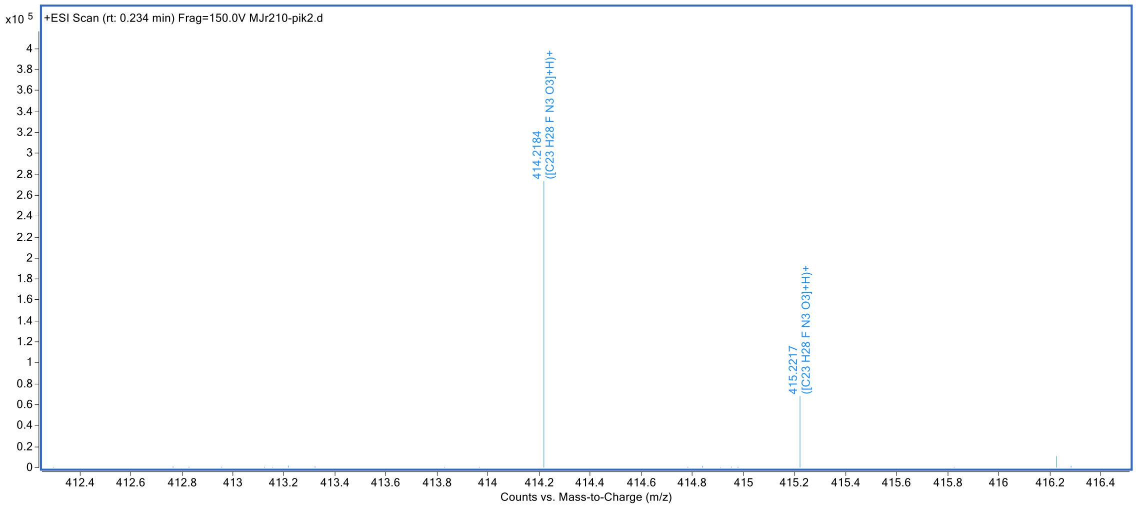

Figure S118. HR-MS (ESI-QTOF) spectra of compound *syn*/*anti*-**5b**.

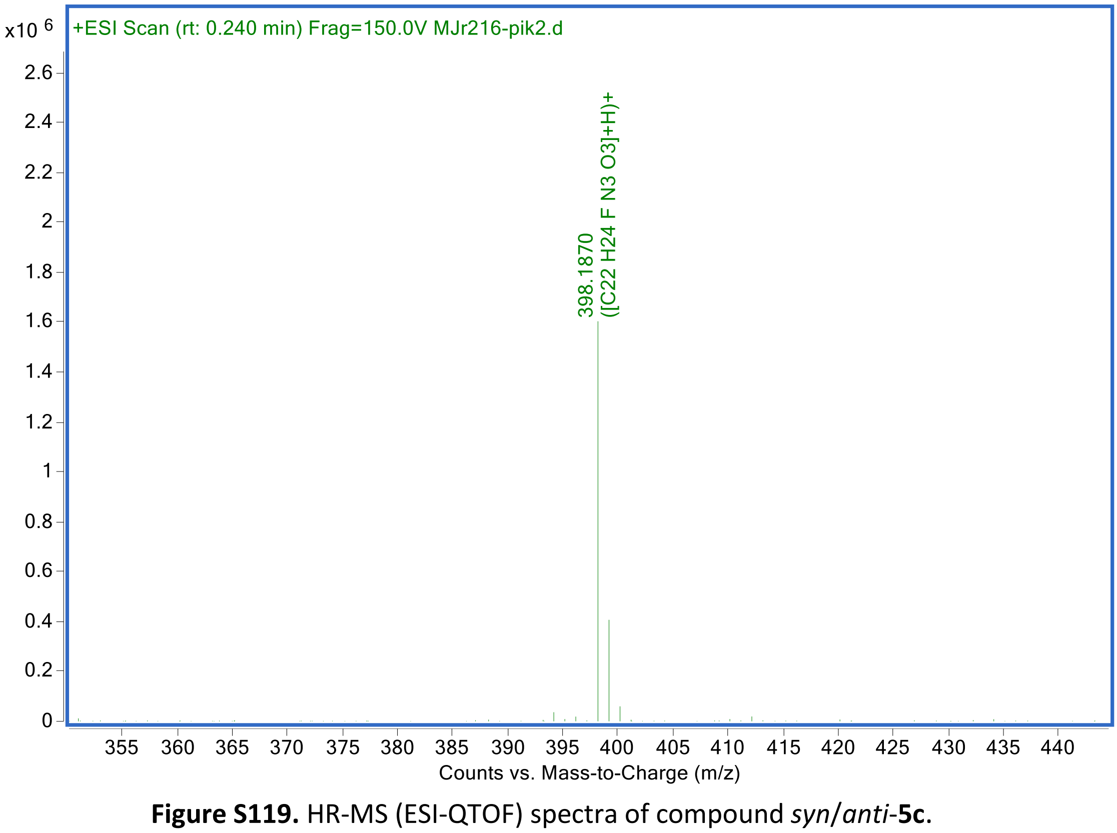

Figure S119. HR-MS (ESI-QTOF) spectra of compound *syn*/*anti*-**5c**.

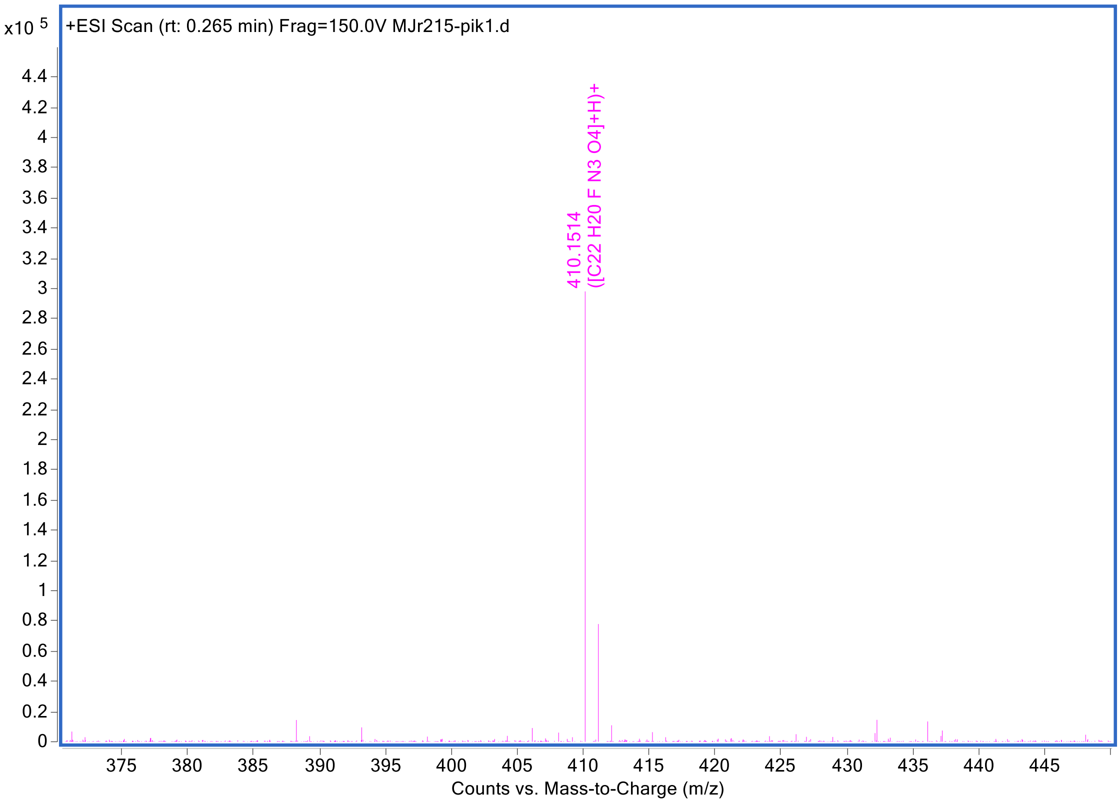

Figure S120. HR-MS (ESI-QTOF) spectra of compound *syn*/*anti*-**5d**.

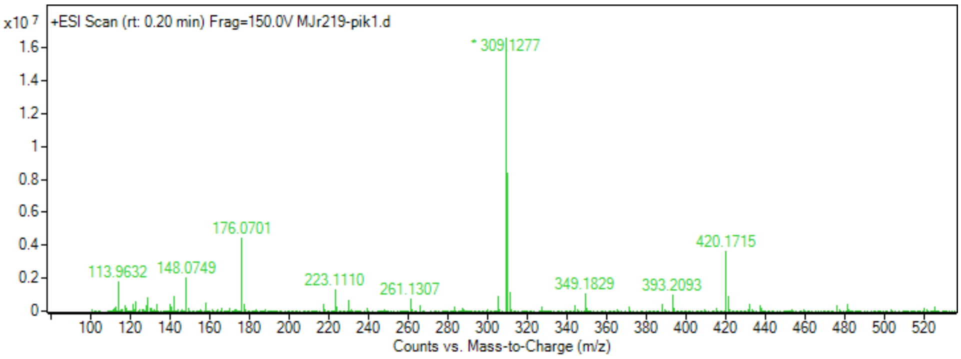

Figure S121. HR-MS (ESI-QTOF) spectra of compound *syn*/*anti*-**5e**.

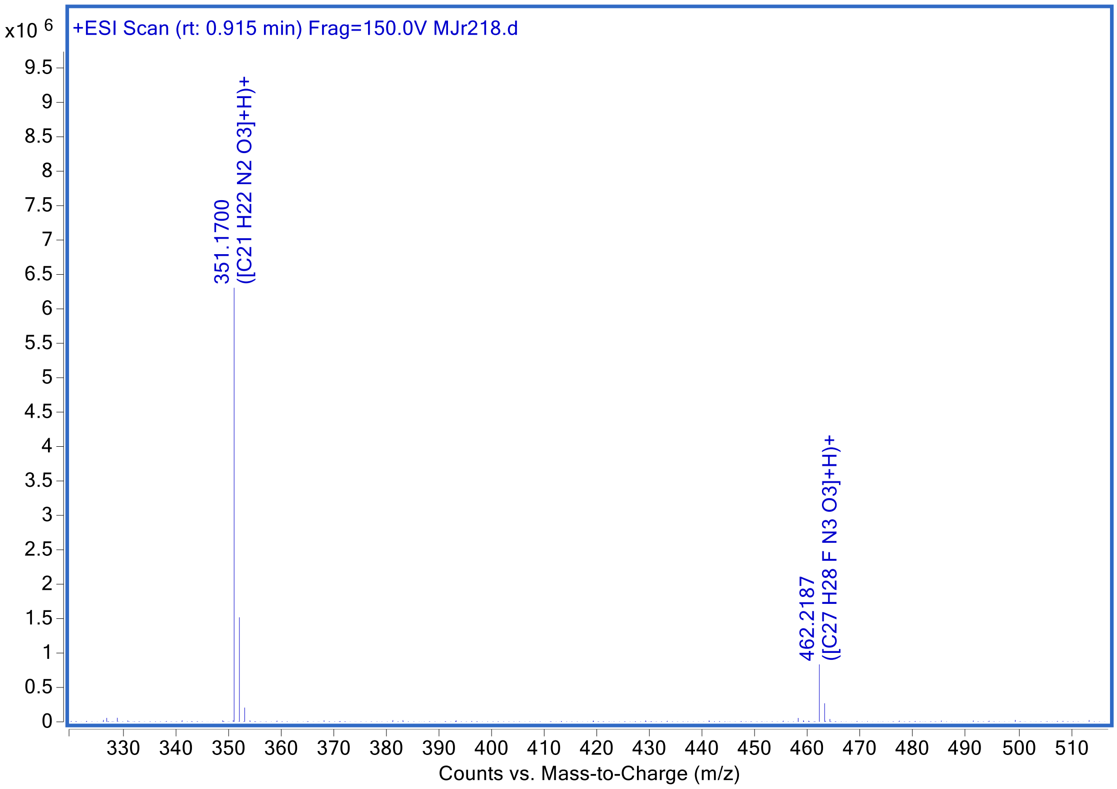

Figure S122. HR-MS (ESI-QTOF) spectra of compound *syn*/*anti*-**5f**.

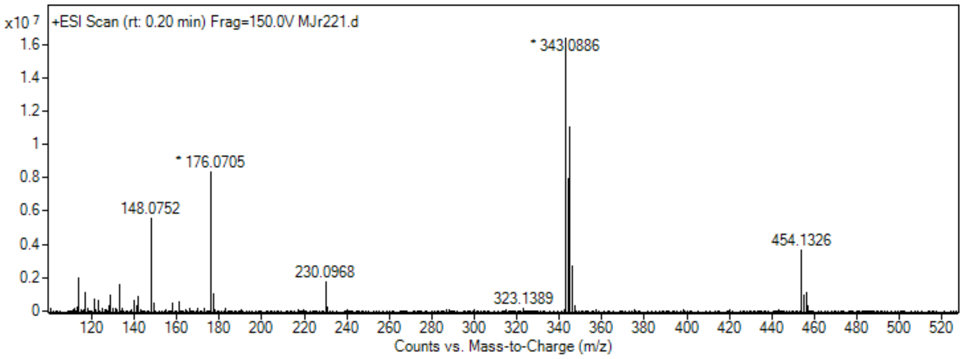

Figure S123. HR-MS (ESI-QTOF) spectra of compound *syn*/*anti*-**5g**.

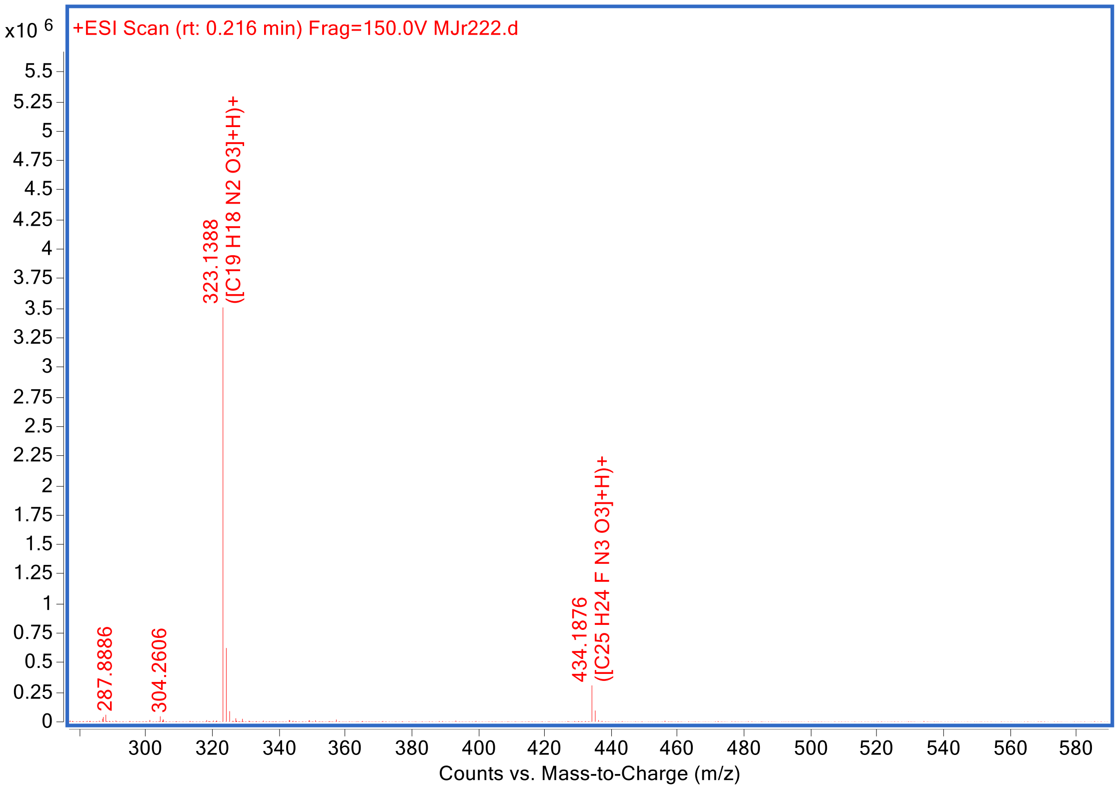

Figure S124. HR-MS (ESI-QTOF) spectra of compound *syn*/*anti*-**5h**.

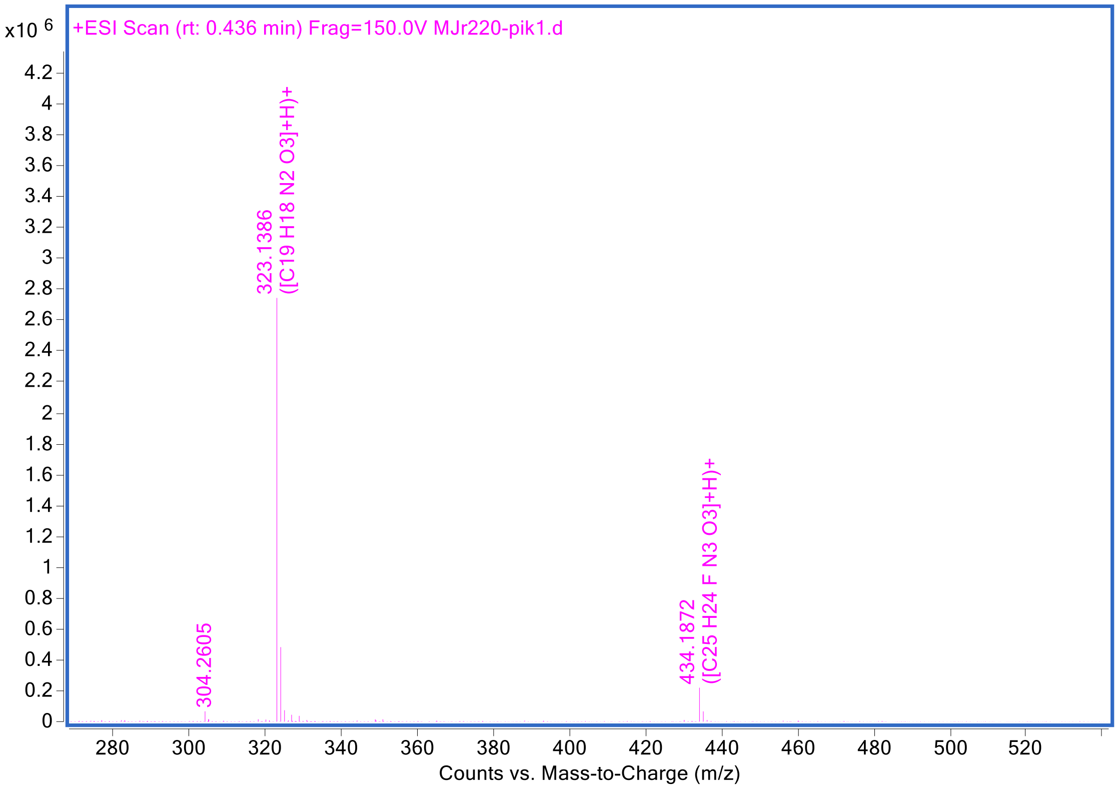

Figure S125. HR-MS (ESI-QTOF) spectra of compound *syn*/*anti*-**5i**.

6. IR spectra

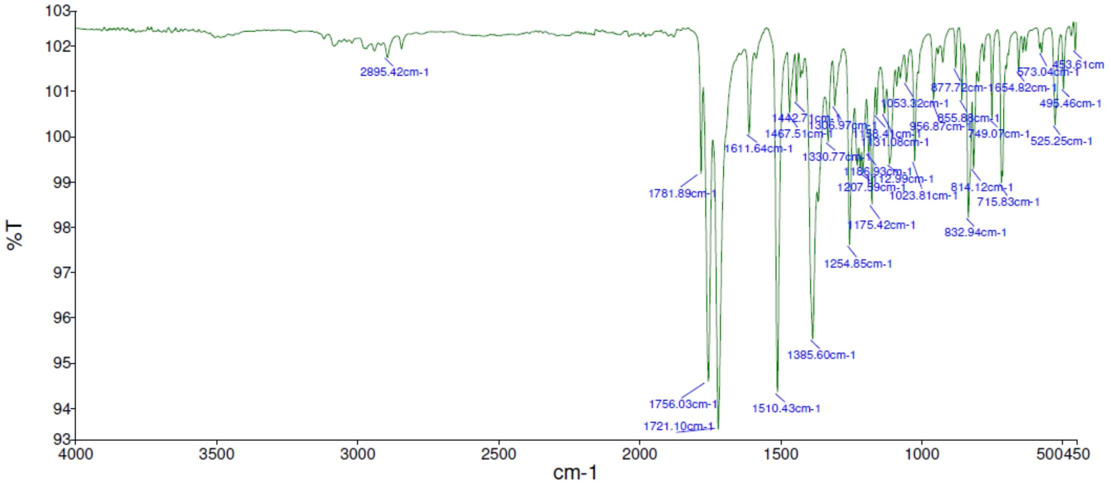

Figure S126. IR spectra of compound **2a**.

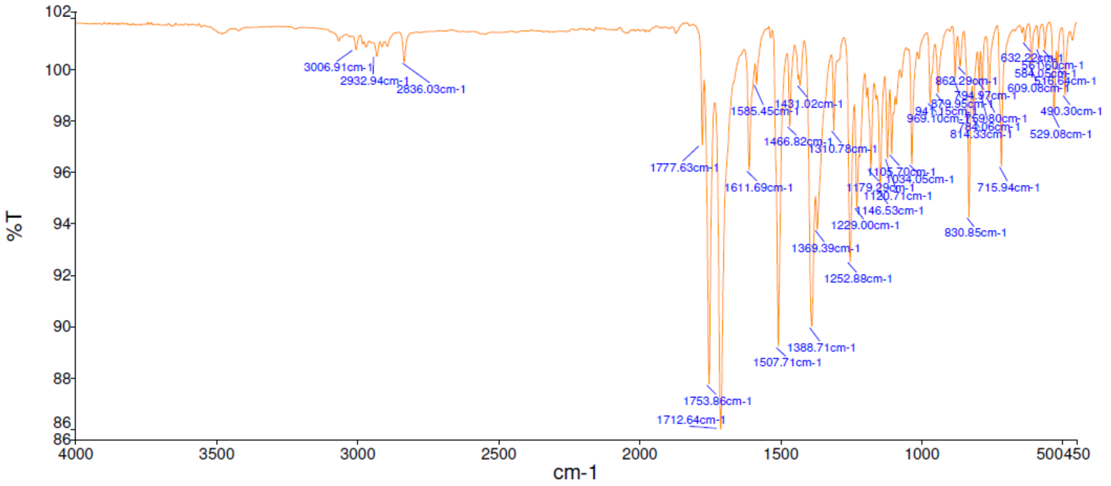

Figure S127. IR spectra of compound **2b**.

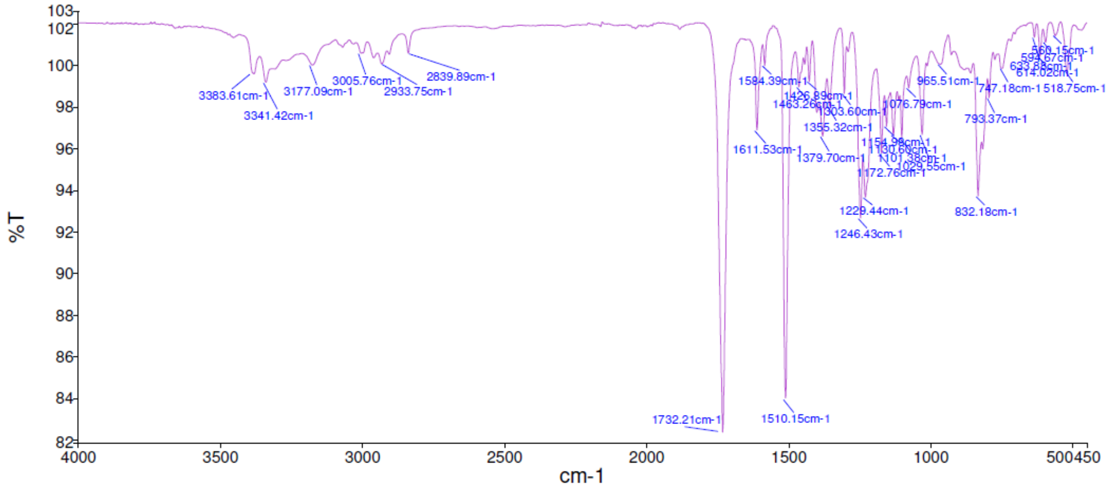

Figure S128. IR spectra of compound **3**.

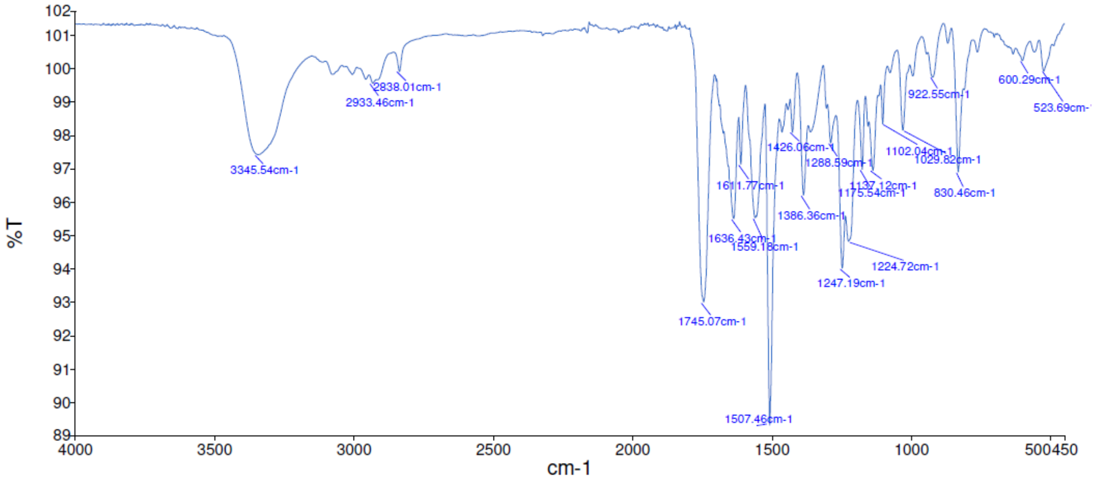

Figure S129. IR spectra of compound **4a**.

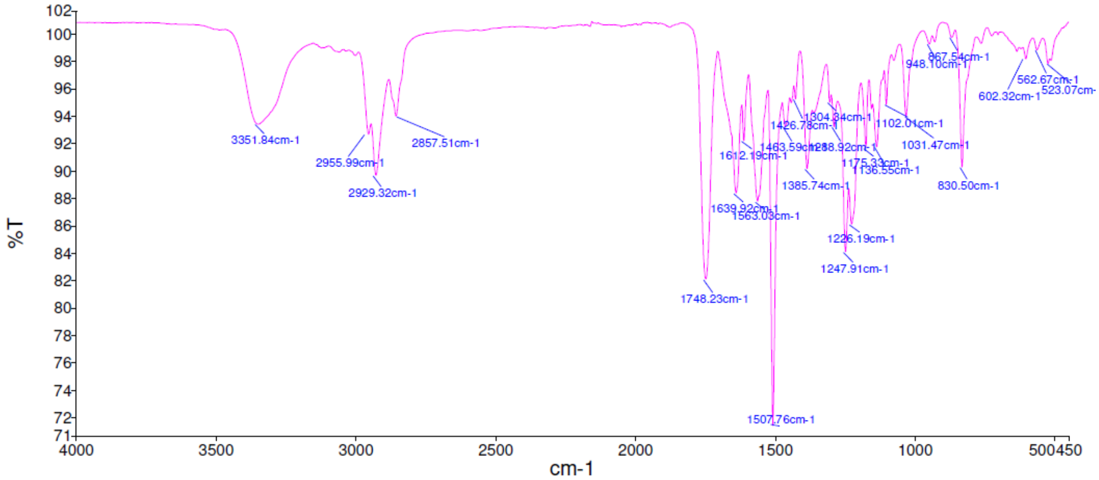

Figure S130. IR spectra of compound **4b**.

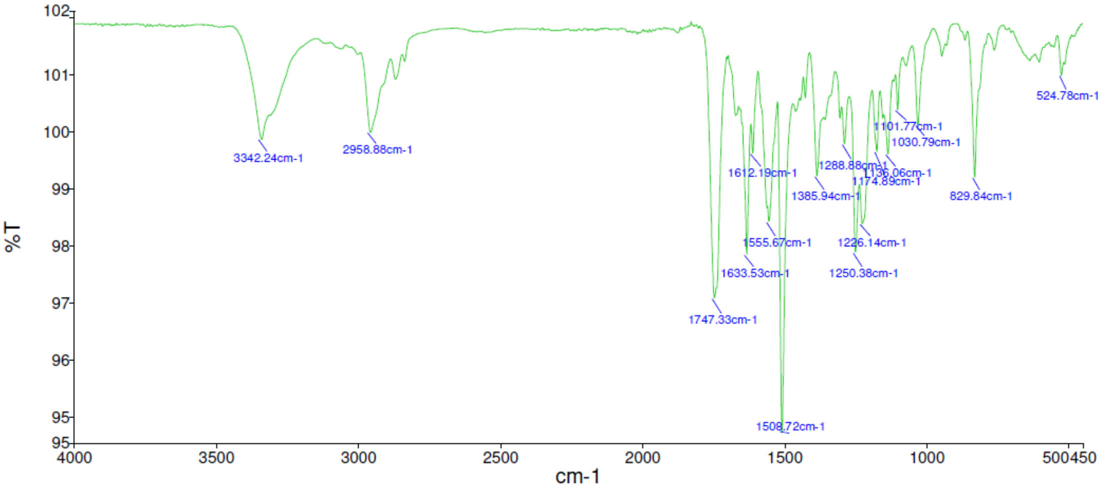

Figure S131. IR spectra of compound **4c**.

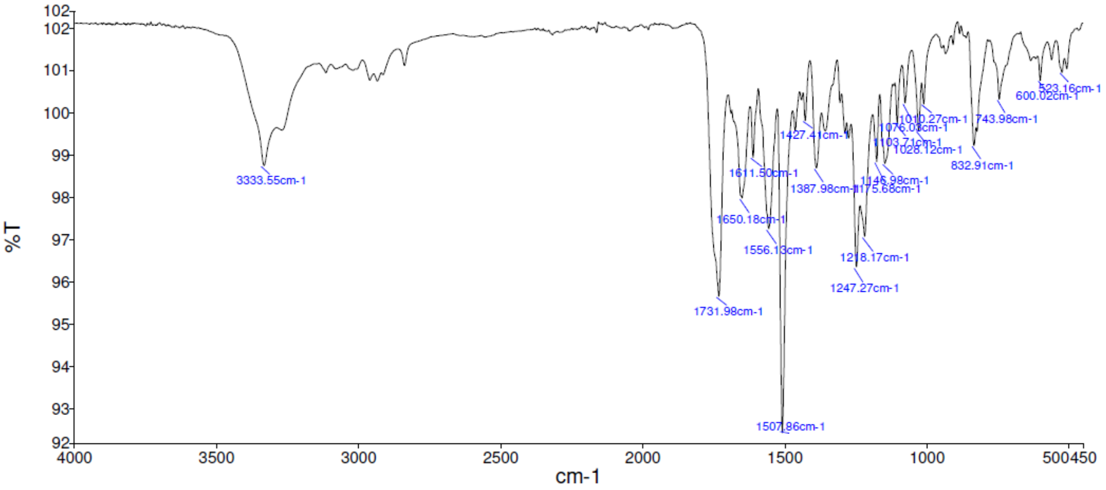

Figure S132. IR spectra of compound **4d**.

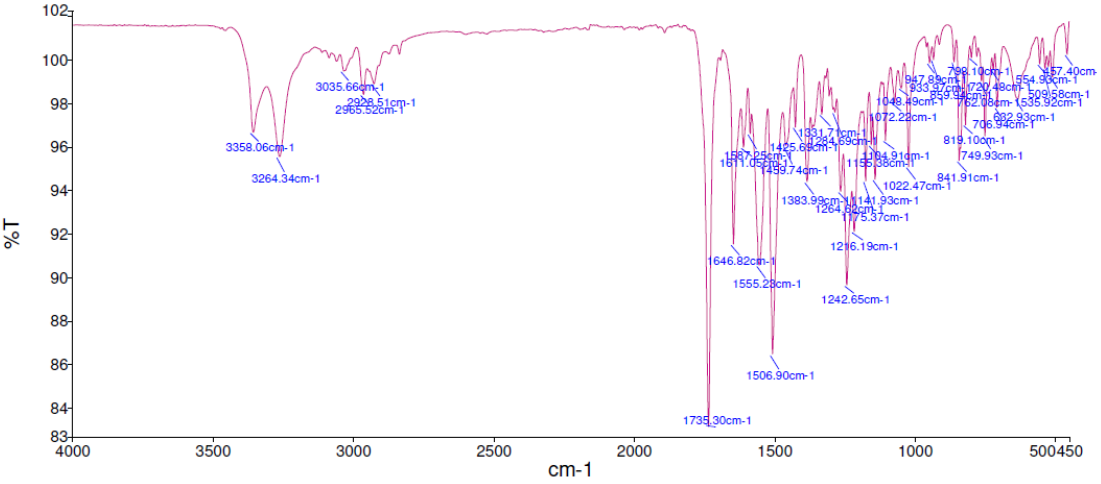

Figure S133. IR spectra of compound **4e**.

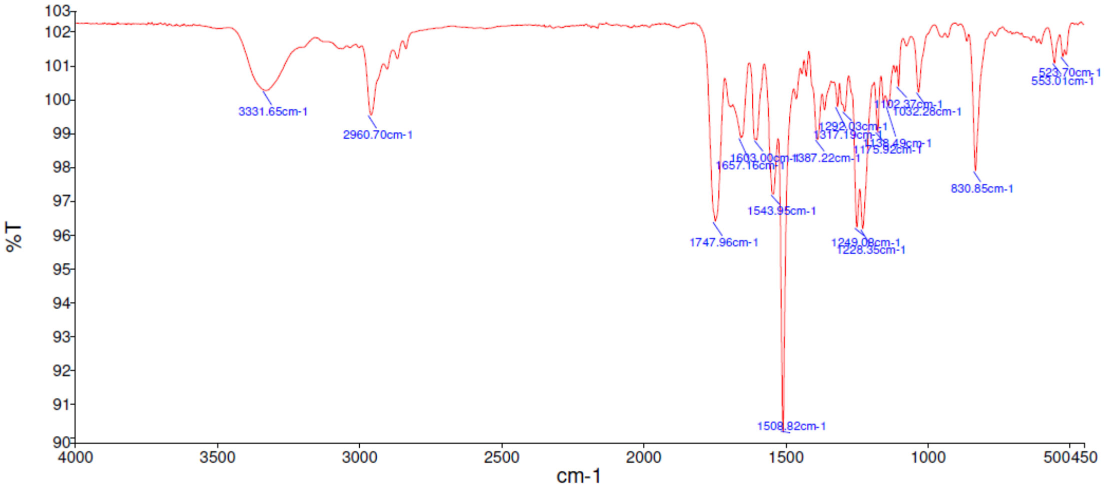

Figure S134. IR spectra of compound **4f**.

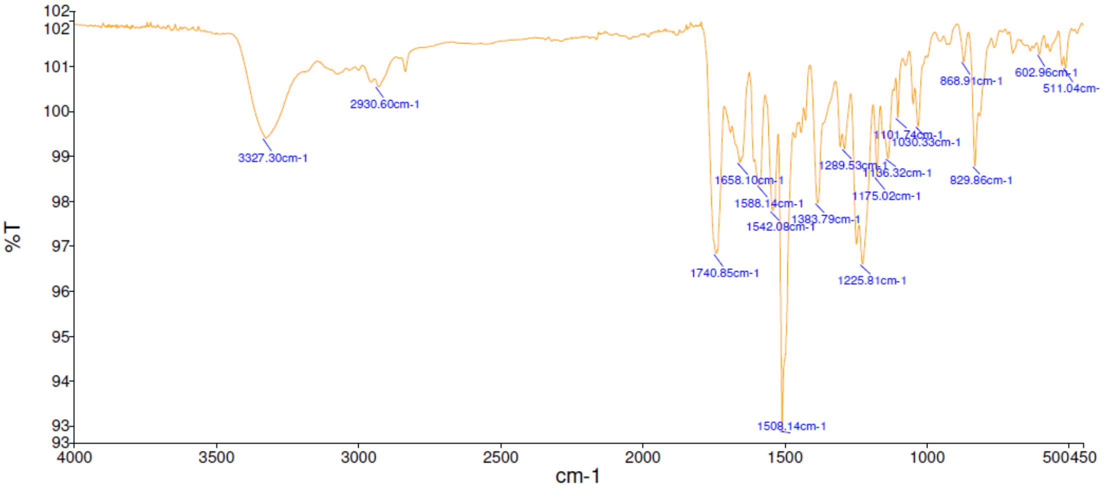

Figure S135. IR spectra of compound **4g**.

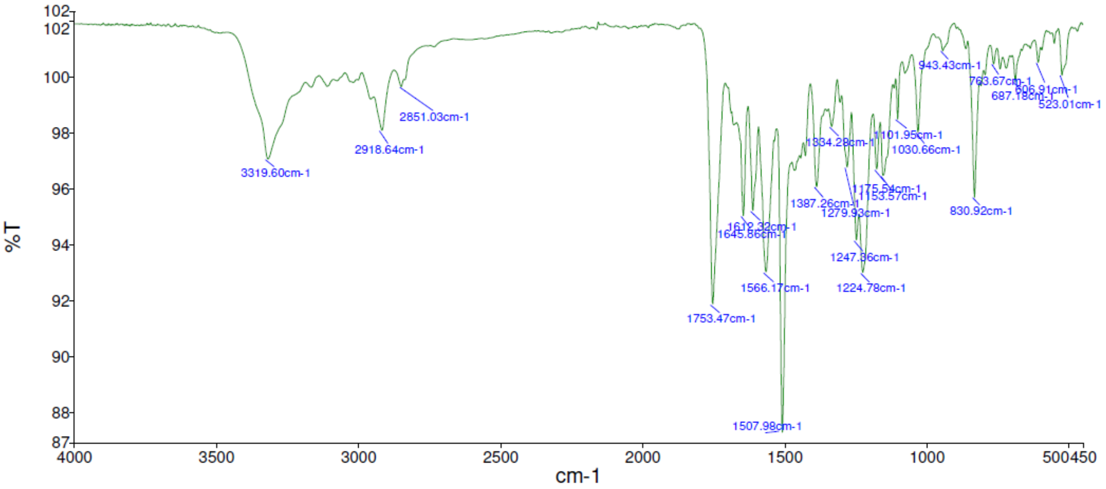

Figure S136. IR spectra of compound **4h**.

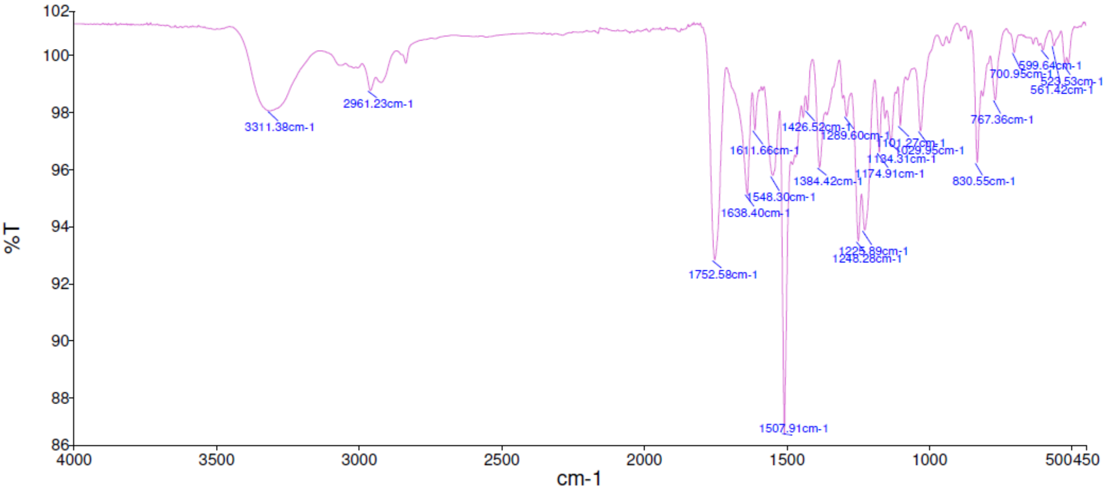

Figure S137. IR spectra of compound **4i**.

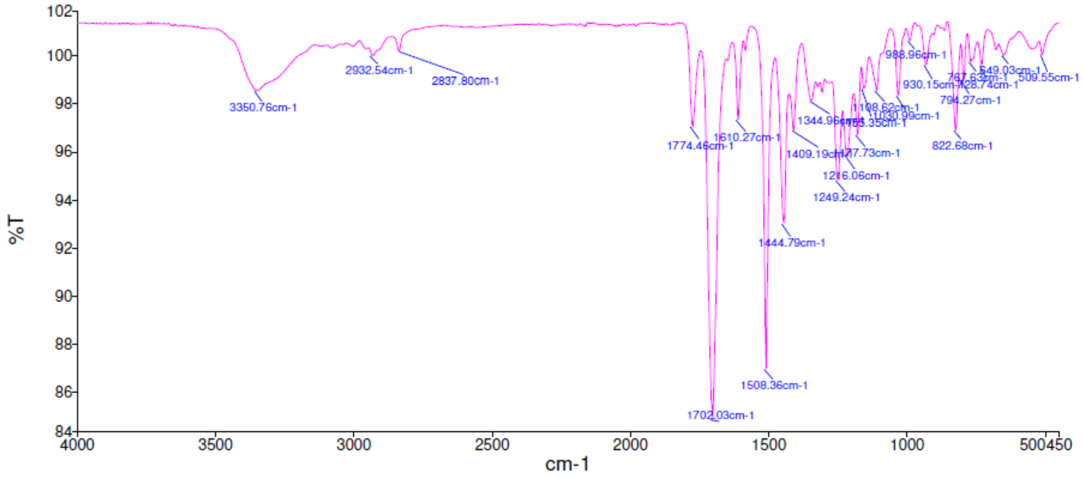

Figure S138. IR spectra of compound *syn*/*anti*-**5a**.

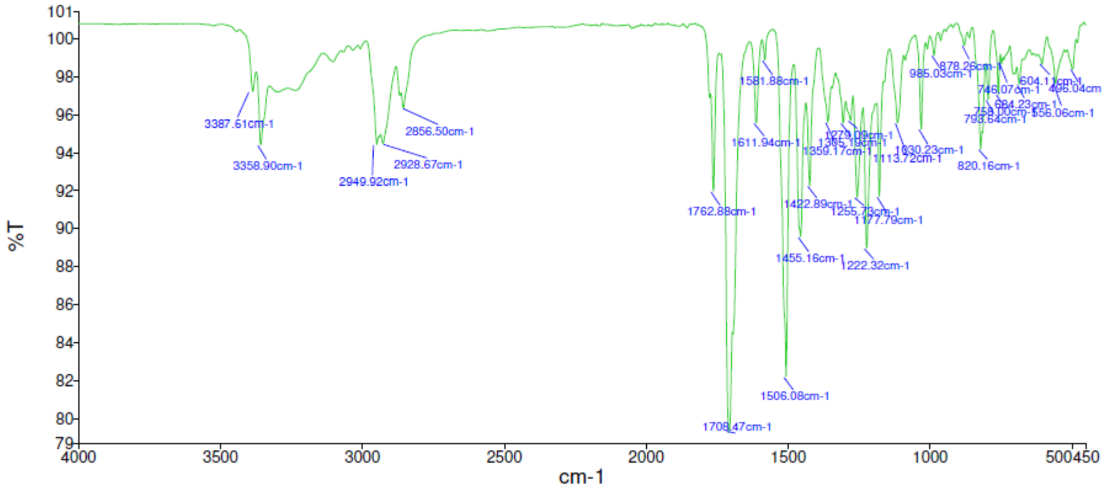

Figure S139. IR spectra of compound *syn*/*anti*-**5b**.

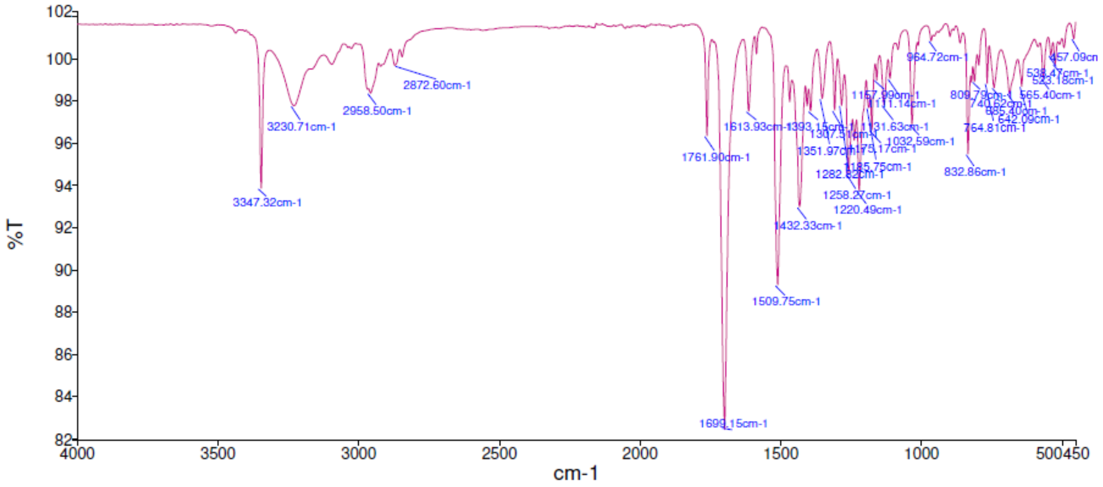

Figure S140. IR spectra of compound *syn*/*anti*-**5c**.

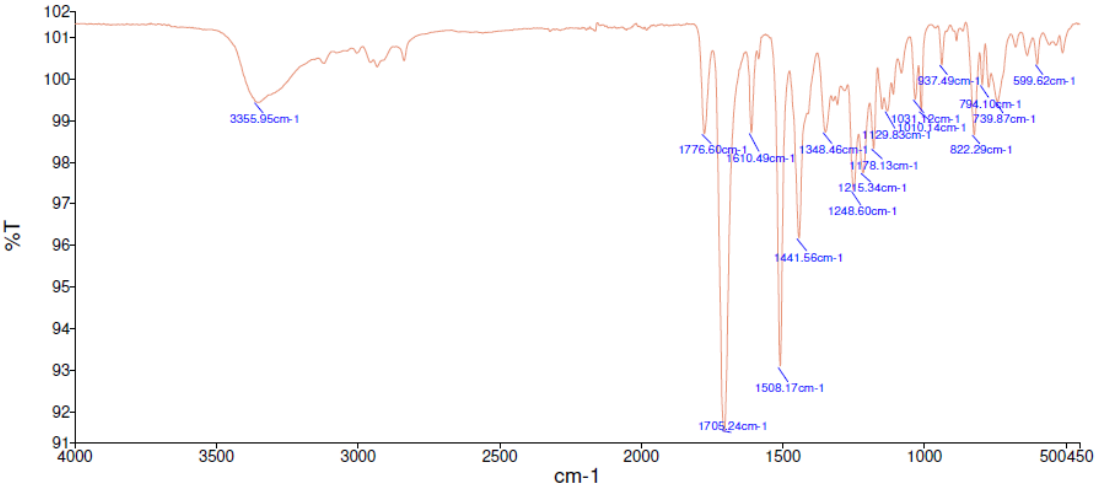

Figure S141. IR spectra of compound *syn*/*anti*-**5d**.

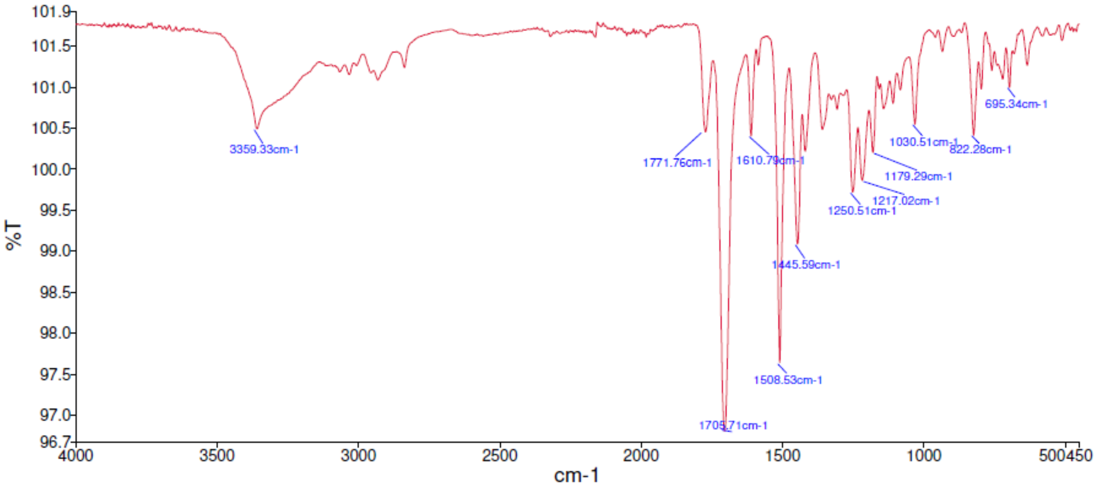

Figure S142. IR spectra of compound *syn*/*anti*-**5e**.

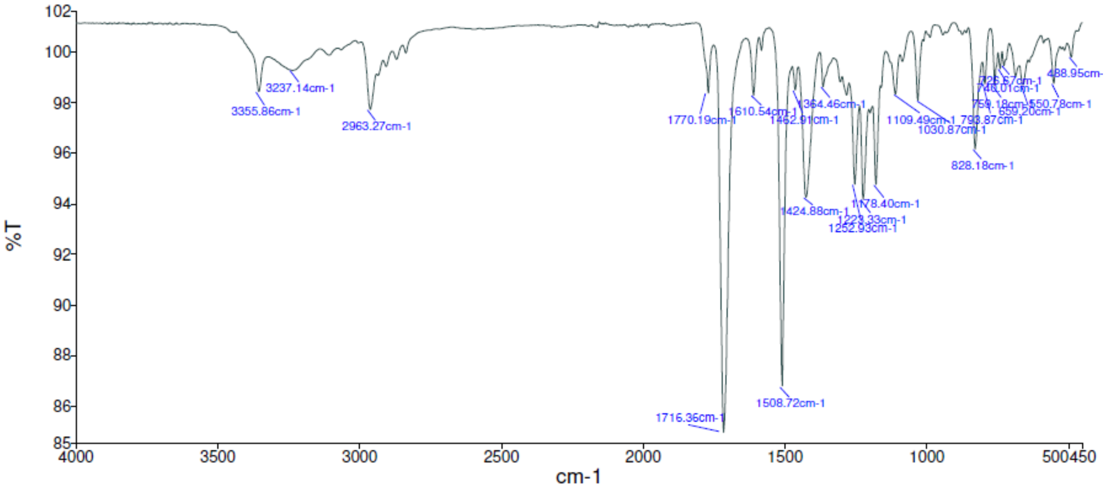

Figure S143. IR spectra of compound *syn*/*anti*-**5f**.

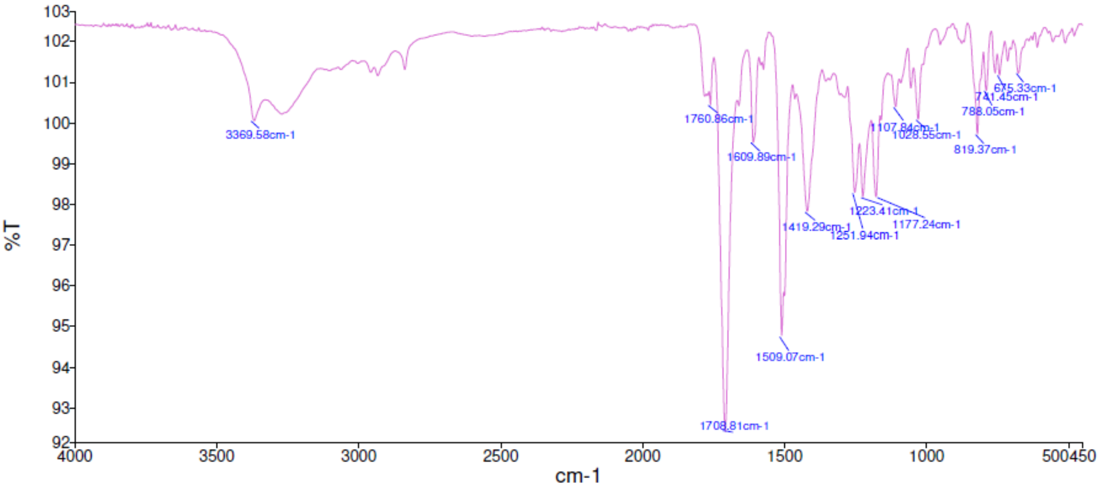

Figure S144. IR spectra of compound *syn*/*anti*-**5g**.

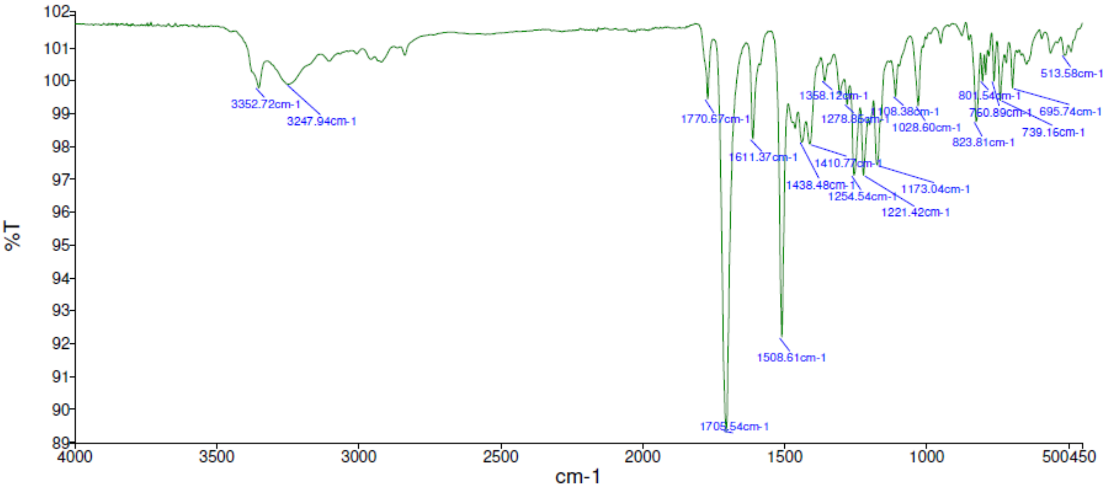

Figure S145. IR spectra of compound *syn*/*anti*-**5h**.

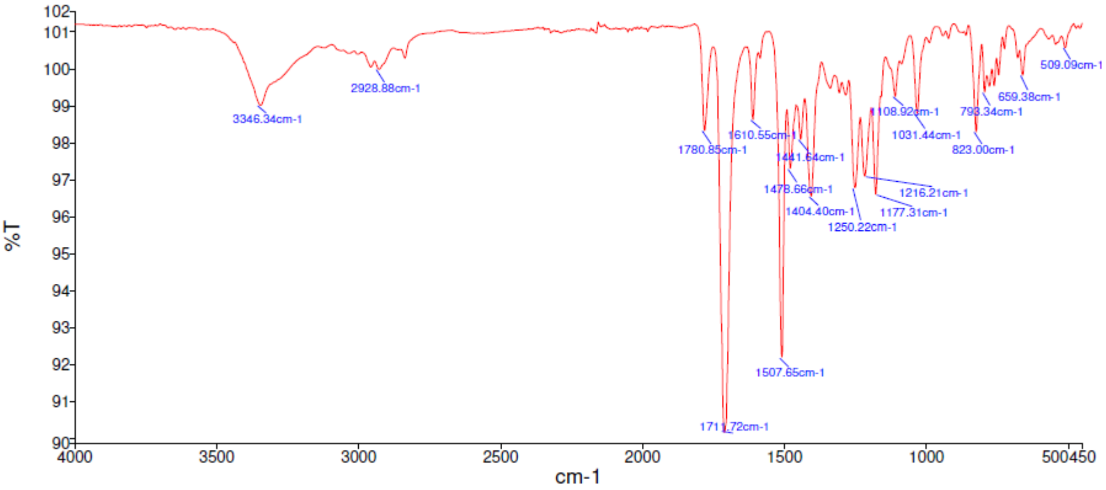

Figure S146. IR spectra of compound *syn*/*anti*-**5i**.

7. DFT and TD-DFT calculations

Table S5. Structures, relative energy (kcal/mol) and Boltzmann populations of low-energy minima calculated for (5*S*,6*S*)-**5a** at B3LYP-D3BJ/6-311+G(d,p)/PCM level.

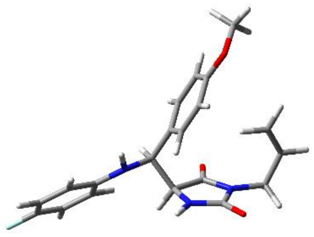 Abs. Min. (26.3%)	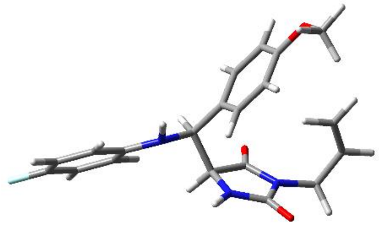 +0.06 (23.7%)	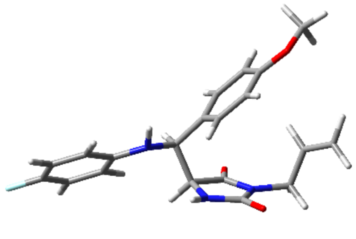 +0.52 (10.9%)
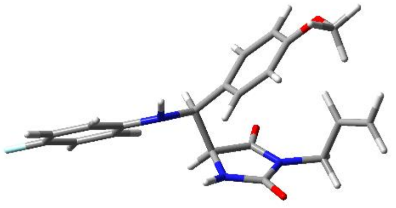 +0.54 (10.6%)	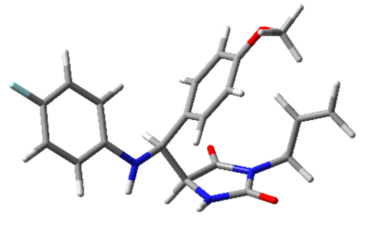 +0.65 (8.8%)	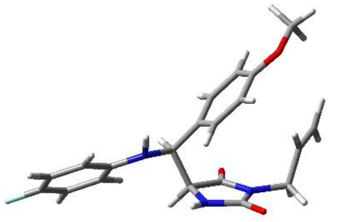 +0.66 (8.6%)
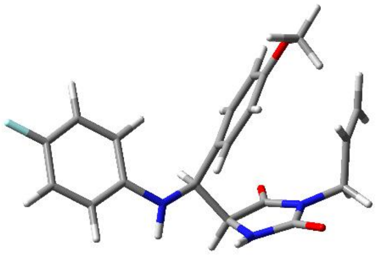 +0.74 (7.5%)	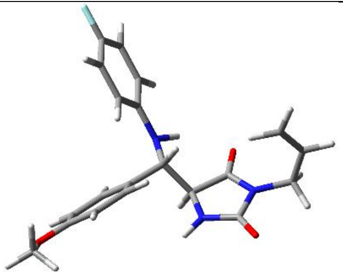 +1.18 (3.6%)	

Table S6. Structures, relative energy (kcal/mol) and Boltzmann populations of low-energy minima calculated for (5*R*,6*S*)-**5a** at B3LYP-D3BJ/6-311+G(d,p)/PCM level.

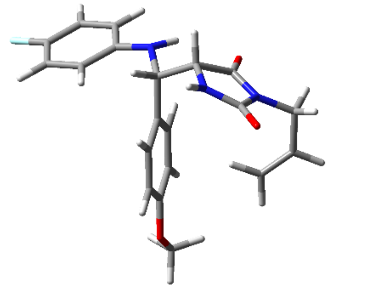 Abs. Min. (40.4%)	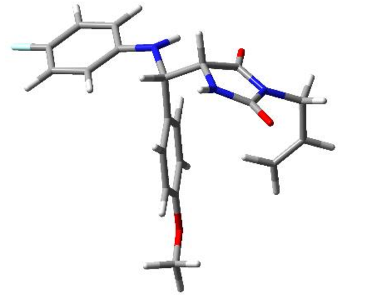 +0.08 (35.1%)	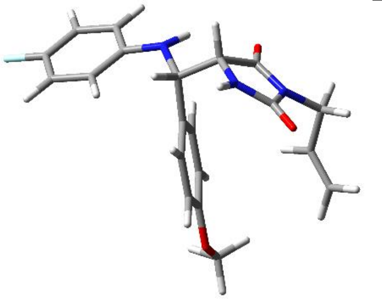 +0.86 (9.5%)
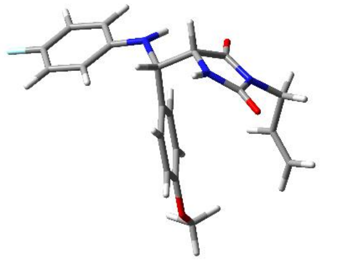 +0.92 (8.6%)	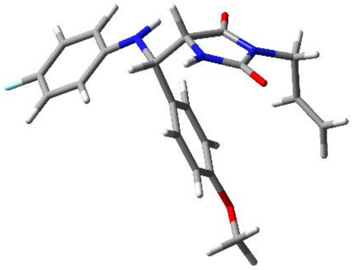 +1.09 (6.4%)	

Table S7. ECD simlarity factors [1] calculated for the range 190-290 nm, and VCD simlarity factors calculated for the range 1500-1200 cm^{-1}.

**ECD similarity factors**	**5a-ent1**	**5a-ent2**	**5a-ent3**	**5a-ent4**
(5*R*,6*S*)-**5a** B3LYP[(a)]	0.757	0.120	0.035	**0.876**
(5*R*,6*S*)-**5a** CAM-B3LYP[(b)]	0.758	0.099	0.018	0.865
(5*S*,6*S*)-**5a** B3LYP[(a)]	**0.855**	0.029	0.080	0.562
(5*S*,6*S*)-**5a** CAM-B3LYP[(b)]	0.698	0.252	0.148	0.027

**VCD similarity factors**	**5a-ent3**	**5a-ent4**
(5*R*,6*S*)-**5a** B3LYP-D3BJ[(c)]	0.077	**0.646**
(5*S*,6*S*)-**5a** B3LYP-D3BJ[(c)]	0.550	0.074

[(a)] B3LYP/def2-TZVP/PCM; [(b)] CAM-B3LYP/def2-TZVP/PCM; [(c)] B3LYP-D3BJ/6-311+G(d,p)/PCM

Table S8. Structures, relative energy (kcal/mol) and Boltzmann populations of low-energy minima calculated for the truncated model of (5*S*,6*S*)-**5a** at B3LYP-D3BJ/6-311+G(d,p)/PCM level.

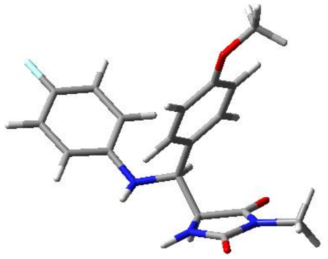<br>Abs. Min. (24.9%)	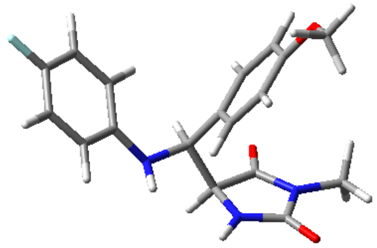<br>+0.03 (23.8%)	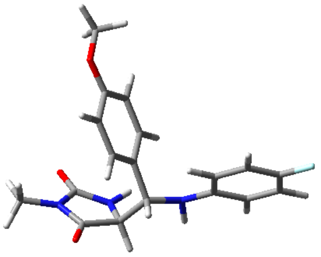<br>+0.55 (9.9%)
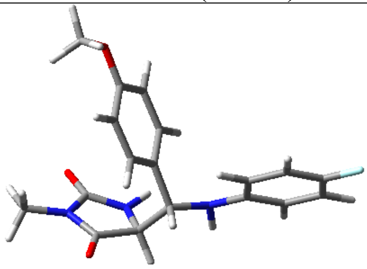<br>+0.58 (9.3%)	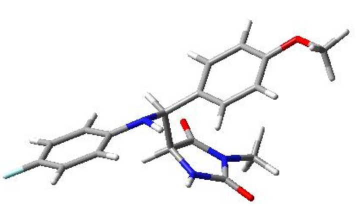<br>+0.71 (7.5%)	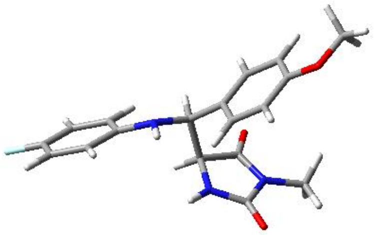<br>+0.74 (7.1%)

Table S9. Structures, relative energy (kcal/mol) and Boltzmann populations of low-energy minima calculated for the truncated model of (5*R*,6*S*)-**5a** at B3LYP-D3BJ/6-311+G(d,p)/PCM level.

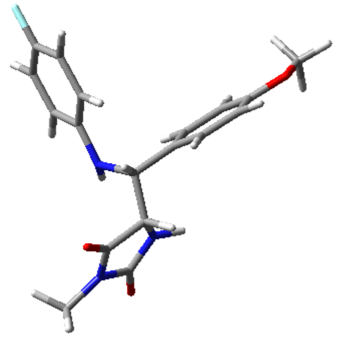<br>Abs. Min. (30.4%)	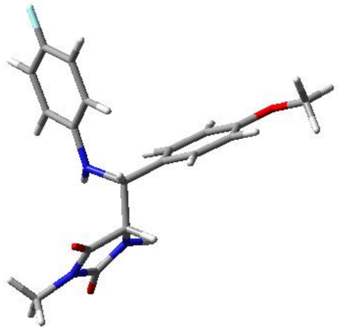<br>+0.01 (29.7%)	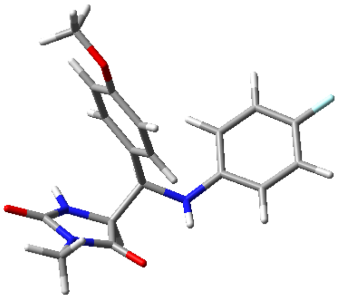<br>+0.38 (16.1%)
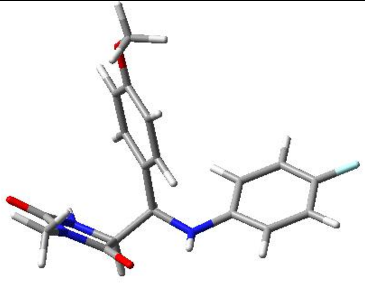<br>+0.38 (16.1%)		

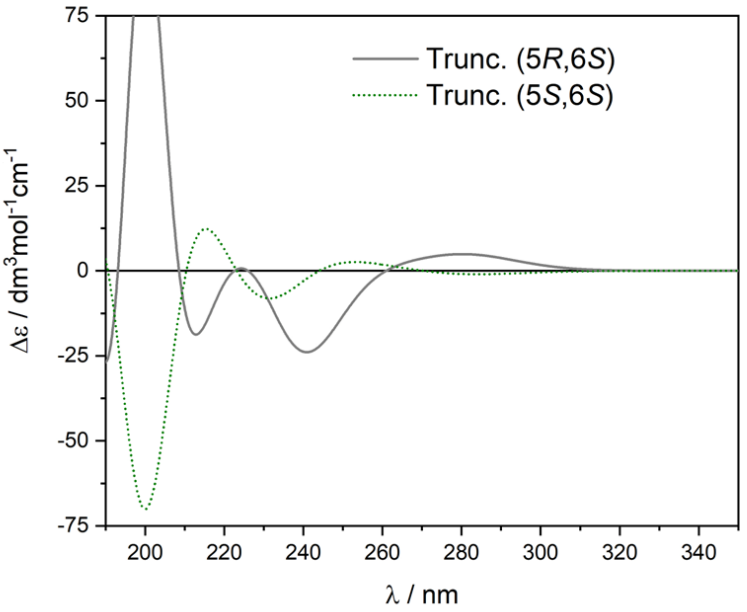

Figure S147. ECD spectra calculated for the truncated analogs of (5*S*,6*S*)-**5a** and (5*R*,6*S*)-**5a** (with the allyl group replaced by a methyl) at TD-B3LYP/def2-TZVP/PCM//B3LYP-D3BJ/6-311+G(d,p) level. Plotting parameters UV shift +15 nm, σ = 0.3 eV.

8. *In silico* physicochemical and biological profiling

Table S10. Prediction of Lipinski's rule of five properties for the hydantoins **5a–i**.

**Compound** *syn/anti*	**MW**	**LogP**	**HBA**	**HBD**	**Lipinski violation**
**5a**	369.40	2.33	6	2	0
**5b**	413.50	4.00	6	2	0
**5c**	397.45	3.05	6	2	0
**5d**	409.42	2.70	7	2	0
**5e**	419.46	3.46	6	2	0
**5f**	461.54	5.01	6	2	1, LP
**5g**	453.90	4.54	6	2	0
**5h**	433.49	4.26	6	2	0
**5i**	433.49	4.04	6	2	0

MW – molecular weight; **logP** – the octanol-water partition coefficient; **HBA** – number of hydrogen bond accepting O and N atoms; **HBA** – number of hydrogen bond donating OH and NH atoms. Parameters were calculated by ADMET Predictor [2].

Table S11. ADMET properties of the hydantoins **5a–i** calculated by ADMET Predictor and admetSAR.

ADMET properties prediction	5a	5b	5c	5d	5e	5f	5g	5h	5i
**TPSA**[a]	70.67	70.67	70.67	83.81	70.67	70.67	70.67	70.67	87.74
**RB**[a]	8	11	7	8	8	8	7	7	7
**Sw** (mg/mL)[a]	0.58	0.14	0.28	0.19	0.06	0.01	0.01	0.02	0.01
**MDCK** (cm/s × 10^7)[a]	564.52	474.97	472.37	418.56	527.66	397.98	625.91	471.00	577.37
**Peff** (cm/s × 10^4)[a]	2.61	2.13	2.79	1.97	2.54	2.48	2.75	2.98	3.07
**BBB_filter**[a]	Low	Low	Low	Low	Low	Low	Low	Low	Low
**LogBB**[a]	0.01	0.16	0.20	-0.38	0.09	0.44	0.45	0.48	0.16
**Pgp sub**[a]	No	Yes	Yes	Yes	Yes	Yes	Yes	Yes	Yes
**Pgp inh**[a]	No	Yes	Yes	No	No	Yes	No	No	No
**hum_fup (%)**[a]	13.48	6.52	9.00	9.91	6.36	4.36	4.12	5.63	4.69
**%Fumic**[a]	71.47	25.46	52.43	62.20	40.24	6.52	13.74	19.24	24.39
**CYP2C9 sub**[b]	No	No	No	No	No	No	Yes	Yes	Yes
**CYP2D6 sub**[b]	No	No	No	No	No	No	No	No	No
**CYP3A4 sub**[b]	Yes	Yes	Yes	Yes	Yes	Yes	Yes	Yes	Yes
**CYP1A2 inh**[b]	No	No	No	No	No	No	No	No	No
**CYP2C9 inh**[b]	No	No	No	No	No	No	No	No	No
**CYP2C19 inh**[b]	No	Yes	No	No	No	No	No	No	No
**CYP2D6 inh**[b]	No	No	No	No	No	No	No	No	No
**CYP3A4 inh**[b]	Yes	Yes	No	Yes	Yes	Yes	No	Yes	Yes
**Rat_TD50**[a] (mg/kg/day)	23.31	18.29	29.85	9.71	7.85	9.34	12.19	12.73	8.88
**Mouse_TD50**[a] (mg/kg/day)	208.16	260.38	234.04	125.26	129.50	265.95	198.32	395.22	130.61
**TOX_Code**[a]	No	No	No	No	No	No	No	No	No
**hERG_Filter**[a]	No	No	No	No	No	Yes	Yes	Yes	Yes

TPSA – topological polar surface area; **RB** – number of rotable bonds; **Sw** – water solubility; **MDCK** – permeability through Madin-Darby canine kidney cell layers; **Peff** – human effective jejunal permeability; **BBB_filter-** qualitative likelihood (High/Low) of crossing the blood-brain barrier; **logBB** – logarithm of the brain/blood partition coefficient; **Pgp sub** – yes/no classification for P-glycoprotein substrate; **Pgp inh-** yes/no classification for P-glycoprotein inhibitor; **hum_fup%** – percent unbound to blood plasma proteins in human; **%Fumic** – fraction unbound in human liver microsomes. **CYP2C9 sub** – cytochrome P450 2C9 substrate; **CYP2D6 sub** – cytochrome P450 2D6 substrate; **CYP3A4 sub** – cytochrome P450 2D6 substrate; **CYP1A2 inh** – cytochrome P450 1A2 inhibitor; **CYP2C9 inh** – cytochrome P450 2C9 inhibitor; **CYP2C19 inh** – cytochrome P450 2C19 inhibitor; **CYP2D6 inh** – cytochrome P450 2D6 inhibitor; **CYP3A4 inh** – cytochrome P450 3A4 inhibitor; **Rat_TD50** –chronic toxic dose (TD50) for rats; **Mouse_TD50** – chronic TD50 for mice; **hERG_Filter** – qualitative yes/no estimation of the likelihood of inhibition the hERG potassium channel; **TOX_Code** – hERG toxicity, acute toxicity in rats (Rat_ Acute < [200,300]), carcinogenicity in chronic mouse studies (Xr Mouse_TD50 < [25,40]); hepatotoxicit< HEPX - liver enzymes elevated in serum (aspartate transaminase (AST), alanine transaminase (ALT) and lactate dehydrogenase (LDH)) toxicity liabilities and mutagenicity (MUT_risk >1).

[a] calculated by ADMET Predictor [2].

[b] calculated by admetSAR web server [3].

9. References

1. T. Bruhn, A. Schaumlöffel, Y. Hemberger, G. Bringmann, SpecDis: Quantifying the Comparison of Calculated and Experimental Electronic Circular Dichroism Spectra, *Chirality* **2013**, *25*, 243-249.
2. M. S. Lowless, M. Waldman, R. Franczkiewicz and R. D. Clark, *Using chemoinformatics in drug discovery*, in: *New Approaches to Drug Discovery, Handbook of Experimental Pharmacology* (Eds. U. Nielsch, U. Fuhrmann and S. Jaroch), Springer Int. Publ. Switzerland, Cham 2016, Vol. 232, pp. 232, 139–170.
3. H. Yang, C. Lou, L. Sun, J. Li, Y. Cai, Z. Wang, W. Li, G. Liu and Y. Tang, admetSAR 2.0: web-service for prediction and optimization of chemical ADMET properties, *Bioinformatics* **35**(6) (2019) 1067–1069; https://doi.org/10.1093/bioinformatics/bty707
